# Intrastrand triplex DNA repeats in bacteria: a source of genomic instability

**DOI:** 10.1093/nar/gkv1017

**Published:** 2015-10-07

**Authors:** Isabelle T. Holder, Stefanie Wagner, Peiwen Xiong, Malte Sinn, Tancred Frickey, Axel Meyer, Jörg S. Hartig

**Affiliations:** 1 Department of Chemistry and Konstanz Research School Chemical Biology (KoRS-CB), University of Konstanz, Universitätsstrasse 10, 78457 Konstanz, Germany; 2 Department of Biology, University of Konstanz, Universitätsstrasse 10, 78457 Konstanz, Germany

## Abstract

Repetitive nucleic acid sequences are often prone to form secondary structures distinct from B-DNA. Prominent examples of such structures are DNA triplexes. We observed that certain intrastrand triplex motifs are highly conserved and abundant in prokaryotic genomes. A systematic search of 5246 different prokaryotic plasmids and genomes for intrastrand triplex motifs was conducted and the results summarized in the ITxF database available online at http://bioinformatics.uni-konstanz.de/utils/ITxF/. Next we investigated biophysical and biochemical properties of a particular G/C-rich triplex motif (TM) that occurs in many copies in more than 260 bacterial genomes by CD and nuclear magnetic resonance spectroscopy as well as *in vivo* footprinting techniques. A characterization of putative properties and functions of these unusually frequent nucleic acid motifs demonstrated that the occurrence of the TM is associated with a high degree of genomic instability. TM-containing genomic loci are significantly more rearranged among closely related *Escherichia coli* strains compared to control sites. In addition, we found very high frequencies of TM motifs in certain *Enterobacteria* and *Cyanobacteria* that were previously described as genetically highly diverse. In conclusion we link intrastrand triplex motifs with the induction of genomic instability. We speculate that the observed instability might be an adaptive feature of these genomes that creates variation for natural selection to act upon.

## INTRODUCTION

Nucleic acid repeat sequences have the potential to fold into alternate or non-canonical structures in genomic DNA, though *in* *vivo* data remain unclear. Prokaryotic repeats have been classified according to different criteria such as size, genomic distribution, coding capability and their abundance in the genome. Examples of different categories are simple sequence repeats (SSR), tandem repeats (TR), miniature inverted repeats (MITE), repetitive extragenic palindromic sequences (REP) and clustered regularly interspaced short palindromic repeats (CRISPRs). The 20–48 bp long CRISPR (1) repeats have been shown to play a role in the adaptive immune response of bacteria. REPs are palindromic, 20–40 bp long DNA repeats that can occur as single units or in clusters (BIMEs: bacterial interspersed mosaic elements) (2,3). MITEs are generally <200 bp in length and require a transposase for transposition. They can fold into long stem-looped structures on the RNA level and frequently carry functional motifs such as promoter sequences or protein binding sites (4,5). TRs contain multiple units that are directly repeated in a head-to-tail manner and span from 1–100 bp (6,7) (1–9, 10–100 and >100 bp unit size are termed micro-, mini- and macrosatellites, respectively). They are found in a variety of prokaryotic species (8,9) with great differences existing even among closely related species (10). Microsatellites with a length of 1–6 bp—short repeats, termed SSRs (11)—are utilized for phase variation in bacterial adaptation (12,13). High mutation rates at repeat sites frequently result in expansion or contraction of the SSRs, causing expression changes of associated genes. Often, phase variation specifically switches ON or OFF factors involved in the interaction with the host, such as the invasiveness or the adherence to host cells (14–16). Most repeats concentrate in intergenic regions up to 200 bp upstream of the start codon.

Nucleic acid repeats have the potential to have pronounced effects on the local DNA structure within genomes. They are prone to fold into hairpins or even more complex structures. Sequences with the potential to adopt such non-canonical nucleic acid structures are abundant in eukaryotic and prokaryotic genomes. Several types of such alternate structures are known since the late 1950s (17). They can form by branching, contain looped-out bases or adopt a left-handed Z-DNA conformation. Even more complex structures occur when more than two strands interact with each other. For example, in triplex and quadruplex motifs additional interactions are found via the Hoogsteen face of involved nucleobases. Both structures can form inter- or intramolecularly. Interestingly, repetitive sequences as well as non-B-DNA structures have been associated with genomic instability. Inverted repeats were found to cause deletions in *Escherichia coli* as early as the 1980s (18–20).

**Figure 1.**
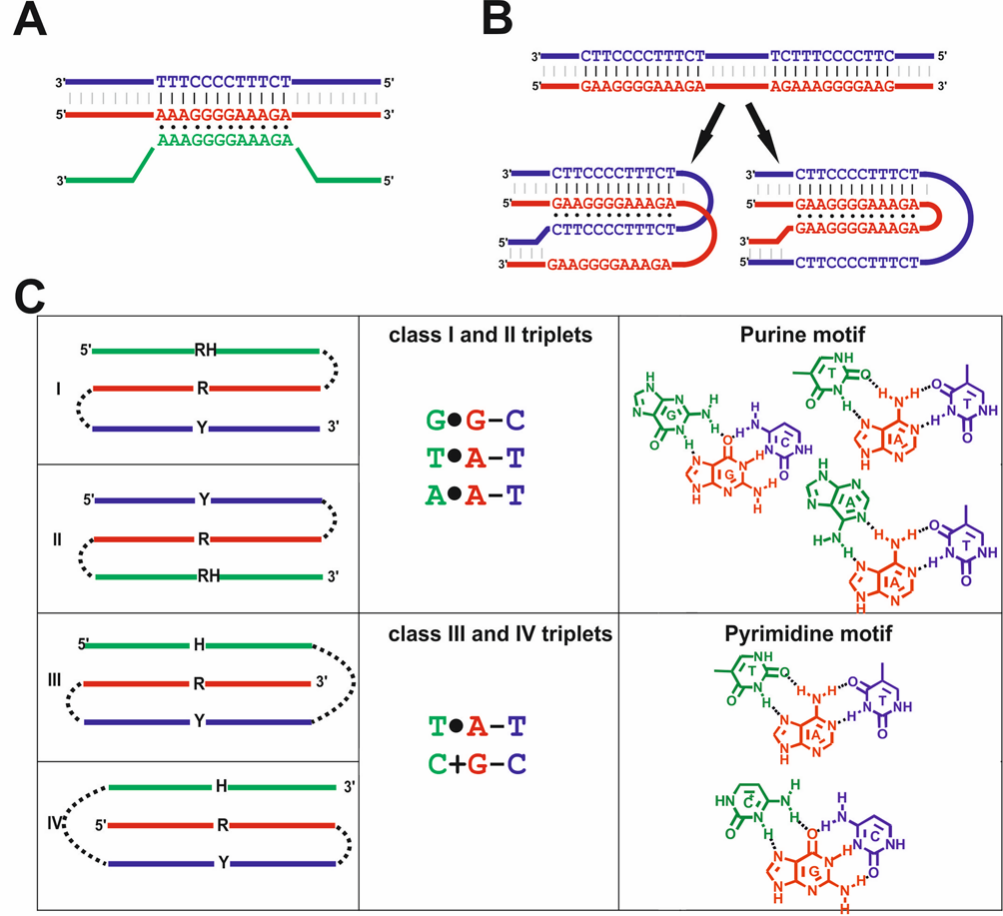
Overview of triplex motifs. (**A**) Diagrammatic presentation of an intermolecular purine motif triplex with antiparallel strand orientation formed by three distinct DNA strands. (**B**) Examples of intramolecular H-DNA structures that can form from a mirror repeat sequence within a DNA double strand. Pyrimidine motif H-DNA with parallel strand orientation is shown on the left side. Purine motif H-DNA with antiparallel orientation is shown on the right side. Pyrimidine-rich strands are shown in blue, purine-rich strands are depicted in red and the reverse Hoogsteen strand is in green; Figure modified from (25). (**C**) Description of intrastrand triplex motifs. The first column contains schematics of the four intrastrand triplex classes; pyrimidine rich (Y) regions are shown in blue, purine rich (R) regions are depicted in red and (reverse) Hoogsteen ((R)H) regions are colored green (modified from (22)). Dashed lines represent arbitrary spacer sequences. The second column shows the potential nucleotide composition of the DNA that may fold into the respective triplex class. The three nucleotides listed refer to the most frequent nucleotides in the respective region. The corresponding nucleotide triplets are shown in the third column. Purine motif triplexes: classes I and II; pyrimidine motif triplexes: Classes III and IV.

Here we investigate a wide-spread and highly conserved sequence with the potential to form an intrastrand triple helical structure. Triple helical nucleic acids were first described in 1957 (21). Triplex structures form between three DNA strands. They occur in purine-rich DNA strands that form Hoogsteen or reverse-Hoogsteen hydrogen bonds in addition to the Watson–Crick basepairs. Two different triplex motifs have been described: the purine motif (R) and the pyrimidine motif (Y) (22). Both require a homo-purine stretch within a Watson–Crick duplex that binds a third strand via the major groove. In the R motif the third strand has an antiparallel orientation to the duplex purine strand and forms A(T)AT and GGC triplexes. The Y motif contains TAT and CGC triplets and has the third strand in parallel orientation (Figure 1). Intermolecular structures are formed from two or three distinct DNA strands—most often between a DNA duplex and a single stranded triplex-forming oligonucleotide (TFO, Figure 1A) (23). Intermolecular triplexes have been used for the artificial regulation of gene expression and may be suitable for therapeutic use (24,25). They have been reported to block protein–DNA interactions (26) and influence site directed recombination (27). In intramolecular triplexes the third strand is physically tethered to the DNA duplex or the structure occurs in one single DNA strand. Most studies investigating intramolecular triplexes focus on H-DNA (Figure 1B). In H-DNA the homo-purine/homo-pyrimidine sequence must be a mirror repeat. That way, half of the pyrimidine tract swivels its backbone to the purine strand of the duplex or the purine strand binds to the purine part of the underlying duplex, forming either a parallel or antiparallel H-DNA structure (28). Four different H-DNA isoforms exist, depending on whether the 3′ half or the 5′ half of the third strand is participating in triplex structure formation. H-DNA has been reported to induce genetic instability and to influence DNA replication, repair and transcription (29). Computational studies revealed that natural sequences with the potential to adopt an H-DNA structure are very abundant in mammalian cells (30). Although only few of those sequences were found so far in prokaryotic species (30), long oligopurine/oligopyrimidine tracts have been discovered in bacterial genomes near regulatory regions (31). Evidence for the *in vivo* existence of triplex DNA structures is increasing—immunodetection by triple-helix-specific antibodies has been reported (32–34).

A different type of intramolecular triplex structure arises from the folding of polypurine/polypyrimidine units along one single strand of DNA. Although those intrastrand triplexes (Figure 1C) have been investigated *in vitro* (35–40), studies investigating their *in vivo* occurrence (22) and function (41) are sparse. Intrastrand triplex motifs can occur in four different conformational classes, depending on their strand orientation (Figure 1C) (22). Here, class I and II refer to purine motif triplexes, with class I having the reverse Hoogsteen domain at the 5′ terminus, followed by the purine- and the pyrimidine-rich domain. Class II triplexes have the pyrimidine-rich domain at the 5′ end, followed by the purine rich domain and the reverse Hoogsteen domain at the 3′ end of the sequence. Class III and IV correspond to the Y motif triplex structures: class III triplexes progress from the 5′ purine-rich domain through the pyrimidine-rich domain to the 3′ Hoogsteen domain and class IV triplexes start with the Hoogsteen domain at the 5′ terminus, followed by the pyrimidine-rich domain and ending with the purine rich domain at the 3′ terminus. It has been shown that both purine- and pyrimidine-type triplex DNA structures can form under physiological conditions for isolated single strands. Pyrimidine-type intrastrand triplex structures even occur in RNA form (42), while purine motif triple helices cannot form in RNA (43). *In vivo* studies revealed that distinct R-type triplex DNA structures can lead to polymerase arrest during replication (44,45).

In this study, we focused on a particular triplex motif, earlier described as BoxC. In the 1990s the BoxC motif was found as a palindromic repeat in the *E. coli* genome (46,47) without further characterization and re-discovered as potential intrastrand-forming triplex (PIT) element by Maher *et* *al*. in 2000 (22). They identified this particular sequence in three different bacterial genomes (*E. coli, Synechocystis sp. and H. influenzae*). Furthermore, they characterized the BoxC triplex motif in *E. coli* and found no significant correlation of the orientation and function of genes that were associated with the triplex motifs. Based on UV and electrophoresis mobility shift assays, they proposed a triplex structure of the corresponding isolated oligonucleotide. In a follow-up study Maher *et* *al*. aimed at clarifying potential roles of the PIT elements. Although they showed that PIT elements are able to block DNA polymerase elongation *in vitro*, they found no effect in *in vivo* studies. Furthermore, the PIT elements did not display promoter and terminator activity and did not affect RNA polymerase and reverse transcriptase activities, nor did it interfere with conjugation.(41).

Here we investigate triplex sequences formerly recognized as BoxC or PIT elements. Due to the increasing availability of genomic data we were able to compare and discover a high degree of sequence conservation and a evolutionarily wide-spread occurrence of intrastrand triplex sequences. We developed a search algorithm for the identification of potential intrastrand triplex motifs and provide a novel database of triplex sequences in more than 5000 bacterial genomes and plasmids. We characterized a specific sequence that occurs in *E. coli* by circular dichroism (CD) and nuclear magnetic resonance (NMR) and showed that indeed stable triplex formation occurs *in vitro*. *In vivo* footprinting experiments suggested the triplex to be formed only to a minor extent or only temporarily in the cell. Due to the high conservation and abundance of the motif, we considered other functions than have been investigated before. When we compared the occurrence of triplex motifs among closely related strains within *E. coli*, we noted an unusually high variability of triplex-associated genetic contexts. Utilizing comparative BLAST analyses of 56 sequenced *E. coli* substrains, we found pronounced genetic instability associated with the described triplex motifs. In addition to *E. coli*, we noted a high prevalence of intrastand triplexes in genomes of bacterial species that have been described as genetically highly variable such as certain Cyano- and Enterobacteria. Hence the characterized triplex motifs appear to act as genetic diversification processes that might provide increased evolutionary potential to bacterial communities.

## MATERIALS AND METHODS

### Bacterial strains and growth conditions

Bacteria were routinely grown in LB medium supplemented with 50 μg/ml streptomycin for plasmid selection if necessary. *E. coli* BW19610 (48) was used for cloning, plasmid purification and maintenance. To generate an *E. coli* strain carrying a mutated 6′ TM, bp 281075–281622 of *E. coli* K-12 MG1655 were amplified introducing point mutations by overlapping polymerase chain reaction using primer pairs SW09 (5′-ACGCGTCGACAGCCGGTGGCAGGTG-3′)/SW10 (5′-CGCTCACGCCGGCGCTCTCGGCAAAGGGGCGAGGGGGAAAAGATG-3′) and SW11 (5′-TTTGCCGAGAGCGCCGGCGTGAGCGGCAATATGTGATCCAGC-3′)/SW12 (5′-GCTCTAGACGCCTGCTTTGATC-3′) and cloned into pKNG101 (49) using restriction sites SalI/XbaI. The resulting plasmid pSW05 was verified by sequencing (GATC biotech) and transferred into *E. coli K12* MG1655 by electroporation. Allelic exchange was selected by plating on 5% sucrose according to Kaniga *et* *al*. (49). The *E. coli* 6′ mutant carrying the mutated 6′ TM was verified by sequencing (GATC biotech).

### Circular dichroism (CD) measurements

Oligonucleotides for CD measurements and melting assays were synthesized by Sigma Aldrich (Steinheim, Germany) at the 1 μmol scale with HPLC purification (sequences listed in Supplementary Table S9). CD spectra were measured on a JASCO-J815 spectropolarimeter equipped with an MPTC-490S/15 multicell temperature unit using a 1 cm optical path. Oligonucleotide samples were prepared as a 5 μM solution in 1× phosphate buffered saline (PBS) (137 mM NaCl, 2,7 mM KCl, 8 mM Na_2_HPO_4_ * 2 H_2_O, 2mM KH2PO_4_) buffer in a reaction volume of 600 μl. If not mentioned otherwise, 10 mM MgCl_2_ was always added. DNA folding was facilitated by heating to 98°C for 5 min, followed by slow cooling to 20°C over night. Scans were performed at 20°C over a wavelength range of 220–320 nm (five accumulations) with a scanning speed of 500 nm/min, 0.5 s response time, 0.5 nm data pitch and 1 nm bandwidth. The buffer spectrum was subtracted and all spectra zero-corrected at 320 nm.

### Thermal denaturation

For thermal denaturation oligonucleotides were prepared as for CD measurements. Folded samples were heated from 20°C to 100°C with a heating rate of 0.5°C min^−1^. The CD signal at 257 nm was recorded every 0.5°C. The temperature of the half-maximal decay of ellipticity T_m_ was obtained from the normalized ellipticity decrease.

### NMR spectroscopy

NMR spectra were acquired at 278 K on a Bruker Avance III 600 MHz spectrometer equipped with a TCI-H/C/N triple resonance cryoprobe. A total of 100 μM of the respective oligonucleotide was dissolved in1× PBS buffer 5% Vol. D_2_O as field lock. Triplexes were folded as described earlier, by heating up to 98°C and slowly cooling down to room temperature. ^1^D-proton spectra were acquired with 32 000 data points using 10k accumulated scans due to low sample concentration and processed with an exponential line broadening window function. Solvent suppression was achieved by excitation sculpting (50). Acquired data were processed and analyzed using Bruker Topspin and MestReNova software.

### *In vivo* DMS footprint

A total of 50 ml *E.coli* MG1655 cells were grown until exponential phase (OD_600_ = 0.1) in M9 minimal medium (supplemented with 1g casamino acids and 0.4% glucose or glycerol). Fifty microliters of a 10% dimethyl sulfate (DMS) solution (5 μl DMS, 25 μl EtOH, 20 μl H_2_O) were added to the bacterial culture and incubated for 5 min at 37°C before placing on ice. The whole 50 ml of the bacterial culture were pelleted at 4°C and washed with 1× PBS buffer. Next, cell pellets were dissolved in 480 μl SET buffer (150 mM NaCl, 15 mM ethylenediaminetetraacetic acid, 60 mM Tris–HCl, pH 8.3) (51) and cell lysis was performed by addition of 20 μl of 20% sodium dodecyl sulphate for 30 min at 37°C. Finally, 1.5 μl of Proteinase K (50 μg/ml, NEB) were added and Phenol/Chloroform purification (Roti-Phenol, Roth) was used for extraction of chromosomal DNA. The DNA was digested with RsaI (NEB) to generate a full-length product in the primer extension assay and purified via Phenol/Chloroform extraction (Roti-Phenol, Roth). Sequencing controls were generated by treating isolated chromosomal DNA with formic acid (FA) for purine sequencing or hydrazine (Hy) for pyrimidine sequencing, as described by Maxam and Gilbert (52). Cleavage at the modified sites was performed by addition of 10% Piperidine at 94°C for 30 min. The piperidine was removed in a vacuum concentrator. For primer extension the primer (5′-GAGGTAAATCGGAAGGGAAGAGG-3′) was radioactively 5′-end-labeled with γ-^32^P-ATP. Primer extension (binding site illustrated in Supplementary Figure S4) was performed with VENTexo- polymerase (NEB) and analyzed on a 10% denaturating PAGE gel.

### ITxF triplex finder

The triplex finding program (findtriplexes.pl, available as Supplementary Materials) consists of a perl script taking fasta format sequences as input and checking each nucleotide position in each sequence in turn. Before analyzing a sequence, N characters from the beginning of the sequence are appended to the end of the sequence to simulate a circularized genome (*N* = maximum_stem_size*3 + maximum_loop_size*2). Each position in that sequence is then analyzed in turn to see whether it matches our criteria for a triplex of type 1, 2, 3, 4 on either the forward or reverse strand. In the following we explain in detail the search for a triplex of type 1, but the search for the other triplex types is analogous, with a simple substitution of the nucleotide preferences in stems A, B or C, as depicted in Supplementary Figure S14. To identify potential triplexes of type 1, the program looks for stretches of between 6 and 15 (minimum and maximum stem size) A, G or T nucleotides. For each of these potential triplex stems, referred to as stemA, the program then checks all possible sequence regions between 6 and 15 nt within a distance of 1–6 nt (minimum to maximum loop size) to the end of stemA for a compatible pairing of residues to the nucleotide present in stemA (AA, GG, TA). If a suitable region is identified, referred to as stemB (the antiparallel stem), the program then searches a further region of 6–15 residues within a distance of 1–6 residues from the end of stemB for compatible residue pairing to those present in stemA and stemB (AAT, GGC, TAT). A single mismatch is allowed for stems of size 7 or longer, no mismatches are allowed for stems of size 6. If a region is identified matching all of the above criteria, an entry is written to a file listing the location in the sequence, the identified triplex type, the stem size, size of loop1, size of loop2, the strand orientation (+ or −), the sequence of stemA, the sequence of loop1, the sequence of stemB, the sequence of loop2 and the sequence of stemC.

### ITxF database design

The ITxF database (http://bioinformatics.uni-konstanz.de/utils/ITxF/) contains 5246 different genomes of bacterial and archaeal species, based on fully sequenced genomes and plasmids from the NCBI webserver (updated on 25 June 2014). In detail the database includes 3173 different bacterial chromosomes and 2073 plasmids. Plasmids and chromosomes were scrambled for usage as controls. Scrambled genomes were created by randomizing the order of the letters in the genome using a Perl Script (mononucleotide composition). The resulting scrambled genomes have an identical size and sequence composition as the original genomes. The algorithm performs searches for consecutive A-, T-, C- or G-rich regions in a specific sequential arrangement (Figure 1C) defining the three stems of the triplex structure. The regions in-between are defined as loops and can contain any nucleotide. The search identifies potential triplex structures with a stem size of 6–15 nt and a loop size of 1–6 nt. One mismatched basepair is allowed when the stem size is 7 nt or larger. As reported earlier (22), it is known that the stability of purine motif triplexes is driven by G•G-C triplets, while the stability of pyrimidine motif trilexes is driven by T•A-T triplets. Although the ITxF database searches for all possible triplex structures (see Figure 1C), the user can easily define search features and restrict the search to certain triplex types having higher potential for stable folding.

### Comparison of TM_ECO_-associated genetic loci for genetic variability studies

In order to shed light on the role of TM motifs we compared the genetic loci in proximity of TM motifs in 56 *E*. *coli* strains. We focused on the TMs occurring in *E. coli* with the consensus sequence 5′-CCCTCNCCCN_3–6_GGGNGAGGGN_3_GGGNGAGGG[GTC-]-3′, termed TM_ECO_, where N represents any nucleotide (A,T,G,C) and nucleotides in brackets stand for the different possibilities at the respective position. Whole genome sequences of 56 *E. coli* strains were downloaded from the National Center for Biotechnology Information (NCBI) (updated on 25 June 2014). Multiple whole genome alignment of all 56 *E. coli* genomes was performed via Mugsy 1.2.3 after which the locally collinear blocks (LCBs) were determined (53). The LCBs containing TM_ECO_ sequences were realigned by MAFFT v7 (54). After all alignments the TM_ECOs_ sharing similar surrounding sequences were categorized into homologous loci. To calculate the sequence variability v_j_ around a particular TM_ECO_ within the LCBs, we scanned all the aligned sequences using the following formula:

\begin{equation*} {\rm V}_{{\rm j}} = \frac{1}{{\rm l}}\sum\nolimits_{{\rm i} = {\rm j} - ({\rm l} - 1)/2}^{{\rm j} + ({\rm l} - 1)/2} {({\rm n}_{\rm i} - 1)} \end{equation*}

where n_j_ describes the nucleotide status at the aligned site j (either A,T,C,G or gap) and l stands for the length of the regarded window, which here is 11 nt. For the jth site of an aligned sequence, v_j_ represents the average variability of the surrounding l nucleotides. Each aligned site has a corresponding v_j._ To identify the dimension of the variable regions in the LCBs, we scanned the measured v_j_ values within one LCB. The start of a variable region was defined with v_j_ > 0.9 continuing for 10 consecutive nucleotides. A v_j_ < 0.5 for 10 continuous nucleotides defined the end of a variable region. That way we defined the variable sequence range for each LCB. We applied the same strategy for three control groups, containing random intergenic sites from the 56 *E. coli* genomes. Although analyzing 63 TM loci we determined only 48 v_j_ in total, because some TM_ECOs_ are occurring as tandem inverted repeats sharing one variable region.

Categorization (no change, region missing, intergenic deletions, not found) of sequence changes for the 23 TM_ECO_ sequences of *E. coli* MG1655 was carried out using the nucleotide BLAST webserver (http://blast.ncbi.nlm.nih.gov/) (55) and by comparison of nucleotide BLAST (algorithm: megablast) results for each of the 23 TM_ECO_ sequences found in *E. coli* MG1655 with the 55 different *E. coli* genomes. Eighteen regions were aligned since in MG1655 five sites contain tandem inverted TM_ECO_ motifs (hence 23 in total). We applied the following parameters: **Query sequence**: respective TM_ECO_ sequence (Table 1), **Database**: ‘NCBI genomes (chromosome)’, **Organism:** all 56 genomes listed in Supplementary Table S7.

**Table 1.** TM_ECO_ sequences in *E. coli* K-12 MG1655

No	TM_ECO_ sequence (5′ to 3′)	Length (nt)	Type	Genome localization	Genome position (')	Strand orientation
1	CCCTCTCCCTGTGGGAGAGGGCCGGGGTGAGGGC	34	B	164 547–164 580	3.5	sense+
2	CCCTCTCCCTTGAGGGAGAGGGTTAGGGTGAGGGT	35	A	164 631–164 597	3.5	antisense−
3	CCCTCGCCCCTTTGGGGAGAGGGCCGGGGTGAGGGG	36	B mm	282 101–282 136	6	sense +
4	CCCTCTCCCTGTGGGAGAGGGCCGGGGTGAGGGC	34	B	289 246–289 279	6.2	sense +
5	CCCTCGCCCCCTTGGGGAGAGGGTTAGGGTGAGGGG	36	A mm	388 664–388 699	8	sense+
6	CCCTCGCCCCCTCGGGGAGAGGGTTAGGGTGAGGGG	36	A mm	497 843–497 878	11	sense+
7	CCCTCGCCCCTTTGGGGAGAGGGTTAGGGTGAGGGG	36	A mm	624 579–624 614	13	sense+
8	CCCTCTCCCTTCCAGGGTGAGGGCTGGGGTGAGGGT	36	B	624 676–624 641	13	antisense−
9	CCCTCGCCCCTCTGGGGAGAGGGTTAGGGTGAGGGG	36	A mm	1 351 239–1 351 204	29	antisense−
10	CCCTCGCCCTTTCAGGGAGAGGGCCGGGGTGAGGGT	36	B mm	3 045 989–3 046 024	66	sense+
11	CCCTCGCCCCTTTGGGGAGAGGGTTAGGGTGAGGGG	36	A mm	3 046 087–3 046 052	66	antisense−
12	CCCTCTCCCTTCCAGGGAGAGGGTCGGGGTGAGGGT	36	B	3 239 599–3 239 634	70	sense+
13	CCCTCGCCCCGTTTGGGGAGAGGGTTAGGGTGAGGGG	37	A mm	3 239 698–3 239 662	70	antisense−
14	CCCTCGCCCCTTTGGGGTGAGGGTTAGGGTGAGGGG	36	A mm	3 390 529–3 390 494	73	antisense−
15	CCCTCGCCCCTTTGGGGAGAGGGTTAGGGTGAGGGG	36	A mm	3 504 892–3 504 857	75.5	antisense−
16	CCCTCGCCCCTCTGGGGAGAGGGTTAGGGTGAGGGG	36	A mm	3 608 684–3 608 719	78	sense+
17	CCCTCTCCCTGAGGGAGAGGGTTAGGGTGAGGGG	34	A	3 781 061–3 781 028	81.5	antisense−
18	CCCTCGCCCCTCCGGGGAGAGGGCCGGGGTGAGGGG	36	B mm	3 781 121–3 781 156	81.5	sense+
19	CCCTCGCCCCTCTGGGGAGAGGGTTAGGGTGAGGGG	36	A mm	3 908 495–3 908 530	84	sense+
20	CCCTCTCCCTGTGGGAGAGGGTCGGGGTGAGGGC	34	B	3 959 491–3 959 458	85	antisense−
21	CCCTCGCCCCTTTGGGGAGAGGGTTAGGGAGAGGGG	36	A mm	4 070 452–4 070 487	88	sense+
22	CCCTCGCCCCTCTGGGGAGAGGGTTAGGGTGAGGGG	36	A mm	4 314 285–4 314 320	93	sense+
23	CCCTCGCCCCTCCGGGGAGAGGGTTAGGGTGAGGGG	36	A mm	4 549 883–4 549 848	98	antisense−

## RESULTS

### The intrastrand triplex finder (ITxF) database

Previously triplex motifs have been identified bioinformatically in eukaryotes and prokaryotes. Most algorithms focus on inverted repeats (30,56) and H-DNA (57,58). As described above intramolecular triplexes do not necessarily form H-DNA within a dsDNA but instead occur within one single-stranded DNA by a double fold back. Databases with selective search functions for such intrastrand triplex motifs are rare. Maher *et* *al*. used the palingol program (59) to search for intrastrand triplexes by describing them as two hairpins that share a common homopurine strand. They identified representative intrastrand triplex motifs in the genomes of *E. coli K-12*, *Synechocystis sp*. and *Haemophilus influenza*, with the class II motif being the most abundant (22). Here we aimed at a more general and straightforward identification for intrastrand triplex motifs. For this purpose we developed a search algorithm for finding potential intrastrand triplexes among the different triplex classes in prokaryotes.

Our Intrastrand Triplex Finder (ITxF) database contains 3173 different genomic and 2073 different plasmid sequences of bacterial and archaeal species from the NCBI webserver. For each genome and plasmid we provide a scrambled version (shuffling was performed by maintaining mononucleotide composition) that was also searched for intrastrand triplex sequences. By comparing the TM content in original and scrambled genomes, the significance of the TM occurrence in bacterial genomes is easily assessed. The user-friendly design of our data base allows for downloading of raw data as well as pre-selected data as text files for further processing and analysis. The basic layout performs searches for consecutive A-, T-, C- or G-rich regions in a specific sequential arrangement (Figure 1C) defining the three stems of the triplex structure. The regions in-between are defined as loops and can contain any nucleotide (nt). The search identifies potential triplex structures with a stem size of 6–15 nt and a loop size of 1–6 nt. As the occurrence of imperfect triplexes has been described in different studies (60–62), our algorithm allows one mismatched basepair in the triplex, if the stem length has a minimal size of 7 nt (42). When looking for a specific type of triplex only, the user can easily choose the nucleotide composition and the G/C-content of the stem region and define certain stem and loop lengths of the query sequence. The program assigns the respective triplex class (class I–IV) to each sequence, distinguishing them by the strand orientation and the respective nucleotide composition (Figure 1C). The applied algorithm allows ‘mixed stems’ when the stem composition belong to the respective class, e.g. a triplex with the major stem sequence C-G•G may contain a T-A•A or T-A•T triplet within the stem, because both triplets refer to class II (illustrated in Figure 1C). In addition the database depicts the orientation and position of the identified triplex structures within genomes. The ITxF database is available online at http://bioinformatics.uni-konstanz.de/utils/ITxF/. To our knowledge, this is the first database that allows selective searches for intrastrand triplexes.

### Intrastrand triplex motifs are abundant in bacteria

Using the described algorithm and ITxF database, we identified large numbers of A/T- and G/C-rich triplexes in different prokaryotes. In total, 988 588 potential triplex sequences were found within the 5246 analyzed genomes and plasmids. Analyzing all genomes (including plasmids), we found that potential class II triplexes are most abundant: 41.6% of the triplexes found belonged to class I, 48.4% to class II, 6% to class III and 4% to class IV. Hoyne *et* *al*. identified 25, 18 and 21 potential intrastrand triplexes in *E. coli* K-12, *Synechocystis sp*. and *H. influenza*, respectively. In contrast, we found much higher numbers of potential triplex sequences: 169 in *E. coli* K-12 MG1655, 264 in *Synechocystis* PCC 6803 and 383 in *H*. *influenzae* 10 810. However, our search strategy is fundamentally different from the previous approach: Whereas Maher *et* *al*. defined their triplexes via a pattern recognition program searching for hairpin structures we used a new algorithm specifically aimed at intrastrand triplexes. Hoyne *et* *al*. searched for triplexes with a stem length of perfectly matched triplets of >7 nt and loops from 0 to 10 nt, we searched for triplex structures having a stem length from 6 to 15 nt, allowing one mismatch when larger than 7 nt and having loops with a size of 1–6 nt. In contrast to Hoyne *et* *al*. we found sequences potentially forming class I triplex structures present in all three species, but—similar to their findings—potential class II triplexes were the most abundant.

The search for intrastrand triplex motifs with other tools is difficult as there are no databases available searching specifically for potential intrastrand triplex structures. However, some databases provide search algorithms identifying intramolecular triplexes, primarily to search for TFO target sites. We tested two of them for comparison with our approach. The triplex database of Lexa *et* *al*. (57) searches for potential intermolecular triplex structures that require the consecutive occurrence of two triplex blocks. Potential intrastrand triplex structures require three consecutive triplex-forming sequence blocks and therefore should be identified with this program as well. As described earlier, the BoxC structures have been identified utilizing this tool (57). Applying this algorithm to *E*. *coli* MG1655 K-12, we were also able to identify the TM_ECO_s. However, the majority of identified sequences formed intermolecular but not intrastrand triplexes. Since it was designed to search for intermolecular triplexes and requires specialized settings for identification of potential intrastrand triplex structures the identification of intrastrand triplex motifs is laborious (57). In a second approach we applied an algorithm that searches for non-B-DNA structures in general (the Non-B database (63)). We searched for mirror repeats that might be potential triplex structures as well. We found 557 mirror repeats within the *E. coli* MG1655 genome, however only two of the TM_ECO_ motifs were identified applying this algorithm (Supplementary Table S13). We conclude from our comparison that the ITxF database is the only database providing a specialized search for potential intrastrand triplexes.

Interestingly, we discovered that the sequence composition of most triplex classes found with the ITxF database on the forward strand determines the occurrence of a different triplex class on the reverse strand, e.g. class II C-G•G on the one strand is often coupled with class III C•C-G triplexes in the complementary strand (Figure 1C). In fact, for G/C-rich sequences, the presence of class I or class II triplexes in one strand would always be associated with the presence of a class III or class IV triplex in the opposite strand. The same phenomenon is observed for A/T-rich sequences in case of A•A-T triads, but not for T•A-T triads. This might partially explain the abundance of class I and II triplexes relative to classes III and IV. Indeed, we observed an increased percentage of potential class I and II triplexes with increasing A/T content of the genomes (86.9% of potential class I and II triplexes for genomes with A/T content >50%; and 74.6% of potential class I and II triplexes for genomes with A/T content <50%). However, the observed increase is only marginal; also, for genomes with high G/C content, like e.g. Cellvibrio gilvus (73.89% G/C) a high percentage of class I and II triplexes (81.5%) can be observed (Supplementary Table S10). To further test our results, we investigated controls by scrambling the 5246 different prokaryotic genomic sequences and searching them for intrastrand triplex motifs. In the scrambled genomes we found 276 270 potential intrastrand triplexes (27.9% of the number of triplexes found in the wt genomes). The distribution of the different triplex classes in the scrambled control genomes is comparable to the distribution in the wt genomic sequences: 46.6% class I, 46.5% class II, 3.4% class III and 3.5% class IV. Matching our expectations, potential triplex structures with smaller stem sizes (6 and 7 nt) are more frequent in the scrambled genomes than those with larger stems, reflecting the higher probability of chance occurrence of shorter sequences. Triplex structures with stem sizes of 8 nt or larger only occur in 8% of the scrambled genomes (but in 17% of wt genomes). Stem sizes larger than 11 are not found at all in the scrambled genomes. Taken together, we find a remarkably higher number of potential triplex structures within genomes compared to scrambled sequences. Thus their occurrence seems to be non-random and hints at potential roles of these unusual nucleic acid structures in bacteria.

### G/C-rich versus A/T-rich intrastrand triplex motifs

The abundance of triplex motifs in bacteria suggests potential regulatory, organizational or adaptive functions, as proposed previously (25). Analyzing our data we found that 560 of all investigated genomes contained more than 500 potential triplexes within their genomes. Among them are bacteria that almost exclusively contain triplexes made up of the nucleotides A and T (e.g., *Flexibacter litoralis* DSM 6794, *Candidatus Carsonella ruddii*, *Enterococcus faecalis* D32) and others that almost exclusively contain the nucleotides G and C (e.g. *Rhodospirillum photometricum* DSM 122, *Microcystis aeruginosa* NIES-843, *Thermus thermophilus* HB8). About 77% of those triplexes had a stem size of 7 nt—this stem size we found for 84% of the triplexes in the scrambled genomes. As this is the smallest stem size that may contain one mismatched basepair it is likely that those sequences occur by chance rather than being significantly enriched. Therefore, we only included triplexes with stem sizes ranging from 8–15 nt for further comparison of A/T- and G/C-rich triplex structures. As mentioned above, our database allows the targeted search for either G/C- or A/T-rich triplexes by setting a user-defined G/C content of the stems. We compared the number of G/C_50_ (G/C content >50%) and A/T_50_ (G/C-content <50%) TMs present in the wt genomes with those found the in scrambled control genomes.

Intriguingly, we found that G/C-rich TMs are less frequent in the scrambled genomes. We found 21% of G/C_50_ and 79% of A/T_50_ potential triplex structures in genomic sequences, whereas 8.5% of G/C_50_ and 91.5% of A/T_50_ potential triplex motifs were found in the scrambled sequences. This proportion points toward a more integral role of G/C-rich triplex structures *in vivo*. Prominent examples with high proportions of G/C-rich triplex motifs are *M*. *aeruginosa* NIES 843, *Enterobacter* and *E. coli* species. Regarding all potential triplexes with stem lengths of 8 nt or larger, in *M*. *aeruginosa* 88% of the potential intrastrand triplexes found in the wt genome have a G/C content >50%, whereas 0% of the potential triplexes in the scrambled genome do (33% of wt G/C_50_ triplex motifs compared to 5% of scrambled G/C_50_ motifs when including stem sizes 6 and 7 nt). Other Cyanobacteria like *Anabaena variabilis* ATCC 29413 and *Nostoc sp*. PCC 7120 carry large numbers of potential G/C-rich triplexes as well (Anabaena: 87.5% G/C_50_ in wt, 0% G/C_50_ in scrambled; Nostdoc: 77.2% G/C_50_ in wt and 0% in scrambled). In *Enterobacter asburiae LF7a*, 98% of the potential triplexes in the wt genome and 33% of the triplexes in the scrambled genomes were G/C_50_ triplex motifs. However, the number of potential triplexes within different Enterobacter strains varies significantly: strains like *Enterobacter asburiae* LF7a, *Enterobacter cloacae subsp. cloacae* ATCC 13 047 and *Enterobacter cloacae subsp. dissolvens* SDM carry more than 300 TMs whereas in other strains such as *Enterobacter aerogenes* EA1509E, *Enterobacter sp*. R4–368 and *Enterobacter aerogenes* KCTC 2190 <30 TMs were identified. These drastic differences in closely related bacterial strains already hints at possible properties or roles of intrastrand triplexes.

When searching for G/C-rich triplex motifs, we noticed that a number of bacteria contain potential triplex motifs corresponding to the overall pattern:

5′-CCCTCNCCCN_3–6_GGGNGAGGGN_3_GGGNGAGGG[GTC-]-3′, where ‘N’ represents any nucleotide (G,T,A,C) and ‘–’ is no nucleotide. This purine motif triplex bears a C-rich stem (9 nt), a first loop (3–6 nt), a first G-rich stem (9 nt), a second loop (3 nt) and a second G-rich stem (9 nt); it may include one mismatched basepair in the stem. This potential triplex motif, subsequently named TM_ECO_ due to its abundance in *E. coli*, has already been described in an earlier study by Maher *et* *al*. However, no specific function could be assigned to the TM_ECO_ sequence. In contrast to earlier publications (22,41) using the ITxF search algorithm we found the TM_ECO_ sequence 3610 times in 262 different proteobacterial genomes (Supplementary Table S1): with a total number of 143 TM_ECO_ sequences *Enterobacter asburiae* LF7a contains the highest frequency of TMs of this type. Closely related genera such as *E. coli* and *Shigella* species carry comparable TM numbers, but the TM_ECO_ is completely absent in other closely related species such as *Salmonella*. However, a search in our database yields results for other triplex-forming sequences in these genomes. Also, the number of TM_ECO_ sequences between different strains within one genus may differ a lot, for example 29 motifs were found in *E. coli* 55 989 but only one in *E. coli* IHE3034. We were interested in potential roles or functions of TM sequences and decided to characterize TM_ECO_ in more detail.

### TM_ECO_ forms a stable intrastrand triplex *in vitro*

**Figure 2.**
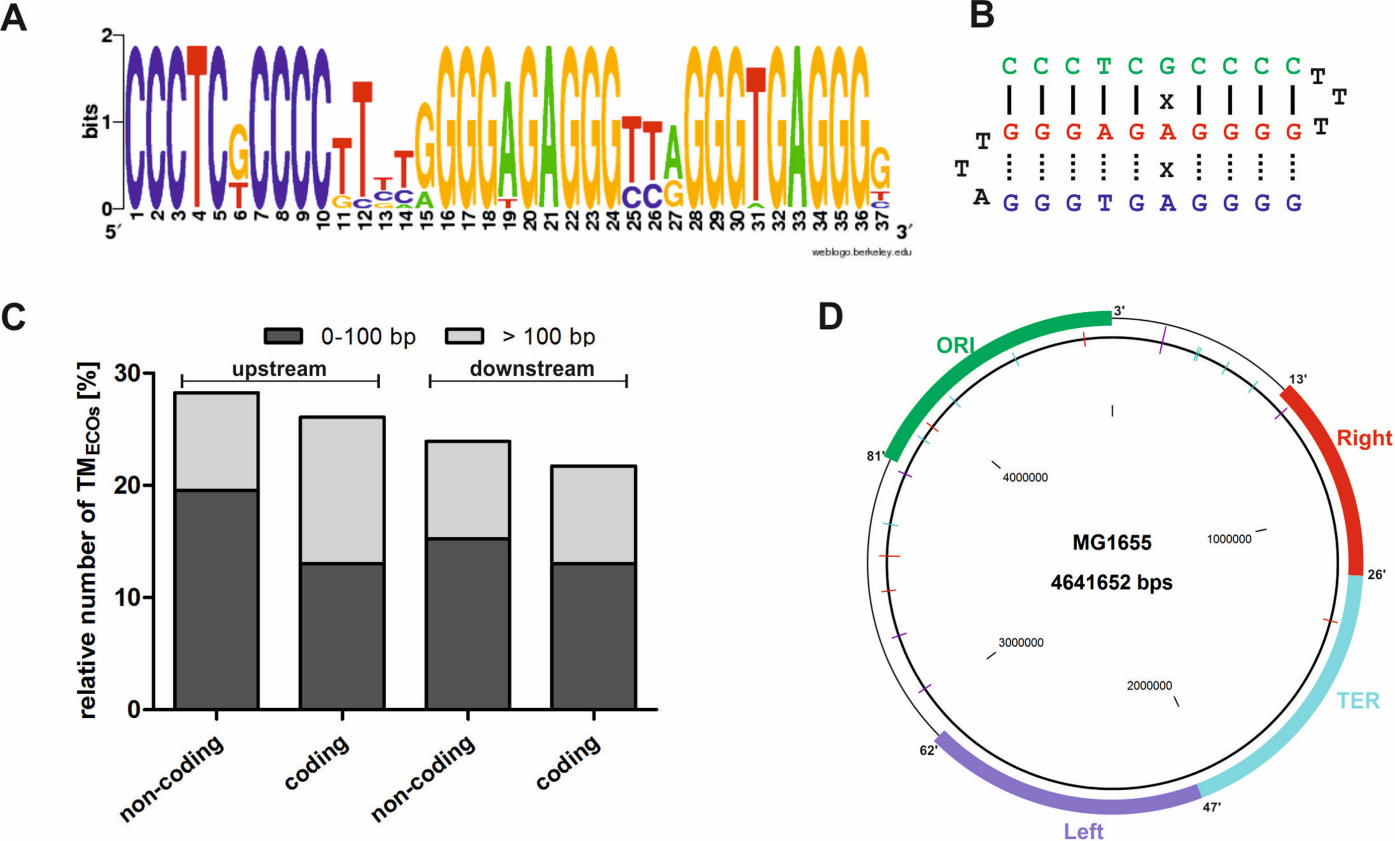
The TM_ECO_ sequence. (**A**) Consensus motif of TM_ECO_ sequences in *Escherichia coli K12 MG1655*. (**B**) TM_ECO_ sequence folding into a DNA class II triplex motif. Hoogsteen hydrogen bonds are indicated by dashed lines. (**C**) Distance of TM_ECOs_ relative to neighboring ORFs. Two categories are shown: 0–100 and >100 bp away from start of the ORF. For both strands (coding and non-coding) the region upstream and downstream of the ORF was analyzed. (**D**) Map of *E*. *coli MG1655* chromosome illustrating TM_ECO_ distribution. TM_ECO_ sequences are indicated as lines: TM_ECOs_ on plus strand (blue), TM_ECOs_ on minus strand (purple) and palindromic TM_ECOs_ sequences (red) are shown.

**Figure 3.**
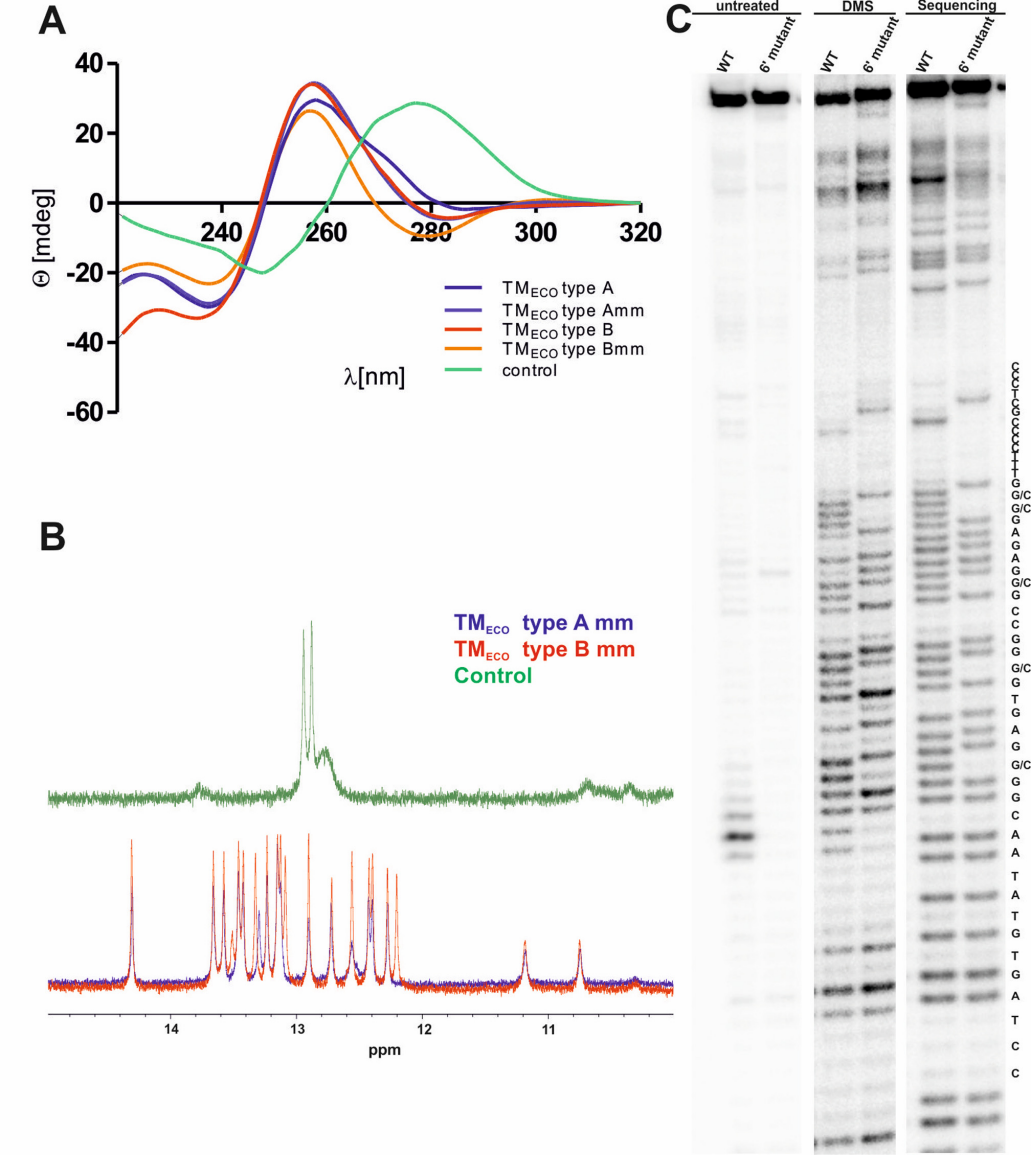
Structural characterization of TM sequences. (**A**) Circular Dichroism Spectroscopy of TM (TM type A in blue, TM type B in red) and control (containing for mutations, green) oligonucleotides. (**B**) Imino proton area of ^1^H-NMR spectra of TM and control oligonucleotides. (**C**) *In vivo* DMS probing of the TM sequence found at the genomic site 6′ in comparison to the 6′ control mutant: Primer extension reaction analyzed on a 10% denaturing PAGE. DMS treated probes (DMS) are shown in comparison to non-treated DNA (untreated) and the sequencing reaction for purine sequencing, according to Maxam and Gilbert (52).

In order to confirm that the identified TM_ECO_ forms a stable triplex structure, oligonucleotides were characterized via CD and NMR spectroscopy. Characteristic CD spectra of DNA triplex structures differ with the sequence of the oligonucleotide (64). However, most of the intramolecular triplex DNA show a minimum around 240 nm, a maximum around 257 nm and a second minimum at ∼280 nm (39,65). These peaks were also observed in the CD spectra of the TM_ECO_ oligonucleotides. The consensus sequence of the TM_ECO_ found in *E. coli K-12* substrain MG1655 is shown in Figure 2A. We measured CD spectra for two types of TM_ECO_: TM_ECO_ type A with the sequence 5′-TTA-3′ and TM_ECO_ type B with the sequence 5′-CCG-3′ in the second loop. We investigated both TM_ECO_ types with and without one mismatched basepair (mm), respectively (Figure 3A). Furthermore we analyzed a control sequence (5′-CCCTCGCCCCTTTGCCGAGAGCGTTAGCGTGAGCGG-3′), which contains four G to C mutations and should not be able to form a triplex—this sequence yielded spectra that resembles duplex (B-DNA) structures (Figure 3A and Supplementary Figure S2E) (64). We found the structures to be very stable as CD spectra showed the characteristic peaks up to a temperature of 75°C, although CD signatures decrease with increasing temperature (Supplementary Figure S2). We compared the influence of magnesium on triplex stability and found only minor changes in CD and thermal denaturation spectra (Supplementary Figure S9) as well as in TM_ECO_ CD spectra at different temperatures (Supplementary Figure S10). Next, we determined the stability of the TM_ECO_ oligonucleotides (5 μM) by thermal denaturation studies: melting temperatures of 82 ± 4°C, 78 ± 1°C, 74 ± 1°C and 70 ± 1°C were determined for TM_ECO_ type A, TM_ECO_ type B, TM_ECO_ type A mm and TM_ECO_ type B mm, respectively (Supplementary Figure S2F). Although CD spectra showed minima and maxima that were observed for triplex structures before, characteristic peaks for parallel G-quadruplex structures are very similar (minimum at 240 nm and maximum at 260 nm). The ability of G-rich triplex sequences to fold into quadruplex structures has been observed before (66). The G-rich part of the TM sequence could in principle form an intramolecular G-quadruplex composed of 3 stacked guanine tetrads (Supplementary Figure S1B) competing with triplex formation. In order to exclude quadruplex formation and prove triplex folding, we carried out NMR measurements. The ^1^H-NMR spectra of TM_ECO_ oligonucleotides displays 18 sharp signals in the imino proton range that clearly demonstrate the formation of well-defined triplex structures (Figure 3B). If an intramolecular G-quadruplex structure would form we would expect much less imino proton signals (3–4 signals). However, when complementary strands were added, CD signals characteristic for duplex structures were observed and the NMR spectrum of TM_ECO_ type B mm showed less and broader signals in the imino proton range (Supplementary Figure S3).

After the demonstration that the studied TM_ECO_ sequences fold into stable triplexes *in vitro*, we were interested in characterizing whether triplex formation is also detectable *in vivo*. In the bacterial cell, triplex conformations do not necessarily have to occur permanently but could form transiently. For example, supercoiling and helicase activity in replication and transcription favor the formation of non-duplex DNA structures during strand separation (67). In order to investigate whether chromosomal sites containing TM_ECO_ sequences are double-stranded or fold into alternate conformations in the living bacterium, we performed *in vivo* footprinting. Bacteria were incubated with DMS that selectively methylates guanines at N7. The central G-rich stretch of the TM (shown in red in Figure 3B) should be protected in a triplex fold whereas N7 should be methylated in the duplex form at the respective locus. After DMS quenching, DNA isolation and cleavage at methylated positions the DMS accessible sites were identified via a primer extension reaction. An *in* *vitro* control experiment probing a synthetic DNA strand demonstrated protection from methylation via triplex (Supplementary Figure S7). Figure 3C shows the DMS footprint of the TM_ECO_ site at the 6′ position of the *E. coli* MG1655 chromosome (TM_ECO_ number 3, a type B triplex containing 1 mm, see Table 1) in comparison to a genomic mutant containing G to C exchanges (6′ mutant). However, we observed cleavage at the respective guanine sites in the wt strain with comparable band intensities to the control. Interestingly, a strong band can be observed directly in front of the triplex sequence, which was not present in the mutated chromosome (Figure 3C: untreated WT versus 6′ mutant). This band likely results from a polymerase stop during the subsequent primer extension reaction, indicating triplex formation under assay conditions.

### Characterization of the TM_ECO_ in *E. coli* MG1655

The TM_ECO_ sequence was found 23 times in the *E. coli K-12* MG1655 chromosome (sequences listed in Table 1). The TM_ECO_ is always located intergenically with no bias to strand orientation (Figure 2C and D). Using NCBI BLAST(55), we did not find the motif associated with high mobility genetic elements such as transposons, phages or plasmids. The consensus sequence of the 23 motifs shows an extraordinary degree of identity (Figure 2A). When searching the 37 nt long TM_ECO_ consensus sequence in the *E. coli* MG1655 strain we received an *E*-value of 6 × 10^−14^, indicating the number of hits expected to occur by chance when searching the database with the effective sequence space of 256 million nucleotides. Hence, the investigated motif is significantly overrepresented in the MG1655 genome. In general, the loop sequences show less sequence conservation compared to the stem regions of the triplex structure. The putative triplex formed by the TM_ECO_ sequence is a G/C-rich class II purine motif structure (Figure 2B). The complementary, C-rich strand might be able to form a pyrimidine motif class III triplex, which is more stable under acidic conditions (Supplementary Figure S1A) that usually do not occur within the bacterial cell. As described above we identified two different TM_ECO_ types: TM_ECO_ type A with the sequence T•T-A and TM_ECO_ type B with the sequence C•C-G in the second loop. In total, 15 of the 23 TM_ECOs_ found belong to type A while the other 8 can be assigned to type B motifs (Table 1). Among the different motifs type A mm (including one mismatched base triplet) is the most frequent (13 TM_ECOs_). Regarding the strand orientation of the TM_ECO_ in the genome we found 13 motifs located on the plus strand and 10 motifs located on the minus strand of the genome. In five cases two TM_ECOs_ are positioned closely in an inverted repeat configuration with a TM_ECO_ on the forward and the other on the reverse strand. In all five cases always a type A motif is was found combined with a type B motif (TM_ECO_ 1 + 2, 7 + 8, 10 + 11, 12 + 13, 17 + 18 in Table 1), comparable findings have been described by Hoyne *et* *al*. (22). In *E. coli K-12* it seems that the TM_ECO_ motifs are clustering in genomic regions that are represented by the first half of the two replicores (i.e., near the origin of replication rather than near the terminus region, see Figure 2D). However, in other *E. coli* strains and in other bacteria we did not observe such a non-random distribution.

We next investigated whether the genes flanking the intergenic TM_ECO_ sequences belong to certain functional classes (Supplementary Table S2). The formation of a triplex structure might affect the regulation of the local gene expression and could be related to a general mechanism for a certain gene class. However, by categorizing gene functions using the KEGG database (68,69) we found the motifs located in proximity to all kinds of gene classes and functions lacking a functional correlation. Most TM_ECOs_ are located in proximity to genes of general categories such as metabolic pathways, amino acids and secondary metabolites biosynthesis, and ABC transporters. Since the formation of non-canonical nucleic acid structures can interfere with transcription or translation (70–73) we investigated the distance of the TM_ECOs_ relative to the open-reading frame (ORF) of the neighboring genes in *E. coli* MG1655 (Figure 2C). The motifs were found more often upstream of an ORF than downstream of an ORF. However, the separation between the TM_ECO_ and the ORF ranges from very close (10 nt) to larger distances (310 nt) and shows no trend to a specific distance. As highly regulatory regions (5′-UTRs, ribosome binding site, promoter regions) are generally located closer than 200 bp from the ORF, our findings do not hint at a general regulatory role of the TM_ECO_ on gene expression. Regarding the location of the TM_ECOs_ within operons we found no bias of operon arrangement relative to the triplex sequences (Supplementary Table S2). These findings are in line with the lack of evidence for a potential role of triplex motifs in regulating gene expression described by Maher *et* *al*. (41).

### TM motifs as a source of genetic instability

**Figure 4.**
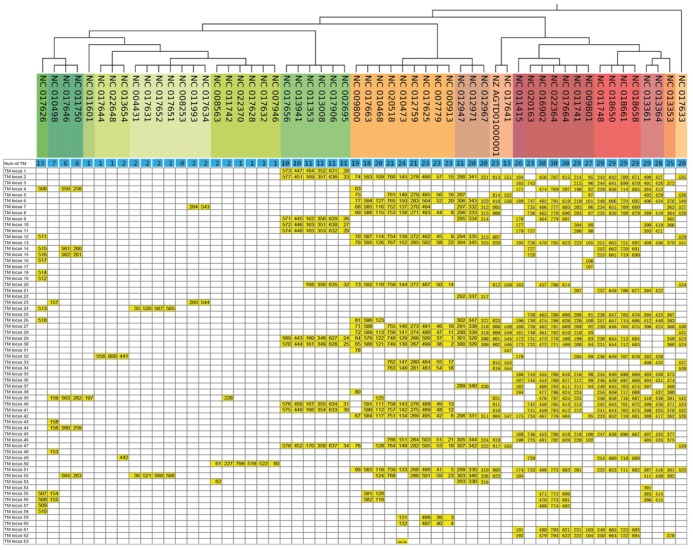
TM_ECO_ motifs in different strains of *Escherichia coli*: Distribution of TM sequences in the 63 TM loci. Each row represents a different *E. coli* substrain (genome number listed). The phylogeny of the strains according to the NCBI web server is shown. The number of TM sequences found in the respective strain is listed in the first line (blue). Each of the other lines represents one TM locus. TM motifs occurring in a respective locus within a respective strain are indicated in yellow (TM numbers according to Supplementary Table S8).

Repetitive sequences and non-canonical DNA structures are often associated with highly variable genetic regions (4,72,74). As mentioned above, we noted that the numbers of TM sequences per genome varied greatly between closely related species and even individual strains within bacterial species. We therefore investigated whether TMs could be associated with an increased frequency of genomic changes such as mutations, rearrangements or recombination events. For this purpose we compared 56 different *E. coli* strains (Supplementary Table S7) from 40 distinct genome groups (http://www.ncbi.nlm.nih.gov/genome/genomegroups/) for TM_ECO_ elements and compared the genetic variability around those regions: In the 56 strains we found in total 823 TM_ECO_ sites (listed in Supplementary Table S8). For a better recognition of homologous regions between different strains we split the genomes into aligned locally collinear blocks in which we mapped the different TM_ECO_ sequences (see ‘Materials and Methods’ section for detailed description and Supplementary Table S5 for block assignment). We identified 62 conserved TM_ECO_ loci, in which the TM_ECOs_ have homologous surrounding sequences but may be located at different positions in the genomes of different *E. coli* strains. The TM_ECO_ locus 63 contains two TM_ECOs_ found in *E. coli* strain DH 10B (NC_010473) which could not be assigned to any block (TM_ECO_ numbers 135 and 136). Figure 4 shows the distribution of the 823 TM_ECO_ sequences in these 63 TM_ECO_ loci in the different strains. We found no correlation between the genome size and the number of TM_ECO_ loci (Supplementary Figure S8).

**Figure 5.**
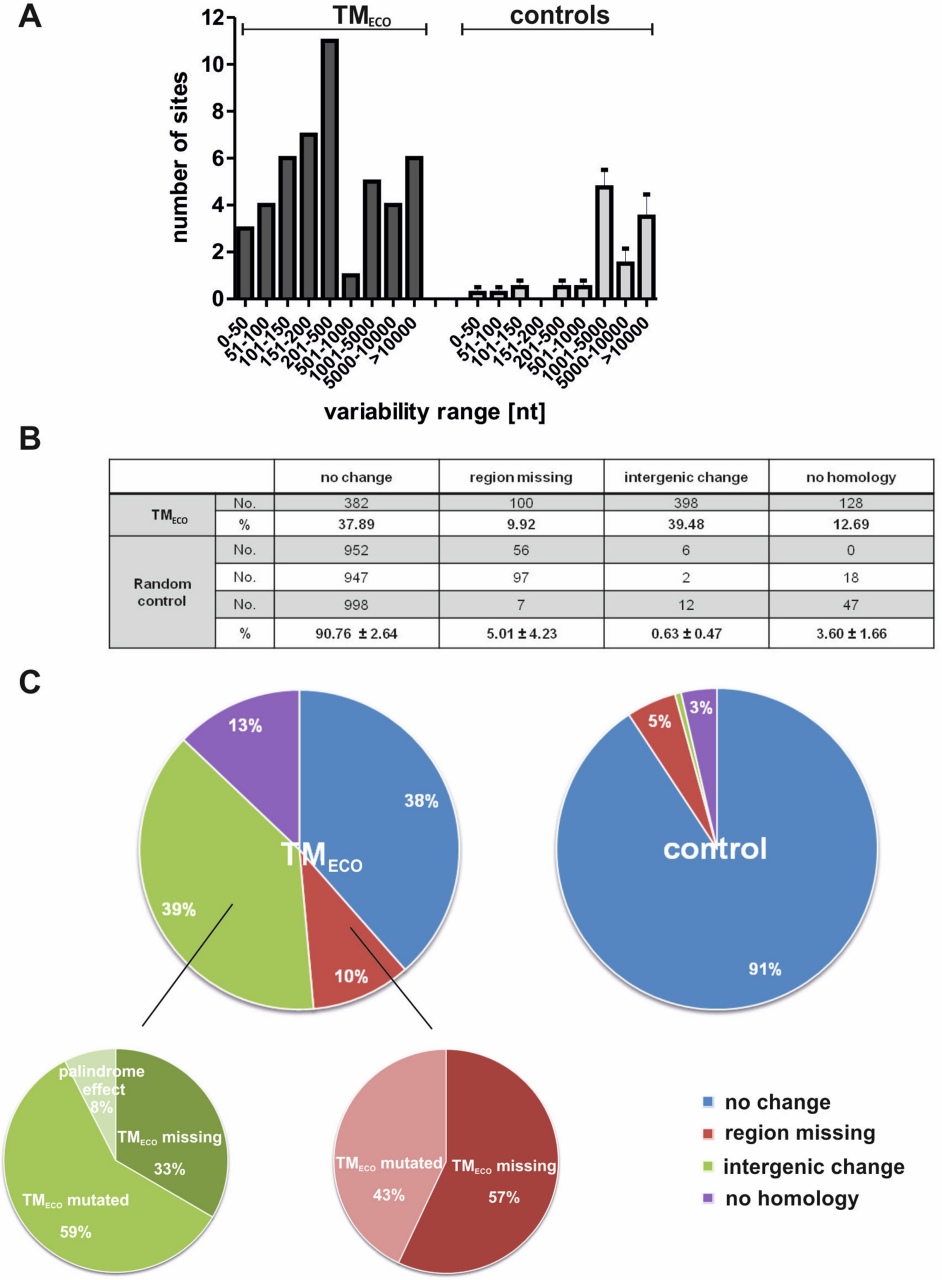
Genomic variance at TM_ECO_ sites of *Escherichia coli K12*. (**A**) Variability range in nucleotides around the TM_ECO_ motifs occurring in the 63 TM_ECO_ loci of the 56 *E. coli* strains compared to random controls. Details on variability calculation are described in the experimental part. (**B** and **C**) Results of megaBLAST sequence comparison of the regions (500 bp up- and downstream) of the 23 TM_ECOs_ found in *E. coli* K12 MG1655 to the other 55 *E. coli* genomes. Four different categories were defined: (i) no change—sequences are identical in the different strains; (ii) region missing—a region larger than 300 bp is missing in the compared strain; (iii) intergenic change—less homology in the intergenic region occur; (iv) no homology—the respective motif is not found in the compared strain. Absolute numbers of strains and percentages for each category are listed for the investigated TM_ECO_ sites and three random control groups in Panel B. Panel C illustrates the distribution of the different categories when comparing TM_ECO_ and random control regions. The categories ‘region missing’ and ‘intergenic change’ were further subdivided whether the TM_ECO_ motif itself was missing or mutated. For the palindromic regions an effect generating a potential hairpin structure was observed. This feature is assigned as palindrome effect.

Next, we investigated the size of variable regions surrounding each TM_ECO_ (indicated in Supplementary Table S6) by analyzing mutations and deletions in the alignment files. We calculated the range of sequence variability around the particular TM_ECO_ within a locally collinear block by splitting each block into windows of 11 nt and defining a sequence variability value v_j_ for each window (see ‘Materials and Methods’ section). We observed that an average length of 2966 nt is variable around each TM_ECO_ locus. For better evaluation of our data we analyzed the genetic instability in four different control groups composed of randomly chosen intergenic sites. In most investigated control regions we observed no genetic instability, although on average 9 of 48 control regions for each group show a certain sequence variability as well (Supplementary Table S6). However, especially for regions between 1 and 500 nt around the TM_ECO_ loci the sequence variability was observed to be much higher compared to the control groups (Figure 5A). To ensure that the instability signature is not only related to the underlying Watson–Crick hairpin, but requires all three sequence domains of the potential triplex structure, we searched for hairpins in the 56 *E. coli* strains. We searched for hairpins with very similar patterns to the TMs (9–15 nt stem, 1–6 nt loop and at most 1 mismatch in the stem). Applying this search we found that hairpins are widely spread in the *E. coli* genomes: 187 898 hairpins were found within the 56 *E. coli* genomes and 2163 hairpins were found in the *E. coli* K-12 MG1655 in the non-coding regions (Supplementary Table S11). From this huge number of hairpins we can infer that they should not cause genetic instability as this would contradict the conservation of genomes. However, to get a deeper insight we randomly selected 23 hairpins occurring in the *E*. *coli* MG1655 genome and compared the length of the variable region around the 23 TM_ECOs_ and the 23 random hairpins. All of the 23 TM_ECOs_ were observed in 18 variable regions (some of them share one variable region), while 12 of the 23 random hairpins were found in variable regions and 11 of them were located in conserved loci (Supplementary Table S12). The average length of the variable regions around the randomly selected hairpins is 1814 nt, which is less than we observed for the TM_ECO_ loci (2966 nt). Taken together, our findings strongly suggest TM_ECOs_ are a source of genetic instability. However, the mechanism responsible for this increase in variability remains unclear.

In order to gain further insights into the observed TM-associated genetic variance we next focused on the 23 TM_ECO_ sequences found in the *E. coli* MG1655 genome. We picked sequences ranging from 500 nt upstream to 500 nt downstream of a TM_ECO_ sequence and used NCBI megaBLAST to analyze the sequence similarity of the region around the motif compared to all other *E. coli* substrains. For analysis we defined five different categories: (i) no observed sequence change—the TM_ECO_ and flanking sequence is similar in the compared genomes; (ii) region missing—a large region (defined as more than 300 nt, containing either non-coding or coding sequences) shows no homology; (iii) intergenic changes—the intergenic (non-coding) region is less homologous (completely/partly deleted or sequence insertions) but the flanking coding regions are similar in the aligned strains; (iv) no homology—the whole region cannot be found at all in the aligned strain. We compared 1008 regions in total (56 strains multiplied with 18 TM_ECO_ sites). Figure 5B and C shows the distribution of those categories when comparing the18 genomic sites in the 56 genomes (listed in Supplementary Table S3). We observed that in 38% of the analyzed regions no considerable change in the genomic sequence can be found. In 39% of the cases we observed intergenic changes. In almost 10% of the analyzed regions a large sequence part (>300 bp) was not homologous (‘region missing’) and about 13% of the TM_ECOs_ occurring in *E. coli* MG1655 cannot be found homologous to other *E. coli* substrains at all. In our analysis, we recognized that in many cases of the categories ‘region missing’ and ‘intergenic changes’, triplex motifs with lower stability are present, which results from degenerated TM_ECO_ sequences (examples are shown in Supplementary Figure S5). For this reason, we further subcategorized those two classes, each in ‘TM_ECO_ missing’ and ‘TM_ECO_ mutated’. For the category ‘region missing’ the results were well balanced: in 57% of the genomes the TM_ECO_ was missing and in 43% of the genomes the TM_ECO_ was mutated. In the category ‘intergenic changes’ we found 33% of the genomes with TM_ECO_ missing and 59% with TM_ECO_ mutated regions. Interestingly, by regarding the palindromic sites more closely we observed that either the TM_ECO_ sequences were completely missing or a part of both sequences was missing so that stable stem loop structures could form (Supplementary Figure S6). This effect was considered as palindromic effect and made up 8% of the ‘intergenic changes’.

For a better evaluation of our results we again compared three sets of control sequences based on the same criteria (Figure 5 and Supplementary Table S4). These control regions had an average length of 1070 bp and were randomly chosen from *E. coli* MG1655; they always contained an intergenic (non-coding) region, carrying no TM_ECO_, framed by two coding regions. Intriguingly, for more than 90% of the control regions we observed no sequence changes. A larger region of the analyzed genomic parts is missing in 5% of the investigated strains, and in 3% of the strains the analyzed region was not found at all. In <1% of the investigated sites a short intergenic sequence part was missing. These findings demonstrate that there is much more variability in terms of DNA sequence change around the TM_ECO_ sequences compared to control regions: both the number of sites missing a larger region and those bearing intergenic deletions are considerably increased compared to the controls.

## DISCUSSION

The relationship between DNA sequence repeats and genomic instability has been described in different studies: instability caused by TR sequences has been attributed to different hereditary diseases such as Huntington disease (75); chromosomal plasticity in *Pseudomonas fluorescens* species has been associated to MITE sequences (76); REP sequences have been linked to genetic instability in *E. coli* toxin–antitoxin systems (77) and other repetitive sequences have been described in correlation to genomic plasticity in bacteria (78,79). Most of the repeat sequences have the potential to fold into non-canonical secondary structures on the DNA and/or RNA level, as it has been described for pneumococcal bacteria (80). Such non-canonical nucleic acid structures are prone to interfere with replication, recombination, transcription and translation, as described earlier (22). The exact mechanisms of those influences, however, have not been elucidated to date. The exact properties and putative functions of many repetitive elements occurring in eukaryotes and prokaryotes are still unclear. Here we focused on intrastrand triplex DNA repeats in prokaryotes.

We generated the ITxF database that enables an easy search for potential intrastrand triplex structures of different structural classes within 5246 prokaryotic genomes and plasmids. Although different computational tools allowing the search for potential triplex sites in genomes have been reported, the ITxF database is—to our knowledge—the first one defining intrastrand triplex structures that are not necessarily H-DNA or TFO binding sites. The data demonstrate a high abundancy of triplex motifs in bacterial chromosomes, suggesting that they are highly enriched and do not occur by chance. We noted particularly high abundances of a certain class of G/C-rich TMs that are present in more than 260 prokaryotes. Using CD and NMR spectroscopy, we demonstrated that the DNA sequence indeed forms a very stable triplex *in vitro*. However, in genomic contexts such sequence motifs are accompanied by the reverse complement strand and duplex formation always competes with triplex formation and other non-canonical structures. CD and NMR studies of the double-stranded TM_ECO_ sequence did not show characteristic triplex signals and showed the formation of dsDNA. Furthermore, in *in vivo* DMS footprinting experiments methylation was not blocked at specific guanines involved in triplex formation. Although the presented data does not support stable triplex formation *in* *vivo*, it cannot be excluded that triplexes form at least transiently during processes such as replication, recombination or transcription.

Further characterization of TM_ECO_ sequences did not discover consensus sequences or functions of flanking genes. In addition, TM_ECO_ motifs (then called PIT motifs) did not show any promoter or terminator activity in an earlier investigation (41). However, when we compared the genetic context in closely related strains, we observed a significantly higher variability compared to intergenic DNA stretches analyzed as controls. H-DNA sequences have been mapped to recombination hotspots in mouse myeloma cells (81). When investigating the genomic changes between the 23 TM_ECO_ sequences found in *E. coli* MG1655 and the other strains in more detail, we found that only in 10% of the strains a larger region was missing around a TM_ECO_. If induction of recombination were the function of TM_ECOs_ we would have expected that a higher percentage of larger regions would be changing. However, H-DNA structures have been proposed to be involved in recombination processes (82–84). In principle recombination could occur between two distant TM_ECO_ sites. Furthermore, TM_ECO_-related recombination events at non-homologous sites, such as illegitimate recombination, could be induced by DNA breaks or strand slippage during replication near the TM_ECO_ structure. But, most of the TM_ECO_ sequences were assigned to a particular TM_ECO_ locus for all of the 56 genomes. Apart from the genetic changes that might result from recombination events, we found increased genetic variation in flanking regions adjacent to the TM_ECO_. Most of the changes associated with TM_ECO_ were small intergenic changes (39%), where the TM_ECO_ sequence was either completely deleted or mutated.

Furthermore, for palindromic TM occurrences (inverted repeat configuration of TMECO on opposite DNA strands) we observed that in most cases small parts were deleted in such a way that a hairpin-like secondary structure might still be able to form. This peculiarity might hint at possible mechanisms of how triplex formation interferes with genetic processes such as replication, thereby resulting in the observed higher genetic instability. Replication of the circular bacterial chromosome proceeds bidirectionally, starting at the ORI where the replisome is recruited. A replication fork acts on each chromosomal arm, and its progression can be blocked when meeting obstacles such as bound proteins, bulky adducts, interruptions in the DNA template or non-B-DNA structures. An inactivation of the replication fork may lead to double-strand breaks, polymerase stalling or replication slippage. Replication slippage could serve as an explanation for the deletion of the TM_ECO_ motif (85,86). Stalled replication can be reinitiated by primase, which creates a new primer that binds after the obstacle and leaves a gap in the DNA sequence (87). This mechanism could also explain the deletion of the TM_ECO_ including parts of the flanking regions. Furthermore, a stalled replication fork may cause double strand breaks that may be processed in a mutagenic fashion. In mammalian cells H-DNA has been reported to induce double strand breaks (88). Usually, the proceeding of replication after stalling or the processing of gaps during replication is initiated by DNA repair enzymes. Non-B-DNA structures have been shown to recruit DNA repair proteins (89,90). A frequent consequence of DNA repair is the introduction of mutations or small deletions, which could explain the high amount of intergenic deletions and mutated TM_ECOs_ we found in our analysis. In another scenario, DNA repair in proximity to the TM_ECO_ sequence may generate superhelical stress and thereby facilitate the transition from the DNA double strand to the triplex structure. Non-B-DNA structures forming during DNA repair could alter the repair process and have been suggested to contribute to error-generating repair and genomic instability (91). Although such mechanisms are known to be related to repeat sequences and non-canonical DNA structures, to our knowledge comparative analyses of genomic variability related to an intrastrand triplex motif had not been conducted so far. However, genome instability was frequently reported to be associated with H-DNA (72,90,92).

In this study we show that TM sequences are associated with increased genomic instability in *E. coli*, contributing to the diversity of different *E. coli* strains. Interestingly, genomic diversity has also been observed in many cyanobacteria where it has been related to high numbers of repeat sequences (93). Especially *M*. *aeruginosa* strains show high genomic plasticity (94). Interestingly, we found very high numbers of G/C-rich TM sequences in cyanobacteria, especially in *M*. *aeruginosa* (443 G/C_50_ motifs). Many pathogenic bacteria are known for highly variable and adaptive genomes (95,96). In our ITxF database we noted high abundancies of G/C_50_ triplex motifs in pathogenic bacteria as well: *Shigella flexneri* (∼40 G/C_50_ TMs), *Pseudomonas aeruginosa* (∼60 G/C_50_ TMs), *Neisseria meningitides* (∼50 G/C_50_ TMs) and *Neisseria gonorrhea* (∼100 G/C_50_ TMs). Hence it might be possible that G/C-rich intrastrand triplex motifs act as general inducers of prokaryotic genome plasticity. As such they might serve as a novel mechanism to increase DNA sequence variation and genomic structural variation as new raw material for selection to act on. If indeed one evolutionary function of TM sequences were to increase variation it might be possible to test the adaptive value of TMs by conducting experimental evolutionary test. This could be done, for example, by having strains of *E. coli* (with different numbers of TMs) compete under previous and new environmental conditions with the predictions that strains with more TMs might be outcompeting those with fewer TMs.

Although it might be possible that TM sequences are involved in genomic evolution and organismal adaptation processes, a benefit of high numbers of TM sequences for the bacterium remains to be demonstrated. However, the origin of the motif remains unclear: has it been inserted or deleted during evolution? In our analysis we found less TM_ECO_ sequences in 27 strains that separated in the third generation. Those strains do not seem to differ in adaptive or environmental functions compared to the 29 strains bearing more TM_ECO_ sequences. Data from a long-term experiment of genomic changes by Lenski *et* *al*. in *E. coli B REL600* (97) showed no correlation to TM_ECO_ sequences either. The study was carried out utilizing a laboratory strain that was not exposed to drastic environmental changes or selection pressure. Possibly adaptive mutations need harsher conditional changes or longer time periods to evolve. A possible hypothesis as to how TM sequences could have been distributed in different bacterial genomes is horizontal gene transfer (HGT). HGT describes a process that brings non-parental (vertical) genetic information into a cell ‘horizontally’ from the genomes of other species. TM sequences could be ‘leftovers’ from such processes. However, when analyzing the gene contexts around TM_ECO_ sites we never found activities that provide genetic mobility such as remnants of transposases or integrases. In conclusion, we characterized highly conserved and very wide-spread intrastrand triplex motifs. We were able to show that TM_ECO_ sequences—forming stable triplex DNA structures *in vitro—*seems to induce higher levels of genetic variation in *E. coli* subspecies. Although different forms of these particular triplex motifs have been noted and studied before (3,22,41,46), our findings provide a significant functional property of TM sequences. However, intriguing questions regarding the mechanism and evolutionary origins of these interesting motifs remain unanswered.

## Supplementary Material

SUPPLEMENTARY DATA
