# Supplementary material for: Intrastrand triplex DNA repeats in bacteria: a source of genomic instability
Source: Nucleic Acids Res. 2015 Oct 7;43(21):10126–42. doi: 10.1093/nar/gkv1017 (PMC4666352; doi:10.1093/nar/gkv1017)
Supplement: SUPPLEMENTARY DATA [file supp_43_21_10126__index.html]

Intrastrand triplex DNA repeats in bacteria: a source of genomic instability — Intrastrand triplex DNA repeats in bacteria: a source of genomic instability — SUPPLEMENTARY DATA 

# Intrastrand triplex DNA repeats in bacteria: a source of genomic instability

## SUPPLEMENTARY DATA

- SUPPLEMENTARY DATA
