# Supplementary material for: Intrastrand triplex DNA repeats in bacteria: a source of genomic instability
Source: Nucleic Acids Res. 2015 Oct 7;43(21):10126–42. doi: 10.1093/nar/gkv1017 (PMC4666352; doi:10.1093/nar/gkv1017)
Supplement: SUPPLEMENTARY DATA [file supp_gkv1017_nar-01747-z-2015-File003.docx]

**Supplementary Information content:**

**Figure S1.** Schematics of alternative structures that could form apart of the class II triplex of the TM_ECO_ sequence.

**Figure S2.** Circular dichrosim spectroscopy at different temperatures.

**Figure S3.** Structural characterization of the double stranded TM_ECO_.

**Figure S4.** Schematical illustration of primer extension reaction for DMS footprint.

**Figure S5**. Representative modifications of the TM_ECO_ sites in the category “intergenic deletions”.

**Figure S6.** Genetic instability at palindromic TM_ECO_ sites.

**Figure S7.** In vitro Footprint of TM_ECO_.

**Figure S8.** Correlation between genome size and number of TM_ECOs_.

**Figure S9.** Influence of MgCl_2_ on the stability of the intrastrand triplex formed by TM_ECO_.

**Figure S10.** CD Spectra at different temperatures.

**Table S1.** TM_ECO_ sequences in proteobacteria

**Table S2.** Flanking genes of TM_ECOs_ in *E. coli*

**Table S3.** Evaluation of genomic instability studies of the 23 TM_ECOs_ found in *E. coli* MG1655.

**Table S4.** Control regions analyzed for genomic instability in *E. coli* MG1655.

**Table S5.** Description of the different TM loci defined for all TM_ECOs_ found in the 56 *E. coli* genomes

**Table S6.** Sequence variability [nt] calculated for the TM_ECOs_ and the different random control groups.

**Table S7.** Description of the 56 fully sequenced *E. coli* genomes used for our analysis.

**Table S8.** TM_ECO_ sequences identified in the 56 *E. coli* genomes listed in Table S7.

**Table S9.** Oligonucleotide sequences used for CD, thermal denaturation and NMR studies.

**Table S10.** Percentage of potential class I and II triplexes in correlation to G/C content of the genome.

**Table S11.** Number of hairpins found in E. coli.

**Table S12**. Sequence variability around random hairpins.

**Table S13.** Triplex search with Non-B-DNA database in *E. coli* MG1655 K-12.

**Table S14.** ITxF triplex finder search algorithm.


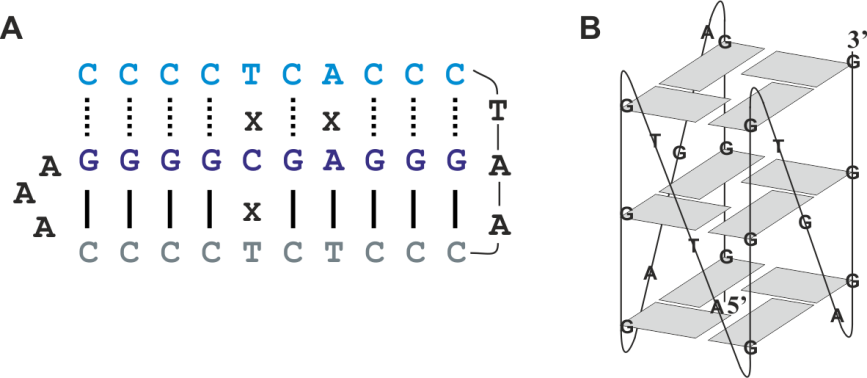


**Figure S1.** Schematics of alternative structures that could form apart of the class II triplex of the TM_ECO_ sequence. **A** Conformation of a pyrimidine motif class III triplex that could potentially be formed by the C-rich antisense strand. **B** Quadruplex motif that could be formed by the G-rich stretches of the TM sequence.


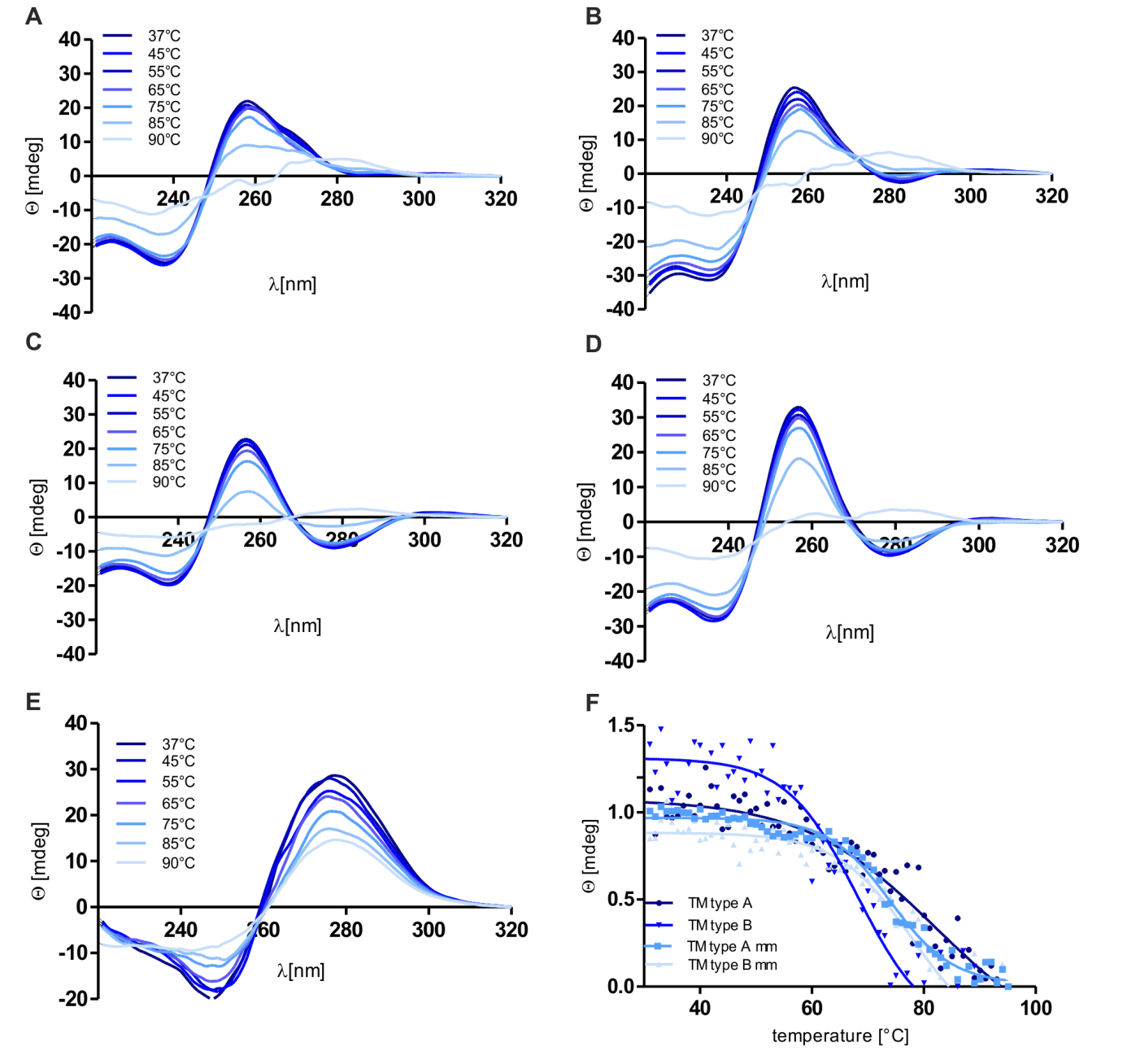


**Figure S2.** Circular dichrosim spectroscopy at different temperatures of **A** TM_ECO_ type A complete match **B** TM_ECO_ type B complete match **C** TM_ECO_ type A mm **D** TM_ECO_ type B mm **E** control **F** thermal denaturation studies of TM_ECO_ type A, B, Amm and B mm at 257nm.


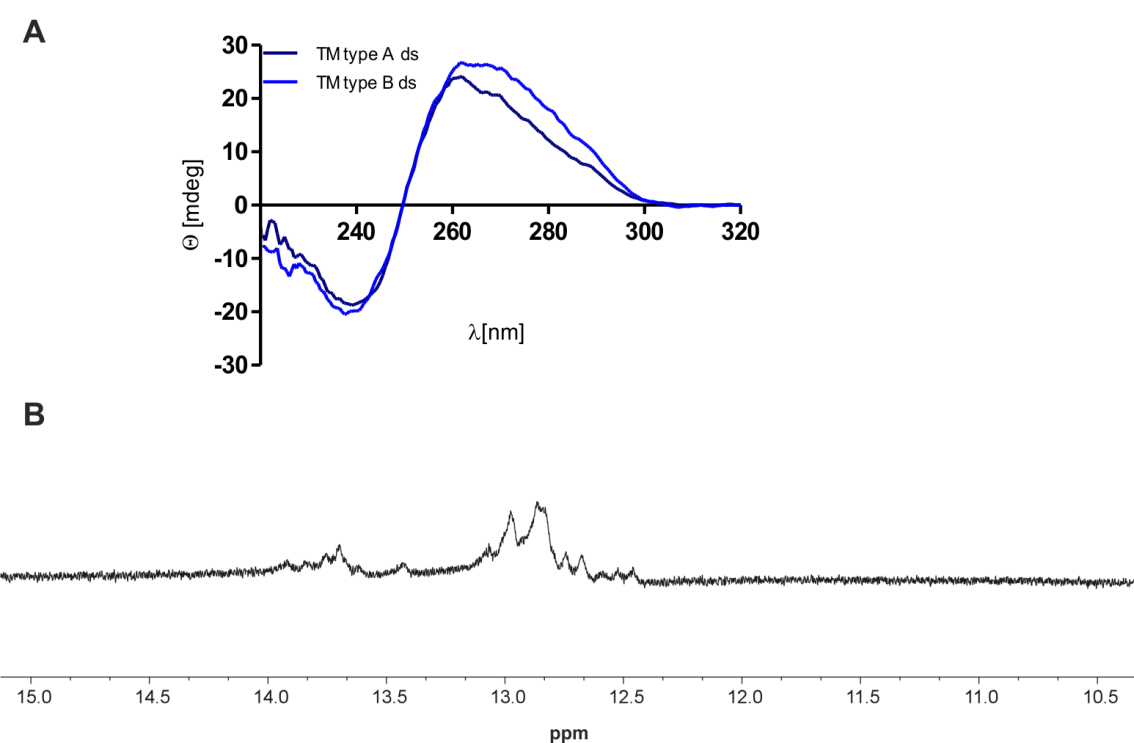


**Figure S3.** Structural characterization of the double stranded TM_ECO_. **A** Circular dichroism of the double stranded TM_ECO_ type A and TM_ECO_ type B. **B** Imino proton range of 1H NMR of the double stranded TM_ECO_ type B mm.


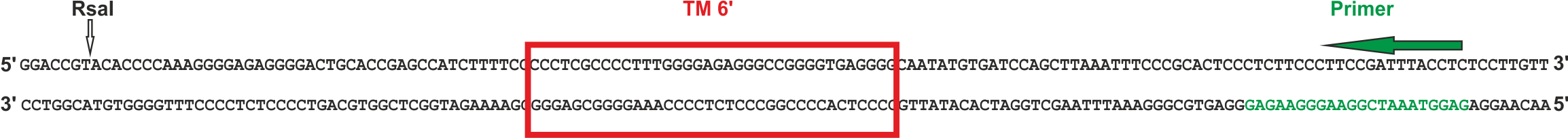


**Figure S4.** Schematical illustration of primer extension reaction for DMS footprint. Red Box indicates TM_ECO_ motif, primer binding site is marked in green and RsaI restriction site is shown in black.


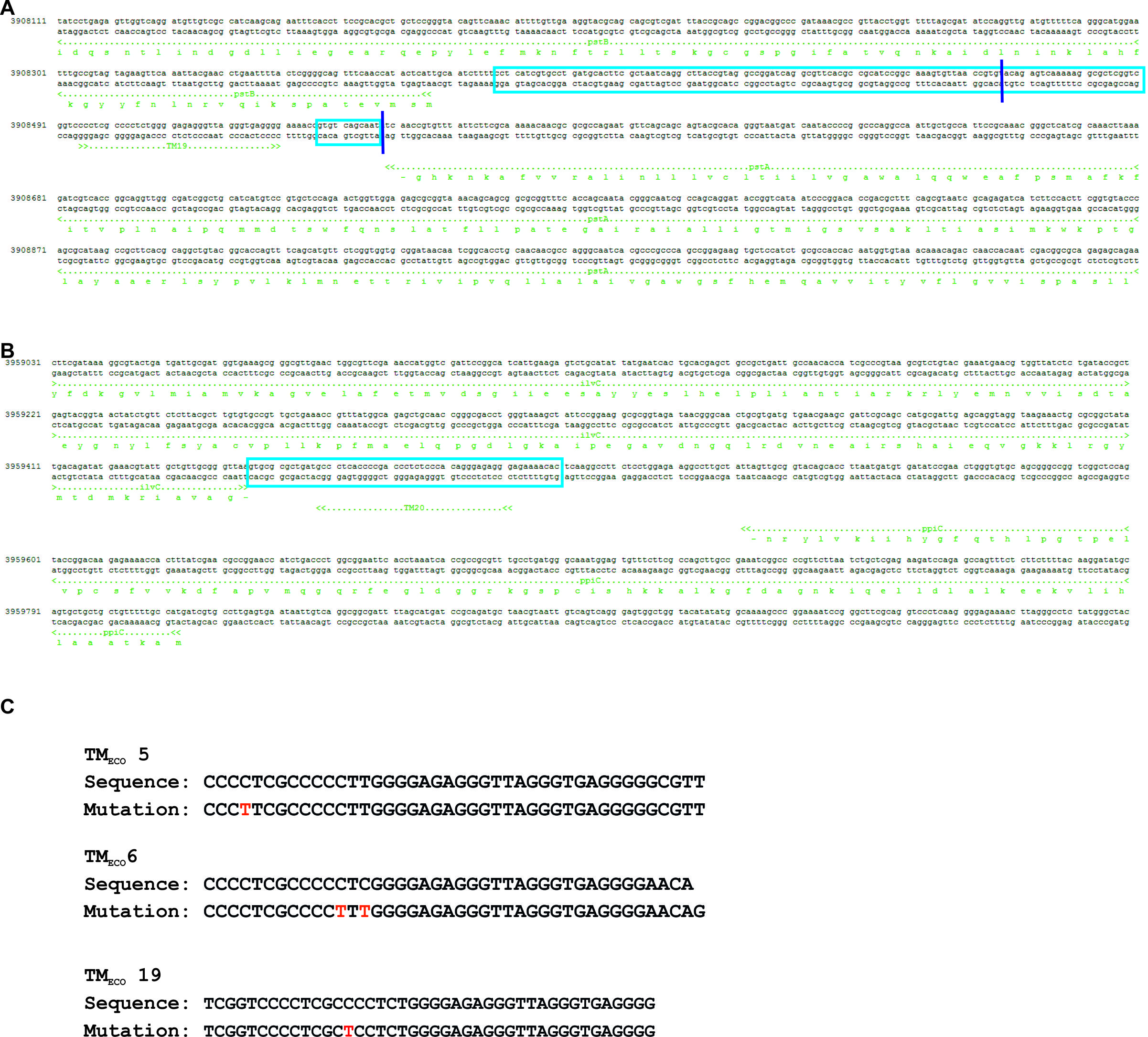


**Figure S5**. Representative modifications of the TM_ECO_ sites in the category “intergenic deletions”. Blue boxes mark regions that are deleted in some strains at those specific sites. Dark blue lines mark region with observed can occur. **A** TM_ECO_ 19 – subcategory TM_ECO_ mutated **B** TM_ECO_ 20 – subcategory: TM deleted. **C** examples of less stable triples motifs occurring via mutation in the subcategory **TM_ECO_** mutated.


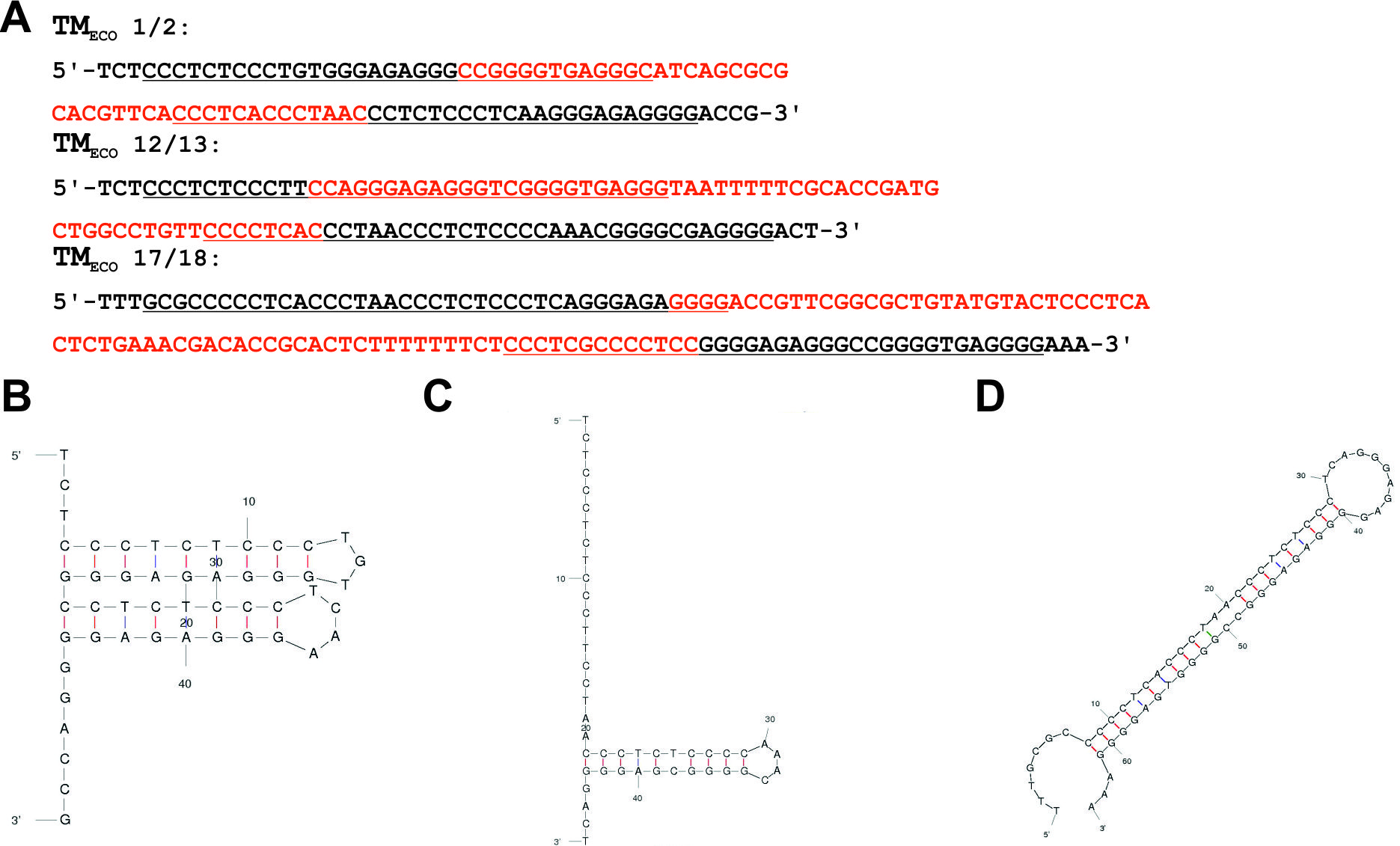


**Figure S6.** Genetic instability at palindromic TM_ECO_ sites. **A** TM sequences at the indicated palidromic regions with intergenic spacers and 3 nt flanking region. TM_ECO_ sites are underlined, deleted sequences are indicated in red. Potential stem-loop structures that could be formed when the red sequence is deleted at the particular site: **B** TM_ECO_ 1/2, **C** TM_ECO_ 12/13 and **D** TM_ECO_ 17/18. Structure prediction according to the mfold webserver


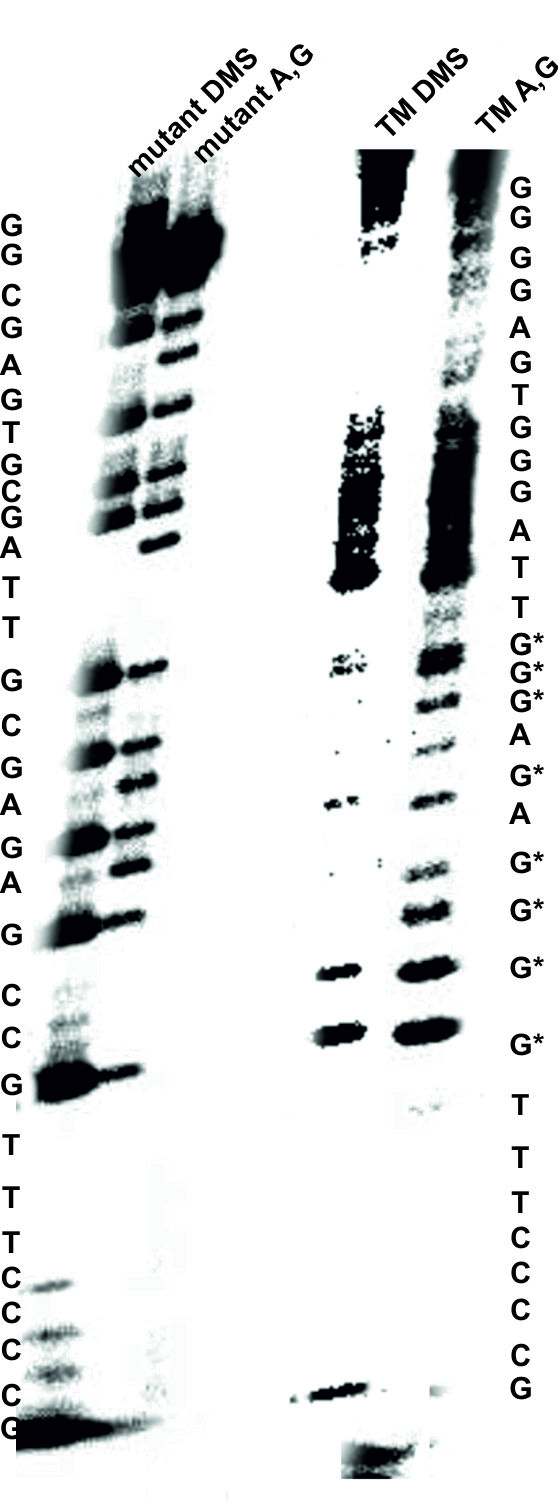


Figure S7. *In vitro* DMS footprint of TM_ECO_ motif and the respective control mutant. Oligonucleotides were 5’ end labeled with γ-^32^P-ATP and DMS probing was performed as described earlier (Seemann, I.T., JACS, 2011([1](#_ENREF_1))). For both oligonucleotides, the mutant, which should not be able to form a triplex structure (sequence: 5’-CCCTCGCCCCTTTGCCGAGAGCGTTAGCGTGAGCGG-3’) and the TM motif oligonucleotide (sequence: 5’-CCCTCGCCCCTTTGGGGAGAGGGTTAGGGTGAGGGG-3’) DMS probing (left; DMS) and purine sequencing (right; A,G) is shown. The respective sequence is listed besides (mutant: right; TM:left). Guanines that should participate in triplex formation via Hoogsteen boning are indicated with * and most of them a clearly protected from cleavage in the TM DMS sample.


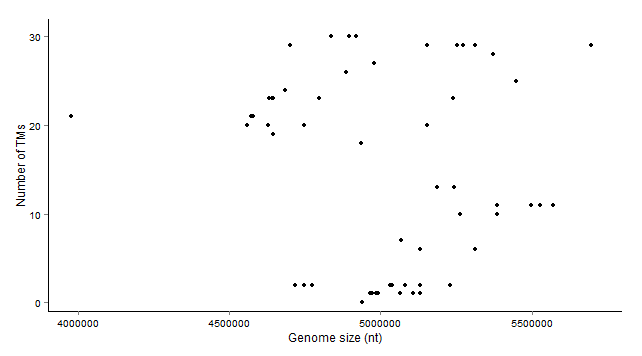


**Figure S8.** Correlation between genome size and number of TMECOs. The vertical axis shows the number of TMs in the 56 *E. coli* genomes and the horizontal axis shows the size of the respective *E. coli* genome.


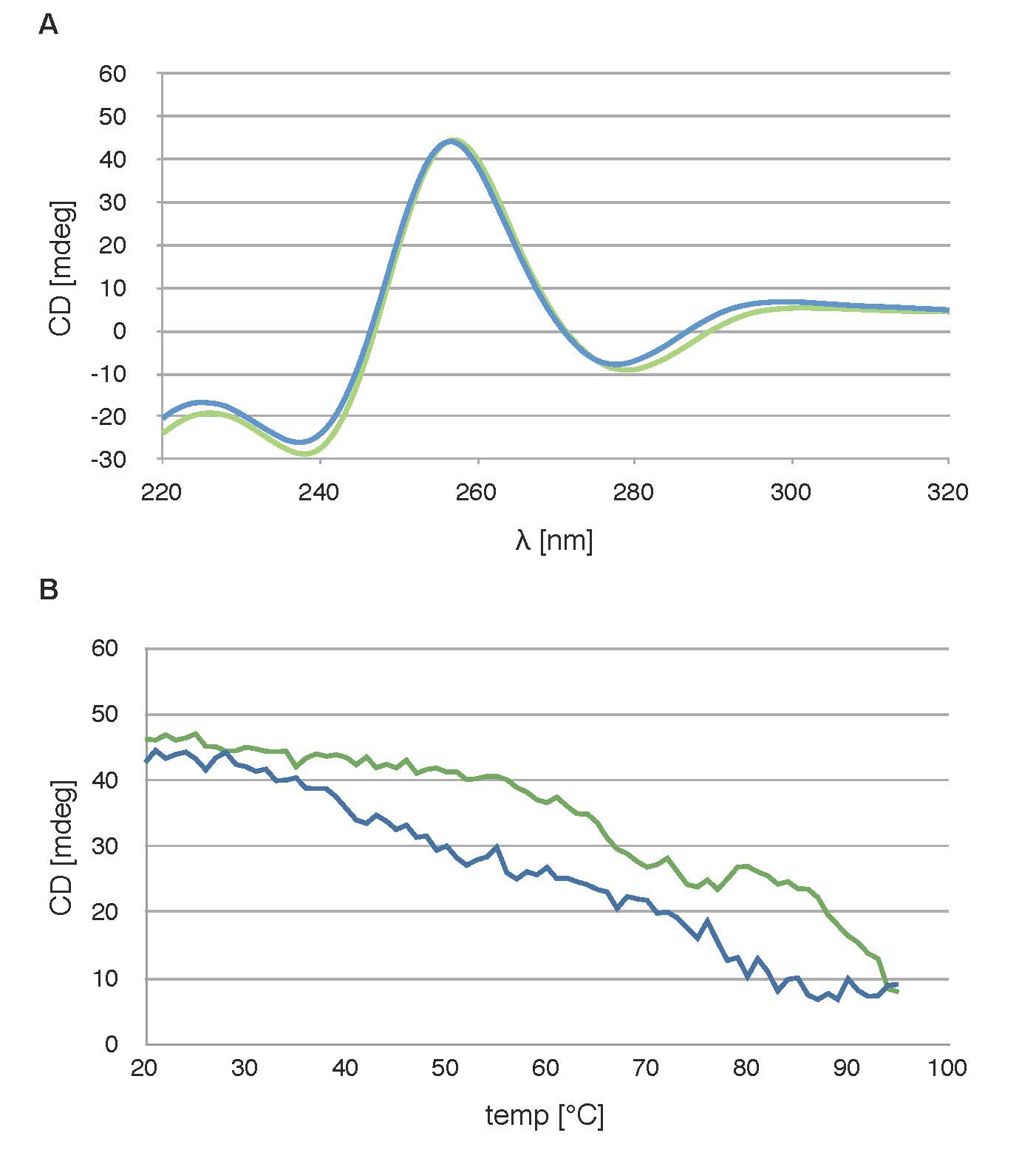


**Figure S9.** Circular dichroism and thermal denaturation of TM_ECO_ with (green) and without (blue) Magnesium.A Circular dichroism spectroscopy. B Thermal denaturation. Spectra are recorded at 20°C as described in the methods part, blue without MgCl_2_ and green with addition of 10 mM MgCl_2_.

**
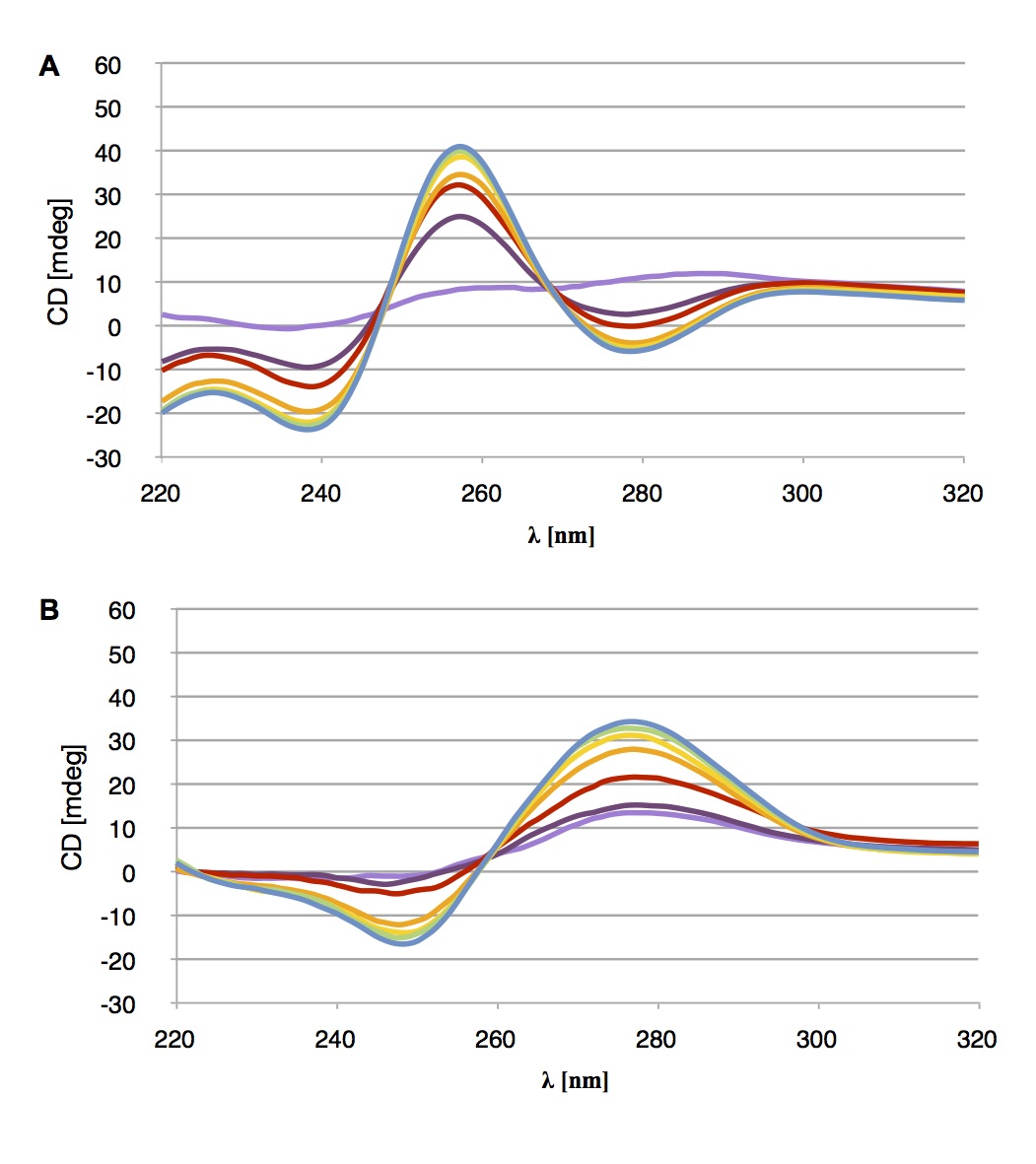
**

**Figure S10.** CD Spectra at different temperatures. Comparison of CD spectra of TMECO (**A**)and the mutated control (**B**) at increasing temperaturesThe CD spectra were recorded at 37 °C (blue), 45 °C (green), 44 °C (yellow), 65 °C (orange), 75 °C (red), 85 °C (purple), and 95 °C (light purple).

**Table S1.** TM_ECO_ sequences in proteobacteria

| **Organism** | **Number of TM_ECOs_** |
| --- | --- |
| Aeromonas_hydrophila_ATCC_7966_uid58617/NC_008570.fna | 5 |
| Aeromonas_hydrophila_ML09_119_uid205540/NC_021290.fna | 3 |
| Aeromonas_salmonicida_A449_uid58631/NC_009348.fna | 1 |
| Aeromonas_veronii_B565_uid66323/NC_015424.fna | 4 |
| Agromonas_oligotrophica_S58_uid192186/NC_020453.fna | 1 |
| Alcanivorax_dieselolei_B5_uid176364/NC_018691.fna | 8 |
| Alicycliphilus_denitrificans_BC_uid49953/NC_014910.fna | 1 |
| Alicycliphilus_denitrificans_K601_uid66307/NC_015422.fna | 1 |
| Arthrospira_platensis_NIES_39_uid197171/NC_016640.fna | 20 |
| Asticcacaulis_excentricus_CB_48_uid55641/NC_014816.fna | 1 |
| Asticcacaulis_excentricus_CB_48_uid55641/NC_014817.fna | 1 |
| Azorhizobium_caulinodans_ORS_571_uid58905/NC_009937.fna | 3 |
| Azospirillum_B510_uid46085/NC_013854.fna | 2 |
| Azospirillum_B510_uid46085/NC_013855.fna | 2 |
| Azospirillum_B510_uid46085/NC_013856.fna | 3 |
| Azospirillum_B510_uid46085/NC_013858.fna | 4 |
| Azospirillum_B510_uid46085/NC_013859.fna | 1 |
| Azospirillum_brasilense_Sp245_uid162161/NC_016594.fna | 28 |
| Azospirillum_brasilense_Sp245_uid162161/NC_016595.fna | 11 |
| Azospirillum_brasilense_Sp245_uid162161/NC_016596.fna | 9 |
| Azospirillum_brasilense_Sp245_uid162161/NC_016617.fna | 33 |
| Azospirillum_brasilense_Sp245_uid162161/NC_016618.fna | 20 |
| Azospirillum_lipoferum_4B_uid82343/NC_016585.fna | 8 |
| Azospirillum_lipoferum_4B_uid82343/NC_016586.fna | 7 |
| Azospirillum_lipoferum_4B_uid82343/NC_016587.fna | 6 |
| Azospirillum_lipoferum_4B_uid82343/NC_016622.fna | 13 |
| Azospirillum_lipoferum_4B_uid82343/NC_016623.fna | 2 |
| Azospirillum_lipoferum_4B_uid82343/NC_016624.fna | 12 |
| Azotobacter_vinelandii_CA_uid198829/NC_021149.fna | 26 |
| Azotobacter_vinelandii_CA6_uid198830/NC_021150.fna | 26 |
| Azotobacter_vinelandii_DJ_uid57597/NC_012560.fna | 26 |
| Bradyrhizobium_BTAi1_uid58505/NC_009485.fna | 2 |
| Bradyrhizobium_japonicum_USDA_110_uid57599/NC_004463.fna | 32 |
| Bradyrhizobium_japonicum_USDA_6_uid158851/NC_017249.fna | 17 |
| Bradyrhizobium_S23321_uid158167/NC_017082.fna | 25 |
| Candidatus_Methylomirabilis_oxyfera_uid161981/NC_013260.fna | 2 |
| Caulobacter_crescentus_CB15_uid57891/NC_002696.fna | 17 |
| Caulobacter_crescentus_NA1000_uid59307/NC_011916.fna | 17 |
| Chlorobium_tepidum_TLS_uid57897/NC_002932.fna | 4 |
| Chthonomonas_calidirosea_T49_uid208678/NC_021487.fna | 1 |
| Comamonadaceae_bacterium_CR_uid223378/NC_022576.fna | 2 |
| Cupriavidus_necator_N_1_uid68689/NC_015723.fna | 9 |
| Cupriavidus_necator_N_1_uid68689/NC_015726.fna | 2 |
| Cupriavidus_taiwanensis_LMG_19424_uid61615/NC_010528.fna | 5 |
| Cupriavidus_taiwanensis_LMG_19424_uid61615/NC_010530.fna | 7 |
| Desulfobacca_acetoxidans_DSM_11109_uid65785/NC_015388.fna | 2 |
| Desulfococcus_oleovorans_Hxd3_uid58777/NC_009943.fna | 1 |
| Enterobacter_638_uid58727/NC_009436.fna | 70 |
| Enterobacter_aerogenes_EA1509E_uid187411/NC_020181.fna | 3 |
| Enterobacter_aerogenes_KCTC_2190_uid68103/NC_015663.fna | 4 |
| Enterobacter_asburiae_LF7a_uid72793/NC_015968.fna | 143 |
| Enterobacter_cloacae_ATCC_13047_uid48363/NC_014121.fna | 129 |
| Enterobacter_cloacae_dissolvens_SDM_uid168997/NC_018079.fna | 132 |
| Enterobacter_cloacae_EcWSU1_uid80739/NC_016514.fna | 12 |
| Enterobacter_cloacae_ENHKU01_uid172463/NC_018405.fna | 87 |
| Enterobacter_cloacae_NCTC_9394_uid197202/NC_021046.fna | 83 |
| Enterobacter_cloacae_SCF1_uid59969/NC_014618.fna | 16 |
| Enterobacter_R4_368_uid208672/NC_021500.fna | 1 |
| Enterobacteriaceae_bacterium_FGI_57_uid185181/NC_020063.fna | 140 |
| **Escherichia_coli__BL21_Gold_DE3_pLysS_AG__uid59245/NC_012947.fna** | **21** |
| **Escherichia_coli__clone_D_i14__uid162049/NC_017652.fna** | **2** |
| **Escherichia_coli__clone_D_i2__uid162047/NC_017651.fna** | **2** |
| **Escherichia_coli_042_uid161985/NC_017626.fna** | **12** |
| **Escherichia_coli_55989_uid59383/NC_011748.fna** | **29** |
| **Escherichia_coli_ABU_83972_uid161975/NC_017631.fna** | **2** |
| **Escherichia_coli_APEC_O1_uid58623/NC_008563.fna** | **2** |
| **Escherichia_coli_APEC_O78_uid187277/NC_020163.fna** | **23** |
| **Escherichia_coli_ATCC_8739_uid58783/NC_010468.fna** | **20** |
| **Escherichia_coli_B_REL606_uid58803/NC_012967.fna** | **20** |
| **Escherichia_coli_BL21_DE3__uid161947/NC_012971.fna** | **20** |
| **Escherichia_coli_BL21_DE3__uid161949/NC_012892.fna** | **20** |
| **Escherichia_coli_BW2952_uid59391/NC_012759.fna** | **21** |
| **Escherichia_coli_CFT073_uid57915/NC_004431.fna** | **2** |
| **Escherichia_coli_DH1_uid161951/NC_017625.fna** | **23** |
| **Escherichia_coli_DH1_uid162051/NC_017638.fna** | **23** |
| **Escherichia_coli_E24377A_uid58395/NC_009801.fna** | **27** |
| **Escherichia_coli_ETEC_H10407_uid161993/NC_017633.fna** | **20** |
| **Escherichia_coli_HS_uid58393/NC_009800.fna** | **19** |
| **Escherichia_coli_IAI1_uid59377/NC_011741.fna** | **29** |
| **Escherichia_coli_IAI39_uid59381/NC_011750.fna** | **6** |
| **Escherichia_coli_IHE3034_uid162007/NC_017628.fna** | **1** |
| **Escherichia_coli_JJ1886_uid226103/NC_022648.fna** | **1** |
| **Escherichia_coli_K_12_substr__DH10B_uid58979/NC_010473.fna** | **24** |
| **Escherichia_coli_K_12_substr__MDS42_uid193705/NC_020518.fna** | **21** |
| **Escherichia_coli_K_12_substr__MG1655_uid57779/NC_000913.fna** | **23** |
| **Escherichia_coli_K_12_substr__W3110_uid161931/NC_007779.fna** | **23** |
| **Escherichia_coli_KO11FL_uid162099/NC_017660.fna** | **30** |
| **Escherichia_coli_KO11FL_uid52593/NC_016902.fna** | **30** |
| **Escherichia_coli_LF82_uid161965/NC_011993.fna** | **2** |
| **Escherichia_coli_LY180_uid219461/NC_022364.fna** | **30** |
| **Escherichia_coli_NA114_uid162139/NC_017644.fna** | **1** |
| **Escherichia_coli_O103_H2_12009_uid41013/NC_013353.fna** | **25** |
| **Escherichia_coli_O104_H4_2009EL_2050_uid175905/NC_018650.fna** | **29** |
| **Escherichia_coli_O104_H4_2009EL_2071_uid176128/NC_018661.fna** | **29** |
| **Escherichia_coli_O104_H4_2011C_3493_uid176127/NC_018658.fna** | **29** |
| **Escherichia_coli_O111_H__11128_uid41023/NC_013364.fna** | **28** |
| **Escherichia_coli_O127_H6_E2348_69_uid59343/NC_011601.fna** | **1** |
| **Escherichia_coli_O157_H7_EC4115_uid59091/NC_011353.fna** | **11** |
| **Escherichia_coli_O157_H7_EDL933_uid57831/NC_002655.fna** | **10** |
| **Escherichia_coli_O157_H7_TW14359_uid59235/NC_013008.fna** | **11** |
| **Escherichia_coli_O157_H7_uid57781/NC_002695.fna** | **11** |
| **Escherichia_coli_O26_H11_11368_uid41021/NC_013361.fna** | **29** |
| **Escherichia_coli_O55_H7_CB9615_uid46655/NC_013941.fna** | **10** |
| **Escherichia_coli_O55_H7_RM12579_uid162153/NC_017656.fna** | **10** |
| **Escherichia_coli_O7_K1_CE10_uid162115/NC_017646.fna** | **6** |
| **Escherichia_coli_O83_H1_NRG_857C_uid161987/NC_017634.fna** | **2** |
| **Escherichia_coli_P12b_uid162061/NC_017663.fna** | **18** |
| **Escherichia_coli_PMV_1_uid219679/NC_022370.fna** | **1** |
| **Escherichia_coli_S88_uid62979/NC_011742.fna** | **2** |
| **Escherichia_coli_SE11_uid59425/NC_011415.fna** | **26** |
| **Escherichia_coli_SE15_uid161939/NC_013654.fna** | **2** |
| **Escherichia_coli_SMS_3_5_uid58919/NC_010498.fna** | **7** |
| **Escherichia_coli_UM146_uid162043/NC_017632.fna** | **1** |
| **Escherichia_coli_UMN026_uid62981/NC_011751.fna** | **15** |
| **Escherichia_coli_UMNK88_uid161991/NC_017641.fna** | **13** |
| **Escherichia_coli_UTI89_uid58541/NC_007946.fna** | **1** |
| **Escherichia_coli_W_uid162011/NC_017635.fna** | **30** |
| **Escherichia_coli_W_uid162101/NC_017664.fna** | **30** |
| **Escherichia_coli_Xuzhou21_uid163995/NC_017906.fna** | **11** |
| Geobacter_bemidjiensis_Bem_uid58749/NC_011146.fna | 42 |
| Geobacter_FRC_32_uid58543/NC_011979.fna | 4 |
| Geobacter_lovleyi_SZ_uid58713/NC_010814.fna | 2 |
| Geobacter_M18_uid55771/NC_014973.fna | 11 |
| Geobacter_M21_uid59037/NC_012918.fna | 42 |
| Geobacter_metallireducens_GS_15_uid57731/NC_007517.fna | 3 |
| Geobacter_uraniireducens_Rf4_uid58475/NC_009483.fna | 17 |
| Halothiobacillus_neapolitanus_c2_uid41317/NC_013422.fna | 4 |
| Klebsiella_oxytoca_E718_uid170256/NC_018106.fna | 2 |
| Klebsiella_oxytoca_KCTC_1686_uid83159/NC_016612.fna | 1 |
| Klebsiella_pneumoniae_1084_uid174151/NC_018522.fna | 10 |
| Klebsiella_pneumoniae_342_uid59145/NC_011283.fna | 38 |
| Klebsiella_pneumoniae_CG43_uid223021/NC_022566.fna | 9 |
| Klebsiella_pneumoniae_HS11286_uid84387/NC_016845.fna | 10 |
| Klebsiella_pneumoniae_JM45_uid215235/NC_022082.fna | 10 |
| Klebsiella_pneumoniae_KCTC_2242_uid162147/NC_017540.fna | 12 |
| Klebsiella_pneumoniae_MGH_78578_uid57619/NC_009648.fna | 8 |
| Klebsiella_pneumoniae_NTUH_K2044_uid59073/NC_012731.fna | 10 |
| Klebsiella_pneumoniae_uid203334/NC_021232.fna | 10 |
| Klebsiella_variicola_At_22_uid42113/NC_013850.fna | 27 |
| Legionella_pneumophila_ATCC_43290_uid86885/NC_016811.fna | 1 |
| Legionella_pneumophila_Lorraine_uid170535/NC_018139.fna | 1 |
| Legionella_pneumophila_Paris_uid58211/NC_006368.fna | 1 |
| Legionella_pneumophila_Philadelphia_1_uid193710/NC_020521.fna | 1 |
| Legionella_pneumophila_Thunder_Bay_uid206517/NC_021350.fna | 1 |
| Magnetospirillum_magneticum_AMB_1_uid58527/NC_007626.fna | 1 |
| Methylibium_petroleiphilum_PM1_uid58085/NC_008825.fna | 2 |
| Methylobacterium_radiotolerans_JCM_2831_uid58845/NC_010505.fna | 42 |
| Methylobacterium_radiotolerans_JCM_2831_uid58845/NC_010510.fna | 4 |
| Methylococcus_capsulatus_Bath_uid57607/NC_002977.fna | 6 |
| Methylomonas_methanica_MC09_uid67363/NC_015572.fna | 3 |
| Micavibrio_aeruginosavorus_ARL_13_uid73585/NC_016026.fna | 18 |
| Micavibrio_EPB_uid194120/NC_020812.fna | 6 |
| Myxococcus_xanthus_DK_1622_uid58003/NC_008095.fna | 1 |
| Neisseria_gonorrhoeae_FA_1090_uid57611/NC_002946.fna | 2 |
| Neisseria_gonorrhoeae_NCCP11945_uid59191/NC_011035.fna | 2 |
| Neisseria_gonorrhoeae_TCDC_NG08107_uid161097/NC_017511.fna | 2 |
| Neisseria_lactamica_020_06_uid60851/NC_014752.fna | 5 |
| Neisseria_meningitidis_053442_uid58587/NC_010120.fna | 2 |
| Neisseria_meningitidis_8013_uid161967/NC_017501.fna | 1 |
| Neisseria_meningitidis_alpha14_uid61649/NC_013016.fna | 4 |
| Neisseria_meningitidis_alpha710_uid161971/NC_017505.fna | 2 |
| Neisseria_meningitidis_FAM18_uid57825/NC_008767.fna | 4 |
| Neisseria_meningitidis_G2136_uid162085/NC_017513.fna | 6 |
| Neisseria_meningitidis_H44_76_uid162083/NC_017516.fna | 3 |
| Neisseria_meningitidis_M01_240149_uid162079/NC_017514.fna | 2 |
| Neisseria_meningitidis_M01_240355_uid162075/NC_017517.fna | 2 |
| Neisseria_meningitidis_M04_240196_uid162081/NC_017515.fna | 4 |
| Neisseria_meningitidis_MC58_uid57817/NC_003112.fna | 2 |
| Neisseria_meningitidis_NZ_05_33_uid162077/NC_017518.fna | 2 |
| Neisseria_meningitidis_WUE_2594_uid162093/NC_017512.fna | 4 |
| Neisseria_meningitidis_Z2491_uid57819/NC_003116.fna | 4 |
| Nitrobacter_winogradskyi_Nb_255_uid58295/NC_007406.fna | 1 |
| Nitrosococcus_halophilus_Nc4_uid46803/NC_013960.fna | 2 |
| Nitrosococcus_oceani_ATCC_19707_uid58403/NC_007484.fna | 2 |
| Oligotropha_carboxidovorans_OM4_uid162135/NC_017538.fna | 13 |
| Oligotropha_carboxidovorans_OM5_uid59155/NC_011386.fna | 13 |
| Oligotropha_carboxidovorans_OM5_uid72795/NC_015684.fna | 13 |
| Pantoea_At_9b_uid55845/NC_014837.fna | 59 |
| Pantoea_At_9b_uid55845/NC_014838.fna | 2 |
| Pelagibacterium_halotolerans_B2_uid74393/NC_016078.fna | 18 |
| Planctomyces_limnophilus_DSM_3776_uid48643/NC_014148.fna | 2 |
| Plautia_stali_symbiont_uid65033/NC_022546.fna | 33 |
| Polaromonas_JS666_uid58207/NC_007948.fna | 69 |
| Polaromonas_naphthalenivorans_CJ2_uid58273/NC_008781.fna | 1 |
| Pseudomonas_aeruginosa_B136_33_uid196598/NC_020912.fna | 2 |
| Pseudomonas_aeruginosa_c7447m_uid219358/NC_022360.fna | 1 |
| Pseudomonas_aeruginosa_DK2_uid168996/NC_018080.fna | 2 |
| Pseudomonas_aeruginosa_LES431_uid232245/NC_023066.fna | 1 |
| Pseudomonas_aeruginosa_LESB58_uid59275/NC_011770.fna | 1 |
| Pseudomonas_aeruginosa_M18_uid162089/NC_017548.fna | 1 |
| Pseudomonas_aeruginosa_MTB_uid231150/NC_023019.fna | 1 |
| Pseudomonas_aeruginosa_NCGM2_S1_uid162173/NC_017549.fna | 3 |
| Pseudomonas_aeruginosa_PA1_uid228931/NC_022808.fna | 1 |
| Pseudomonas_aeruginosa_PA1R_uid228932/NC_022806.fna | 1 |
| Pseudomonas_aeruginosa_PAO1_uid57945/NC_002516.fna | 1 |
| Pseudomonas_aeruginosa_PAO1_VE13_uid225027/NC_022594.fna | 1 |
| Pseudomonas_aeruginosa_PAO1_VE2_uid225026/NC_022591.fna | 1 |
| Pseudomonas_aeruginosa_PAO581_uid219357/NC_022361.fna | 1 |
| Pseudomonas_aeruginosa_RP73_uid209328/NC_021577.fna | 2 |
| Pseudomonas_aeruginosa_SCV20265_uid232358/NC_023149.fna | 1 |
| Pseudomonas_aeruginosa_UCBPP_PA14_uid57977/NC_008463.fna | 1 |
| Pseudomonas_denitrificans_ATCC_13867_uid195459/NC_020829.fna | 36 |
| Pseudomonas_fluorescens_Pf0_1_uid57591/NC_007492.fna | 80 |
| Pseudomonas_mendocina_NK_01_uid66299/NC_015410.fna | 14 |
| Pseudomonas_mendocina_ymp_uid58723/NC_009439.fna | 9 |
| Pseudomonas_resinovorans_NBRC_106553_uid208671/NC_021499.fna | 19 |
| Pseudomonas_stutzeri_A1501_uid58641/NC_009434.fna | 4 |
| Pseudomonas_stutzeri_ATCC_17588___LMG_11199_uid68749/NC_015740.fna | 8 |
| Pseudomonas_stutzeri_CCUG_29243_uid168379/NC_018028.fna | 17 |
| Pseudomonas_stutzeri_DSM_10701_uid170940/NC_018177.fna | 15 |
| Pseudomonas_stutzeri_DSM_4166_uid162113/NC_017532.fna | 2 |
| Pseudomonas_stutzeri_RCH2_uid184342/NC_019936.fna | 3 |
| Pseudomonas_VLB120_uid226717/NC_022739.fna | 1 |
| Pseudoxanthomonas_spadix_BD_a59_uid75113/NC_016147.fna | 1 |
| Ralstonia_eutropha_H16_uid62925/NC_008313.fna | 4 |
| Ralstonia_eutropha_H16_uid62925/NC_008314.fna | 2 |
| Ralstonia_eutropha_JMP134_uid58047/NC_007347.fna | 1 |
| Ralstonia_pickettii_DTP0602_uid222229/NC_022513.fna | 5 |
| Ramlibacter_tataouinensis_TTB310_uid68279/NC_015677.fna | 5 |
| Rhodanobacter_2APBS1_uid74431/NC_020541.fna | 3 |
| Rhodopseudomonas_palustris_BisB5_uid58441/NC_007958.fna | 31 |
| Rhodopseudomonas_palustris_CGA009_uid62901/NC_005296.fna | 22 |
| Rhodopseudomonas_palustris_DX_1_uid43327/NC_014834.fna | 24 |
| Rhodopseudomonas_palustris_HaA2_uid58439/NC_007778.fna | 50 |
| Rhodopseudomonas_palustris_TIE_1_uid58995/NC_011004.fna | 24 |
| Serratia_proteamaculans_568_uid58725/NC_009832.fna | 1 |
| Shigella_boydii_CDC_3083_94_uid58415/NC_010658.fna | 17 |
| Shigella_boydii_Sb227_uid58215/NC_007613.fna | 19 |
| Shigella_dysenteriae_1617_uid229875/NC_022912.fna | 11 |
| Shigella_dysenteriae_Sd197_uid58213/NC_007606.fna | 12 |
| Shigella_flexneri_2002017_uid159233/NC_017328.fna | 21 |
| Shigella_flexneri_2a_2457T_uid57991/NC_004741.fna | 21 |
| Shigella_flexneri_2a_301_uid62907/NC_004337.fna | 21 |
| Shigella_flexneri_5_8401_uid58583/NC_008258.fna | 21 |
| Shigella_sonnei_53G_uid84383/NC_016822.fna | 24 |
| Shigella_sonnei_Ss046_uid58217/NC_007384.fna | 24 |
| Sideroxydans_lithotrophicus_ES_1_uid46801/NC_013959.fna | 29 |
| Sphingobium_chlorophenolicum_L_1_uid52597/NC_015593.fna | 1 |
| Sphingobium_SYK_6_uid73353/NC_015976.fna | 2 |
| Sphingomonas_MM_1_uid193771/NC_020561.fna | 2 |
| Sphingomonas_wittichii_RW1_uid58691/NC_009511.fna | 1 |
| Sphingopyxis_alaskensis_RB2256_uid58351/NC_008048.fna | 6 |
| Starkeya_novella_DSM_506_uid48815/NC_014217.fna | 4 |
| Sulfuricella_denitrificans_skB26_uid170240/NC_022357.fna | 25 |
| Synechococcus_JA_3_3Ab_uid58535/NC_007775.fna | 1 |
| Thioalkalivibrio_nitratireducens_DSM_14787_uid184011/NC_019902.fna | 1 |
| Thioalkalivibrio_sulfidophilus_HL_EbGr7_uid59179/NC_011901.fna | 9 |
| Thioflavicoccus_mobilis_8321_uid184343/NC_019940.fna | 6 |
| Variovorax_paradoxus_B4_uid218005/NC_022234.fna | 2 |
| Variovorax_paradoxus_B4_uid218005/NC_022247.fna | 18 |
| Variovorax_paradoxus_EPS_uid62107/NC_014931.fna | 34 |
| Variovorax_paradoxus_S110_uid59437/NC_012791.fna | 36 |
| Variovorax_paradoxus_S110_uid59437/NC_012792.fna | 1 |
| Xanthomonas_axonopodis_citri_306_uid57889/NC_003919.fna | 2 |
| Xanthomonas_axonopodis_citrumelo_F1_uid73179/NC_016010.fna | 1 |
| Xanthomonas_axonopodis_Xac29_1_uid193774/NC_020800.fna | 3 |
| Xanthomonas_campestris_8004_uid57595/NC_007086.fna | 2 |
| Xanthomonas_campestris_ATCC_33913_uid57887/NC_003902.fna | 2 |
| Xanthomonas_campestris_raphani_756C_uid159539/NC_017271.fna | 1 |
| Xanthomonas_campestris_uid61643/NC_010688.fna | 2 |
| Xanthomonas_campestris_vesicatoria_85_10_uid58321/NC_007508.fna | 1 |
| Xanthomonas_citri_Aw12879_uid194444/NC_020815.fna | 2 |
| Xanthomonas_fuscans_4834_R_uid222814/NC_022541.fna | 1 |

**Table S2.** Flanking genes of TM_ECOs_ in *E. coli*

| **5‘ gene** | **5‘ gene locus tag** | **5‘ gene orientation** | **Separation (nt)** | **TM No** | **Separation (nt)2** | **3‘ gene** | **3‘ gene locus tag** | **3‘ gene orientation** | **Operon location** |
| --- | --- | --- | --- | --- | --- | --- | --- | --- | --- |
| *hrpB* | [b0148](http://www.kegg.jp/dbget-bin/www_bget?eco:b0148) | sense + | 13 | 1 | 150 | *mrcB* | [b0149](http://www.kegg.jp/dbget-bin/www_bget?eco:b0149) | sense + |  |
| *hrpB* | [b0148](http://www.kegg.jp/dbget-bin/www_bget?eco:b0148) | sense + | 63 | 2 | 98 | *mrcB* | [b0149](http://www.kegg.jp/dbget-bin/www_bget?eco:b0149) | sense+ |  |
| *yagA* | [b0267](http://www.kegg.jp/dbget-bin/www_bget?eco:b0267) | antisense - | 117 | 3 | 141 | *yagE* | [b0268](http://www.kegg.jp/dbget-bin/www_bget?eco:b0268) | sense+ | 5' of yagA/B/N (no exp. evidence) and 5' of yagE/F (no exp. evidence) |
| *yagI* | [b0272](http://www.kegg.jp/dbget-bin/www_bget?eco:b0272) | antisense - | 83 | 4 | 21 | *argF* | [b0273](http://www.kegg.jp/dbget-bin/www_bget?eco:b0273) | antisense- |  |
| *tauD* | [b0368](http://www.kegg.jp/dbget-bin/www_bget?eco:b0368) | sense+ | 17 | 5 | 51 | *hemB* | [b0369](http://www.kegg.jp/dbget-bin/www_bget?eco:b0369) | antisense- | 3' of tauA/B/C/D, 3'of hemB |
|
| *adk* | [b0474](http://www.kegg.jp/dbget-bin/www_bget?eco:b0474) | sense+ | 23 | 6 | 176 | *hemH* | [b0475](http://www.kegg.jp/dbget-bin/www_bget?eco:b0475) | sense+ |  |
| *fepB* | [b0592](http://www.kegg.jp/dbget-bin/www_bget?eco:b0592) | antisense - | 67 | 7 | 270 | *entC* | [b0593](http://www.kegg.jp/dbget-bin/www_bget?eco:b0593) | sense+ | 5' of ent C/E/B/A/H |
|
| *fepB* | [b0592](http://www.kegg.jp/dbget-bin/www_bget?eco:b0592) | antisense - | 130 | 8 | 208 | *entC* | [b0593](http://www.kegg.jp/dbget-bin/www_bget?eco:b0593) | sense+ | 5' of ent C/E/B/A/H |
|
| *fabI* | [b1288](http://www.kegg.jp/dbget-bin/www_bget?eco:b1288) | antisense - | 164 | 9 | 167 | *ycjD* | [b1289](http://www.kegg.jp/dbget-bin/www_bget?eco:b1289) | antisense- |  |
| *ygfF* | [b2902](http://www.kegg.jp/dbget-bin/www_bget?eco:b2902) | antisense- | 85 | 10 | 144 | *gcvP* | [b2903](http://www.kegg.jp/dbget-bin/www_bget?eco:b2903) | antisense- | 3' of gcvT/H/P (no exp. evidence) |
| *ygfF* | [b2902](http://www.kegg.jp/dbget-bin/www_bget?eco:b2902) | antisense- | 150 | 11 | 80 | *gcvP* | [b2903](http://www.kegg.jp/dbget-bin/www_bget?eco:b2903) | antisense- | 3' of gcvT/H/P (no exp. evidence) |
|
| *alx* | [b3088](http://www.kegg.jp/dbget-bin/www_bget?eco:b3088) | sense+ | 53 | 12 | 310 | *sstT* | [b3089](http://www.kegg.jp/dbget-bin/www_bget?eco:b3089) | sense+ |  |
| *alx* | [b3088](http://www.kegg.jp/dbget-bin/www_bget?eco:b3088) | sense+ | 116 | 13 | 245 | *sstT* | [b3089](http://www.kegg.jp/dbget-bin/www_bget?eco:b3089) | sense+ |  |
| *aaeR* | [b3243](http://www.kegg.jp/dbget-bin/www_bget?eco:b3243) | sense+ | 44 | 14 | 53 | *tldD* | [b3244](http://www.kegg.jp/dbget-bin/www_bget?eco:b3244) | antisense- |  |
| *frlR* | [b3375](http://www.kegg.jp/dbget-bin/www_bget?eco:b3375) | sense+ | 73 | 15 | 41 | *yhfS* | [b3376](http://www.kegg.jp/dbget-bin/www_bget?eco:b3376) | antisense- | 3' of yhfX/W-php- yhfU/T/S (no exp. evidence) |
|
| *zntA* | [b3469](http://www.kegg.jp/dbget-bin/www_bget?eco:b3469) | sense+ | 33 | 16 | 31 | *tusA* | [b3470](http://www.kegg.jp/dbget-bin/www_bget?eco:b3470) | antisense- |  |
| *lldD* | [b3605](http://www.kegg.jp/dbget-bin/www_bget?eco:b3605) | sense+ | 11 | 17 | 153 | *trmL* | [b3606](http://www.kegg.jp/dbget-bin/www_bget?eco:b3606) | sense+ | 3' of IIdP/R/D (no exp. evidence) |
|
| *lldD* | [b3605](http://www.kegg.jp/dbget-bin/www_bget?eco:b3605) | sense+ | 102 | 18 | 57 | *trmL* | [b3606](http://www.kegg.jp/dbget-bin/www_bget?eco:b3606) | sense+ | 3' of IIdP/R/D (no exp. evidence) |
|
| *pstB* | [b3725](http://www.kegg.jp/dbget-bin/www_bget?eco:b3725) | antisense- | 128 | 19 | 19 | *pstA* | [b3726](http://www.kegg.jp/dbget-bin/www_bget?eco:b3726) | antisense- | middle of operon pstS/C/A/B-phoU |
|
| *ilvC* | [b3774](http://www.kegg.jp/dbget-bin/www_bget?eco:b3774) | sense+ | 14 | 20 | 39 | *ppiC* | [b3775](http://www.kegg.jp/dbget-bin/www_bget?eco:b3775) | antisense- |  |
| *yihR* | [b3879](http://www.kegg.jp/dbget-bin/www_bget?eco:b3879) | antisense- | 49 | 21 | 27 | *yhiS* | [b3504](http://www.kegg.jp/dbget-bin/www_bget?eco:b3504) | antisense- |  |
| *yjdP* | [b4487](http://www.kegg.jp/dbget-bin/www_bget?eco:b4487) | sense+ | 88 | 22 | 23 | *phnP* | [b4092](http://www.kegg.jp/dbget-bin/www_bget?eco:b4092) | antisense- | 3' of phnC/D/E/F/G/H/I/J/K/L/M/N/O/P (no exp. Evidence) |
|
| *imH* | [b4320](http://www.kegg.jp/dbget-bin/www_bget?eco:b4320) | sense+ | 137 | 23 | 70 | *gntP* | [b4321](http://www.kegg.jp/dbget-bin/www_bget?eco:b4321) | antisense- | 3' of fimA/I/C/D/F/G/H |
|

**Table S3.** Evaluation of genomic instability studies of the 23 TM_ECOs_ found in *E. coli* MG1655. Each motif is assigned to one of the different categories for each particular genome (genomes listed in Table S7).

| **TM No** | **no change** | **region missing** | | **intergenic deletions** | | | **not found** |
| --- | --- | --- | --- | --- | --- | --- | --- |
|  |  | **TM missing** | **TM mutated** | **TM missing** | **TM mutated** | **palindrome effect** |  |
| TM_ECO_ 1/2 | 33 |  |  | 21 |  | 2 |  |
| TM_ECO_ 3 | 4 |  |  |  |  |  | 52 |
| TM_ECO_ 4 | 4 |  |  |  |  |  | 52 |
| TM_ECO_ 5 | 27 |  | 1 | 22 | 6 |  |  |
| TM_ECO_ 6 | 28 |  |  | 11 | 17 |  |  |
| TM_ECO_ 7/8 | 26 |  |  | 17 | 13 |  |  |
| TM_ECO_ 9 | 16 | 9 | 17 |  | 13 |  | 1 |
| TM_ECO_ 10/11 | 28 | 28 |  |  |  |  |  |
| TM_ECO_ 12/13 | 27 |  |  |  | 11 | 18 |  |
| TM_ECO_ 14 | 20 |  |  | 1 | 35 |  |  |
| TM_ECO_ 15 | 28 | 13 | 15 |  |  |  |  |
| TM_ECO_ 16 | 14 |  |  |  | 42 |  |  |
| TM_ECO_ 17/18 | 11 |  |  | 21 | 14 | 10 |  |
| TM_ECO_ 19 | 20 |  |  | 9 | 27 |  |  |
| TM_ECO_ 20 | 28 |  |  | 19 | 9 |  |  |
| TM_ECO_ 21 | 20 |  |  |  | 14 |  | 22 |
| TM_ECO_ 22 | 28 |  |  | 8 | 20 |  |  |
| TM_ECO_ 23 | 20 | 7 | 10 | 4 | 14 |  | 1 |

**Table S4.** Control regions analyzed for genomic instability in *E. coli* MG1655. Genomic range of control regions is indicated.

| **control group 1** | | **control group 2** | | **control group 3** | |
| --- | --- | --- | --- | --- | --- |
| start | end | start | end | start | end |
| 1892791 | 1893870 | 990061 | 991180 | 778538 | 779587 |
| 2651670 | 2652959 | 770451 | 771616 | 4588729 | 4589773 |
| 4190011 | 4191099 | 775855 | 776860 | 3552213 | 3553320 |
| 3005531 | 3006557 | 1084321 | 1085336 | 367903 | 369012 |
| 1690811 | 1691872 | 1957862 | 1958871 | 2231277 | 2232320 |
| 3586371 | 3587411 | 377759 | 378910 | 499642 | 500640 |
| 1388311 | 1389358 | 363084 | 364145 | 112891 | 113893 |
| 1290876 | 1291940 | 1196126 | 1197128 | 239791 | 240816 |
| 3321371 | 3322414 | 528712 | 529723 | 3605411 | 3606512 |
| 2559711 | 2560740 | 4560326 | 4561326 | 4521491 | 4522654 |
| 2628578 | 2629612 | 2316436 | 2317457 | 2917422 | 2918521 |
| 2494160 | 2495188 | 4487978 | 4488980 | 2122483 | 2123610 |
| 2620428 | 2621474 | 3771502 | 3772593 | 1100107 | 1101228 |
| 4228961 | 4230004 | 3278313 | 3279351 | 2733611 | 2734747 |
| 1108629 | 1109663 | 1613008 | 1614097 | 62736 | 63835 |
| 2733738 | 2734756 | 376221 | 377344 | 140820 | 141920 |
| 519221 | 520300 | 4312633 | 4313700 | 4541228 | 4542361 |
| 1338730 | 1339780 | 1664686 | 1665820 | 1123535 | 1124550 |
| 3498771 | 3500065 | 1491965 | 1493010 | 3015295 | 3016310 |

**Table S5.** Description of the different TM loci defined for all TM_ECOs_ found in the 56 *E. coli* genomes (Table S7)

| **TM_ECO_ No** | **blocks** | **strand** | **start** | **end** | **length** |
| --- | --- | --- | --- | --- | --- |
| TM locus 1 | 2_56|2_56 | - | 1071 | 7254 | 6184 |
| TM locus 2 | 3_56|3_56 | - | 28627 | 42351 | 13725 |
| TM locus 3 | 3_56|3_56 | - | 28627 | 42351 |  |
| TM locus 4 | 4_56|4_56 | - | 3028 | 36830 | 33803 |
| TM locus 5 | 277_56|5_56 | + | 62854 | 66351 | 3498 |
| TM locus 6 | 6_56|6_56 | - | 12880 | 12923 | 44 |
| TM locus 7 | 9_56|9_56 | + | 14395 | 14580 | 186 |
| TM locus 8 | 9_56|9_56 | - | 14395 | 14580 |  |
| TM locus 9 | 9_56|9_56 | - | 21882 | 21951 | 70 |
| TM locus 10 | 10_56|10_56 | + | 24842 | 36460 | 11619 |
| TM locus 11 | 16_56|16_56 | - | 44077 | 44367 | 291 |
| TM locus 12 | 18_56|41_56 | - | 39293 | 39631 | 339 |
| TM locus 13 | 21_56|21_56 | + | 1 | 7063 | 7063 |
| TM locus 14 | 21_56|21_56 | - | 11093 | 11257 | 165 |
| TM locus 15 | 21_56|21_56 | + | 11093 | 11257 |  |
| TM locus 16 | 21_56|21_56 | + | 16496 | 16650 | 155 |
| TM locus 17 | 21_56|21_56 | - | 16496 | 16650 |  |
| TM locus 18 | 113_56|28_56 | + | 17193 | 21456 | 4264 |
| TM locus 19 | 41_56|41_56 | + | 1 | 443 | 443 |
| TM locus 20 | 416_56|47_56 | - | 25196 | 26762 | 1567 |
| TM locus 21 | 200_56|48_56 | - | 9710 | 11456 | 1747 |
| TM locus 22 | 200_56|48_56 | + | 9710 | 11456 |  |
| TM locus 23 | 53_56|53_56 | - | 1448 | 1917 | 470 |
| TM locus 24 | 53_56|53_56 | - | 15264 | 15346 | 83 |
| TM locus 25 | 54_56|54_56 | - | 8698 | 8867 | 170 |
| TM locus 26 | 62_56|62_56 | - | 6885 | 6907 | 23 |
| TM locus 27 | 66_56|66_56 | + | 2654 | 3643 | 990 |
| TM locus 28 | 66_56|66_56 | - | 2654 | 3643 |  |
| TM locus 29 | 67_56|67_56 | + | 13821 | 13928 | 108 |
| TM locus 30 | 67_56|67_56 | - | 13821 | 13928 |  |
| TM locus 31 | 70_56|70_56 | + | 5024 | 5157 | 134 |
| TM locus 32 | 81_56|81_56 | + | 17454 | 17743 | 290 |
| TM locus 33 | 82_56|82_56 | - | 8456 | 8620 | 165 |
| TM locus 34 | 82_56|82_56 | + | 8456 | 8620 |  |
| TM locus 35 | 91_56|91_56 | - | 10154 | 10429 | 276 |
| TM locus 36 | 91_56|91_56 | + | 10154 | 10429 |  |
| TM locus 37 | 97_56|97_56 | + | 2421 | 2615 | 195 |
| TM locus 38 | 99_56|99_56 | + | 3383 | 3707 | 325 |
| TM locus 39 | 99_56|99_56 | + | 7732 | 7845 | 114 |
| TM locus 40 | 103_56|210_56 | - | 13820 | 15654 | 1835 |
| TM locus 41 | 103_56|210_56 | + | 13820 | 15654 |  |
| TM locus 42 | 125_56|125_56 | + | 7861 | 7990 | 130 |
| TM locus 43 | 132_56|132_56 | + | 1496 | 1707 | 212 |
| TM locus 44 | 145_56|145_56 | + | 7092 | 7258 | 167 |
| TM locus 45 | 154_56|154_56 | - | 5186 | 5469 | 284 |
| TM locus 46 | 165_56|165_56 | + | 1 | 7023 | 7023 |
| TM locus 47 | 202_56|202_56 | + | 4125 | 4211 | 87 |
| TM locus 48 | 228_56|228_56 | + | 4499 | 16331 | 11833 |
| TM locus 49 | 273_56|273_56 | + | 4337 | 4634 | 298 |
| TM locus 50 | 326_56|326_56 | + | 1 | 147 | 147 |
| TM locus 51 | 390_56|390_56 | + | 1 | 216 | 216 |
| TM locus 52 * | 78_55|78_55 | - | 2238 | 2321 | 84 |
| TM locus 53 * | 102_55|102_55 | + | 10988 | 23960 | 12973 |
| TM locus 54 * | 102_55|102_55 | - | 10988 | 23960 |  |
| TM locus 55 ** | 383_15|383_15 | - | 1 | 11411 | 11411 |
| TM locus 56 ** | 383_15|383_15 | + | 1 | 11411 |  |
| TM locus 57 ** | 383_15|383_15 | - | 1 | 11411 |  |
| TM locus 58 * | 463_11|463_11 | + | 1 | 105 | 105 |
| TM locus 59 * | 633_5|633_5 | + | 1236 | 8263 | 7028 |
| TM locus 60 * | 1241_7|1241_7 | + | 701 | 732 | 32 |
| TM locus 61 *** | 1381_11|1381_11 | - | 0 | 0 | 0 |
| TM locus 62 *** | 1381_11|1381_11 | + | 0 | 0 |  |
| TM locus 63 | NO|NO |  |  |  |  |

**Table S6.** Sequence variability [nt] calculated for the TM_ECOs_ and the different random control groups. Bold line indicates the average sequence variability.

| **TM_ECO_** | **Random1** | **Random2** | **Random3** | **Random4** |
| --- | --- | --- | --- | --- |
| 6184 | 311 | 1611 | 0 | 0 |
| 13725 | 0 | 0 | 1910 | 0 |
| 33803 | 0 | 0 | 75 | 0 |
| 3498 | 4504 | 0 | 226 | 0 |
| 44 | 0 | 0 | 0 | 863 |
| 186 | 0 | 0 | 12973 | 0 |
| 70 | 4246 | 0 | 0 | 0 |
| 11619 | 4169 | 0 | 1330 | 1917 |
| 291 | 0 | 8779 | 16340 | 19128 |
| 339 | 0 | 0 | 0 | 0 |
| 7063 | 0 | 0 | 0 | 0 |
| 165 | 7891 | 0 | 33803 | 0 |
| 155 | 0 | 13444 | 10967 | 9832 |
| 4264 | 0 | 3459 | 0 | 11619 |
| 443 | 0 | 0 | 6674 | 0 |
| 1567 | 5621 | 0 | 4289 | 0 |
| 1747 | 0 | 0 | 0 | 0 |
| 470 | 14747 | 0 | 0 | 0 |
| 83 | 0 | 3081 | 134 | 0 |
| 170 | 0 | 0 | 13725 | 0 |
| 23 | 0 | 0 | 0 | 0 |
| 990 | 0 | 0 | 0 | 0 |
| 108 | 0 | 0 | 0 | 0 |
| 134 | 0 | 0 | 0 | 31 |
| 290 | 0 | 0 | 0 | 0 |
| 165 | 0 | 0 | 0 | 0 |
| 276 | 6240 | 14050 | 6600 | 0 |
| 195 | 0 | 0 | 0 | 0 |
| 325 | 0 | 0 | 0 | 0 |
| 114 | 0 | 0 | 2707 | 0 |
| 1835 | 14278 | 0 | 0 | 0 |
| 130 | 0 | 10677 | 4685 | 0 |
| 212 | 0 | 0 | 4911 | 1344 |
| 167 | 0 | 33803 | 0 | 2341 |
| 284 | 0 | 0 | 16340 | 6497 |
| 7023 | 0 | 0 | 0 | 0 |
| 87 | 0 | 0 | 0 | 0 |
| 11833 | 3992 | 0 | 0 | 0 |
| 298 | 0 | 0 | 0 | 0 |
| 147 | 0 | 0 | 0 | 0 |
| 216 | 0 | 0 | 0 | 0 |
| 84 | 0 | 1453 | 0 | 0 |
| 12973 | 113 | 0 | 842 | 6145 |
| 11411 | 0 | 0 | 0 | 4911 |
| 105 | 0 | 0 | 0 | 0 |
| 7028 | 0 | 0 | 0 | 0 |
| 32 | 0 | 0 | 1629 | 0 |
| 0 | 0 | 0 | 0 | 0 |
| **2966,063** | **1377,33333** | **1882,4375** | **2920** | **1346,41667** |

**Table S7.** Description of the 56 fully sequenced *E. coli* genomes used for our analysis. Number of TM_ECOs_ is shown for each particular strain.

| **Number of genome** | **Organism/Name** | **Chromosomes** | **Plasmids** | **Number of TM_ECOs_** |
| --- | --- | --- | --- | --- |
| 1 | Escherichia coli O157:H7 str. Sakai | NC_002695.1 | NC_002127.1 NC_002128.1 | 11 |
| 2 | Escherichia coli Xuzhou21 | NC_017906.1 | NC_017903.1 NC_017907.1 | 11 |
| 3 | Escherichia coli O157:H7 str. TW14359 | NC_013008.1 | NC_013010.1 | 11 |
| 4 | Escherichia coli O157:H7 str. EC4115 | NC_011353.1 | NC_011350.1 NC_011351.1 | 11 |
| 5 | Escherichia coli O55:H7 str. CB9615 | NC_013941.1 | NC_013942.1 | 10 |
| 6 | Escherichia coli O55:H7 str. RM12579 | NC_017656.1 | NC_017658.1 NC_017653.1 NC_017654.1 NC_017657.1 NC_017655.1 | 10 |
| 7 | Escherichia coli SE11 | NC_011415.1 | NC_011407.1 NC_011408.1 NC_011411.1 NC_011413.1 NC_011416.1 NC_011419.1 | 26 |
| 8 | Escherichia coli IAI1 | NC_011741.1 | - | 29 |
| 9 | Escherichia coli W | NC_017664.1 | NC_017665.1 NC_017662.1 | 30 |
| 10 | Escherichia coli KO11FL | NC_016902.1 | NC_016903.1 NC_016904.1 | 30 |
| 11 | Escherichia coli LY180 | NC_022364.1 | - | 30 |
| 12 | Escherichia coli APEC O78 | NC_020163.1 | - | 23 |
| 13 | Escherichia coli E24377A | NC_009801.1 | NC_009786.1 NC_009787.1 NC_009788.1 NC_009789.1 NC_009790.1 NC_009791.1 | 27 |
| 14 | Escherichia coli CFT073 | NC_004431.1 | - | 2 |
| 15 | Escherichia coli str. 'clone D i2' | NC_017651.1 | - | 2 |
| 16 | Escherichia coli str. 'clone D i14' | NC_017652.1 | - | 2 |
| 17 | Escherichia coli ABU 83972 | NC_017631.1 | NC_017629.1 | 2 |
| 18 | Escherichia coli 536 | NC_008253.1 | - |  |
| 19 | Escherichia coli LF82 | NC_011993.1 | - | 2 |
| 20 | Escherichia coli O83:H1 str. NRG 857C | NC_017634.1 | NC_017659.1 | 2 |
| 21 | Escherichia coli 042 | NC_017626.1 | NC_017627.1 | 13 |
| 22 | Escherichia coli UTI89 | NC_007946.1 | NC_007941.1 | 1 |
| 23 | Escherichia coli UM146 | NC_017632.1 | NC_017630.1 | 1 |
| 24 | Escherichia coli IHE3034 | NC_017628.1 | - | 1 |
| 25 | Escherichia coli PMV-1 | NC_022370.1 | NC_022371.1 | 1 |
| 26 | Escherichia coli S88 | NC_011742.1 | NC_011747.1 | 2 |
| 27 | Escherichia coli APEC O1 | NC_008563.1 | NC_009838.1 NC_009837.1 | 2 |
| 28 | Escherichia coli str. K-12 substr. MG1655 | NC_000913.3 | - | 23 |
| 29 | Escherichia coli str. K-12 substr. W3110 | NC_007779.1 | - | 23 |
| 30 | Escherichia coli DH1 | NC_017625.1 | - | 23 |
| 31 | Escherichia coli BW2952 | NC_012759.1 | - | 21 |
| 32 | Escherichia coli str. K-12 substr. DH10B | NC_010473.1 | - | 24 |
| 33 | Escherichia coli str. K-12 substr. MDS42 | NC_020518.1 | - | 21 |
| 34 | Escherichia coli ATCC 8739 | NC_010468.1 | - | 20 |
| 35 | Escherichia coli HS | NC_009800.1 | - | 19 |
| 36 | Escherichia coli 55989 | NC_011748.1 | - | 29 |
| 37 | Escherichia coli O104:H4 str. 2009EL-2071 | NC_018661.1 | NC_018662.1 NC_018663.1 | 29 |
| 38 | Escherichia coli O104:H4 str. 2011C-3493 | NC_018658.1 | NC_018659.1 NC_018660.1 NC_018666.1 | 29 |
| 39 | Escherichia coli O104:H4 str. 2009EL-2050 | NC_018650.1 | NC_018652.1 NC_018654.1 NC_018651.1 | 29 |
| 40 | Escherichia coli SE15 | NC_013654.1 | NC_013655.1 | 2 |
| 41 | Escherichia coli JJ1886 | NC_022648.1 | NC_022649.1 NC_022650.1 NC_022651.1 NC_022661.1 NC_022662.1 | 1 |
| 42 | Escherichia coli NA114 | NC_017644.1 | - | 1 |
| 43 | Escherichia coli O26:H11 str. 11368 | NC_013361.1 | NC_013363.1 NC_013362.1 NC_013369.1 NC_014543.1 | 29 |
| 44 | Escherichia coli O111:H- str. 11128 | NC_013364.1 | NC_013366.1 NC_013367.1 NC_013368.1 NC_013365.1 NC_013370.1 | 28 |
| 45 | Escherichia coli ETEC H10407 | NC_017633.1 | NC_017722.1 NC_017724.1 NC_017721.1 NC_017723.1 | 20 |
| 46 | Escherichia coli O103:H2 str. 12009 | NC_013353.1 | NC_013354.1 | 25 |
| 47 | Escherichia coli O127:H6 str. E2348/69 | NC_011601.1 | NC_011602.1 NC_011603.1 | 1 |
| 48 | Escherichia coli P12b | NC_017663.1 | - | 18 |
| 49 | Escherichia coli B str. REL606 | NC_012967.1 | - | 20 |
| 50 | Escherichia coli BL21(DE3) | NC_012971.2 | - | 20 |
| 51 | Escherichia coli 'BL21-Gold(DE3)pLysS AG' | NC_012947.1 | - | 21 |
| 52 | Escherichia coli SMS-3-5 | NC_010498.1 | NC_010485.1 NC_010486.1 NC_010487.1 NC_010488.1 | 7 |
| 53 | Escherichia coli IAI39 | NC_011750.1 | - | 6 |
| 54 | Escherichia coli O7:K1 str. CE10 | NC_017646.1 | NC_017649.1 NC_017650.1 NC_017648.1 NC_017647.1 | 6 |
| 55 | Escherichia coli UMNK88 | NC_017641.1 | NC_017642.1 NC_017639.1 NC_017640.1 NC_017643.1 NC_017645.1 | 13 |
| 56 | Escherichia coli UMNF18 | NZ_AGTD01000001.1 | NZ_AGTD01000002.1 NZ_AGTD01000003.1 NZ_AGTD01000004.1 NZ_AGTD01000005.1 NZ_AGTD01000006.1 | 23 |

**Table S8.** TM_ECO_ sequences identified in the 56 *E. coli* genomes listed in Table S7.

| TM_ECO_ No | code | start | end | sequence 5' to 3' | strain |
| --- | --- | --- | --- | --- | --- |
| 1 | NC_000913 | 164547 | 164580 | CCCTCTCCCTGTGGGAGAGGGCCGGGGTGAGGGC | Escherichia coli str. K-12 substr. MG1655, complete genome. |
| 2 | NC_000913 | 164631 | 164597 | CCCTCTCCCTTGAGGGAGAGGGTTAGGGTGAGGGT | Escherichia coli str. K-12 substr. MG1655, complete genome. |
| 3 | NC_000913 | 282101 | 282136 | CCCTCGCCCCTTTGGGGAGAGGGCCGGGGTGAGGGG | Escherichia coli str. K-12 substr. MG1655, complete genome. |
| 4 | NC_000913 | 289246 | 289279 | CCCTCTCCCTGTGGGAGAGGGCCGGGGTGAGGGC | Escherichia coli str. K-12 substr. MG1655, complete genome. |
| 5 | NC_000913 | 388664 | 388699 | CCCTCGCCCCCTTGGGGAGAGGGTTAGGGTGAGGGG | Escherichia coli str. K-12 substr. MG1655, complete genome. |
| 6 | NC_000913 | 497843 | 497878 | CCCTCGCCCCCTCGGGGAGAGGGTTAGGGTGAGGGG | Escherichia coli str. K-12 substr. MG1655, complete genome. |
| 7 | NC_000913 | 624579 | 624614 | CCCTCGCCCCTTTGGGGAGAGGGTTAGGGTGAGGGG | Escherichia coli str. K-12 substr. MG1655, complete genome. |
| 8 | NC_000913 | 624676 | 624641 | CCCTCTCCCTTCCAGGGTGAGGGCTGGGGTGAGGGT | Escherichia coli str. K-12 substr. MG1655, complete genome. |
| 9 | NC_000913 | 1351239 | 1351204 | CCCTCGCCCCTCTGGGGAGAGGGTTAGGGTGAGGGG | Escherichia coli str. K-12 substr. MG1655, complete genome. |
| 10 | NC_000913 | 3045989 | 3046024 | CCCTCGCCCTTTCAGGGAGAGGGCCGGGGTGAGGGT | Escherichia coli str. K-12 substr. MG1655, complete genome. |
| 11 | NC_000913 | 3046087 | 3046052 | CCCTCGCCCCTTTGGGGAGAGGGTTAGGGTGAGGGG | Escherichia coli str. K-12 substr. MG1655, complete genome. |
| 12 | NC_000913 | 3239599 | 3239634 | CCCTCTCCCTTCCAGGGAGAGGGTCGGGGTGAGGGT | Escherichia coli str. K-12 substr. MG1655, complete genome. |
| 13 | NC_000913 | 3239698 | 3239662 | CCCTCGCCCCGTTTGGGGAGAGGGTTAGGGTGAGGGG | Escherichia coli str. K-12 substr. MG1655, complete genome. |
| 14 | NC_000913 | 3390529 | 3390494 | CCCTCGCCCCTTTGGGGTGAGGGTTAGGGTGAGGGG | Escherichia coli str. K-12 substr. MG1655, complete genome. |
| 15 | NC_000913 | 3504892 | 3504857 | CCCTCGCCCCTTTGGGGAGAGGGTTAGGGTGAGGGG | Escherichia coli str. K-12 substr. MG1655, complete genome. |
| 16 | NC_000913 | 3608684 | 3608719 | CCCTCGCCCCTCTGGGGAGAGGGTTAGGGTGAGGGG | Escherichia coli str. K-12 substr. MG1655, complete genome. |
| 17 | NC_000913 | 3781061 | 3781028 | CCCTCTCCCTGAGGGAGAGGGTTAGGGTGAGGGG | Escherichia coli str. K-12 substr. MG1655, complete genome. |
| 18 | NC_000913 | 3781121 | 3781156 | CCCTCGCCCCTCCGGGGAGAGGGCCGGGGTGAGGGG | Escherichia coli str. K-12 substr. MG1655, complete genome. |
| 19 | NC_000913 | 3908495 | 3908530 | CCCTCGCCCCTCTGGGGAGAGGGTTAGGGTGAGGGG | Escherichia coli str. K-12 substr. MG1655, complete genome. |
| 20 | NC_000913 | 3959491 | 3959458 | CCCTCTCCCTGTGGGAGAGGGTCGGGGTGAGGGC | Escherichia coli str. K-12 substr. MG1655, complete genome. |
| 21 | NC_000913 | 4070452 | 4070487 | CCCTCGCCCCTTTGGGGAGAGGGTTAGGGAGAGGGG | Escherichia coli str. K-12 substr. MG1655, complete genome. |
| 22 | NC_000913 | 4314285 | 4314320 | CCCTCGCCCCTCTGGGGAGAGGGTTAGGGTGAGGGG | Escherichia coli str. K-12 substr. MG1655, complete genome. |
| 23 | NC_000913 | 4549883 | 4549848 | CCCTCGCCCCTCCGGGGAGAGGGTTAGGGTGAGGGG | Escherichia coli str. K-12 substr. MG1655, complete genome. |
| 24 | NC_002695 | 168869 | 168902 | CCCTCTCCCTGTGGGAGAGGGCCGGGGTGAGGGC | Escherichia coli O157:H7 str. Sakai chromosome, complete genome. |
| 25 | NC_002695 | 168953 | 168919 | CCCTCTCCCTTAAGGGAGAGGGTTAGGGTGAGGGT | Escherichia coli O157:H7 str. Sakai chromosome, complete genome. |
| 26 | NC_002695 | 713556 | 713521 | CCCTCGCCCCATCGGGGAGAGGGTTAGGGTGAGGGG | Escherichia coli O157:H7 str. Sakai chromosome, complete genome. |
| 27 | NC_002695 | 1493741 | 1493776 | CCCTCGCCCCTTTGGGGAGAGGGTTAGGGTGAGGGG | Escherichia coli O157:H7 str. Sakai chromosome, complete genome. |
| 28 | NC_002695 | 2959442 | 2959408 | CCCTCGCCCCTTCGGGAGAGGGTTAGGGTGAGGGT | Escherichia coli O157:H7 str. Sakai chromosome, complete genome. |
| 29 | NC_002695 | 3383056 | 3383021 | CCCTCGCCCCTTCGGGGAGAGGGTTAGGGTGAGGGG | Escherichia coli O157:H7 str. Sakai chromosome, complete genome. |
| 30 | NC_002695 | 3979231 | 3979266 | CCCTCTCCCTTCCAGGGAGAGGGTCGGGGTGAGGGT | Escherichia coli O157:H7 str. Sakai chromosome, complete genome. |
| 31 | NC_002695 | 3979330 | 3979294 | CCCTCGCCCCGTTTGGGGAGAGGGTTAGGGTGAGGGG | Escherichia coli O157:H7 str. Sakai chromosome, complete genome. |
| 32 | NC_002695 | 4125763 | 4125728 | CCCTCGCCCCTTTGGGGAGAGGGTTAGGGTGAGGGG | Escherichia coli O157:H7 str. Sakai chromosome, complete genome. |
| 33 | NC_002695 | 4224808 | 4224773 | CCCTCGCCCCTTTGGGGAGAGGGTTAGGGTGAGGGG | Escherichia coli O157:H7 str. Sakai chromosome, complete genome. |
| 34 | NC_002695 | 4695203 | 4695238 | CCCTCGCCCCTCTGGGGAGAGGGTTAGGGTGAGGGG | Escherichia coli O157:H7 str. Sakai chromosome, complete genome. |
| 35 | NC_004431 | 2852458 | 2852423 | CCCTCGCCCCTCTGGGGAGAGGGTTAGGGTGAGGGG | Escherichia coli CFT073 chromosome, complete genome. |
| 36 | NC_004431 | 5144612 | 5144577 | CCCTCGCCCCTCCGGGGAGAGGGTTAGGGTGAGGGG | Escherichia coli CFT073 chromosome, complete genome. |
| 37 | NC_007779 | 164547 | 164580 | CCCTCTCCCTGTGGGAGAGGGCCGGGGTGAGGGC | Escherichia coli str. K-12 substr. W3110, complete genome. |
| 38 | NC_007779 | 164631 | 164597 | CCCTCTCCCTTGAGGGAGAGGGTTAGGGTGAGGGT | Escherichia coli str. K-12 substr. W3110, complete genome. |
| 39 | NC_007779 | 281325 | 281360 | CCCTCGCCCCTTTGGGGAGAGGGCCGGGGTGAGGGG | Escherichia coli str. K-12 substr. W3110, complete genome. |
| 40 | NC_007779 | 288470 | 288503 | CCCTCTCCCTGTGGGAGAGGGCCGGGGTGAGGGC | Escherichia coli str. K-12 substr. W3110, complete genome. |
| 41 | NC_007779 | 387888 | 387923 | CCCTCGCCCCCTTGGGGAGAGGGTTAGGGTGAGGGG | Escherichia coli str. K-12 substr. W3110, complete genome. |
| 42 | NC_007779 | 497067 | 497102 | CCCTCGCCCCCTCGGGGAGAGGGTTAGGGTGAGGGG | Escherichia coli str. K-12 substr. W3110, complete genome. |
| 43 | NC_007779 | 623802 | 623837 | CCCTCGCCCCTTTGGGGAGAGGGTTAGGGTGAGGGG | Escherichia coli str. K-12 substr. W3110, complete genome. |
| 44 | NC_007779 | 623899 | 623864 | CCCTCTCCCTTCCAGGGTGAGGGCTGGGGTGAGGGT | Escherichia coli str. K-12 substr. W3110, complete genome. |
| 45 | NC_007779 | 1352953 | 1352918 | CCCTCGCCCCTCTGGGGAGAGGGTTAGGGTGAGGGG | Escherichia coli str. K-12 substr. W3110, complete genome. |
| 46 | NC_007779 | 3044645 | 3044680 | CCCTCGCCCTTTCAGGGAGAGGGCCGGGGTGAGGGT | Escherichia coli str. K-12 substr. W3110, complete genome. |
| 47 | NC_007779 | 3044743 | 3044708 | CCCTCGCCCCTTTGGGGAGAGGGTTAGGGTGAGGGG | Escherichia coli str. K-12 substr. W3110, complete genome. |
| 48 | NC_007779 | 3238255 | 3238290 | CCCTCTCCCTTCCAGGGAGAGGGTCGGGGTGAGGGT | Escherichia coli str. K-12 substr. W3110, complete genome. |
| 49 | NC_007779 | 3238354 | 3238318 | CCCTCGCCCCGTTTGGGGAGAGGGTTAGGGTGAGGGG | Escherichia coli str. K-12 substr. W3110, complete genome. |
| 50 | NC_007779 | 3390384 | 3390349 | CCCTCGCCCCTTTGGGGTGAGGGTTAGGGTGAGGGG | Escherichia coli str. K-12 substr. W3110, complete genome. |
| 51 | NC_007779 | 3566229 | 3566194 | CCCTCGCCCCTTTGGGGAGAGGGTTAGGGAGAGGGG | Escherichia coli str. K-12 substr. W3110, complete genome. |
| 52 | NC_007779 | 3677190 | 3677223 | CCCTCTCCCTGTGGGAGAGGGTCGGGGTGAGGGC | Escherichia coli str. K-12 substr. W3110, complete genome. |
| 53 | NC_007779 | 3728186 | 3728151 | CCCTCGCCCCTCTGGGGAGAGGGTTAGGGTGAGGGG | Escherichia coli str. K-12 substr. W3110, complete genome. |
| 54 | NC_007779 | 3859294 | 3859259 | CCCTCGCCCCTCCGGGGAGAGGGCCGGGGTGAGGGG | Escherichia coli str. K-12 substr. W3110, complete genome. |
| 55 | NC_007779 | 3859354 | 3859387 | CCCTCTCCCTGAGGGAGAGGGTTAGGGTGAGGGG | Escherichia coli str. K-12 substr. W3110, complete genome. |
| 56 | NC_007779 | 4031731 | 4031696 | CCCTCGCCCCTCTGGGGAGAGGGTTAGGGTGAGGGG | Escherichia coli str. K-12 substr. W3110, complete genome. |
| 57 | NC_007779 | 4135524 | 4135559 | CCCTCGCCCCTTTGGGGAGAGGGTTAGGGTGAGGGG | Escherichia coli str. K-12 substr. W3110, complete genome. |
| 58 | NC_007779 | 4318963 | 4318998 | CCCTCGCCCCTCTGGGGAGAGGGTTAGGGTGAGGGG | Escherichia coli str. K-12 substr. W3110, complete genome. |
| 59 | NC_007779 | 4554563 | 4554528 | CCCTCGCCCCTCCGGGGAGAGGGTTAGGGTGAGGGG | Escherichia coli str. K-12 substr. W3110, complete genome. |
| 60 | NC_007946 | 562046 | 562081 | CCCTCGCCCCTGTGGGGAGAGGGTTAGGGTGAGGGG | Escherichia coli UTI89 chromosome, complete genome. |
| 61 | NC_008563 | 563644 | 563679 | CCCTCGCCCCTGTGGGGAGAGGGTTAGGGTGAGGGG | Escherichia coli APEC O1 chromosome, complete genome. |
| 62 | NC_008563 | 2205660 | 2205694 | CCCTCGCCCCCTGGGGAGAGGGTTAGGGTGAGGGG | Escherichia coli APEC O1 chromosome, complete genome. |
| 63 | NC_009800 | 68479 | 68444 | CCCTCGCCCCTTTGGGGAGAGGGCCGGGGTGAGGGG | Escherichia coli HS, complete genome. |
| 64 | NC_009800 | 163595 | 163628 | CCCTCTCCCTGTGGGAGAGGGCCGGGGTGAGGGC | Escherichia coli HS, complete genome. |
| 65 | NC_009800 | 163679 | 163645 | CCCTCTCCCTTGAGGGAGAGGGTTAGGGTGAGGGT | Escherichia coli HS, complete genome. |
| 66 | NC_009800 | 452609 | 452644 | CCCTCGCCCCCTTGGGGAGAGGGTTAGGGTGAGGGG | Escherichia coli HS, complete genome. |
| 67 | NC_009800 | 563908 | 563943 | CCCTCGCCCCCTCGGGGAGAGGGTTAGGGTGAGGGG | Escherichia coli HS, complete genome. |
| 68 | NC_009800 | 660660 | 660695 | CCCTCGCCCCTTTGGGGAGAGGGTTAGGGTGAGGGG | Escherichia coli HS, complete genome. |
| 69 | NC_009800 | 660757 | 660722 | CCCTCTCCCTTCCAGGGTGAGGGCTGGGGTGAGGGT | Escherichia coli HS, complete genome. |
| 70 | NC_009800 | 1398799 | 1398764 | CCCTCGCCCCTCTGGGGAGAGGGTTAGGGTGAGGGG | Escherichia coli HS, complete genome. |
| 71 | NC_009800 | 3073501 | 3073536 | CCCTCGCCCTTTCAGGGAGAGGGCCGGGGTGAGGGT | Escherichia coli HS, complete genome. |
| 72 | NC_009800 | 3073599 | 3073564 | CCCTCACCCCTTTGGGGAGAGGGTTAGGGTGAGGGG | Escherichia coli HS, complete genome. |
| 73 | NC_009800 | 3434117 | 3434082 | CCCTCGCCCCTTTGGGGAGAGGGTTAGGGTGAGGGG | Escherichia coli HS, complete genome. |
| 74 | NC_009800 | 3549237 | 3549202 | CCCTCGCCCCTTTGGGGAGAGGGTTAGGGTGAGGGG | Escherichia coli HS, complete genome. |
| 75 | NC_009800 | 3654954 | 3654989 | CCCTCGCCCCTCTGGGGAGAGGGTTAGGGTGAGGGG | Escherichia coli HS, complete genome. |
| 76 | NC_009800 | 3935675 | 3935710 | CCCTCGCCCCTCTGGGGAGAGGGTTAGGGTGAGGGG | Escherichia coli HS, complete genome. |
| 77 | NC_009800 | 3987904 | 3987871 | CCCTCTCCCTGTGGGAGAGGGTCGGGGTGAGGGC | Escherichia coli HS, complete genome. |
| 78 | NC_009800 | 4259915 | 4259950 | CCCTCGCCCCTTCGGGGAGAGGGTTAGGGTGAGGGG | Escherichia coli HS, complete genome. |
| 79 | NC_009800 | 4338855 | 4338890 | CCCTCGCCCCTCTGGGGAGAGGGTTAGGGTGAGGGG | Escherichia coli HS, complete genome. |
| 80 | NC_009800 | 4520554 | 4520587 | CCCTCTCCCTTAGGGAGAGGGTTAGGGTGAGGGT | Escherichia coli HS, complete genome. |
| 81 | NC_009800 | 4623889 | 4623856 | CCCTCTCCCTGTGGGAGAGGGCCGGGGTGAGGGC | Escherichia coli HS, complete genome. |
| 82 | NC_009801 | 68414 | 68379 | CCCTCGCCCCTTTGGGGAGAGGGCCGGGGTGAGGGG | Escherichia coli E24377A chromosome, complete genome. |
| 83 | NC_009801 | 166013 | 166046 | CCCTCTCCCTGTGGGAGAGGGCCGGGGTGAGGGC | Escherichia coli E24377A chromosome, complete genome. |
| 84 | NC_009801 | 166097 | 166063 | CCCTCTCCCTTGAGGGAGAGGGTTAGGGTGAGGGT | Escherichia coli E24377A chromosome, complete genome. |
| 85 | NC_009801 | 532990 | 533025 | CCCTCGCCCCCTCGGGGAGAGGGTTAGGGTGAGGGG | Escherichia coli E24377A chromosome, complete genome. |
| 86 | NC_009801 | 647253 | 647288 | CCCTCGCCCCTTTGGGGAGAGGGTTAGGGTGAGGGG | Escherichia coli E24377A chromosome, complete genome. |
| 87 | NC_009801 | 647350 | 647315 | CCCTCTCCCTTCCAGGGTGAGGGCTGGGGTGAGGGT | Escherichia coli E24377A chromosome, complete genome. |
| 88 | NC_009801 | 1212064 | 1212099 | CCCTCGCCCCTTTGGGGAGAGGGTTAGGGTGAGGGG | Escherichia coli E24377A chromosome, complete genome. |
| 89 | NC_009801 | 2526240 | 2526275 | CCCTCTCCCCTTGCGGGAGAGGGTTAGGGTGAGGGG | Escherichia coli E24377A chromosome, complete genome. |
| 90 | NC_009801 | 2812474 | 2812439 | CCCTCGCCCCTTCGGGGAGAGGGTTAGGGTGAGGGG | Escherichia coli E24377A chromosome, complete genome. |
| 91 | NC_009801 | 2983331 | 2983296 | CCCTCGCCCCTTTGGGGAGAGGGTTAGGGTGAGGGG | Escherichia coli E24377A chromosome, complete genome. |
| 92 | NC_009801 | 3249330 | 3249365 | CCCTCGCCCTTTCAGGGAGAGGGCCGGGGTGAGGGT | Escherichia coli E24377A chromosome, complete genome. |
| 93 | NC_009801 | 3249428 | 3249393 | CCCTCCCCCCTTTGGGGAGAGGGTTAGGGTGAGGGG | Escherichia coli E24377A chromosome, complete genome. |
| 94 | NC_009801 | 3532844 | 3532879 | CCCTCGCCCCTTCGGGGAGAGGGTTAGGGTGAGGGG | Escherichia coli E24377A chromosome, complete genome. |
| 95 | NC_009801 | 3819600 | 3819565 | CCCTCGCCCCTTTGGGGAGAGGGTTAGGGTGAGGGG | Escherichia coli E24377A chromosome, complete genome. |
| 96 | NC_009801 | 3827299 | 3827264 | CCCTCGCCCCTTTGGGGAGAGGGTTAGGGTGAGGGG | Escherichia coli E24377A chromosome, complete genome. |
| 97 | NC_009801 | 3926496 | 3926531 | CCCTCGCCCCTCTGGGGAGAGGGTTAGGGTGAGGGG | Escherichia coli E24377A chromosome, complete genome. |
| 98 | NC_009801 | 3987972 | 3987937 | CCCTCTCCCTTTCAGGGAGAGGGCTGGGGTGAGGGT | Escherichia coli E24377A chromosome, complete genome. |
| 99 | NC_009801 | 3988073 | 3988108 | CCCTCGCCCCTTTGGGGAGAGGGTTAGGGTGAGGGG | Escherichia coli E24377A chromosome, complete genome. |
| 100 | NC_009801 | 4170416 | 4170381 | CCCTCGCCCCTTTGGGGAGAGGGCTAGGGTGAGGGG | Escherichia coli E24377A chromosome, complete genome. |
| 101 | NC_009801 | 4281333 | 4281300 | CCCTCTCCCTGTGGGAGAGGGTCGGGGTGAGGGC | Escherichia coli E24377A chromosome, complete genome. |
| 102 | NC_009801 | 4395515 | 4395550 | CCCTCGCCCCTTTGGGGAGAGGGTTAGGGTGAGGGG | Escherichia coli E24377A chromosome, complete genome. |
| 103 | NC_009801 | 4422243 | 4422208 | CCCTCGCCCCTACGGGGAGAGGGTTAGGGTGAGGGG | Escherichia coli E24377A chromosome, complete genome. |
| 104 | NC_009801 | 4422296 | 4422331 | CCCTCGCCCTTTCAGGGAGAGGGCCGGGGTGAGGGT | Escherichia coli E24377A chromosome, complete genome. |
| 105 | NC_009801 | 4646171 | 4646206 | CCCTCGCCCCTCTGGGGAGAGGGTTAGGGTGAGGGG | Escherichia coli E24377A chromosome, complete genome. |
| 106 | NC_009801 | 4655133 | 4655166 | CCCTCTCCCTGTGGGAGAGGGCCGGGGTGAGGGC | Escherichia coli E24377A chromosome, complete genome. |
| 107 | NC_009801 | 4655217 | 4655183 | CCCTCTCCCTTGAGGGAGAGGGTTAGGGTGAGGGG | Escherichia coli E24377A chromosome, complete genome. |
| 108 | NC_009801 | 4959919 | 4959886 | CCCTCTCCCTGAGGGAGAGGGCCGGGGTGAGGGC | Escherichia coli E24377A chromosome, complete genome. |
| 109 | NC_010468 | 390037 | 390072 | CCCTCGCCCCTCTGGGGAGAGGGTTAGGGTGAGGGG | Escherichia coli ATCC 8739 chromosome, complete genome. |
| 110 | NC_010468 | 505168 | 505203 | CCCTCGCCCCTTTGGGGAGAGGGTTAGGGTGAGGGG | Escherichia coli ATCC 8739 chromosome, complete genome. |
| 111 | NC_010468 | 656220 | 656256 | CCCTCGCCCCGTTTGGGGAGAGGGTTAGGGTGAGGGG | Escherichia coli ATCC 8739 chromosome, complete genome. |
| 112 | NC_010468 | 656319 | 656284 | CCCTCTCCCTTCCAGGGAGAGGGTCGGGGTGAGGGT | Escherichia coli ATCC 8739 chromosome, complete genome. |
| 113 | NC_010468 | 866657 | 866692 | CCCTCGCCCCTTTGGGGAGAGGGTTAGGGTGAGGGG | Escherichia coli ATCC 8739 chromosome, complete genome. |
| 114 | NC_010468 | 2575392 | 2575427 | CCCTCGCCCCTCTGGGGAGAGGGTTAGGGTGAGGGG | Escherichia coli ATCC 8739 chromosome, complete genome. |
| 115 | NC_010468 | 3332560 | 3332595 | CCCTCTCCCTTCCAGGGTGAGGGCTGGGGTGAGGGT | Escherichia coli ATCC 8739 chromosome, complete genome. |
| 116 | NC_010468 | 3332657 | 3332622 | CCCTCGCCCCTTTGGGGAGAGGGTTAGGGTGAGGGG | Escherichia coli ATCC 8739 chromosome, complete genome. |
| 117 | NC_010468 | 3443717 | 3443682 | CCCTCGCCCCCTCGGGGAGAGGGTTAGGGTGAGGGG | Escherichia coli ATCC 8739 chromosome, complete genome. |
| 118 | NC_010468 | 3569813 | 3569778 | CCCTCGCCCCCTTGGGGAGAGGGTTAGGGTGAGGGG | Escherichia coli ATCC 8739 chromosome, complete genome. |
| 119 | NC_010468 | 3694841 | 3694806 | CCCTCGCCCCTTTGGGGAGAGGGTTAGGGTGAGGGG | Escherichia coli ATCC 8739 chromosome, complete genome. |
| 120 | NC_010468 | 3694882 | 3694915 | CCCTCTCCCTGTGGGAGAGGGTTGGGGTGAGGGC | Escherichia coli ATCC 8739 chromosome, complete genome. |
| 121 | NC_010468 | 3835162 | 3835196 | CCCTCTCCCTTGAGGGAGAGGGTTAGGGTGAGGGT | Escherichia coli ATCC 8739 chromosome, complete genome. |
| 122 | NC_010468 | 3835246 | 3835213 | CCCTCTCCCTGTGGGAGAGGGCCGGGGTGAGGGC | Escherichia coli ATCC 8739 chromosome, complete genome. |
| 123 | NC_010468 | 4021997 | 4022030 | CCCTCTCCCTGTGGGAGAGGGCCGGGGTGAGGGC | Escherichia coli ATCC 8739 chromosome, complete genome. |
| 124 | NC_010468 | 4095316 | 4095351 | CCCTCGCCCCTCCGGGGAGAGGGTTAGGGTGAGGGG | Escherichia coli ATCC 8739 chromosome, complete genome. |
| 125 | NC_010468 | 4102216 | 4102181 | CCCTCGCCCCTTTGGGGAGAGGGTTAGGGTGAGGGG | Escherichia coli ATCC 8739 chromosome, complete genome. |
| 126 | NC_010468 | 4319676 | 4319641 | CCCTCGCCCCTCTGGGGAGAGGGTTAGGGTGAGGGG | Escherichia coli ATCC 8739 chromosome, complete genome. |
| 127 | NC_010468 | 4670493 | 4670526 | CCCTCTCCCTGTGGGAGAGGGTCGGGGTGAGGGC | Escherichia coli ATCC 8739 chromosome, complete genome. |
| 128 | NC_010468 | 4721492 | 4721457 | CCCTCGCCCCTCTGGGGAGAGGGTTAGGGTGAGGGG | Escherichia coli ATCC 8739 chromosome, complete genome. |
| 129 | NC_010473 | 138651 | 138684 | CCCTCTCCCTGTGGGAGAGGGCCGGGGTGAGGGC | Escherichia coli str. K-12 substr. DH10B chromosome, complete genome. |
| 130 | NC_010473 | 138735 | 138701 | CCCTCTCCCTTGAGGGAGAGGGTTAGGGTGAGGGT | Escherichia coli str. K-12 substr. DH10B chromosome, complete genome. |
| 131 | NC_010473 | 255429 | 255464 | CCCTCGCCCCTTTGGGGAGAGGGCCGGGGTGAGGGG | Escherichia coli str. K-12 substr. DH10B chromosome, complete genome. |
| 132 | NC_010473 | 264020 | 264053 | CCCTCTCCCTGTGGGAGAGGGCCGGGGTGAGGGC | Escherichia coli str. K-12 substr. DH10B chromosome, complete genome. |
| 133 | NC_010473 | 327219 | 327254 | CCCTCGCCCCCTTGGGGAGAGGGTTAGGGTGAGGGG | Escherichia coli str. K-12 substr. DH10B chromosome, complete genome. |
| 134 | NC_010473 | 436398 | 436433 | CCCTCGCCCCCTCGGGGAGAGGGTTAGGGTGAGGGG | Escherichia coli str. K-12 substr. DH10B chromosome, complete genome. |
| 135 | NC_010473 | 563134 | 563169 | CCCTCGCCCCTTTGGGGAGAGGGTTAGGGTGAGGGG | Escherichia coli str. K-12 substr. DH10B chromosome, complete genome. |
| 136 | NC_010473 | 563231 | 563196 | CCCTCTCCCTTCCAGGGTGAGGGCTGGGGTGAGGGT | Escherichia coli str. K-12 substr. DH10B chromosome, complete genome. |
| 137 | NC_010473 | 676394 | 676429 | CCCTCGCCCCTTTGGGGAGAGGGTTAGGGTGAGGGG | Escherichia coli str. K-12 substr. DH10B chromosome, complete genome. |
| 138 | NC_010473 | 676491 | 676456 | CCCTCTCCCTTCCAGGGTGAGGGCTGGGGTGAGGGT | Escherichia coli str. K-12 substr. DH10B chromosome, complete genome. |
| 139 | NC_010473 | 1438659 | 1438624 | CCCTCGCCCCTCTGGGGAGAGGGTTAGGGTGAGGGG | Escherichia coli str. K-12 substr. DH10B chromosome, complete genome. |
| 140 | NC_010473 | 3137881 | 3137916 | CCCTCGCCCTTTCAGGGAGAGGGCCGGGGTGAGGGT | Escherichia coli str. K-12 substr. DH10B chromosome, complete genome. |
| 141 | NC_010473 | 3137979 | 3137944 | CCCTCGCCCCTTTGGGGAGAGGGTTAGGGTGAGGGG | Escherichia coli str. K-12 substr. DH10B chromosome, complete genome. |
| 142 | NC_010473 | 3335366 | 3335401 | CCCTCTCCCTTCCAGGGAGAGGGTCGGGGTGAGGGT | Escherichia coli str. K-12 substr. DH10B chromosome, complete genome. |
| 143 | NC_010473 | 3335465 | 3335429 | CCCTCGCCCCGTTTGGGGAGAGGGTTAGGGTGAGGGG | Escherichia coli str. K-12 substr. DH10B chromosome, complete genome. |
| 144 | NC_010473 | 3486296 | 3486261 | CCCTCGCCCCTTTGGGGTGAGGGTTAGGGTGAGGGG | Escherichia coli str. K-12 substr. DH10B chromosome, complete genome. |
| 145 | NC_010473 | 3600659 | 3600624 | CCCTCGCCCCTTTGGGGAGAGGGTTAGGGTGAGGGG | Escherichia coli str. K-12 substr. DH10B chromosome, complete genome. |
| 146 | NC_010473 | 3704452 | 3704487 | CCCTCGCCCCTCTGGGGAGAGGGTTAGGGTGAGGGG | Escherichia coli str. K-12 substr. DH10B chromosome, complete genome. |
| 147 | NC_010473 | 3876661 | 3876628 | CCCTCTCCCTGAGGGAGAGGGTTAGGGTGAGGGG | Escherichia coli str. K-12 substr. DH10B chromosome, complete genome. |
| 148 | NC_010473 | 3876721 | 3876756 | CCCTCGCCCCTCCGGGGAGAGGGCCGGGGTGAGGGG | Escherichia coli str. K-12 substr. DH10B chromosome, complete genome. |
| 149 | NC_010473 | 4004102 | 4004137 | CCCTCGCCCCTCTGGGGAGAGGGTTAGGGTGAGGGG | Escherichia coli str. K-12 substr. DH10B chromosome, complete genome. |
| 150 | NC_010473 | 4056434 | 4056401 | CCCTCTCCCTGTGGGAGAGGGTCGGGGTGAGGGC | Escherichia coli str. K-12 substr. DH10B chromosome, complete genome. |
| 151 | NC_010473 | 4168172 | 4168207 | CCCTCGCCCCTTTGGGGAGAGGGTTAGGGAGAGGGG | Escherichia coli str. K-12 substr. DH10B chromosome, complete genome. |
| 152 | NC_010473 | 4412670 | 4412705 | CCCTCGCCCCTCTGGGGAGAGGGTTAGGGTGAGGGG | Escherichia coli str. K-12 substr. DH10B chromosome, complete genome. |
| 153 | NC_010498 | 261597 | 261633 | CCCTCGCCCCATCCGGGGAGAGGGTTAGGGTGAGGGG | Escherichia coli SMS-3-5 chromosome, complete genome. |
| 154 | NC_010498 | 272864 | 272831 | CCCTCTCCCTGTGGGAGAGGGTCGGGGTGAGGGC | Escherichia coli SMS-3-5 chromosome, complete genome. |
| 155 | NC_010498 | 272905 | 272940 | CCCTCGCCCCTTTGGGGAGAGGGTTAGGGGGAGGGG | Escherichia coli SMS-3-5 chromosome, complete genome. |
| 156 | NC_010498 | 695776 | 695809 | CCCTCGCCCCTCGGGTGAGGGTTAGGGTGAGGGG | Escherichia coli SMS-3-5 chromosome, complete genome. |
| 157 | NC_010498 | 2652608 | 2652573 | CCCTCGCCCCTTTGGGGAGAGGGTTAGGGTGAGGGG | Escherichia coli SMS-3-5 chromosome, complete genome. |
| 158 | NC_010498 | 4815701 | 4815735 | CCCTCTCCCTTGAGGGAGAGGGTTAGGGTGAGGGG | Escherichia coli SMS-3-5 chromosome, complete genome. |
| 159 | NC_010498 | 4843415 | 4843450 | CCCTCGCCCCTTTGGGGAGAGGGTTAGGGTGAGGGG | Escherichia coli SMS-3-5 chromosome, complete genome. |
| 160 | NC_011353 | 168850 | 168883 | CCCTCTCCCTGTGGGAGAGGGCCGGGGTGAGGGC | Escherichia coli O157:H7 str. EC4115 chromosome, complete genome. |
| 161 | NC_011353 | 168934 | 168900 | CCCTCTCCCTTAAGGGAGAGGGTTAGGGTGAGGGT | Escherichia coli O157:H7 str. EC4115 chromosome, complete genome. |
| 162 | NC_011353 | 716831 | 716796 | CCCTCGCCCCATCGGGGAGAGGGTTAGGGTGAGGGG | Escherichia coli O157:H7 str. EC4115 chromosome, complete genome. |
| 163 | NC_011353 | 1437327 | 1437362 | CCCTCGCCCCTTTGGGGAGAGGGTTAGGGTGAGGGG | Escherichia coli O157:H7 str. EC4115 chromosome, complete genome. |
| 164 | NC_011353 | 3000804 | 3000770 | CCCTCGCCCCTTCGGGAGAGGGTTAGGGTGAGGGT | Escherichia coli O157:H7 str. EC4115 chromosome, complete genome. |
| 165 | NC_011353 | 3487173 | 3487138 | CCCTCGCCCCTTCGGGGAGAGGGTTAGGGTGAGGGG | Escherichia coli O157:H7 str. EC4115 chromosome, complete genome. |
| 166 | NC_011353 | 4082314 | 4082349 | CCCTCTCCCTTCCAGGGAGAGGGTCGGGGTGAGGGT | Escherichia coli O157:H7 str. EC4115 chromosome, complete genome. |
| 167 | NC_011353 | 4082413 | 4082377 | CCCTCGCCCCGTTTGGGGAGAGGGTTAGGGTGAGGGG | Escherichia coli O157:H7 str. EC4115 chromosome, complete genome. |
| 168 | NC_011353 | 4227530 | 4227495 | CCCTCGCCCCTTTGGGGAGAGGGTTAGGGTGAGGGG | Escherichia coli O157:H7 str. EC4115 chromosome, complete genome. |
| 169 | NC_011353 | 4327888 | 4327853 | CCCTCGCCCCTTTGGGGAGAGGGTTAGGGTGAGGGG | Escherichia coli O157:H7 str. EC4115 chromosome, complete genome. |
| 170 | NC_011353 | 4798298 | 4798333 | CCCTCGCCCCTCTGGGGAGAGGGTTAGGGTGAGGGG | Escherichia coli O157:H7 str. EC4115 chromosome, complete genome. |
| 171 | NC_011415 | 68408 | 68373 | CCCTCGCCCCTTTGGGGAGAGGGCCGGGGTGAGGGG | Escherichia coli SE11 chromosome, complete genome. |
| 172 | NC_011415 | 165911 | 165944 | CCCTCTCCCTGTGGGAGAGGGCCGGGGTGAGGGC | Escherichia coli SE11 chromosome, complete genome. |
| 173 | NC_011415 | 165995 | 165961 | CCCTCTCCCTTGAGGGAGAGGGTTAGGGTGAGGGT | Escherichia coli SE11 chromosome, complete genome. |
| 174 | NC_011415 | 431716 | 431751 | CCCTCGCCCCCTTGGGGAGAGGGTTAGGGTGAGGGG | Escherichia coli SE11 chromosome, complete genome. |
| 175 | NC_011415 | 541673 | 541708 | CCCTCGCCCCCTCGGGGAGAGGGTTAGGGTGAGGGG | Escherichia coli SE11 chromosome, complete genome. |
| 176 | NC_011415 | 717092 | 717057 | CCCTCGCCCCATCGGGGAGAGGGTTAGGGTGAGGGG | Escherichia coli SE11 chromosome, complete genome. |
| 177 | NC_011415 | 1217464 | 1217499 | CCCTCGCCCCTTTGGGGAGAGGGTTAGGGTGAGGGG | Escherichia coli SE11 chromosome, complete genome. |
| 178 | NC_011415 | 2592880 | 2592915 | CCCTCTCCCCTTGCGGGAGAGGGTTAGGGTGAGGGG | Escherichia coli SE11 chromosome, complete genome. |
| 179 | NC_011415 | 2921462 | 2921427 | CCCTCGCCCCTTCGGGGAGAGGGTTAGGGTGAGGGG | Escherichia coli SE11 chromosome, complete genome. |
| 180 | NC_011415 | 3291534 | 3291569 | CCCTCGCCCTTTCAGGGAGAGGGCCGGGGTGAGGGT | Escherichia coli SE11 chromosome, complete genome. |
| 181 | NC_011415 | 3291632 | 3291597 | CCCTCACCCCTTTGGGGAGAGGGTTAGGGTGAGGGG | Escherichia coli SE11 chromosome, complete genome. |
| 182 | NC_011415 | 3465928 | 3465963 | CCCTCGCCCCTTCGGGGAGAGGGTTAGGGTGAGGGG | Escherichia coli SE11 chromosome, complete genome. |
| 183 | NC_011415 | 3653589 | 3653554 | CCCTCGCCCCTTTGGGGAGAGGGTTAGGGTGAGGGG | Escherichia coli SE11 chromosome, complete genome. |
| 184 | NC_011415 | 3752687 | 3752652 | CCCTCGCCCCTATGGGGAGAGGGTTAGGGTGAGGGG | Escherichia coli SE11 chromosome, complete genome. |
| 185 | NC_011415 | 3760386 | 3760351 | CCCTCGCCCCTTTGGGGAGAGGGTTAGGGTGAGGGG | Escherichia coli SE11 chromosome, complete genome. |
| 186 | NC_011415 | 3921304 | 3921269 | CCCTCTCCCTTTCAGGGAGAGGGCTGGGGTGAGGGT | Escherichia coli SE11 chromosome, complete genome. |
| 187 | NC_011415 | 3921405 | 3921440 | CCCTCGCCCCTTTGGGGAGAGGGTTAGGGTGAGGGG | Escherichia coli SE11 chromosome, complete genome. |
| 188 | NC_011415 | 4102670 | 4102635 | CCCTCGCCCCTTTGGGGAGAGGGCTAGGGTGAGGGG | Escherichia coli SE11 chromosome, complete genome. |
| 189 | NC_011415 | 4213585 | 4213552 | CCCTCTCCCTGTGGGAGAGGGTCGGGGTGAGGGC | Escherichia coli SE11 chromosome, complete genome. |
| 190 | NC_011415 | 4330638 | 4330673 | CCCTCGCCCCTTTGGGGAGAGGGTTAGGGTGAGGGG | Escherichia coli SE11 chromosome, complete genome. |
| 191 | NC_011415 | 4356069 | 4356034 | CCCTCGCCCCTACGGGGAGAGGGTTAGGGTGAGGGG | Escherichia coli SE11 chromosome, complete genome. |
| 192 | NC_011415 | 4356122 | 4356157 | CCCTCGCCCTTTCAGGGAGAGGGCCGGGGTGAGGGT | Escherichia coli SE11 chromosome, complete genome. |
| 193 | NC_011415 | 4585567 | 4585602 | CCCTCGCCCCTCTGGGGAGAGGGTTAGGGTGAGGGG | Escherichia coli SE11 chromosome, complete genome. |
| 194 | NC_011415 | 4763726 | 4763759 | CCCTCTCCCTTAGGGAGAGGGTTAGGGTGAGGGT | Escherichia coli SE11 chromosome, complete genome. |
| 195 | NC_011415 | 4798844 | 4798809 | CCCTCGCCCCTCCGGGGTGAGGGTTAGGGTGAGGGG | Escherichia coli SE11 chromosome, complete genome. |
| 196 | NC_011415 | 4867867 | 4867834 | CCCTCTCCCTGAGGGAGAGGGTCGGGGTGAGGGC | Escherichia coli SE11 chromosome, complete genome. |
| 197 | NC_011601 | 4844357 | 4844392 | CCCTCGCCCCTTTGGGGAGAGGGTTAGGGTGAGGGG | Escherichia coli O127:H6 str. E2348/69 chromosome, complete genome. |
| 198 | NC_011741 | 67131 | 67096 | CCCTCGCCCCTTTGGGGAGAGGGCCGGGGTGAGGGG | Escherichia coli IAI1 chromosome, complete genome. |
| 199 | NC_011741 | 164790 | 164823 | CCCTCTCCCTGTGGGAGAGGGCCGGGGTGAGGGC | Escherichia coli IAI1 chromosome, complete genome. |
| 200 | NC_011741 | 164874 | 164840 | CCCTCTCCCTTGAGGGAGAGGGTTAGGGTGAGGGT | Escherichia coli IAI1 chromosome, complete genome. |
| 201 | NC_011741 | 401280 | 401315 | CCCTCGCCCCCTTGGGGAGAGGGTTAGGGTGAGGGG | Escherichia coli IAI1 chromosome, complete genome. |
| 202 | NC_011741 | 627412 | 627447 | CCCTCGCCCCTTTGGGGAGAGGGTTAGGGTGAGGGG | Escherichia coli IAI1 chromosome, complete genome. |
| 203 | NC_011741 | 627509 | 627474 | CCCTCTCCCTTCCAGGGTGAGGGCTGGGGTGAGGGT | Escherichia coli IAI1 chromosome, complete genome. |
| 204 | NC_011741 | 1176418 | 1176453 | CCCTCGCCCCTTTGGGGAGAGGGTTAGGGTGAGGGG | Escherichia coli IAI1 chromosome, complete genome. |
| 205 | NC_011741 | 2398328 | 2398363 | CCCTCTCCCCTTGCGGGAGAGGGTAAGGGTGAGGGG | Escherichia coli IAI1 chromosome, complete genome. |
| 206 | NC_011741 | 2686262 | 2686227 | CCCTCGCCCCTTCGGGGAGAGGGTTAGGGTGAGGGG | Escherichia coli IAI1 chromosome, complete genome. |
| 207 | NC_011741 | 2839043 | 2839010 | CCCTCTCCCTTAGGGAGAGGGCCGGGGTGAGGGT | Escherichia coli IAI1 chromosome, complete genome. |
| 208 | NC_011741 | 2867445 | 2867410 | CCCTCGCCCCTTTGGGGAGAGGGTTAGGGTGAGGGG | Escherichia coli IAI1 chromosome, complete genome. |
| 209 | NC_011741 | 3104063 | 3104098 | CCCTCGCCCTTTCAGGGAGAGGGCCGGGGTGAGGGT | Escherichia coli IAI1 chromosome, complete genome. |
| 210 | NC_011741 | 3104161 | 3104126 | CCCTCCCCCCTTTGGGGAGAGGGTTAGGGTGAGGGG | Escherichia coli IAI1 chromosome, complete genome. |
| 211 | NC_011741 | 3284648 | 3284683 | CCCTCGCCCCTTCGGGGAGAGGGTTAGGGTGAGGGG | Escherichia coli IAI1 chromosome, complete genome. |
| 212 | NC_011741 | 3320622 | 3320657 | CCCTCTCCCTTCCAGGGAGAGGGTCGGGGTGAGGGT | Escherichia coli IAI1 chromosome, complete genome. |
| 213 | NC_011741 | 3320721 | 3320685 | CCCTCGCCCCGTTTGGGGAGAGGGTTAGGGTGAGGGG | Escherichia coli IAI1 chromosome, complete genome. |
| 214 | NC_011741 | 3570845 | 3570810 | CCCTCGCCCCTTTGGGGAGAGGGTTAGGGTGAGGGG | Escherichia coli IAI1 chromosome, complete genome. |
| 215 | NC_011741 | 3578544 | 3578509 | CCCTCGCCCCTTTGGGGAGAGGGTTAGGGTGAGGGG | Escherichia coli IAI1 chromosome, complete genome. |
| 216 | NC_011741 | 3740147 | 3740112 | CCCTCTCCCTTTCAGGGAGAGGGCTGGGGTGAGGGT | Escherichia coli IAI1 chromosome, complete genome. |
| 217 | NC_011741 | 3740248 | 3740283 | CCCTCGCCCCTTTGGGGAGAGGGTTAGGGTGAGGGG | Escherichia coli IAI1 chromosome, complete genome. |
| 218 | NC_011741 | 3924941 | 3924906 | CCCTCGCCCCTTTGGGGAGAGGGCTAGGGTGAGGGG | Escherichia coli IAI1 chromosome, complete genome. |
| 219 | NC_011741 | 4035851 | 4035818 | CCCTCTCCCTGTGGGAGAGGGTCGGGGTGAGGGC | Escherichia coli IAI1 chromosome, complete genome. |
| 220 | NC_011741 | 4150212 | 4150247 | CCCTCGCCCCTTTGGGGAGAGGGTTAGGGTGAGGGG | Escherichia coli IAI1 chromosome, complete genome. |
| 221 | NC_011741 | 4175674 | 4175639 | CCCTCGCCCCTACGGGGAGAGGGTTAGGGTGAGGGG | Escherichia coli IAI1 chromosome, complete genome. |
| 222 | NC_011741 | 4175727 | 4175762 | CCCTCGCCCTTTCAGGGAGAGGGCCGGGGTGAGGGT | Escherichia coli IAI1 chromosome, complete genome. |
| 223 | NC_011741 | 4398197 | 4398232 | CCCTCGCCCCTCTGGGGAGAGGGTTAGGGTGAGGGG | Escherichia coli IAI1 chromosome, complete genome. |
| 224 | NC_011741 | 4568675 | 4568708 | CCCTCTCCCTTAGGGAGAGGGTTAGGGTGAGGGT | Escherichia coli IAI1 chromosome, complete genome. |
| 225 | NC_011741 | 4572746 | 4572781 | CCCTCGCCCCTTTGGGGAGAGGGTTAGGGTGAGGGG | Escherichia coli IAI1 chromosome, complete genome. |
| 226 | NC_011741 | 4679581 | 4679548 | CCCTCTCCCTGTGGGAGAGGGCCGGGGTGAGGGC | Escherichia coli IAI1 chromosome, complete genome. |
| 227 | NC_011742 | 551430 | 551465 | CCCTCGCCCCTGTGGGGAGAGGGTTAGGGTGAGGGG | Escherichia coli S88 chromosome, complete genome. |
| 228 | NC_011742 | 4817879 | 4817914 | CCCTCGCCCCTTTGGGGAGAGGGTTAGGGTGAGGGG | Escherichia coli S88 chromosome, complete genome. |
| 229 | NC_011748 | 67144 | 67109 | CCCTCGCCCCTTTGGGGAGAGGGCCGGGGTGAGGGG | Escherichia coli 55989 chromosome, complete genome. |
| 230 | NC_011748 | 164648 | 164681 | CCCTCTCCCTGTGGGAGAGGGCCGGGGTGAGGGC | Escherichia coli 55989 chromosome, complete genome. |
| 231 | NC_011748 | 164732 | 164698 | CCCTCTCCCTTGAGGGAGAGGGTTAGGGTGAGGGT | Escherichia coli 55989 chromosome, complete genome. |
| 232 | NC_011748 | 414383 | 414418 | CCCTCGCCCCCTTGGGGAGAGGGTTAGGGTGAGGGG | Escherichia coli 55989 chromosome, complete genome. |
| 233 | NC_011748 | 525154 | 525189 | CCCTCGCCCCCTCGGGGAGAGGGTTAGGGTGAGGGG | Escherichia coli 55989 chromosome, complete genome. |
| 234 | NC_011748 | 640142 | 640177 | CCCTCGCCCCTTTGGGGAGAGGGTTAGGGTGAGGGG | Escherichia coli 55989 chromosome, complete genome. |
| 235 | NC_011748 | 640239 | 640204 | CCCTCTCCCTTCCAGGGTGAGGGCTGGGGTGAGGGT | Escherichia coli 55989 chromosome, complete genome. |
| 236 | NC_011748 | 2553406 | 2553441 | CCCTCTCCCCTTGCGGGAGAGGGTTAGGGTGAGGGG | Escherichia coli 55989 chromosome, complete genome. |
| 237 | NC_011748 | 3004823 | 3004790 | CCCTCTCCCTTAGGGAGAGGGCCGGGGTGAGGGT | Escherichia coli 55989 chromosome, complete genome. |
| 238 | NC_011748 | 3033667 | 3033632 | CCCTCGCCCCTTTGGGGAGAGGGTTAGGGTGAGGGG | Escherichia coli 55989 chromosome, complete genome. |
| 239 | NC_011748 | 3271109 | 3271144 | CCCTCGCCCTTTCAGGGAGAGGGCCGGGGTGAGGGT | Escherichia coli 55989 chromosome, complete genome. |
| 240 | NC_011748 | 3551911 | 3551946 | CCCTCGCCCCTTCGGGGAGAGGGTTAGGGTGAGGGG | Escherichia coli 55989 chromosome, complete genome. |
| 241 | NC_011748 | 3587888 | 3587923 | CCCTCTCCCTTCCAGGGAGAGGGTCGGGGTGAGGGT | Escherichia coli 55989 chromosome, complete genome. |
| 242 | NC_011748 | 3587987 | 3587951 | CCCTCGCCCCGTTTGGGGAGAGGGTTAGGGTGAGGGG | Escherichia coli 55989 chromosome, complete genome. |
| 243 | NC_011748 | 3839425 | 3839390 | CCCTCGCCCCTTTGGGGAGAGGGTTAGGGTGAGGGG | Escherichia coli 55989 chromosome, complete genome. |
| 244 | NC_011748 | 3847124 | 3847089 | CCCTCGCCCCTTTGGGGAGAGGGTTAGGGTGAGGGG | Escherichia coli 55989 chromosome, complete genome. |
| 245 | NC_011748 | 4041270 | 4041235 | CCCTCTCCCTTTCAGGGAGAGGGCTGGGGTGAGGGT | Escherichia coli 55989 chromosome, complete genome. |
| 246 | NC_011748 | 4041371 | 4041406 | CCCTCGCCCCTTTGGGGAGAGGGTTAGGGTGAGGGG | Escherichia coli 55989 chromosome, complete genome. |
| 247 | NC_011748 | 4228816 | 4228781 | CCCTCGCCCCTTTGGGGAGAGGGCTAGGGTGAGGGG | Escherichia coli 55989 chromosome, complete genome. |
| 248 | NC_011748 | 4339786 | 4339753 | CCCTCTCCCTGTGGGAGAGGGTCGGGGTGAGGGC | Escherichia coli 55989 chromosome, complete genome. |
| 249 | NC_011748 | 4476324 | 4476289 | CCCTCGCCCCTACGGGGAGAGGGTTAGGGTGAGGGG | Escherichia coli 55989 chromosome, complete genome. |
| 250 | NC_011748 | 4476377 | 4476412 | CCCTCGCCCTTTCAGGGAGAGGGCCGGGGTGAGGGT | Escherichia coli 55989 chromosome, complete genome. |
| 251 | NC_011748 | 4698973 | 4699008 | CCCTCGCCCCTCTGGGGAGAGGGTTAGGGTGAGGGG | Escherichia coli 55989 chromosome, complete genome. |
| 252 | NC_011748 | 4702663 | 4702628 | CCCTCGCCCCTTCGGGGAGAGGGTTAGGGTGAGGGG | Escherichia coli 55989 chromosome, complete genome. |
| 253 | NC_011748 | 4702704 | 4702737 | CCCTCTCCCTCCGGGAGAGGGCCGGGGTGAGGGG | Escherichia coli 55989 chromosome, complete genome. |
| 254 | NC_011748 | 4897791 | 4897826 | CCCTCGCCCCTTCGGGGAGAGGGTTAGGGTGAGGGG | Escherichia coli 55989 chromosome, complete genome. |
| 255 | NC_011748 | 4932212 | 4932245 | CCCTCTCCCTTAGGGAGAGGGTTAGGGTGAGGGT | Escherichia coli 55989 chromosome, complete genome. |
| 256 | NC_011748 | 4936283 | 4936318 | CCCTCGCCCCTTTGGGGAGAGGGTTAGGGTGAGGGG | Escherichia coli 55989 chromosome, complete genome. |
| 257 | NC_011748 | 5134004 | 5133971 | CCCTCTCCCTGAGGGAGAGGGCCGGGGTGAGGGC | Escherichia coli 55989 chromosome, complete genome. |
| 258 | NC_011750 | 82649 | 82614 | CCCTCGCCCCTTCGGGGAGAGGGTTAGGGTGAGGGG | Escherichia coli IAI39 chromosome, complete genome. |
| 259 | NC_011750 | 665251 | 665284 | CCCTCGCCCCTCGGGTGAGGGTTAGGGTGAGGGG | Escherichia coli IAI39 chromosome, complete genome. |
| 260 | NC_011750 | 4726243 | 4726208 | CCCTCGCCCCTTCGGGGAGAGGGTTAGGGTGAGGGG | Escherichia coli IAI39 chromosome, complete genome. |
| 261 | NC_011750 | 4726283 | 4726318 | CCCTCTCCCCTCTGGGGAGAGGGCCGGGGTGAGGGG | Escherichia coli IAI39 chromosome, complete genome. |
| 262 | NC_011750 | 4942649 | 4942684 | CCCTCGCCCCTTTGGGGAGAGGGTTAGGGTGAGGGG | Escherichia coli IAI39 chromosome, complete genome. |
| 263 | NC_011750 | 4997343 | 4997308 | CCCTCGCCCCTCCGGGGAGAGGGTTAGGGTGAGGGG | Escherichia coli IAI39 chromosome, complete genome. |
| 264 | NC_011993 | 604718 | 604683 | CCCTCGCCCCACCGGGGAGAGGGTTAGGGTGAGGGG | Escherichia coli LF82, complete genome. |
| 265 | NC_011993 | 2553920 | 2553885 | CCCTCGCCCCTTTGGGGAGAGGGTTAGGGTGAGGGG | Escherichia coli LF82, complete genome. |
| 266 | NC_012759 | 164546 | 164579 | CCCTCTCCCTGTGGGAGAGGGCCGGGGTGAGGGC | Escherichia coli BW2952 chromosome, complete genome. |
| 267 | NC_012759 | 164630 | 164596 | CCCTCTCCCTTGAGGGAGAGGGTTAGGGTGAGGGT | Escherichia coli BW2952 chromosome, complete genome. |
| 268 | NC_012759 | 290647 | 290682 | CCCTCGCCCCCTTGGGGAGAGGGTTAGGGTGAGGGG | Escherichia coli BW2952 chromosome, complete genome. |
| 269 | NC_012759 | 399826 | 399861 | CCCTCGCCCCCTCGGGGAGAGGGTTAGGGTGAGGGG | Escherichia coli BW2952 chromosome, complete genome. |
| 270 | NC_012759 | 526562 | 526597 | CCCTCGCCCCTTTGGGGAGAGGGTTAGGGTGAGGGG | Escherichia coli BW2952 chromosome, complete genome. |
| 271 | NC_012759 | 526659 | 526624 | CCCTCTCCCTTCCAGGGTGAGGGCTGGGGTGAGGGT | Escherichia coli BW2952 chromosome, complete genome. |
| 272 | NC_012759 | 1240112 | 1240077 | CCCTCGCCCCTCTGGGGAGAGGGTTAGGGTGAGGGG | Escherichia coli BW2952 chromosome, complete genome. |
| 273 | NC_012759 | 2931159 | 2931194 | CCCTCGCCCTTTCAGGGAGAGGGCCGGGGTGAGGGT | Escherichia coli BW2952 chromosome, complete genome. |
| 274 | NC_012759 | 2931257 | 2931222 | CCCTCGCCCCTTTGGGGAGAGGGTTAGGGTGAGGGG | Escherichia coli BW2952 chromosome, complete genome. |
| 275 | NC_012759 | 3124769 | 3124804 | CCCTCTCCCTTCCAGGGAGAGGGTCGGGGTGAGGGT | Escherichia coli BW2952 chromosome, complete genome. |
| 276 | NC_012759 | 3124868 | 3124832 | CCCTCGCCCCGTTTGGGGAGAGGGTTAGGGTGAGGGG | Escherichia coli BW2952 chromosome, complete genome. |
| 277 | NC_012759 | 3275699 | 3275664 | CCCTCGCCCCTTTGGGGTGAGGGTTAGGGTGAGGGG | Escherichia coli BW2952 chromosome, complete genome. |
| 278 | NC_012759 | 3390071 | 3390036 | CCCTCGCCCCTTTGGGGAGAGGGTTAGGGTGAGGGG | Escherichia coli BW2952 chromosome, complete genome. |
| 279 | NC_012759 | 3495201 | 3495236 | CCCTCGCCCCTCTGGGGAGAGGGTTAGGGTGAGGGG | Escherichia coli BW2952 chromosome, complete genome. |
| 280 | NC_012759 | 3667410 | 3667377 | CCCTCTCCCTGAGGGAGAGGGTTAGGGTGAGGGG | Escherichia coli BW2952 chromosome, complete genome. |
| 281 | NC_012759 | 3667470 | 3667505 | CCCTCGCCCCTCCGGGGAGAGGGCCGGGGTGAGGGG | Escherichia coli BW2952 chromosome, complete genome. |
| 282 | NC_012759 | 3794851 | 3794886 | CCCTCGCCCCTCTGGGGAGAGGGTTAGGGTGAGGGG | Escherichia coli BW2952 chromosome, complete genome. |
| 283 | NC_012759 | 3847183 | 3847150 | CCCTCTCCCTGTGGGAGAGGGTCGGGGTGAGGGC | Escherichia coli BW2952 chromosome, complete genome. |
| 284 | NC_012759 | 3958144 | 3958179 | CCCTCGCCCCTTTGGGGAGAGGGTTAGGGAGAGGGG | Escherichia coli BW2952 chromosome, complete genome. |
| 285 | NC_012759 | 4251043 | 4251078 | CCCTCGCCCCTCTGGGGAGAGGGTTAGGGTGAGGGG | Escherichia coli BW2952 chromosome, complete genome. |
| 286 | NC_012759 | 4486391 | 4486356 | CCCTCGCCCCTCCGGGGAGAGGGTTAGGGTGAGGGG | Escherichia coli BW2952 chromosome, complete genome. |
| 287 | NC_012947 | 301804 | 301769 | CCCTCGCCCCTCTGGGGAGAGGGTTAGGGTGAGGGG | Escherichia coli 'BL21-Gold(DE3)pLysS AG' chromosome, complete genome. |
| 288 | NC_012947 | 409311 | 409346 | CCCTCGCCCCTTTGGGGAGAGGGTTAGGGTGAGGGG | Escherichia coli 'BL21-Gold(DE3)pLysS AG' chromosome, complete genome. |
| 289 | NC_012947 | 704545 | 704510 | CCCTCGCCCCTTCGGGGAGAGGGTTAGGGTGAGGGG | Escherichia coli 'BL21-Gold(DE3)pLysS AG' chromosome, complete genome. |
| 290 | NC_012947 | 862958 | 862993 | CCCTCACCCCTTTGGGGAGAGGGTTAGGGTGAGGGG | Escherichia coli 'BL21-Gold(DE3)pLysS AG' chromosome, complete genome. |
| 291 | NC_012947 | 863056 | 863021 | CCCTCGCCCTTTCAGGGAGAGGGCCGGGGTGAGGGT | Escherichia coli 'BL21-Gold(DE3)pLysS AG' chromosome, complete genome. |
| 292 | NC_012947 | 1105994 | 1105959 | CCCTCGCCCCTCTGGGGAGAGGGTTAGGGTGAGGGG | Escherichia coli 'BL21-Gold(DE3)pLysS AG' chromosome, complete genome. |
| 293 | NC_012947 | 1722277 | 1722243 | CCCTCGCCCCCTGGGGAGAGGGTTAGGGTGAGGGG | Escherichia coli 'BL21-Gold(DE3)pLysS AG' chromosome, complete genome. |
| 294 | NC_012947 | 2444776 | 2444811 | CCCTCGCCCCTCTGGGGAGAGGGTTAGGGTGAGGGG | Escherichia coli 'BL21-Gold(DE3)pLysS AG' chromosome, complete genome. |
| 295 | NC_012947 | 3189821 | 3189856 | CCCTCGCCCCATCGGGGAGAGGGTTAGGGTGAGGGG | Escherichia coli 'BL21-Gold(DE3)pLysS AG' chromosome, complete genome. |
| 296 | NC_012947 | 3197221 | 3197256 | CCCTCTCCCTTCCAGGGTGAGGGCTGGGGTGAGGGT | Escherichia coli 'BL21-Gold(DE3)pLysS AG' chromosome, complete genome. |
| 297 | NC_012947 | 3197318 | 3197283 | CCCTCGCCCCTTTGGGGAGAGGGTTAGGGTGAGGGG | Escherichia coli 'BL21-Gold(DE3)pLysS AG' chromosome, complete genome. |
| 298 | NC_012947 | 3315495 | 3315460 | CCCTCGCCCCCTCGGGGAGAGGGTTAGGGTGAGGGG | Escherichia coli 'BL21-Gold(DE3)pLysS AG' chromosome, complete genome. |
| 299 | NC_012947 | 3427833 | 3427798 | CCCTCGCCCCCTTGGGGAGAGGGTTAGGGTGAGGGG | Escherichia coli 'BL21-Gold(DE3)pLysS AG' chromosome, complete genome. |
| 300 | NC_012947 | 3616239 | 3616273 | CCCTCTCCCTTGAGGGAGAGGGTTAGGGTGAGGGT | Escherichia coli 'BL21-Gold(DE3)pLysS AG' chromosome, complete genome. |
| 301 | NC_012947 | 3616323 | 3616290 | CCCTCTCCCTGTGGGAGAGGGCCGGGGTGAGGGC | Escherichia coli 'BL21-Gold(DE3)pLysS AG' chromosome, complete genome. |
| 302 | NC_012947 | 3803422 | 3803455 | CCCTCTCCCTGTGGGAGAGGGCCGGGGTGAGGGC | Escherichia coli 'BL21-Gold(DE3)pLysS AG' chromosome, complete genome. |
| 303 | NC_012947 | 3883311 | 3883346 | CCCTCGCCCCTCCGGGGAGAGGGTTAGGGTGAGGGG | Escherichia coli 'BL21-Gold(DE3)pLysS AG' chromosome, complete genome. |
| 304 | NC_012947 | 4121001 | 4120966 | CCCTCGCCCCTCTGGGGAGAGGGTTAGGGTGAGGGG | Escherichia coli 'BL21-Gold(DE3)pLysS AG' chromosome, complete genome. |
| 305 | NC_012947 | 4365433 | 4365398 | CCCTCGCCCCTTTGGGGAGAGGGTTAGGGTGAGGGG | Escherichia coli 'BL21-Gold(DE3)pLysS AG' chromosome, complete genome. |
| 306 | NC_012947 | 4494077 | 4494110 | CCCTCTCCCTGTGGGAGAGGGTCGGGGTGAGGGC | Escherichia coli 'BL21-Gold(DE3)pLysS AG' chromosome, complete genome. |
| 307 | NC_012947 | 4546513 | 4546478 | CCCTCGCCCCTCTGGGGAGAGGGTTAGGGTGAGGGG | Escherichia coli 'BL21-Gold(DE3)pLysS AG' chromosome, complete genome. |
| 308 | NC_012967 | 167398 | 167431 | CCCTCTCCCTGTGGGAGAGGGCCGGGGTGAGGGC | Escherichia coli B str. REL606 chromosome, complete genome. |
| 309 | NC_012967 | 167482 | 167448 | CCCTCTCCCTTGAGGGAGAGGGTTAGGGTGAGGGT | Escherichia coli B str. REL606 chromosome, complete genome. |
| 310 | NC_012967 | 357572 | 357607 | CCCTCGCCCCCTTGGGGAGAGGGTTAGGGTGAGGGG | Escherichia coli B str. REL606 chromosome, complete genome. |
| 311 | NC_012967 | 469910 | 469945 | CCCTCGCCCCCTCGGGGAGAGGGTTAGGGTGAGGGG | Escherichia coli B str. REL606 chromosome, complete genome. |
| 312 | NC_012967 | 607287 | 607322 | CCCTCGCCCCTTTGGGGAGAGGGTTAGGGTGAGGGG | Escherichia coli B str. REL606 chromosome, complete genome. |
| 313 | NC_012967 | 607384 | 607349 | CCCTCTCCCTTCCAGGGTGAGGGCTGGGGTGAGGGT | Escherichia coli B str. REL606 chromosome, complete genome. |
| 314 | NC_012967 | 614784 | 614749 | CCCTCGCCCCATCGGGGAGAGGGTTAGGGTGAGGGG | Escherichia coli B str. REL606 chromosome, complete genome. |
| 315 | NC_012967 | 1349608 | 1349573 | CCCTCGCCCCTCTGGGGAGAGGGTTAGGGTGAGGGG | Escherichia coli B str. REL606 chromosome, complete genome. |
| 316 | NC_012967 | 2051730 | 2051764 | CCCTCGCCCCCTGGGGAGAGGGTTAGGGTGAGGGG | Escherichia coli B str. REL606 chromosome, complete genome. |
| 317 | NC_012967 | 2688803 | 2688838 | CCCTCGCCCCTCTGGGGAGAGGGTTAGGGTGAGGGG | Escherichia coli B str. REL606 chromosome, complete genome. |
| 318 | NC_012967 | 2931741 | 2931776 | CCCTCGCCCTTTCAGGGAGAGGGCCGGGGTGAGGGT | Escherichia coli B str. REL606 chromosome, complete genome. |
| 319 | NC_012967 | 2931839 | 2931804 | CCCTCACCCCTTTGGGGAGAGGGTTAGGGTGAGGGG | Escherichia coli B str. REL606 chromosome, complete genome. |
| 320 | NC_012967 | 3135994 | 3136029 | CCCTCGCCCCTTCGGGGAGAGGGTTAGGGTGAGGGG | Escherichia coli B str. REL606 chromosome, complete genome. |
| 321 | NC_012967 | 3432797 | 3432762 | CCCTCGCCCCTTTGGGGAGAGGGTTAGGGTGAGGGG | Escherichia coli B str. REL606 chromosome, complete genome. |
| 322 | NC_012967 | 3868862 | 3868897 | CCCTCGCCCCTCTGGGGAGAGGGTTAGGGTGAGGGG | Escherichia coli B str. REL606 chromosome, complete genome. |
| 323 | NC_012967 | 3921293 | 3921260 | CCCTCTCCCTGTGGGAGAGGGTCGGGGTGAGGGC | Escherichia coli B str. REL606 chromosome, complete genome. |
| 324 | NC_012967 | 4048599 | 4048634 | CCCTCGCCCCTTTGGGGAGAGGGTTAGGGTGAGGGG | Escherichia coli B str. REL606 chromosome, complete genome. |
| 325 | NC_012967 | 4293107 | 4293142 | CCCTCGCCCCTCTGGGGAGAGGGTTAGGGTGAGGGG | Escherichia coli B str. REL606 chromosome, complete genome. |
| 326 | NC_012967 | 4530797 | 4530762 | CCCTCGCCCCTCCGGGGAGAGGGTTAGGGTGAGGGG | Escherichia coli B str. REL606 chromosome, complete genome. |
| 327 | NC_012967 | 4610112 | 4610079 | CCCTCTCCCTGTGGGAGAGGGCCGGGGTGAGGGC | Escherichia coli B str. REL606 chromosome, complete genome. |
| 328 | NC_012971 | 167399 | 167432 | CCCTCTCCCTGTGGGAGAGGGCCGGGGTGAGGGC | Escherichia coli BL21(DE3) chromosome, complete genome. |
| 329 | NC_012971 | 167483 | 167449 | CCCTCTCCCTTGAGGGAGAGGGTTAGGGTGAGGGT | Escherichia coli BL21(DE3) chromosome, complete genome. |
| 330 | NC_012971 | 354551 | 354586 | CCCTCGCCCCCTTGGGGAGAGGGTTAGGGTGAGGGG | Escherichia coli BL21(DE3) chromosome, complete genome. |
| 331 | NC_012971 | 466889 | 466924 | CCCTCGCCCCCTCGGGGAGAGGGTTAGGGTGAGGGG | Escherichia coli BL21(DE3) chromosome, complete genome. |
| 332 | NC_012971 | 585066 | 585101 | CCCTCGCCCCTTTGGGGAGAGGGTTAGGGTGAGGGG | Escherichia coli BL21(DE3) chromosome, complete genome. |
| 333 | NC_012971 | 585163 | 585128 | CCCTCTCCCTTCCAGGGTGAGGGCTGGGGTGAGGGT | Escherichia coli BL21(DE3) chromosome, complete genome. |
| 334 | NC_012971 | 592563 | 592528 | CCCTCGCCCCATCGGGGAGAGGGTTAGGGTGAGGGG | Escherichia coli BL21(DE3) chromosome, complete genome. |
| 335 | NC_012971 | 1337606 | 1337571 | CCCTCGCCCCTCTGGGGAGAGGGTTAGGGTGAGGGG | Escherichia coli BL21(DE3) chromosome, complete genome. |
| 336 | NC_012971 | 2017531 | 2017565 | CCCTCGCCCCCTGGGGAGAGGGTTAGGGTGAGGGG | Escherichia coli BL21(DE3) chromosome, complete genome. |
| 337 | NC_012971 | 2633910 | 2633945 | CCCTCGCCCCTCTGGGGAGAGGGTTAGGGTGAGGGG | Escherichia coli BL21(DE3) chromosome, complete genome. |
| 338 | NC_012971 | 2876848 | 2876883 | CCCTCGCCCTTTCAGGGAGAGGGCCGGGGTGAGGGT | Escherichia coli BL21(DE3) chromosome, complete genome. |
| 339 | NC_012971 | 2876946 | 2876911 | CCCTCACCCCTTTGGGGAGAGGGTTAGGGTGAGGGG | Escherichia coli BL21(DE3) chromosome, complete genome. |
| 340 | NC_012971 | 3069259 | 3069294 | CCCTCGCCCCTTCGGGGAGAGGGTTAGGGTGAGGGG | Escherichia coli BL21(DE3) chromosome, complete genome. |
| 341 | NC_012971 | 3364493 | 3364458 | CCCTCGCCCCTTTGGGGAGAGGGTTAGGGTGAGGGG | Escherichia coli BL21(DE3) chromosome, complete genome. |
| 342 | NC_012971 | 3797297 | 3797332 | CCCTCGCCCCTCTGGGGAGAGGGTTAGGGTGAGGGG | Escherichia coli BL21(DE3) chromosome, complete genome. |
| 343 | NC_012971 | 3849733 | 3849700 | CCCTCTCCCTGTGGGAGAGGGTCGGGGTGAGGGC | Escherichia coli BL21(DE3) chromosome, complete genome. |
| 344 | NC_012971 | 3977039 | 3977074 | CCCTCGCCCCTTTGGGGAGAGGGTTAGGGTGAGGGG | Escherichia coli BL21(DE3) chromosome, complete genome. |
| 345 | NC_012971 | 4221472 | 4221507 | CCCTCGCCCCTCTGGGGAGAGGGTTAGGGTGAGGGG | Escherichia coli BL21(DE3) chromosome, complete genome. |
| 346 | NC_012971 | 4459162 | 4459127 | CCCTCGCCCCTCCGGGGAGAGGGTTAGGGTGAGGGG | Escherichia coli BL21(DE3) chromosome, complete genome. |
| 347 | NC_012971 | 4539253 | 4539220 | CCCTCTCCCTGTGGGAGAGGGCCGGGGTGAGGGC | Escherichia coli BL21(DE3) chromosome, complete genome. |
| 348 | NC_013008 | 168850 | 168883 | CCCTCTCCCTGTGGGAGAGGGCCGGGGTGAGGGC | Escherichia coli O157:H7 str. TW14359 chromosome, complete genome. |
| 349 | NC_013008 | 168934 | 168900 | CCCTCTCCCTTAAGGGAGAGGGTTAGGGTGAGGGT | Escherichia coli O157:H7 str. TW14359 chromosome, complete genome. |
| 350 | NC_013008 | 718424 | 718389 | CCCTCGCCCCATCGGGGAGAGGGTTAGGGTGAGGGG | Escherichia coli O157:H7 str. TW14359 chromosome, complete genome. |
| 351 | NC_013008 | 1437614 | 1437649 | CCCTCGCCCCTTTGGGGAGAGGGTTAGGGTGAGGGG | Escherichia coli O157:H7 str. TW14359 chromosome, complete genome. |
| 352 | NC_013008 | 2955675 | 2955641 | CCCTCGCCCCTTCGGGAGAGGGTTAGGGTGAGGGT | Escherichia coli O157:H7 str. TW14359 chromosome, complete genome. |
| 353 | NC_013008 | 3442044 | 3442009 | CCCTCGCCCCTTCGGGGAGAGGGTTAGGGTGAGGGG | Escherichia coli O157:H7 str. TW14359 chromosome, complete genome. |
| 354 | NC_013008 | 4038355 | 4038390 | CCCTCTCCCTTCCAGGGAGAGGGTCGGGGTGAGGGT | Escherichia coli O157:H7 str. TW14359 chromosome, complete genome. |
| 355 | NC_013008 | 4038454 | 4038418 | CCCTCGCCCCGTTTGGGGAGAGGGTTAGGGTGAGGGG | Escherichia coli O157:H7 str. TW14359 chromosome, complete genome. |
| 356 | NC_013008 | 4183571 | 4183536 | CCCTCGCCCCTTTGGGGAGAGGGTTAGGGTGAGGGG | Escherichia coli O157:H7 str. TW14359 chromosome, complete genome. |
| 357 | NC_013008 | 4283929 | 4283894 | CCCTCGCCCCTTTGGGGAGAGGGTTAGGGTGAGGGG | Escherichia coli O157:H7 str. TW14359 chromosome, complete genome. |
| 358 | NC_013008 | 4754341 | 4754376 | CCCTCGCCCCTCTGGGGAGAGGGTTAGGGTGAGGGG | Escherichia coli O157:H7 str. TW14359 chromosome, complete genome. |
| 359 | NC_013353 | 165945 | 165978 | CCCTCTCCCTGTGGGAGAGGGCCGGGGTGAGGGC | Escherichia coli O103:H2 str. 12009, complete genome. |
| 360 | NC_013353 | 166029 | 165995 | CCCTCTCCCTTGAGGGAGAGGGTTAGGGTGAGGGT | Escherichia coli O103:H2 str. 12009, complete genome. |
| 361 | NC_013353 | 396274 | 396309 | CCCTCGCCCCCTTGGGGAGAGGGTTAGGGTGAGGGG | Escherichia coli O103:H2 str. 12009, complete genome. |
| 362 | NC_013353 | 506432 | 506467 | CCCTCGCCCCCTCGGGGAGAGGGTTAGGGTGAGGGG | Escherichia coli O103:H2 str. 12009, complete genome. |
| 363 | NC_013353 | 662371 | 662406 | CCCTCGCCCCTTTGGGGAGAGGGTTAGGGTGAGGGG | Escherichia coli O103:H2 str. 12009, complete genome. |
| 364 | NC_013353 | 662468 | 662433 | CCCTCTCCCTTCCAGGGTGAGGGCTGGGGTGAGGGT | Escherichia coli O103:H2 str. 12009, complete genome. |
| 365 | NC_013353 | 669878 | 669843 | CCCTCGCCCCATCGGGGAGAGGGTTAGGGTGAGGGG | Escherichia coli O103:H2 str. 12009, complete genome. |
| 366 | NC_013353 | 1219342 | 1219377 | CCCTCGCCCCTTTGGGGAGAGGGTTAGGGTGAGGGG | Escherichia coli O103:H2 str. 12009, complete genome. |
| 367 | NC_013353 | 3285469 | 3285434 | CCCTCGCCCCTTTGGGGAGAGGGTTAGGGTGAGGGG | Escherichia coli O103:H2 str. 12009, complete genome. |
| 368 | NC_013353 | 3554582 | 3554617 | CCCTCGCCCTTTCAGGGAGAGGGCCGGGGTGAGGGT | Escherichia coli O103:H2 str. 12009, complete genome. |
| 369 | NC_013353 | 3814220 | 3814255 | CCCTCGCCCCTTCGGGGAGAGGGTTAGGGTGAGGGG | Escherichia coli O103:H2 str. 12009, complete genome. |
| 370 | NC_013353 | 3914792 | 3914827 | CCCTCTCCCTTCCAGGGAGAGGGTCGGGGTGAGGGT | Escherichia coli O103:H2 str. 12009, complete genome. |
| 371 | NC_013353 | 3914891 | 3914855 | CCCTCGCCCCGTTTGGGGAGAGGGTTAGGGTGAGGGG | Escherichia coli O103:H2 str. 12009, complete genome. |
| 372 | NC_013353 | 4174258 | 4174223 | CCCTCGCCCCTTTGGGGAGAGGGTTAGGGTGAGGGG | Escherichia coli O103:H2 str. 12009, complete genome. |
| 373 | NC_013353 | 4343372 | 4343337 | CCCTCTCCCTTTCAGGGAGAGGGCTGGGGTGAGGGT | Escherichia coli O103:H2 str. 12009, complete genome. |
| 374 | NC_013353 | 4343473 | 4343508 | CCCTCGCCCCTTTGGGGAGAGGGTTAGGGTGAGGGG | Escherichia coli O103:H2 str. 12009, complete genome. |
| 375 | NC_013353 | 4389367 | 4389332 | CCCTCGCCCCTTTGGGGAGAGGGTTAGGGTGAGGGG | Escherichia coli O103:H2 str. 12009, complete genome. |
| 376 | NC_013353 | 4503566 | 4503599 | CCCTCTCCCTGTGGGAGAGGGTCGGGGTGAGGGC | Escherichia coli O103:H2 str. 12009, complete genome. |
| 377 | NC_013353 | 4610318 | 4610353 | CCCTCGCCCCTTTGGGGAGAGGGCTAGGGTGAGGGG | Escherichia coli O103:H2 str. 12009, complete genome. |
| 378 | NC_013353 | 4754789 | 4754754 | CCCTCGCCCTTTCAGGGAGAGGGCCGGGGTGAGGGT | Escherichia coli O103:H2 str. 12009, complete genome. |
| 379 | NC_013353 | 5025374 | 5025409 | CCCTCGCCCCTCTGGGGAGAGGGTTAGGGTGAGGGG | Escherichia coli O103:H2 str. 12009, complete genome. |
| 380 | NC_013353 | 5249621 | 5249654 | CCCTCTCCCTTAGGGAGAGGGTTAGGGTGAGGGT | Escherichia coli O103:H2 str. 12009, complete genome. |
| 381 | NC_013353 | 5253692 | 5253727 | CCCTCGCCCCTTTGGGGAGAGGGTTAGGGTGAGGGG | Escherichia coli O103:H2 str. 12009, complete genome. |
| 382 | NC_013353 | 5304759 | 5304724 | CCCTCGCCCCTCCGGGGAGAGGGTTAGGGTGAGGGG | Escherichia coli O103:H2 str. 12009, complete genome. |
| 383 | NC_013353 | 5429614 | 5429581 | CCCTCTCCCTGAGGGAGAGGGCCGGGGTGAGGGC | Escherichia coli O103:H2 str. 12009, complete genome. |
| 384 | NC_013361 | 68495 | 68460 | CCCTCGCCCCTTTGGGGAGAGGGCCGGGGTGAGGGG | Escherichia coli O26:H11 str. 11368 chromosome, complete genome. |
| 385 | NC_013361 | 288479 | 288446 | CCCTCTCCCTGTGGGAGAGGGTCGGGGTGAGGGC | Escherichia coli O26:H11 str. 11368 chromosome, complete genome. |
| 386 | NC_013361 | 288520 | 288555 | CCCTCGCCCCTTTGGGGAGAGGGTTAGGGTGAGGGG | Escherichia coli O26:H11 str. 11368 chromosome, complete genome. |
| 387 | NC_013361 | 433953 | 433988 | CCCTCGCCCCCTTGGGGAGAGGGTTAGGGTGAGGGG | Escherichia coli O26:H11 str. 11368 chromosome, complete genome. |
| 388 | NC_013361 | 546651 | 546686 | CCCTCGCCCCCTCGGGGAGAGGGTTAGGGTGAGGGG | Escherichia coli O26:H11 str. 11368 chromosome, complete genome. |
| 389 | NC_013361 | 710708 | 710673 | CCCTCTCCCTTCCAGGGTGAGGGCTGGGGTGAGGGT | Escherichia coli O26:H11 str. 11368 chromosome, complete genome. |
| 390 | NC_013361 | 1423778 | 1423813 | CCCTCGCCCCTTTGGGGAGAGGGTTAGGGTGAGGGG | Escherichia coli O26:H11 str. 11368 chromosome, complete genome. |
| 391 | NC_013361 | 2875141 | 2875106 | CCCTCGCCCCTTTGGGGAGAGGGCCGGGGTGAGGGG | Escherichia coli O26:H11 str. 11368 chromosome, complete genome. |
| 392 | NC_013361 | 3151646 | 3151681 | CCCTCTCCCCTTGCGGGAGAGGGTTAGGGTGAGGGG | Escherichia coli O26:H11 str. 11368 chromosome, complete genome. |
| 393 | NC_013361 | 3511609 | 3511574 | CCCTCGCCCCTTCGGGGAGAGGGTTAGGGTGAGGGG | Escherichia coli O26:H11 str. 11368 chromosome, complete genome. |
| 394 | NC_013361 | 3672574 | 3672541 | CCCTCTCCCTTAGGGAGAGGGCCGGGGTGAGGGT | Escherichia coli O26:H11 str. 11368 chromosome, complete genome. |
| 395 | NC_013361 | 3701151 | 3701116 | CCCTCGCCCCTTTGGGGAGAGGGTTAGGGGGAGGGG | Escherichia coli O26:H11 str. 11368 chromosome, complete genome. |
| 396 | NC_013361 | 3950381 | 3950416 | CCCTCGCCCTTTCAGGGAGAGGGCCGGGGTGAGGGT | Escherichia coli O26:H11 str. 11368 chromosome, complete genome. |
| 397 | NC_013361 | 4124972 | 4125007 | CCCTCGCCCCTTCGGGGAGAGGGTTAGGGTGAGGGG | Escherichia coli O26:H11 str. 11368 chromosome, complete genome. |
| 398 | NC_013361 | 4160945 | 4160980 | CCCTCTCCCTTCCAGGGAGAGGGTCGGGGTGAGGGT | Escherichia coli O26:H11 str. 11368 chromosome, complete genome. |
| 399 | NC_013361 | 4161044 | 4161008 | CCCTCGCCCCGTTTGGGGAGAGGGTTAGGGTGAGGGG | Escherichia coli O26:H11 str. 11368 chromosome, complete genome. |
| 400 | NC_013361 | 4422993 | 4422958 | CCCTCGCCCCTTTGGGGAGAGGGTTAGGGTGAGGGG | Escherichia coli O26:H11 str. 11368 chromosome, complete genome. |
| 401 | NC_013361 | 4430692 | 4430657 | CCCTCGCCCCTTTGGGGAGAGGGTTAGGGTGAGGGG | Escherichia coli O26:H11 str. 11368 chromosome, complete genome. |
| 402 | NC_013361 | 4529160 | 4529195 | CCCTCGCCCCTCTGGGGAGAGGGTTAGGGTGAGGGG | Escherichia coli O26:H11 str. 11368 chromosome, complete genome. |
| 403 | NC_013361 | 4596931 | 4596896 | CCCTCTCCCTTTCAGGGAGAGGGCTGGGGTGAGGGT | Escherichia coli O26:H11 str. 11368 chromosome, complete genome. |
| 404 | NC_013361 | 4597032 | 4597067 | CCCTCGCCCCTTTGGGGAGAGGGTTAGGGTGAGGGG | Escherichia coli O26:H11 str. 11368 chromosome, complete genome. |
| 405 | NC_013361 | 4708379 | 4708344 | CCCTCGCCCCTTTGGGGAGAGGGTTAGGGTGAGGGG | Escherichia coli O26:H11 str. 11368 chromosome, complete genome. |
| 406 | NC_013361 | 4824010 | 4824043 | CCCTCTCCCTGTGGGAGAGGGTCGGGGTGAGGGC | Escherichia coli O26:H11 str. 11368 chromosome, complete genome. |
| 407 | NC_013361 | 4934980 | 4935015 | CCCTCGCCCCTTTGGGGAGAGGGCTAGGGTGAGGGG | Escherichia coli O26:H11 str. 11368 chromosome, complete genome. |
| 408 | NC_013361 | 5019544 | 5019577 | CCCTCTCCCTGAGGGAGAGGGTTAGGGTGAGGGG | Escherichia coli O26:H11 str. 11368 chromosome, complete genome. |
| 409 | NC_013361 | 5281085 | 5281120 | CCCTCGCCCCTCTGGGGAGAGGGTTAGGGTGAGGGG | Escherichia coli O26:H11 str. 11368 chromosome, complete genome. |
| 410 | NC_013361 | 5521062 | 5521097 | CCCTCGCCCCTTTGGGGAGAGGGTTAGGGTGAGGGG | Escherichia coli O26:H11 str. 11368 chromosome, complete genome. |
| 411 | NC_013361 | 5598398 | 5598363 | CCCTCGCCCCTCCGGGGAGAGGGTTAGGGTGAGGGG | Escherichia coli O26:H11 str. 11368 chromosome, complete genome. |
| 412 | NC_013361 | 5677540 | 5677507 | CCCTCTCCCTGTGGGAGAGGGCCGGGGTGAGGGC | Escherichia coli O26:H11 str. 11368 chromosome, complete genome. |
| 413 | NC_013364 | 68480 | 68445 | CCCTCGCCCCTTTGGGGAGAGGGCCGGGGTGAGGGG | Escherichia coli O111:H- str. 11128, complete genome. |
| 414 | NC_013364 | 297319 | 297286 | CCCTCTCCCTGTGGGAGAGGGTCGGGGTGAGGGC | Escherichia coli O111:H- str. 11128, complete genome. |
| 415 | NC_013364 | 297360 | 297395 | CCCTCGCCCCTTTGGGGAGAGGGTTAGGGTGAGGGG | Escherichia coli O111:H- str. 11128, complete genome. |
| 416 | NC_013364 | 430450 | 430485 | CCCTCGCCCCCTTGGGGAGAGGGTTAGGGTGAGGGG | Escherichia coli O111:H- str. 11128, complete genome. |
| 417 | NC_013364 | 542324 | 542359 | CCCTCGCCCCCTCGGGGAGAGGGTTAGGGTGAGGGG | Escherichia coli O111:H- str. 11128, complete genome. |
| 418 | NC_013364 | 671002 | 670967 | CCCTCTCCCTTCCAGGGTGAGGGCTGGGGTGAGGGT | Escherichia coli O111:H- str. 11128, complete genome. |
| 419 | NC_013364 | 1386866 | 1386901 | CCCTCGCCCCTTTGGGGAGAGGGTTAGGGTGAGGGG | Escherichia coli O111:H- str. 11128, complete genome. |
| 420 | NC_013364 | 2930630 | 2930665 | CCCTCTCCCCTTGCGGGAGAGGGTTAGGGTGAGGGG | Escherichia coli O111:H- str. 11128, complete genome. |
| 421 | NC_013364 | 3223253 | 3223218 | CCCTCGCCCCTTCGGGGAGAGGGTTAGGGTGAGGGG | Escherichia coli O111:H- str. 11128, complete genome. |
| 422 | NC_013364 | 3357748 | 3357715 | CCCTCTCCCTTAGGGAGAGGGCCGGGGTGAGGGT | Escherichia coli O111:H- str. 11128, complete genome. |
| 423 | NC_013364 | 3386324 | 3386289 | CCCTCGCCCCTTTGGGGAGAGGGTTAGGGTGAGGGG | Escherichia coli O111:H- str. 11128, complete genome. |
| 424 | NC_013364 | 3626033 | 3626068 | CCCTCGCCCTTTCAGGGAGAGGGCCGGGGTGAGGGT | Escherichia coli O111:H- str. 11128, complete genome. |
| 425 | NC_013364 | 3899758 | 3899793 | CCCTCTCCCTTCCAGGGAGAGGGTCGGGGTGAGGGT | Escherichia coli O111:H- str. 11128, complete genome. |
| 426 | NC_013364 | 3899857 | 3899821 | CCCTCGCCCCGTTTGGGGAGAGGGTTAGGGTGAGGGG | Escherichia coli O111:H- str. 11128, complete genome. |
| 427 | NC_013364 | 4160635 | 4160600 | CCCTCGCCCCTTTGGGGAGAGGGTTAGGGTGAGGGG | Escherichia coli O111:H- str. 11128, complete genome. |
| 428 | NC_013364 | 4168335 | 4168300 | CCCTCGCCCCTTTGGGGAGAGGGTTAGGGTGAGGGG | Escherichia coli O111:H- str. 11128, complete genome. |
| 429 | NC_013364 | 4266801 | 4266836 | CCCTCGCCCCTCTGGGGAGAGGGTTAGGGTGAGGGG | Escherichia coli O111:H- str. 11128, complete genome. |
| 430 | NC_013364 | 4335651 | 4335616 | CCCTCTCCCTTTCAGGGAGAGGGCTGGGGTGAGGGT | Escherichia coli O111:H- str. 11128, complete genome. |
| 431 | NC_013364 | 4335752 | 4335787 | CCCTCGCCCCTTTGGGGAGAGGGTTAGGGTGAGGGG | Escherichia coli O111:H- str. 11128, complete genome. |
| 432 | NC_013364 | 4451436 | 4451403 | CCCTCTCCCTGAGGGAGAGGGTTAGGGTGAGGGG | Escherichia coli O111:H- str. 11128, complete genome. |
| 433 | NC_013364 | 4521716 | 4521681 | CCCTCGCCCCTTTGGGGAGAGGGCTAGGGTGAGGGG | Escherichia coli O111:H- str. 11128, complete genome. |
| 434 | NC_013364 | 4635821 | 4635788 | CCCTCTCCCTGTGGGAGAGGGTCGGGGTGAGGGC | Escherichia coli O111:H- str. 11128, complete genome. |
| 435 | NC_013364 | 4750154 | 4750189 | CCCTCGCCCCTTTGGGGAGAGGGTTAGGGTGAGGGG | Escherichia coli O111:H- str. 11128, complete genome. |
| 436 | NC_013364 | 5046894 | 5046929 | CCCTCGCCCCTCTGGGGAGAGGGTTAGGGTGAGGGG | Escherichia coli O111:H- str. 11128, complete genome. |
| 437 | NC_013364 | 5237955 | 5237988 | CCCTCTCCCTTAGGGAGAGGGTTAGGGTGAGGGT | Escherichia coli O111:H- str. 11128, complete genome. |
| 438 | NC_013364 | 5242025 | 5242060 | CCCTCGCCCCTTTGGGGAGAGGGTTAGGGTGAGGGG | Escherichia coli O111:H- str. 11128, complete genome. |
| 439 | NC_013364 | 5281351 | 5281316 | CCCTCGCCCCTCCGGGGAGAGGGTTAGGGTGAGGGG | Escherichia coli O111:H- str. 11128, complete genome. |
| 440 | NC_013364 | 5351377 | 5351344 | CCCTCTCCCTGTGGGAGAGGGCCGGGGTGAGGGC | Escherichia coli O111:H- str. 11128, complete genome. |
| 441 | NC_013654 | 2294215 | 2294250 | CCCTCTCCCCTTGCGGGAGAGGGGACGGGTGAGGGG | Escherichia coli SE15, complete genome. |
| 442 | NC_013654 | 4489452 | 4489487 | CCCTCGCCCCTTTGGGGAGAGGGTTAGGGTGAGGGG | Escherichia coli SE15, complete genome. |
| 443 | NC_013941 | 168817 | 168850 | CCCTCTCCCTGTGGGAGAGGGCCGGGGTGAGGGC | Escherichia coli O55:H7 str. CB9615 chromosome, complete genome. |
| 444 | NC_013941 | 168901 | 168867 | CCCTCTCCCTTAAGGGAGAGGGTTAGGGTGAGGGT | Escherichia coli O55:H7 str. CB9615 chromosome, complete genome. |
| 445 | NC_013941 | 802984 | 802949 | CCCTCGCCCCATCGGGGAGAGGGTTAGGGTGAGGGG | Escherichia coli O55:H7 str. CB9615 chromosome, complete genome. |
| 446 | NC_013941 | 1392176 | 1392211 | CCCTCGCCCCTTTGGGGAGAGGGTTAGGGTGAGGGG | Escherichia coli O55:H7 str. CB9615 chromosome, complete genome. |
| 447 | NC_013941 | 2700196 | 2700162 | CCCTCGCCCCTTCGGGAGAGGGTTAGGGTGAGGGT | Escherichia coli O55:H7 str. CB9615 chromosome, complete genome. |
| 448 | NC_013941 | 3124121 | 3124086 | CCCTCGCCCCTTCGGGGAGAGGGTTAGGGTGAGGGG | Escherichia coli O55:H7 str. CB9615 chromosome, complete genome. |
| 449 | NC_013941 | 3868985 | 3869020 | CCCTCTCCCTTCCAGGGAGAGGGTCGGGGTGAGGGT | Escherichia coli O55:H7 str. CB9615 chromosome, complete genome. |
| 450 | NC_013941 | 3869084 | 3869048 | CCCTCGCCCCGTTTGGGGAGAGGGTTAGGGTGAGGGG | Escherichia coli O55:H7 str. CB9615 chromosome, complete genome. |
| 451 | NC_013941 | 4113536 | 4113501 | CCCTCGCCCCTTTGGGGAGAGGGTTAGGGTGAGGGG | Escherichia coli O55:H7 str. CB9615 chromosome, complete genome. |
| 452 | NC_013941 | 4582701 | 4582736 | CCCTCGCCCCTCTGGGGAGAGGGTTAGGGTGAGGGG | Escherichia coli O55:H7 str. CB9615 chromosome, complete genome. |
| 453 | NC_016902 | 30121 | 30156 | CCCTCGCCCCTTTGGGGAGAGGGCTAGGGTGAGGGG | Escherichia coli KO11FL chromosome, complete genome. |
| 454 | NC_016902 | 234031 | 233996 | CCCTCGCCCCTTTGGGGAGAGGGTTAGGGTGAGGGG | Escherichia coli KO11FL chromosome, complete genome. |
| 455 | NC_016902 | 234132 | 234167 | CCCTCTCCCTTTCAGGGAGAGGGCTGGGGTGAGGGT | Escherichia coli KO11FL chromosome, complete genome. |
| 456 | NC_016902 | 403317 | 403352 | CCCTCGCCCCTATGGGGAGAGGGTTAGGGTGAGGGG | Escherichia coli KO11FL chromosome, complete genome. |
| 457 | NC_016902 | 502585 | 502620 | CCCTCGCCCCTTTGGGGAGAGGGTTAGGGTGAGGGG | Escherichia coli KO11FL chromosome, complete genome. |
| 458 | NC_016902 | 654109 | 654145 | CCCTCGCCCCGTTTGGGGAGAGGGTTAGGGTGAGGGG | Escherichia coli KO11FL chromosome, complete genome. |
| 459 | NC_016902 | 654208 | 654173 | CCCTCTCCCTTCCAGGGAGAGGGTCGGGGTGAGGGT | Escherichia coli KO11FL chromosome, complete genome. |
| 460 | NC_016902 | 690184 | 690149 | CCCTCGCCCCTTCGGGGAGAGGGTTAGGGTGAGGGG | Escherichia coli KO11FL chromosome, complete genome. |
| 461 | NC_016902 | 863241 | 863276 | CCCTCACCCCTTTGGGGAGAGGGTTAGGGTGAGGGG | Escherichia coli KO11FL chromosome, complete genome. |
| 462 | NC_016902 | 863339 | 863304 | CCCTCGCCCTTTCAGGGAGAGGGCCGGGGTGAGGGT | Escherichia coli KO11FL chromosome, complete genome. |
| 463 | NC_016902 | 1134739 | 1134774 | CCCTCGCCCCTTTGGGGAGAGGGTTAGGGTGAGGGG | Escherichia coli KO11FL chromosome, complete genome. |
| 464 | NC_016902 | 3408939 | 3408974 | CCCTCGCCCCATCGGGGAGAGGGTTAGGGTGAGGGG | Escherichia coli KO11FL chromosome, complete genome. |
| 465 | NC_016902 | 3416349 | 3416384 | CCCTCTCCCTTCCAGGGTGAGGGCTGGGGTGAGGGT | Escherichia coli KO11FL chromosome, complete genome. |
| 466 | NC_016902 | 3416446 | 3416411 | CCCTCGCCCCTTTGGGGAGAGGGTTAGGGTGAGGGG | Escherichia coli KO11FL chromosome, complete genome. |
| 467 | NC_016902 | 3535814 | 3535779 | CCCTCGCCCCCTCGGGGAGAGGGTTAGGGTGAGGGG | Escherichia coli KO11FL chromosome, complete genome. |
| 468 | NC_016902 | 3645957 | 3645922 | CCCTCGCCCCCTTGGGGAGAGGGTTAGGGTGAGGGG | Escherichia coli KO11FL chromosome, complete genome. |
| 469 | NC_016902 | 3772575 | 3772608 | CCCTCTCCCTGTGGGAGAGGGCCGGGGTGAGGGC | Escherichia coli KO11FL chromosome, complete genome. |
| 470 | NC_016902 | 3779737 | 3779702 | CCCTCGCCCCTTTGGGGAGAGGGTTAGGGTGAGGGG | Escherichia coli KO11FL chromosome, complete genome. |
| 471 | NC_016902 | 3779778 | 3779811 | CCCTCTCCCTGTGGGAGAGGGTCGGGGTGAGGGC | Escherichia coli KO11FL chromosome, complete genome. |
| 472 | NC_016902 | 3945916 | 3945950 | CCCTCTCCCTTGAGGGAGAGGGTTAGGGTGAGGGT | Escherichia coli KO11FL chromosome, complete genome. |
| 473 | NC_016902 | 3946000 | 3945967 | CCCTCTCCCTGTGGGAGAGGGCCGGGGTGAGGGC | Escherichia coli KO11FL chromosome, complete genome. |
| 474 | NC_016902 | 4043491 | 4043526 | CCCTCGCCCCTTTGGGGAGAGGGCCGGGGTGAGGGG | Escherichia coli KO11FL chromosome, complete genome. |
| 475 | NC_016902 | 4131550 | 4131583 | CCCTCTCCCTGAGGGAGAGGGCCGGGGTGAGGGC | Escherichia coli KO11FL chromosome, complete genome. |
| 476 | NC_016902 | 4266380 | 4266345 | CCCTCGCCCCTTTGGGGAGAGGGTTAGGGTGAGGGG | Escherichia coli KO11FL chromosome, complete genome. |
| 477 | NC_016902 | 4270451 | 4270418 | CCCTCTCCCTTAGGGAGAGGGTTAGGGTGAGGGT | Escherichia coli KO11FL chromosome, complete genome. |
| 478 | NC_016902 | 4446313 | 4446278 | CCCTCGCCCCTCTGGGGAGAGGGTTAGGGTGAGGGG | Escherichia coli KO11FL chromosome, complete genome. |
| 479 | NC_016902 | 4706123 | 4706088 | CCCTCGCCCTTTCAGGGAGAGGGCCGGGGTGAGGGT | Escherichia coli KO11FL chromosome, complete genome. |
| 480 | NC_016902 | 4706176 | 4706211 | CCCTCGCCCCTACGGGGAGAGGGTTAGGGTGAGGGG | Escherichia coli KO11FL chromosome, complete genome. |
| 481 | NC_016902 | 4731626 | 4731591 | CCCTCGCCCCTTTGGGGAGAGGGTTAGGGTGAGGGG | Escherichia coli KO11FL chromosome, complete genome. |
| 482 | NC_016902 | 4842319 | 4842352 | CCCTCTCCCTGTGGGAGAGGGTCGGGGTGAGGGC | Escherichia coli KO11FL chromosome, complete genome. |
| 483 | NC_017625 | 102642 | 102607 | CCCTCGCCCCTCCGGGGAGAGGGCCGGGGTGAGGGG | Escherichia coli DH1 chromosome, complete genome. |
| 484 | NC_017625 | 102702 | 102735 | CCCTCTCCCTGAGGGAGAGGGTTAGGGTGAGGGG | Escherichia coli DH1 chromosome, complete genome. |
| 485 | NC_017625 | 275079 | 275044 | CCCTCGCCCCTCTGGGGAGAGGGTTAGGGTGAGGGG | Escherichia coli DH1 chromosome, complete genome. |
| 486 | NC_017625 | 378872 | 378907 | CCCTCGCCCCTTTGGGGAGAGGGTTAGGGTGAGGGG | Escherichia coli DH1 chromosome, complete genome. |
| 487 | NC_017625 | 493235 | 493270 | CCCTCGCCCCTTTGGGGTGAGGGTTAGGGTGAGGGG | Escherichia coli DH1 chromosome, complete genome. |
| 488 | NC_017625 | 644066 | 644102 | CCCTCGCCCCGTTTGGGGAGAGGGTTAGGGTGAGGGG | Escherichia coli DH1 chromosome, complete genome. |
| 489 | NC_017625 | 644165 | 644130 | CCCTCTCCCTTCCAGGGAGAGGGTCGGGGTGAGGGT | Escherichia coli DH1 chromosome, complete genome. |
| 490 | NC_017625 | 837676 | 837711 | CCCTCGCCCCTTTGGGGAGAGGGTTAGGGTGAGGGG | Escherichia coli DH1 chromosome, complete genome. |
| 491 | NC_017625 | 837774 | 837739 | CCCTCGCCCTTTCAGGGAGAGGGCCGGGGTGAGGGT | Escherichia coli DH1 chromosome, complete genome. |
| 492 | NC_017625 | 2528438 | 2528473 | CCCTCGCCCCTCTGGGGAGAGGGTTAGGGTGAGGGG | Escherichia coli DH1 chromosome, complete genome. |
| 493 | NC_017625 | 3256443 | 3256478 | CCCTCTCCCTTCCAGGGTGAGGGCTGGGGTGAGGGT | Escherichia coli DH1 chromosome, complete genome. |
| 494 | NC_017625 | 3256540 | 3256505 | CCCTCGCCCCTTTGGGGAGAGGGTTAGGGTGAGGGG | Escherichia coli DH1 chromosome, complete genome. |
| 495 | NC_017625 | 3373112 | 3373077 | CCCTCGCCCCCTCGGGGAGAGGGTTAGGGTGAGGGG | Escherichia coli DH1 chromosome, complete genome. |
| 496 | NC_017625 | 3482291 | 3482256 | CCCTCGCCCCCTTGGGGAGAGGGTTAGGGTGAGGGG | Escherichia coli DH1 chromosome, complete genome. |
| 497 | NC_017625 | 3582908 | 3582875 | CCCTCTCCCTGTGGGAGAGGGCCGGGGTGAGGGC | Escherichia coli DH1 chromosome, complete genome. |
| 498 | NC_017625 | 3590053 | 3590018 | CCCTCGCCCCTTTGGGGAGAGGGCCGGGGTGAGGGG | Escherichia coli DH1 chromosome, complete genome. |
| 499 | NC_017625 | 3706746 | 3706780 | CCCTCTCCCTTGAGGGAGAGGGTTAGGGTGAGGGT | Escherichia coli DH1 chromosome, complete genome. |
| 500 | NC_017625 | 3706830 | 3706797 | CCCTCTCCCTGTGGGAGAGGGCCGGGGTGAGGGC | Escherichia coli DH1 chromosome, complete genome. |
| 501 | NC_017625 | 3963146 | 3963181 | CCCTCGCCCCTCCGGGGAGAGGGTTAGGGTGAGGGG | Escherichia coli DH1 chromosome, complete genome. |
| 502 | NC_017625 | 4200296 | 4200261 | CCCTCGCCCCTCTGGGGAGAGGGTTAGGGTGAGGGG | Escherichia coli DH1 chromosome, complete genome. |
| 503 | NC_017625 | 4444018 | 4443983 | CCCTCGCCCCTTTGGGGAGAGGGTTAGGGAGAGGGG | Escherichia coli DH1 chromosome, complete genome. |
| 504 | NC_017625 | 4554979 | 4555012 | CCCTCTCCCTGTGGGAGAGGGTCGGGGTGAGGGC | Escherichia coli DH1 chromosome, complete genome. |
| 505 | NC_017625 | 4605974 | 4605939 | CCCTCGCCCCTCTGGGGAGAGGGTTAGGGTGAGGGG | Escherichia coli DH1 chromosome, complete genome. |
| 506 | NC_017626 | 73657 | 73622 | CCCTCGCCCCTTTGGGGAGAGGGCCGGGGTGAGGGG | Escherichia coli 042, complete genome. |
| 507 | NC_017626 | 300859 | 300826 | CCCTCTCCCTGTGGGAGAGGGTCGGGGTGAGGGC | Escherichia coli 042, complete genome. |
| 508 | NC_017626 | 300900 | 300935 | CCCTCGCCCCTTTGGGGAGAGGGTTGGGGTGAGGGG | Escherichia coli 042, complete genome. |
| 509 | NC_017626 | 308095 | 308062 | CCCTCTCCCTGTGGGAGAGGGCCGGGGTGAGGGC | Escherichia coli 042, complete genome. |
| 510 | NC_017626 | 308137 | 308171 | CCCTCTCCCTTGAGGGAGAGGGTTAGGGTGAGGGG | Escherichia coli 042, complete genome. |
| 511 | NC_017626 | 1486849 | 1486814 | CCCTCGCCCCTCTGGGGAGAGGGTTAGGGTGAGGGG | Escherichia coli 042, complete genome. |
| 512 | NC_017626 | 1487021 | 1487056 | CCCTCGCCCTTTCAGGGAGAGGGTTGGGGTGAGGGT | Escherichia coli 042, complete genome. |
| 513 | NC_017626 | 2824724 | 2824689 | CCCTCGCCCCTCTGGGGAGAGGGTTAGGGTGAGGGG | Escherichia coli 042, complete genome. |
| 514 | NC_017626 | 4612757 | 4612792 | CCCTCTCCCCTTTGGGGAGAGGGTTAGGGTGAGGGG | Escherichia coli 042, complete genome. |
| 515 | NC_017626 | 4789194 | 4789159 | CCCTCTCCCCAGAGGGGAGAGGGCTAGGGTGAGGGG | Escherichia coli 042, complete genome. |
| 516 | NC_017626 | 4789172 | 4789207 | CCCTCTCCCCTCTGGGGAGAGGGCCGGGGTGAGGGG | Escherichia coli 042, complete genome. |
| 517 | NC_017626 | 4794472 | 4794505 | CCCTCTCCCTGTGGGAGAGGGCCGGGGTGAGGGC | Escherichia coli 042, complete genome. |
| 518 | NC_017626 | 5222279 | 5222246 | CCCTCTCCCTGTGGGAGAGGGCCGGGGTGAGGGC | Escherichia coli 042, complete genome. |
| 519 | NC_017628 | 551330 | 551365 | CCCTCGCCCCTGTGGGGAGAGGGTTAGGGTGAGGGG | Escherichia coli IHE3034 chromosome, complete genome. |
| 520 | NC_017631 | 2823098 | 2823063 | CCCTCGCCCCTCTGGGGAGAGGGTTAGGGTGAGGGG | Escherichia coli ABU 83972 chromosome, complete genome. |
| 521 | NC_017631 | 5045295 | 5045260 | CCCTCGCCCCTCCGGGGAGAGGGTTAGGGTGAGGGG | Escherichia coli ABU 83972 chromosome, complete genome. |
| 522 | NC_017632 | 3053024 | 3052989 | CCCTCGCCCCTGTGGGGAGAGGGTTAGGGTGAGGGG | Escherichia coli UM146 chromosome, complete genome. |
| 523 | NC_017633 | 167865 | 167898 | CCCTCTCCCTGTGGGAGAGGGCCGGGGTGAGGGC | Escherichia coli ETEC H10407, complete genome. |
| 524 | NC_017633 | 167949 | 167915 | CCCTCTCCCTTGAGGGAGAGGGTTAGGGTGAGGGT | Escherichia coli ETEC H10407, complete genome. |
| 525 | NC_017633 | 449528 | 449563 | CCCTCGCCCCCTTGGGGAGAGGGTTAGGGTGAGGGG | Escherichia coli ETEC H10407, complete genome. |
| 526 | NC_017633 | 558837 | 558872 | CCCTCGCCCCCTCGGGGAGAGGGTTAGGGTGAGGGG | Escherichia coli ETEC H10407, complete genome. |
| 527 | NC_017633 | 678209 | 678244 | CCCTCGCCCCTTTGGGGAGAGGGTTAGGGTGAGGGG | Escherichia coli ETEC H10407, complete genome. |
| 528 | NC_017633 | 678306 | 678271 | CCCTCTCCCTTCCAGGGTGAGGGCTGGGGTGAGGGT | Escherichia coli ETEC H10407, complete genome. |
| 529 | NC_017633 | 1489759 | 1489724 | CCCTCGCCCCTCTGGGGAGAGGGTTAGGGTGAGGGG | Escherichia coli ETEC H10407, complete genome. |
| 530 | NC_017633 | 3348787 | 3348822 | CCCTCGCCCTTTCAGGGAGAGGGCCGGGGTGAGGGT | Escherichia coli ETEC H10407, complete genome. |
| 531 | NC_017633 | 3348885 | 3348850 | CCCTCGCCCCTTTGGGGAGAGGGTTAGGGTGAGGGG | Escherichia coli ETEC H10407, complete genome. |
| 532 | NC_017633 | 3630286 | 3630321 | CCCTCTCCCTTCCAGGGAGAGGGTCGGGGTGAGGGT | Escherichia coli ETEC H10407, complete genome. |
| 533 | NC_017633 | 3630385 | 3630349 | CCCTCGCCCCGTTTGGGGAGAGGGTTAGGGTGAGGGG | Escherichia coli ETEC H10407, complete genome. |
| 534 | NC_017633 | 3777005 | 3776970 | CCCTCGCCCCTTTGGGGAGAGGGTTAGGGTGAGGGG | Escherichia coli ETEC H10407, complete genome. |
| 535 | NC_017633 | 3891366 | 3891331 | CCCTCGCCCCTTTGGGGAGAGGGTTAGGGTGAGGGG | Escherichia coli ETEC H10407, complete genome. |
| 536 | NC_017633 | 3994376 | 3994411 | CCCTCGCCCCTCTGGGGAGAGGGTTAGGGTGAGGGG | Escherichia coli ETEC H10407, complete genome. |
| 537 | NC_017633 | 4162350 | 4162317 | CCCTCTCCCTGAGGGAGAGGGTTAGGGTGAGGGG | Escherichia coli ETEC H10407, complete genome. |
| 538 | NC_017633 | 4162410 | 4162445 | CCCTCGCCCCTCCGGGGAGAGGGCCGGGGTGAGGGG | Escherichia coli ETEC H10407, complete genome. |
| 539 | NC_017633 | 4330666 | 4330701 | CCCTCGCCCCTCTGGGGAGAGGGTTAGGGTGAGGGG | Escherichia coli ETEC H10407, complete genome. |
| 540 | NC_017633 | 4381664 | 4381631 | CCCTCTCCCTGTGGGAGAGGGTCGGGGTGAGGGC | Escherichia coli ETEC H10407, complete genome. |
| 541 | NC_017633 | 4776314 | 4776349 | CCCTCGCCCCTCTGGGGAGAGGGTTAGGGTGAGGGG | Escherichia coli ETEC H10407, complete genome. |
| 542 | NC_017633 | 5000256 | 5000291 | CCCTCGCCCCTTTGGGGAGAGGGTTAGGGTGAGGGG | Escherichia coli ETEC H10407, complete genome. |
| 543 | NC_017634 | 600518 | 600483 | CCCTCGCCCCACCGGGGAGAGGGTTAGGGTGAGGGG | Escherichia coli O83:H1 str. NRG 857C chromosome, complete genome. |
| 544 | NC_017634 | 2561759 | 2561724 | CCCTCGCCCCTTTGGGGAGAGGGTTAGGGTGAGGGG | Escherichia coli O83:H1 str. NRG 857C chromosome, complete genome. |
| 545 | NC_017641 | 167379 | 167412 | CCCTCTCCCTGTGGGAGAGGGCCGGGGTGAGGGC | Escherichia coli UMNK88 chromosome, complete genome. |
| 546 | NC_017641 | 167463 | 167429 | CCCTCTCCCTTGAGGGAGAGGGTTAGGGTGAGGGT | Escherichia coli UMNK88 chromosome, complete genome. |
| 547 | NC_017641 | 533844 | 533879 | CCCTCGCCCCCTCGGGGAGAGGGTTAGGGTGAGGGG | Escherichia coli UMNK88 chromosome, complete genome. |
| 548 | NC_017641 | 3479510 | 3479545 | CCCTCGCCCTTTCAGGGAGAGGGCCGGGGTGAGGGT | Escherichia coli UMNK88 chromosome, complete genome. |
| 549 | NC_017641 | 3479608 | 3479573 | CCCTCACCCCTTTGGGGAGAGGGTTAGGGTGAGGGG | Escherichia coli UMNK88 chromosome, complete genome. |
| 550 | NC_017641 | 3877905 | 3877870 | CCCTCGCCCCTTTGGGGAGAGGGTTAGGGTGAGGGG | Escherichia coli UMNK88 chromosome, complete genome. |
| 551 | NC_017641 | 3992287 | 3992252 | CCCTCGCCCCTTTGGGGAGAGGGTTAGGGTGAGGGG | Escherichia coli UMNK88 chromosome, complete genome. |
| 552 | NC_017641 | 4098507 | 4098542 | CCCTCGCCCCTCTGGGGAGAGGGTTAGGGTGAGGGG | Escherichia coli UMNK88 chromosome, complete genome. |
| 553 | NC_017641 | 4271010 | 4270977 | CCCTCTCCCTGAGGGAGAGGGTTAGGGTGAGGGG | Escherichia coli UMNK88 chromosome, complete genome. |
| 554 | NC_017641 | 4271070 | 4271105 | CCCTCGCCCCTCCGGGGAGAGGGCCGGGGTGAGGGG | Escherichia coli UMNK88 chromosome, complete genome. |
| 555 | NC_017641 | 4411420 | 4411455 | CCCTCGCCCCTCTGGGGAGAGGGTTAGGGTGAGGGG | Escherichia coli UMNK88 chromosome, complete genome. |
| 556 | NC_017641 | 4462418 | 4462385 | CCCTCTCCCTGTGGGAGAGGGTCGGGGTGAGGGC | Escherichia coli UMNK88 chromosome, complete genome. |
| 557 | NC_017641 | 4755321 | 4755356 | CCCTCGCCCCTTCGGGGAGAGGGTTAGGGTGAGGGG | Escherichia coli UMNK88 chromosome, complete genome. |
| 558 | NC_017644 | 2363016 | 2363051 | CCCTCTCCCCTTGCGGGAGAGGGGACGGGTGAGGGG | Escherichia coli NA114 chromosome, complete genome. |
| 559 | NC_017646 | 81221 | 81186 | CCCTCGCCCCTTCGGGGAGAGGGTTAGGGTGAGGGG | Escherichia coli O7:K1 str. CE10 chromosome, complete genome. |
| 560 | NC_017646 | 706015 | 706048 | CCCTCGCCCCTCGGGTGAGGGTTAGGGTGAGGGG | Escherichia coli O7:K1 str. CE10 chromosome, complete genome. |
| 561 | NC_017646 | 4917716 | 4917681 | CCCTCGCCCCTTCGGGGAGAGGGTTAGGGTGAGGGG | Escherichia coli O7:K1 str. CE10 chromosome, complete genome. |
| 562 | NC_017646 | 4917756 | 4917791 | CCCTCTCCCCTCTGGGGAGAGGGCCGGGGTGAGGGG | Escherichia coli O7:K1 str. CE10 chromosome, complete genome. |
| 563 | NC_017646 | 5119026 | 5119061 | CCCTCGCCCCTTTGGGGAGAGGGTTAGGGTGAGGGG | Escherichia coli O7:K1 str. CE10 chromosome, complete genome. |
| 564 | NC_017646 | 5182855 | 5182820 | CCCTCGCCCCTCCGGGGAGAGGGTTAGGGTGAGGGG | Escherichia coli O7:K1 str. CE10 chromosome, complete genome. |
| 565 | NC_017651 | 2789532 | 2789497 | CCCTCGCCCCTCTGGGGAGAGGGTTAGGGTGAGGGG | Escherichia coli str. 'clone D i2' chromosome, complete genome. |
| 566 | NC_017651 | 4952285 | 4952250 | CCCTCGCCCCTCCGGGGAGAGGGTTAGGGTGAGGGG | Escherichia coli str. 'clone D i2' chromosome, complete genome. |
| 567 | NC_017652 | 2789532 | 2789497 | CCCTCGCCCCTCTGGGGAGAGGGTTAGGGTGAGGGG | Escherichia coli str. 'clone D i14' chromosome, complete genome. |
| 568 | NC_017652 | 4952285 | 4952250 | CCCTCGCCCCTCCGGGGAGAGGGTTAGGGTGAGGGG | Escherichia coli str. 'clone D i14' chromosome, complete genome. |
| 569 | NC_017656 | 168817 | 168850 | CCCTCTCCCTGTGGGAGAGGGCCGGGGTGAGGGC | Escherichia coli O55:H7 str. RM12579 chromosome, complete genome. |
| 570 | NC_017656 | 168901 | 168867 | CCCTCTCCCTTAAGGGAGAGGGTTAGGGTGAGGGT | Escherichia coli O55:H7 str. RM12579 chromosome, complete genome. |
| 571 | NC_017656 | 797219 | 797184 | CCCTCGCCCCATCGGGGAGAGGGTTAGGGTGAGGGG | Escherichia coli O55:H7 str. RM12579 chromosome, complete genome. |
| 572 | NC_017656 | 1385750 | 1385785 | CCCTCGCCCCTTTGGGGAGAGGGTTAGGGTGAGGGG | Escherichia coli O55:H7 str. RM12579 chromosome, complete genome. |
| 573 | NC_017656 | 2710349 | 2710315 | CCCTCGCCCCTTCGGGAGAGGGTTAGGGTGAGGGT | Escherichia coli O55:H7 str. RM12579 chromosome, complete genome. |
| 574 | NC_017656 | 3134358 | 3134323 | CCCTCGCCCCTTCGGGGAGAGGGTTAGGGTGAGGGG | Escherichia coli O55:H7 str. RM12579 chromosome, complete genome. |
| 575 | NC_017656 | 3777468 | 3777503 | CCCTCTCCCTTCCAGGGAGAGGGTCGGGGTGAGGGT | Escherichia coli O55:H7 str. RM12579 chromosome, complete genome. |
| 576 | NC_017656 | 3777567 | 3777531 | CCCTCGCCCCGTTTGGGGAGAGGGTTAGGGTGAGGGG | Escherichia coli O55:H7 str. RM12579 chromosome, complete genome. |
| 577 | NC_017656 | 4022007 | 4021972 | CCCTCGCCCCTTTGGGGAGAGGGTTAGGGTGAGGGG | Escherichia coli O55:H7 str. RM12579 chromosome, complete genome. |
| 578 | NC_017656 | 4491146 | 4491181 | CCCTCGCCCCTCTGGGGAGAGGGTTAGGGTGAGGGG | Escherichia coli O55:H7 str. RM12579 chromosome, complete genome. |
| 579 | NC_017663 | 158502 | 158535 | CCCTCTCCCTGTGGGAGAGGGCCGGGGTGAGGGC | Escherichia coli P12b chromosome, complete genome. |
| 580 | NC_017663 | 158586 | 158552 | CCCTCTCCCTTGAGGGAGAGGGTTAGGGTGAGGGT | Escherichia coli P12b chromosome, complete genome. |
| 581 | NC_017663 | 263073 | 263040 | CCCTCTCCCTGTGGGAGAGGGTTGGGGTGAGGGC | Escherichia coli P12b chromosome, complete genome. |
| 582 | NC_017663 | 263114 | 263149 | CCCTCGCCCCTTTGGGGAGAGGGTTAGGGTGAGGGG | Escherichia coli P12b chromosome, complete genome. |
| 583 | NC_017663 | 408818 | 408853 | CCCTCGCCCCCTTGGGGAGAGGGTTAGGGTGAGGGG | Escherichia coli P12b chromosome, complete genome. |
| 584 | NC_017663 | 517978 | 518013 | CCCTCGCCCCCTCGGGGAGAGGGTTAGGGTGAGGGG | Escherichia coli P12b chromosome, complete genome. |
| 585 | NC_017663 | 622702 | 622737 | CCCTCGCCCCTTTGGGGAGAGGGTTAGGGTGAGGGG | Escherichia coli P12b chromosome, complete genome. |
| 586 | NC_017663 | 622799 | 622764 | CCCTCTCCCTTCCAGGGTGAGGGCTGGGGTGAGGGT | Escherichia coli P12b chromosome, complete genome. |
| 587 | NC_017663 | 1966903 | 1966938 | CCCTCGCCCCTCTGGGGAGAGGGTTAGGGTGAGGGG | Escherichia coli P12b chromosome, complete genome. |
| 588 | NC_017663 | 3278814 | 3278849 | CCCTCGCCCTTTCAGGGAGAGGGCCGGGGTGAGGGT | Escherichia coli P12b chromosome, complete genome. |
| 589 | NC_017663 | 3278912 | 3278877 | CCCTCGCCCCTTTGGGGAGAGGGTTAGGGTGAGGGG | Escherichia coli P12b chromosome, complete genome. |
| 590 | NC_017663 | 3510807 | 3510842 | CCCTCTCCCTTCCAGGGAGAGGGTCGGGGTGAGGGT | Escherichia coli P12b chromosome, complete genome. |
| 591 | NC_017663 | 3510906 | 3510870 | CCCTCGCCCCGTTTGGGGAGAGGGTTAGGGTGAGGGG | Escherichia coli P12b chromosome, complete genome. |
| 592 | NC_017663 | 3661980 | 3661945 | CCCTCGCCCCTTTGGGGAGAGGGTTAGGGTGAGGGG | Escherichia coli P12b chromosome, complete genome. |
| 593 | NC_017663 | 3772547 | 3772512 | CCCTCGCCCCTCTGGGGAGAGGGTTAGGGTGAGGGG | Escherichia coli P12b chromosome, complete genome. |
| 594 | NC_017663 | 4268881 | 4268848 | CCCTCTCCCTGTGGGTGAGGGTCGGGGTGAGGGC | Escherichia coli P12b chromosome, complete genome. |
| 595 | NC_017663 | 4628612 | 4628647 | CCCTCGCCCCTCTGGGGAGAGGGTTAGGGTGAGGGG | Escherichia coli P12b chromosome, complete genome. |
| 596 | NC_017663 | 4914647 | 4914614 | CCCTCTCCCTGAGGGAGAGGGCCGGGGTGAGGGC | Escherichia coli P12b chromosome, complete genome. |
| 597 | NC_017664 | 68411 | 68376 | CCCTCGCCCCTTTGGGGAGAGGGCCGGGGTGAGGGG | Escherichia coli W chromosome, complete genome. |
| 598 | NC_017664 | 165903 | 165936 | CCCTCTCCCTGTGGGAGAGGGCCGGGGTGAGGGC | Escherichia coli W chromosome, complete genome. |
| 599 | NC_017664 | 165987 | 165953 | CCCTCTCCCTTGAGGGAGAGGGTTAGGGTGAGGGT | Escherichia coli W chromosome, complete genome. |
| 600 | NC_017664 | 332126 | 332093 | CCCTCTCCCTGTGGGAGAGGGTCGGGGTGAGGGC | Escherichia coli W chromosome, complete genome. |
| 601 | NC_017664 | 332167 | 332202 | CCCTCGCCCCTTTGGGGAGAGGGTTAGGGTGAGGGG | Escherichia coli W chromosome, complete genome. |
| 602 | NC_017664 | 339329 | 339296 | CCCTCTCCCTGTGGGAGAGGGCCGGGGTGAGGGC | Escherichia coli W chromosome, complete genome. |
| 603 | NC_017664 | 465947 | 465982 | CCCTCGCCCCCTTGGGGAGAGGGTTAGGGTGAGGGG | Escherichia coli W chromosome, complete genome. |
| 604 | NC_017664 | 576090 | 576125 | CCCTCGCCCCCTCGGGGAGAGGGTTAGGGTGAGGGG | Escherichia coli W chromosome, complete genome. |
| 605 | NC_017664 | 695458 | 695493 | CCCTCGCCCCTTTGGGGAGAGGGTTAGGGTGAGGGG | Escherichia coli W chromosome, complete genome. |
| 606 | NC_017664 | 695555 | 695520 | CCCTCTCCCTTCCAGGGTGAGGGCTGGGGTGAGGGT | Escherichia coli W chromosome, complete genome. |
| 607 | NC_017664 | 702965 | 702930 | CCCTCGCCCCATCGGGGAGAGGGTTAGGGTGAGGGG | Escherichia coli W chromosome, complete genome. |
| 608 | NC_017664 | 2967695 | 2967660 | CCCTCGCCCCTTTGGGGAGAGGGTTAGGGTGAGGGG | Escherichia coli W chromosome, complete genome. |
| 609 | NC_017664 | 3239317 | 3239352 | CCCTCGCCCTTTCAGGGAGAGGGCCGGGGTGAGGGT | Escherichia coli W chromosome, complete genome. |
| 610 | NC_017664 | 3239415 | 3239380 | CCCTCACCCCTTTGGGGAGAGGGTTAGGGTGAGGGG | Escherichia coli W chromosome, complete genome. |
| 611 | NC_017664 | 3412473 | 3412508 | CCCTCGCCCCTTCGGGGAGAGGGTTAGGGTGAGGGG | Escherichia coli W chromosome, complete genome. |
| 612 | NC_017664 | 3448449 | 3448484 | CCCTCTCCCTTCCAGGGAGAGGGTCGGGGTGAGGGT | Escherichia coli W chromosome, complete genome. |
| 613 | NC_017664 | 3448548 | 3448512 | CCCTCGCCCCGTTTGGGGAGAGGGTTAGGGTGAGGGG | Escherichia coli W chromosome, complete genome. |
| 614 | NC_017664 | 3600072 | 3600037 | CCCTCGCCCCTTTGGGGAGAGGGTTAGGGTGAGGGG | Escherichia coli W chromosome, complete genome. |
| 615 | NC_017664 | 3699249 | 3699214 | CCCTCGCCCCTATGGGGAGAGGGTTAGGGTGAGGGG | Escherichia coli W chromosome, complete genome. |
| 616 | NC_017664 | 3868434 | 3868399 | CCCTCTCCCTTTCAGGGAGAGGGCTGGGGTGAGGGT | Escherichia coli W chromosome, complete genome. |
| 617 | NC_017664 | 3868535 | 3868570 | CCCTCGCCCCTTTGGGGAGAGGGTTAGGGTGAGGGG | Escherichia coli W chromosome, complete genome. |
| 618 | NC_017664 | 4072433 | 4072398 | CCCTCGCCCCTTTGGGGAGAGGGCTAGGGTGAGGGG | Escherichia coli W chromosome, complete genome. |
| 619 | NC_017664 | 4180405 | 4180372 | CCCTCTCCCTGTGGGAGAGGGTCGGGGTGAGGGC | Escherichia coli W chromosome, complete genome. |
| 620 | NC_017664 | 4291098 | 4291133 | CCCTCGCCCCTTTGGGGAGAGGGTTAGGGTGAGGGG | Escherichia coli W chromosome, complete genome. |
| 621 | NC_017664 | 4316548 | 4316513 | CCCTCGCCCCTACGGGGAGAGGGTTAGGGTGAGGGG | Escherichia coli W chromosome, complete genome. |
| 622 | NC_017664 | 4316601 | 4316636 | CCCTCGCCCTTTCAGGGAGAGGGCCGGGGTGAGGGT | Escherichia coli W chromosome, complete genome. |
| 623 | NC_017664 | 4569259 | 4569294 | CCCTCGCCCCTCTGGGGAGAGGGTTAGGGTGAGGGG | Escherichia coli W chromosome, complete genome. |
| 624 | NC_017664 | 4738902 | 4738935 | CCCTCTCCCTTAGGGAGAGGGTTAGGGTGAGGGT | Escherichia coli W chromosome, complete genome. |
| 625 | NC_017664 | 4742973 | 4743008 | CCCTCGCCCCTTTGGGGAGAGGGTTAGGGTGAGGGG | Escherichia coli W chromosome, complete genome. |
| 626 | NC_017664 | 4877804 | 4877771 | CCCTCTCCCTGAGGGAGAGGGCCGGGGTGAGGGC | Escherichia coli W chromosome, complete genome. |
| 627 | NC_017906 | 168869 | 168902 | CCCTCTCCCTGTGGGAGAGGGCCGGGGTGAGGGC | Escherichia coli Xuzhou21 chromosome, complete genome. |
| 628 | NC_017906 | 168953 | 168919 | CCCTCTCCCTTAAGGGAGAGGGTTAGGGTGAGGGT | Escherichia coli Xuzhou21 chromosome, complete genome. |
| 629 | NC_017906 | 714567 | 714532 | CCCTCGCCCCATCGGGGAGAGGGTTAGGGTGAGGGG | Escherichia coli Xuzhou21 chromosome, complete genome. |
| 630 | NC_017906 | 1495678 | 1495713 | CCCTCGCCCCTTTGGGGAGAGGGTTAGGGTGAGGGG | Escherichia coli Xuzhou21 chromosome, complete genome. |
| 631 | NC_017906 | 2887527 | 2887493 | CCCTCGCCCCTTCGGGAGAGGGTTAGGGTGAGGGT | Escherichia coli Xuzhou21 chromosome, complete genome. |
| 632 | NC_017906 | 3310693 | 3310658 | CCCTCGCCCCTTCGGGGAGAGGGTTAGGGTGAGGGG | Escherichia coli Xuzhou21 chromosome, complete genome. |
| 633 | NC_017906 | 3906491 | 3906526 | CCCTCTCCCTTCCAGGGAGAGGGTCGGGGTGAGGGT | Escherichia coli Xuzhou21 chromosome, complete genome. |
| 634 | NC_017906 | 3906590 | 3906554 | CCCTCGCCCCGTTTGGGGAGAGGGTTAGGGTGAGGGG | Escherichia coli Xuzhou21 chromosome, complete genome. |
| 635 | NC_017906 | 4053023 | 4052988 | CCCTCGCCCCTTTGGGGAGAGGGTTAGGGTGAGGGG | Escherichia coli Xuzhou21 chromosome, complete genome. |
| 636 | NC_017906 | 4152068 | 4152033 | CCCTCGCCCCTTTGGGGAGAGGGTTAGGGTGAGGGG | Escherichia coli Xuzhou21 chromosome, complete genome. |
| 637 | NC_017906 | 4622185 | 4622220 | CCCTCGCCCCTCTGGGGAGAGGGTTAGGGTGAGGGG | Escherichia coli Xuzhou21 chromosome, complete genome. |
| 638 | NC_018650 | 30884 | 30919 | CCCTCGCCCCTTTGGGGAGAGGGCTAGGGTGAGGGG | Escherichia coli O104:H4 str. 2009EL-2050 chromosome, complete genome. |
| 639 | NC_018650 | 264667 | 264632 | CCCTCGCCCCTTTGGGGAGAGGGTTAGGGTGAGGGG | Escherichia coli O104:H4 str. 2009EL-2050 chromosome, complete genome. |
| 640 | NC_018650 | 264768 | 264803 | CCCTCTCCCTTTCAGGGAGAGGGCTGGGGTGAGGGT | Escherichia coli O104:H4 str. 2009EL-2050 chromosome, complete genome. |
| 641 | NC_018650 | 460686 | 460721 | CCCTCGCCCCTTTGGGGAGAGGGTTAGGGTGAGGGG | Escherichia coli O104:H4 str. 2009EL-2050 chromosome, complete genome. |
| 642 | NC_018650 | 468385 | 468420 | CCCTCGCCCCTTTGGGGAGAGGGTTAGGGTGAGGGG | Escherichia coli O104:H4 str. 2009EL-2050 chromosome, complete genome. |
| 643 | NC_018650 | 719687 | 719723 | CCCTCGCCCCGTTTGGGGAGAGGGTTAGGGTGAGGGG | Escherichia coli O104:H4 str. 2009EL-2050 chromosome, complete genome. |
| 644 | NC_018650 | 719786 | 719751 | CCCTCTCCCTTCCAGGGAGAGGGTCGGGGTGAGGGT | Escherichia coli O104:H4 str. 2009EL-2050 chromosome, complete genome. |
| 645 | NC_018650 | 755763 | 755728 | CCCTCGCCCCTTCGGGGAGAGGGTTAGGGTGAGGGG | Escherichia coli O104:H4 str. 2009EL-2050 chromosome, complete genome. |
| 646 | NC_018650 | 1016568 | 1016533 | CCCTCGCCCTTTCAGGGAGAGGGCCGGGGTGAGGGT | Escherichia coli O104:H4 str. 2009EL-2050 chromosome, complete genome. |
| 647 | NC_018650 | 1252976 | 1253011 | CCCTCGCCCCTTTGGGGAGAGGGTTAGGGTGAGGGG | Escherichia coli O104:H4 str. 2009EL-2050 chromosome, complete genome. |
| 648 | NC_018650 | 1281819 | 1281852 | CCCTCTCCCTTAGGGAGAGGGCCGGGGTGAGGGT | Escherichia coli O104:H4 str. 2009EL-2050 chromosome, complete genome. |
| 649 | NC_018650 | 1705043 | 1705008 | CCCTCTCCCCTTGCGGGAGAGGGTTAGGGTGAGGGG | Escherichia coli O104:H4 str. 2009EL-2050 chromosome, complete genome. |
| 650 | NC_018650 | 3793440 | 3793475 | CCCTCTCCCTTCCAGGGTGAGGGCTGGGGTGAGGGT | Escherichia coli O104:H4 str. 2009EL-2050 chromosome, complete genome. |
| 651 | NC_018650 | 3793537 | 3793502 | CCCTCGCCCCTTTGGGGAGAGGGTTAGGGTGAGGGG | Escherichia coli O104:H4 str. 2009EL-2050 chromosome, complete genome. |
| 652 | NC_018650 | 3909097 | 3909062 | CCCTCGCCCCCTCGGGGAGAGGGTTAGGGTGAGGGG | Escherichia coli O104:H4 str. 2009EL-2050 chromosome, complete genome. |
| 653 | NC_018650 | 4019091 | 4019056 | CCCTCGCCCCCTTGGGGAGAGGGTTAGGGTGAGGGG | Escherichia coli O104:H4 str. 2009EL-2050 chromosome, complete genome. |
| 654 | NC_018650 | 4263523 | 4263557 | CCCTCTCCCTTGAGGGAGAGGGTTAGGGTGAGGGT | Escherichia coli O104:H4 str. 2009EL-2050 chromosome, complete genome. |
| 655 | NC_018650 | 4263607 | 4263574 | CCCTCTCCCTGTGGGAGAGGGCCGGGGTGAGGGC | Escherichia coli O104:H4 str. 2009EL-2050 chromosome, complete genome. |
| 656 | NC_018650 | 4361112 | 4361147 | CCCTCGCCCCTTTGGGGAGAGGGCCGGGGTGAGGGG | Escherichia coli O104:H4 str. 2009EL-2050 chromosome, complete genome. |
| 657 | NC_018650 | 4447892 | 4447925 | CCCTCTCCCTGAGGGAGAGGGCCGGGGTGAGGGC | Escherichia coli O104:H4 str. 2009EL-2050 chromosome, complete genome. |
| 658 | NC_018650 | 4585690 | 4585655 | CCCTCGCCCCTTTGGGGAGAGGGTTAGGGTGAGGGG | Escherichia coli O104:H4 str. 2009EL-2050 chromosome, complete genome. |
| 659 | NC_018650 | 4589761 | 4589728 | CCCTCTCCCTTAGGGAGAGGGTTAGGGTGAGGGT | Escherichia coli O104:H4 str. 2009EL-2050 chromosome, complete genome. |
| 660 | NC_018650 | 4624960 | 4624925 | CCCTCGCCCCTTCGGGGAGAGGGTTAGGGTGAGGGG | Escherichia coli O104:H4 str. 2009EL-2050 chromosome, complete genome. |
| 661 | NC_018650 | 4809210 | 4809177 | CCCTCTCCCTCCGGGAGAGGGCCGGGGTGAGGGG | Escherichia coli O104:H4 str. 2009EL-2050 chromosome, complete genome. |
| 662 | NC_018650 | 4809251 | 4809286 | CCCTCGCCCCTTCGGGGAGAGGGTTAGGGTGAGGGG | Escherichia coli O104:H4 str. 2009EL-2050 chromosome, complete genome. |
| 663 | NC_018650 | 4812941 | 4812906 | CCCTCGCCCCTCTGGGGAGAGGGTTAGGGTGAGGGG | Escherichia coli O104:H4 str. 2009EL-2050 chromosome, complete genome. |
| 664 | NC_018650 | 5035634 | 5035599 | CCCTCGCCCTTTCAGGGAGAGGGCCGGGGTGAGGGT | Escherichia coli O104:H4 str. 2009EL-2050 chromosome, complete genome. |
| 665 | NC_018650 | 5035687 | 5035722 | CCCTCGCCCCTACGGGGAGAGGGTTAGGGTGAGGGG | Escherichia coli O104:H4 str. 2009EL-2050 chromosome, complete genome. |
| 666 | NC_018650 | 5172275 | 5172308 | CCCTCTCCCTGTGGGAGAGGGTCGGGGTGAGGGC | Escherichia coli O104:H4 str. 2009EL-2050 chromosome, complete genome. |
| 667 | NC_018658 | 30107 | 30142 | CCCTCGCCCCTTTGGGGAGAGGGCTAGGGTGAGGGG | Escherichia coli O104:H4 str. 2011C-3493 chromosome, complete genome. |
| 668 | NC_018658 | 262590 | 262555 | CCCTCGCCCCTTTGGGGAGAGGGTTAGGGTGAGGGG | Escherichia coli O104:H4 str. 2011C-3493 chromosome, complete genome. |
| 669 | NC_018658 | 262691 | 262726 | CCCTCTCCCTTTCAGGGAGAGGGCTGGGGTGAGGGT | Escherichia coli O104:H4 str. 2011C-3493 chromosome, complete genome. |
| 670 | NC_018658 | 456058 | 456093 | CCCTCGCCCCTTTGGGGAGAGGGTTAGGGTGAGGGG | Escherichia coli O104:H4 str. 2011C-3493 chromosome, complete genome. |
| 671 | NC_018658 | 463757 | 463792 | CCCTCGCCCCTTTGGGGAGAGGGTTAGGGTGAGGGG | Escherichia coli O104:H4 str. 2011C-3493 chromosome, complete genome. |
| 672 | NC_018658 | 715173 | 715209 | CCCTCGCCCCGTTTGGGGAGAGGGTTAGGGTGAGGGG | Escherichia coli O104:H4 str. 2011C-3493 chromosome, complete genome. |
| 673 | NC_018658 | 715272 | 715237 | CCCTCTCCCTTCCAGGGAGAGGGTCGGGGTGAGGGT | Escherichia coli O104:H4 str. 2011C-3493 chromosome, complete genome. |
| 674 | NC_018658 | 751249 | 751214 | CCCTCGCCCCTTCGGGGAGAGGGTTAGGGTGAGGGG | Escherichia coli O104:H4 str. 2011C-3493 chromosome, complete genome. |
| 675 | NC_018658 | 1013121 | 1013086 | CCCTCGCCCTTTCAGGGAGAGGGCCGGGGTGAGGGT | Escherichia coli O104:H4 str. 2011C-3493 chromosome, complete genome. |
| 676 | NC_018658 | 1249515 | 1249550 | CCCTCGCCCCTTTGGGGAGAGGGTTAGGGTGAGGGG | Escherichia coli O104:H4 str. 2011C-3493 chromosome, complete genome. |
| 677 | NC_018658 | 1277808 | 1277841 | CCCTCTCCCTTAGGGAGAGGGCCGGGGTGAGGGT | Escherichia coli O104:H4 str. 2011C-3493 chromosome, complete genome. |
| 678 | NC_018658 | 1717779 | 1717744 | CCCTCTCCCCTTGCGGGAGAGGGTTAGGGTGAGGGG | Escherichia coli O104:H4 str. 2011C-3493 chromosome, complete genome. |
| 679 | NC_018658 | 3801436 | 3801471 | CCCTCTCCCTTCCAGGGTGAGGGCTGGGGTGAGGGT | Escherichia coli O104:H4 str. 2011C-3493 chromosome, complete genome. |
| 680 | NC_018658 | 3801533 | 3801498 | CCCTCGCCCCTTTGGGGAGAGGGTTAGGGTGAGGGG | Escherichia coli O104:H4 str. 2011C-3493 chromosome, complete genome. |
| 681 | NC_018658 | 3918441 | 3918406 | CCCTCGCCCCCTCGGGGAGAGGGTTAGGGTGAGGGG | Escherichia coli O104:H4 str. 2011C-3493 chromosome, complete genome. |
| 682 | NC_018658 | 4028435 | 4028400 | CCCTCGCCCCCTTGGGGAGAGGGTTAGGGTGAGGGG | Escherichia coli O104:H4 str. 2011C-3493 chromosome, complete genome. |
| 683 | NC_018658 | 4285814 | 4285848 | CCCTCTCCCTTGAGGGAGAGGGTTAGGGTGAGGGT | Escherichia coli O104:H4 str. 2011C-3493 chromosome, complete genome. |
| 684 | NC_018658 | 4285898 | 4285865 | CCCTCTCCCTGTGGGAGAGGGCCGGGGTGAGGGC | Escherichia coli O104:H4 str. 2011C-3493 chromosome, complete genome. |
| 685 | NC_018658 | 4383402 | 4383437 | CCCTCGCCCCTTTGGGGAGAGGGCCGGGGTGAGGGG | Escherichia coli O104:H4 str. 2011C-3493 chromosome, complete genome. |
| 686 | NC_018658 | 4470182 | 4470215 | CCCTCTCCCTGAGGGAGAGGGCCGGGGTGAGGGC | Escherichia coli O104:H4 str. 2011C-3493 chromosome, complete genome. |
| 687 | NC_018658 | 4607841 | 4607806 | CCCTCGCCCCTTTGGGGAGAGGGTTAGGGTGAGGGG | Escherichia coli O104:H4 str. 2011C-3493 chromosome, complete genome. |
| 688 | NC_018658 | 4611912 | 4611879 | CCCTCTCCCTTAGGGAGAGGGTTAGGGTGAGGGT | Escherichia coli O104:H4 str. 2011C-3493 chromosome, complete genome. |
| 689 | NC_018658 | 4646333 | 4646298 | CCCTCGCCCCTTCGGGGAGAGGGTTAGGGTGAGGGG | Escherichia coli O104:H4 str. 2011C-3493 chromosome, complete genome. |
| 690 | NC_018658 | 4828985 | 4828952 | CCCTCTCCCTCCGGGAGAGGGCCGGGGTGAGGGG | Escherichia coli O104:H4 str. 2011C-3493 chromosome, complete genome. |
| 691 | NC_018658 | 4829026 | 4829061 | CCCTCGCCCCTTCGGGGAGAGGGTTAGGGTGAGGGG | Escherichia coli O104:H4 str. 2011C-3493 chromosome, complete genome. |
| 692 | NC_018658 | 4832716 | 4832681 | CCCTCGCCCCTCTGGGGAGAGGGTTAGGGTGAGGGG | Escherichia coli O104:H4 str. 2011C-3493 chromosome, complete genome. |
| 693 | NC_018658 | 5055499 | 5055464 | CCCTCGCCCTTTCAGGGAGAGGGCCGGGGTGAGGGT | Escherichia coli O104:H4 str. 2011C-3493 chromosome, complete genome. |
| 694 | NC_018658 | 5055552 | 5055587 | CCCTCGCCCCTACGGGGAGAGGGTTAGGGTGAGGGG | Escherichia coli O104:H4 str. 2011C-3493 chromosome, complete genome. |
| 695 | NC_018658 | 5192141 | 5192174 | CCCTCTCCCTGTGGGAGAGGGTCGGGGTGAGGGC | Escherichia coli O104:H4 str. 2011C-3493 chromosome, complete genome. |
| 696 | NC_018661 | 30864 | 30899 | CCCTCGCCCCTTTGGGGAGAGGGCTAGGGTGAGGGG | Escherichia coli O104:H4 str. 2009EL-2071 chromosome, complete genome. |
| 697 | NC_018661 | 252563 | 252528 | CCCTCGCCCCTTTGGGGAGAGGGTTAGGGTGAGGGG | Escherichia coli O104:H4 str. 2009EL-2071 chromosome, complete genome. |
| 698 | NC_018661 | 252664 | 252699 | CCCTCTCCCTTTCAGGGAGAGGGCTGGGGTGAGGGT | Escherichia coli O104:H4 str. 2009EL-2071 chromosome, complete genome. |
| 699 | NC_018661 | 449359 | 449394 | CCCTCGCCCCTTTGGGGAGAGGGTTAGGGTGAGGGG | Escherichia coli O104:H4 str. 2009EL-2071 chromosome, complete genome. |
| 700 | NC_018661 | 457058 | 457093 | CCCTCGCCCCTTTGGGGAGAGGGTTAGGGTGAGGGG | Escherichia coli O104:H4 str. 2009EL-2071 chromosome, complete genome. |
| 701 | NC_018661 | 708697 | 708733 | CCCTCGCCCCGTTTGGGGAGAGGGTTAGGGTGAGGGG | Escherichia coli O104:H4 str. 2009EL-2071 chromosome, complete genome. |
| 702 | NC_018661 | 708796 | 708761 | CCCTCTCCCTTCCAGGGAGAGGGTCGGGGTGAGGGT | Escherichia coli O104:H4 str. 2009EL-2071 chromosome, complete genome. |
| 703 | NC_018661 | 744773 | 744738 | CCCTCGCCCCTTCGGGGAGAGGGTTAGGGTGAGGGG | Escherichia coli O104:H4 str. 2009EL-2071 chromosome, complete genome. |
| 704 | NC_018661 | 1006228 | 1006193 | CCCTCGCCCTTTCAGGGAGAGGGCCGGGGTGAGGGT | Escherichia coli O104:H4 str. 2009EL-2071 chromosome, complete genome. |
| 705 | NC_018661 | 1242717 | 1242752 | CCCTCGCCCCTTTGGGGAGAGGGTTAGGGTGAGGGG | Escherichia coli O104:H4 str. 2009EL-2071 chromosome, complete genome. |
| 706 | NC_018661 | 1271561 | 1271594 | CCCTCTCCCTTAGGGAGAGGGCCGGGGTGAGGGT | Escherichia coli O104:H4 str. 2009EL-2071 chromosome, complete genome. |
| 707 | NC_018661 | 1712733 | 1712698 | CCCTCTCCCCTTGCGGGAGAGGGTTAGGGTGAGGGG | Escherichia coli O104:H4 str. 2009EL-2071 chromosome, complete genome. |
| 708 | NC_018661 | 3851063 | 3851098 | CCCTCTCCCTTCCAGGGTGAGGGCTGGGGTGAGGGT | Escherichia coli O104:H4 str. 2009EL-2071 chromosome, complete genome. |
| 709 | NC_018661 | 3851160 | 3851125 | CCCTCGCCCCTTTGGGGAGAGGGTTAGGGTGAGGGG | Escherichia coli O104:H4 str. 2009EL-2071 chromosome, complete genome. |
| 710 | NC_018661 | 3968069 | 3968034 | CCCTCGCCCCCTCGGGGAGAGGGTTAGGGTGAGGGG | Escherichia coli O104:H4 str. 2009EL-2071 chromosome, complete genome. |
| 711 | NC_018661 | 4078063 | 4078028 | CCCTCGCCCCCTTGGGGAGAGGGTTAGGGTGAGGGG | Escherichia coli O104:H4 str. 2009EL-2071 chromosome, complete genome. |
| 712 | NC_018661 | 4322494 | 4322528 | CCCTCTCCCTTGAGGGAGAGGGTTAGGGTGAGGGT | Escherichia coli O104:H4 str. 2009EL-2071 chromosome, complete genome. |
| 713 | NC_018661 | 4322578 | 4322545 | CCCTCTCCCTGTGGGAGAGGGCCGGGGTGAGGGC | Escherichia coli O104:H4 str. 2009EL-2071 chromosome, complete genome. |
| 714 | NC_018661 | 4420083 | 4420118 | CCCTCGCCCCTTTGGGGAGAGGGCCGGGGTGAGGGG | Escherichia coli O104:H4 str. 2009EL-2071 chromosome, complete genome. |
| 715 | NC_018661 | 4506863 | 4506896 | CCCTCTCCCTGAGGGAGAGGGCCGGGGTGAGGGC | Escherichia coli O104:H4 str. 2009EL-2071 chromosome, complete genome. |
| 716 | NC_018661 | 4644662 | 4644627 | CCCTCGCCCCTTTGGGGAGAGGGTTAGGGTGAGGGG | Escherichia coli O104:H4 str. 2009EL-2071 chromosome, complete genome. |
| 717 | NC_018661 | 4648733 | 4648700 | CCCTCTCCCTTAGGGAGAGGGTTAGGGTGAGGGT | Escherichia coli O104:H4 str. 2009EL-2071 chromosome, complete genome. |
| 718 | NC_018661 | 4683931 | 4683896 | CCCTCGCCCCTTCGGGGAGAGGGTTAGGGTGAGGGG | Escherichia coli O104:H4 str. 2009EL-2071 chromosome, complete genome. |
| 719 | NC_018661 | 4868735 | 4868702 | CCCTCTCCCTCCGGGAGAGGGCCGGGGTGAGGGG | Escherichia coli O104:H4 str. 2009EL-2071 chromosome, complete genome. |
| 720 | NC_018661 | 4868776 | 4868811 | CCCTCGCCCCTTCGGGGAGAGGGTTAGGGTGAGGGG | Escherichia coli O104:H4 str. 2009EL-2071 chromosome, complete genome. |
| 721 | NC_018661 | 4872466 | 4872431 | CCCTCGCCCCTCTGGGGAGAGGGTTAGGGTGAGGGG | Escherichia coli O104:H4 str. 2009EL-2071 chromosome, complete genome. |
| 722 | NC_018661 | 5095064 | 5095029 | CCCTCGCCCTTTCAGGGAGAGGGCCGGGGTGAGGGT | Escherichia coli O104:H4 str. 2009EL-2071 chromosome, complete genome. |
| 723 | NC_018661 | 5095117 | 5095152 | CCCTCGCCCCTACGGGGAGAGGGTTAGGGTGAGGGG | Escherichia coli O104:H4 str. 2009EL-2071 chromosome, complete genome. |
| 724 | NC_018661 | 5231704 | 5231737 | CCCTCTCCCTGTGGGAGAGGGTCGGGGTGAGGGC | Escherichia coli O104:H4 str. 2009EL-2071 chromosome, complete genome. |
| 725 | NC_020163 | 29573 | 29608 | CCCTCGCCCCTTTGGGGAGAGGGTTAGGGTGAGGGG | Escherichia coli APEC O78, complete genome. |
| 726 | NC_020163 | 304531 | 304566 | CCCTCGCCCCTCTGGGGAGAGGGTTAGGGTGAGGGG | Escherichia coli APEC O78, complete genome. |
| 727 | NC_020163 | 308221 | 308186 | CCCTCGCCCCTTCGGGGAGAGGGTTAGGGTGAGGGG | Escherichia coli APEC O78, complete genome. |
| 728 | NC_020163 | 308262 | 308295 | CCCTCTCCCTCCGGGAGAGGGCCGGGGTGAGGGG | Escherichia coli APEC O78, complete genome. |
| 729 | NC_020163 | 449498 | 449533 | CCCTCGCCCCTTCGGGGAGAGGGTTAGGGTGAGGGG | Escherichia coli APEC O78, complete genome. |
| 730 | NC_020163 | 681615 | 681582 | CCCTCTCCCTGAGGGAGAGGGCCGGGGTGAGGGC | Escherichia coli APEC O78, complete genome. |
| 731 | NC_020163 | 866174 | 866207 | CCCTCTCCCTGTGGGAGAGGGCCGGGGTGAGGGC | Escherichia coli APEC O78, complete genome. |
| 732 | NC_020163 | 866258 | 866224 | CCCTCTCCCTTGAGGGAGAGGGTTAGGGTGAGGGT | Escherichia coli APEC O78, complete genome. |
| 733 | NC_020163 | 1100399 | 1100434 | CCCTCGCCCCCTTGGGGAGAGGGTTAGGGTGAGGGG | Escherichia coli APEC O78, complete genome. |
| 734 | NC_020163 | 1209593 | 1209628 | CCCTCGCCCCCTCGGGGAGAGGGTTAGGGTGAGGGG | Escherichia coli APEC O78, complete genome. |
| 735 | NC_020163 | 1344373 | 1344408 | CCCTCGCCCCTTTGGGGAGAGGGTTAGGGTGAGGGG | Escherichia coli APEC O78, complete genome. |
| 736 | NC_020163 | 1344470 | 1344435 | CCCTCTCCCTTCCAGGGTGAGGGCTGGGGTGAGGGT | Escherichia coli APEC O78, complete genome. |
| 737 | NC_020163 | 3407207 | 3407172 | CCCTCGCCCCTTCGGGGAGAGGGTTAGGGTGAGGGG | Escherichia coli APEC O78, complete genome. |
| 738 | NC_020163 | 3553129 | 3553094 | CCCTCGCCCCTTTGGGGAGAGGGTTAGGGTGAGGGG | Escherichia coli APEC O78, complete genome. |
| 739 | NC_020163 | 3789945 | 3789980 | CCCTCGCCCTTTCAGGGAGAGGGCCGGGGTGAGGGT | Escherichia coli APEC O78, complete genome. |
| 740 | NC_020163 | 3790043 | 3790008 | CCCTCACCCCTTTGGGGAGAGGGTTAGGGTGAGGGG | Escherichia coli APEC O78, complete genome. |
| 741 | NC_020163 | 3999158 | 3999193 | CCCTCTCCCTTCCAGGGAGAGGGTCGGGGTGAGGGT | Escherichia coli APEC O78, complete genome. |
| 742 | NC_020163 | 3999259 | 3999223 | CCCTCGCCCCGTTTGGGGAGAGGGTTAGGGTGAGGGG | Escherichia coli APEC O78, complete genome. |
| 743 | NC_020163 | 4274039 | 4274004 | CCCTCGCCCCTTTGGGGAGAGGGTTAGGGTGAGGGG | Escherichia coli APEC O78, complete genome. |
| 744 | NC_020163 | 4431206 | 4431171 | CCCTCTCCCTTTCAGGGAGAGGGCTGGGGTGAGGGT | Escherichia coli APEC O78, complete genome. |
| 745 | NC_020163 | 4431307 | 4431342 | CCCTCGCCCCTTTGGGGAGAGGGTTAGGGTGAGGGG | Escherichia coli APEC O78, complete genome. |
| 746 | NC_020163 | 4613485 | 4613450 | CCCTCGCCCCTTTGGGGAGAGGGCTAGGGTGAGGGG | Escherichia coli APEC O78, complete genome. |
| 747 | NC_020163 | 4724490 | 4724457 | CCCTCTCCCTGTGGGAGAGGGTCGGGGTGAGGGC | Escherichia coli APEC O78, complete genome. |
| 748 | NC_020518 | 159372 | 159405 | CCCTCTCCCTGTGGGAGAGGGCCGGGGTGAGGGC | Escherichia coli str. K-12 substr. MDS42 DNA, complete genome. |
| 749 | NC_020518 | 159456 | 159422 | CCCTCTCCCTTGAGGGAGAGGGTTAGGGTGAGGGT | Escherichia coli str. K-12 substr. MDS42 DNA, complete genome. |
| 750 | NC_020518 | 306315 | 306350 | CCCTCGCCCCCTTGGGGAGAGGGTTAGGGTGAGGGG | Escherichia coli str. K-12 substr. MDS42 DNA, complete genome. |
| 751 | NC_020518 | 405586 | 405621 | CCCTCGCCCCCTCGGGGAGAGGGTTAGGGTGAGGGG | Escherichia coli str. K-12 substr. MDS42 DNA, complete genome. |
| 752 | NC_020518 | 498095 | 498130 | CCCTCGCCCCTTTGGGGAGAGGGTTAGGGTGAGGGG | Escherichia coli str. K-12 substr. MDS42 DNA, complete genome. |
| 753 | NC_020518 | 498192 | 498157 | CCCTCTCCCTTCCAGGGTGAGGGCTGGGGTGAGGGT | Escherichia coli str. K-12 substr. MDS42 DNA, complete genome. |
| 754 | NC_020518 | 1155468 | 1155433 | CCCTCGCCCCTCTGGGGAGAGGGTTAGGGTGAGGGG | Escherichia coli str. K-12 substr. MDS42 DNA, complete genome. |
| 755 | NC_020518 | 2552647 | 2552682 | CCCTCGCCCTTTCAGGGAGAGGGCCGGGGTGAGGGT | Escherichia coli str. K-12 substr. MDS42 DNA, complete genome. |
| 756 | NC_020518 | 2552745 | 2552710 | CCCTCGCCCCTTTGGGGAGAGGGTTAGGGTGAGGGG | Escherichia coli str. K-12 substr. MDS42 DNA, complete genome. |
| 757 | NC_020518 | 2712932 | 2712967 | CCCTCTCCCTTCCAGGGAGAGGGTCGGGGTGAGGGT | Escherichia coli str. K-12 substr. MDS42 DNA, complete genome. |
| 758 | NC_020518 | 2713031 | 2712995 | CCCTCGCCCCGTTTGGGGAGAGGGTTAGGGTGAGGGG | Escherichia coli str. K-12 substr. MDS42 DNA, complete genome. |
| 759 | NC_020518 | 2858381 | 2858346 | CCCTCGCCCCTTTGGGGTGAGGGTTAGGGTGAGGGG | Escherichia coli str. K-12 substr. MDS42 DNA, complete genome. |
| 760 | NC_020518 | 2956818 | 2956783 | CCCTCGCCCCTTTGGGGAGAGGGTTAGGGTGAGGGG | Escherichia coli str. K-12 substr. MDS42 DNA, complete genome. |
| 761 | NC_020518 | 3056701 | 3056736 | CCCTCGCCCCTCTGGGGAGAGGGTTAGGGTGAGGGG | Escherichia coli str. K-12 substr. MDS42 DNA, complete genome. |
| 762 | NC_020518 | 3210275 | 3210242 | CCCTCTCCCTGAGGGAGAGGGTTAGGGTGAGGGG | Escherichia coli str. K-12 substr. MDS42 DNA, complete genome. |
| 763 | NC_020518 | 3210335 | 3210370 | CCCTCGCCCCTCCGGGGAGAGGGCCGGGGTGAGGGG | Escherichia coli str. K-12 substr. MDS42 DNA, complete genome. |
| 764 | NC_020518 | 3337708 | 3337743 | CCCTCGCCCCTCTGGGGAGAGGGTTAGGGTGAGGGG | Escherichia coli str. K-12 substr. MDS42 DNA, complete genome. |
| 765 | NC_020518 | 3388704 | 3388671 | CCCTCTCCCTGTGGGAGAGGGTCGGGGTGAGGGC | Escherichia coli str. K-12 substr. MDS42 DNA, complete genome. |
| 766 | NC_020518 | 3499665 | 3499700 | CCCTCGCCCCTTTGGGGAGAGGGTTAGGGAGAGGGG | Escherichia coli str. K-12 substr. MDS42 DNA, complete genome. |
| 767 | NC_020518 | 3743387 | 3743422 | CCCTCGCCCCTCTGGGGAGAGGGTTAGGGTGAGGGG | Escherichia coli str. K-12 substr. MDS42 DNA, complete genome. |
| 768 | NC_020518 | 3925949 | 3925914 | CCCTCGCCCCTCCGGGGAGAGGGTTAGGGTGAGGGG | Escherichia coli str. K-12 substr. MDS42 DNA, complete genome. |
| 769 | NC_022364 | 68411 | 68376 | CCCTCGCCCCTTTGGGGAGAGGGCCGGGGTGAGGGG | Escherichia coli LY180, complete genome. |
| 770 | NC_022364 | 165903 | 165936 | CCCTCTCCCTGTGGGAGAGGGCCGGGGTGAGGGC | Escherichia coli LY180, complete genome. |
| 771 | NC_022364 | 165987 | 165953 | CCCTCTCCCTTGAGGGAGAGGGTTAGGGTGAGGGT | Escherichia coli LY180, complete genome. |
| 772 | NC_022364 | 332126 | 332093 | CCCTCTCCCTGTGGGAGAGGGTCGGGGTGAGGGC | Escherichia coli LY180, complete genome. |
| 773 | NC_022364 | 332167 | 332202 | CCCTCGCCCCTTTGGGGAGAGGGTTAGGGTGAGGGG | Escherichia coli LY180, complete genome. |
| 774 | NC_022364 | 339329 | 339296 | CCCTCTCCCTGTGGGAGAGGGCCGGGGTGAGGGC | Escherichia coli LY180, complete genome. |
| 775 | NC_022364 | 468623 | 468658 | CCCTCGCCCCCTTGGGGAGAGGGTTAGGGTGAGGGG | Escherichia coli LY180, complete genome. |
| 776 | NC_022364 | 569672 | 569707 | CCCTCGCCCCCTCGGGGAGAGGGTTAGGGTGAGGGG | Escherichia coli LY180, complete genome. |
| 777 | NC_022364 | 689040 | 689075 | CCCTCGCCCCTTTGGGGAGAGGGTTAGGGTGAGGGG | Escherichia coli LY180, complete genome. |
| 778 | NC_022364 | 689137 | 689102 | CCCTCTCCCTTCCAGGGTGAGGGCTGGGGTGAGGGT | Escherichia coli LY180, complete genome. |
| 779 | NC_022364 | 696547 | 696512 | CCCTCGCCCCATCGGGGAGAGGGTTAGGGTGAGGGG | Escherichia coli LY180, complete genome. |
| 780 | NC_022364 | 2875157 | 2875122 | CCCTCGCCCCTTTGGGGAGAGGGTTAGGGTGAGGGG | Escherichia coli LY180, complete genome. |
| 781 | NC_022364 | 3148117 | 3148152 | CCCTCGCCCTTTCAGGGAGAGGGCCGGGGTGAGGGT | Escherichia coli LY180, complete genome. |
| 782 | NC_022364 | 3148215 | 3148180 | CCCTCACCCCTTTGGGGAGAGGGTTAGGGTGAGGGG | Escherichia coli LY180, complete genome. |
| 783 | NC_022364 | 3322050 | 3322085 | CCCTCGCCCCTTCGGGGAGAGGGTTAGGGTGAGGGG | Escherichia coli LY180, complete genome. |
| 784 | NC_022364 | 3359363 | 3359398 | CCCTCTCCCTTCCAGGGAGAGGGTCGGGGTGAGGGT | Escherichia coli LY180, complete genome. |
| 785 | NC_022364 | 3359462 | 3359426 | CCCTCGCCCCGTTTGGGGAGAGGGTTAGGGTGAGGGG | Escherichia coli LY180, complete genome. |
| 786 | NC_022364 | 3512325 | 3512290 | CCCTCGCCCCTTTGGGGAGAGGGTTAGGGTGAGGGG | Escherichia coli LY180, complete genome. |
| 787 | NC_022364 | 3611502 | 3611467 | CCCTCGCCCCTATGGGGAGAGGGTTAGGGTGAGGGG | Escherichia coli LY180, complete genome. |
| 788 | NC_022364 | 3782025 | 3781990 | CCCTCTCCCTTTCAGGGAGAGGGCTGGGGTGAGGGT | Escherichia coli LY180, complete genome. |
| 789 | NC_022364 | 3782126 | 3782161 | CCCTCGCCCCTTTGGGGAGAGGGTTAGGGTGAGGGG | Escherichia coli LY180, complete genome. |
| 790 | NC_022364 | 3992714 | 3992679 | CCCTCGCCCCTTTGGGGAGAGGGCTAGGGTGAGGGG | Escherichia coli LY180, complete genome. |
| 791 | NC_022364 | 4100686 | 4100653 | CCCTCTCCCTGTGGGAGAGGGTCGGGGTGAGGGC | Escherichia coli LY180, complete genome. |
| 792 | NC_022364 | 4214054 | 4214089 | CCCTCGCCCCTTTGGGGAGAGGGTTAGGGTGAGGGG | Escherichia coli LY180, complete genome. |
| 793 | NC_022364 | 4239504 | 4239469 | CCCTCGCCCCTACGGGGAGAGGGTTAGGGTGAGGGG | Escherichia coli LY180, complete genome. |
| 794 | NC_022364 | 4239557 | 4239592 | CCCTCGCCCTTTCAGGGAGAGGGCCGGGGTGAGGGT | Escherichia coli LY180, complete genome. |
| 795 | NC_022364 | 4505311 | 4505346 | CCCTCGCCCCTCTGGGGAGAGGGTTAGGGTGAGGGG | Escherichia coli LY180, complete genome. |
| 796 | NC_022364 | 4677101 | 4677134 | CCCTCTCCCTTAGGGAGAGGGTTAGGGTGAGGGT | Escherichia coli LY180, complete genome. |
| 797 | NC_022364 | 4681172 | 4681207 | CCCTCGCCCCTTTGGGGAGAGGGTTAGGGTGAGGGG | Escherichia coli LY180, complete genome. |
| 798 | NC_022364 | 4816003 | 4815970 | CCCTCTCCCTGAGGGAGAGGGCCGGGGTGAGGGC | Escherichia coli LY180, complete genome. |
| 799 | NC_022370 | 551016 | 551051 | CCCTCGCCCCTGTGGGGAGAGGGTTAGGGTGAGGGG | Escherichia coli PMV-1 main chromosome, complete genome. |
| 800 | NC_022648 | 2544412 | 2544447 | CCCTCTCCCCTTGCGGGAGAGGGGACGGGTGAGGGG | Escherichia coli JJ1886, complete genome. |
| 801 | NZ_AGTD01000001 | 167993 | 168026 | CCCTCTCCCTGTGGGAGAGGGCCGGGGTGAGGGC | Escherichia coli UMNF18 chromosome, whole genome shotgun sequence. |
| 802 | NZ_AGTD01000001 | 168077 | 168043 | CCCTCTCCCTTGAGGGAGAGGGTTAGGGTGAGGGT | Escherichia coli UMNF18 chromosome, whole genome shotgun sequence. |
| 803 | NZ_AGTD01000001 | 368581 | 368616 | CCCTCGCCCCCTTGGGGAGAGGGTTAGGGTGAGGGG | Escherichia coli UMNF18 chromosome, whole genome shotgun sequence. |
| 804 | NZ_AGTD01000001 | 476396 | 476431 | CCCTCGCCCCCTCGGGGAGAGGGTTAGGGTGAGGGG | Escherichia coli UMNF18 chromosome, whole genome shotgun sequence. |
| 805 | NZ_AGTD01000001 | 611467 | 611502 | CCCTCGCCCCTTTGGGGAGAGGGTTAGGGTGAGGGG | Escherichia coli UMNF18 chromosome, whole genome shotgun sequence. |
| 806 | NZ_AGTD01000001 | 611564 | 611529 | CCCTCTCCCTTCCAGGGTGAGGGCTGGGGTGAGGGT | Escherichia coli UMNF18 chromosome, whole genome shotgun sequence. |
| 807 | NZ_AGTD01000001 | 1623396 | 1623361 | CCCTCGCCCCTCTGGGGAGAGGGTTAGGGTGAGGGG | Escherichia coli UMNF18 chromosome, whole genome shotgun sequence. |
| 808 | NZ_AGTD01000001 | 3535527 | 3535562 | CCCTCGCCCTTTCAGGGAGAGGGCCGGGGTGAGGGT | Escherichia coli UMNF18 chromosome, whole genome shotgun sequence. |
| 809 | NZ_AGTD01000001 | 3535625 | 3535590 | CCCTCGCCCCTTTGGGGAGAGGGTTAGGGTGAGGGG | Escherichia coli UMNF18 chromosome, whole genome shotgun sequence. |
| 810 | NZ_AGTD01000001 | 3786283 | 3786318 | CCCTCTCCCTTCCAGGGAGAGGGTCGGGGTGAGGGT | Escherichia coli UMNF18 chromosome, whole genome shotgun sequence. |
| 811 | NZ_AGTD01000001 | 3786382 | 3786346 | CCCTCGCCCCGTTTGGGGAGAGGGTTAGGGTGAGGGG | Escherichia coli UMNF18 chromosome, whole genome shotgun sequence. |
| 812 | NZ_AGTD01000001 | 3938207 | 3938172 | CCCTCGCCCCTTTGGGGTGAGGGTTAGGGTGAGGGG | Escherichia coli UMNF18 chromosome, whole genome shotgun sequence. |
| 813 | NZ_AGTD01000001 | 4052237 | 4052202 | CCCTCGCCCCTTTGGGGAGAGGGTTAGGGTGAGGGG | Escherichia coli UMNF18 chromosome, whole genome shotgun sequence. |
| 814 | NZ_AGTD01000001 | 4155251 | 4155286 | CCCTCGCCCCTCTGGGGAGAGGGTTAGGGTGAGGGG | Escherichia coli UMNF18 chromosome, whole genome shotgun sequence. |
| 815 | NZ_AGTD01000001 | 4326260 | 4326227 | CCCTCTCCCTGAGGGAGAGGGTTAGGGTGAGGGG | Escherichia coli UMNF18 chromosome, whole genome shotgun sequence. |
| 816 | NZ_AGTD01000001 | 4326320 | 4326355 | CCCTCGCCCCTCCGGGGAGAGGGCCGGGGTGAGGGG | Escherichia coli UMNF18 chromosome, whole genome shotgun sequence. |
| 817 | NZ_AGTD01000001 | 4448314 | 4448349 | CCCTCGCCCCTCTGGGGAGAGGGTTAGGGTGAGGGG | Escherichia coli UMNF18 chromosome, whole genome shotgun sequence. |
| 818 | NZ_AGTD01000001 | 4502965 | 4502932 | CCCTCTCCCTGTGGGAGAGGGTCGGGGTGAGGGC | Escherichia coli UMNF18 chromosome, whole genome shotgun sequence. |
| 819 | NZ_AGTD01000001 | 4613834 | 4613869 | CCCTCGCCCCTTTGGGGAGAGGGTTAGGGAGAGGGG | Escherichia coli UMNF18 chromosome, whole genome shotgun sequence. |
| 820 | NZ_AGTD01000001 | 4858979 | 4859014 | CCCTCGCCCCTCTGGGGAGAGGGTTAGGGTGAGGGG | Escherichia coli UMNF18 chromosome, whole genome shotgun sequence. |
| 821 | NZ_AGTD01000001 | 5075028 | 5075063 | CCCTCGCCCCTTTGGGGAGAGGGTTAGGGTGAGGGG | Escherichia coli UMNF18 chromosome, whole genome shotgun sequence. |
| 822 | NZ_AGTD01000001 | 5149171 | 5149136 | CCCTCGCCCCTCCGGGGAGAGGGTTAGGGTGAGGGG | Escherichia coli UMNF18 chromosome, whole genome shotgun sequence. |
| 823 | NZ_AGTD01000001 | 5219449 | 5219416 | CCCTCTCCCTGTGGGAGAGGGCCGGGGTGAGGGC | Escherichia coli UMNF18 chromosome, whole genome shotgun sequence. |

**Table S9.** Oligonucleotide sequences used for CD, thermal denaturation and NMR studies.

| name | sequence 5' to 3' |
| --- | --- |
| type A | CCCTCTCCCTTGAGGGAGAGGGTTAGGGTGAGGGT |
| type A mm | CCCTCGCCCCTTTGGGGAGAGGGTTAGGGTGAGGGG |
| type B | CCCTCTCCCTTCCAGGGAGAGGGTCGGGGTGAGGGT |
| type B mm | CCCTCGCCCCTTTGGGGAGAGGGCCGGGGTGAGGGG |
| control | CCCTCGCCCCTTTGCCGAGAGCGTTAGCGTGAGCGG |

**Table S10.** Percentage of potential class I and II triplexes in correlation to G/C content of the genome.

| Genome/Plasmid name | Sum of triplexes per genome/plasmid | G/C content | % class I and II triplexes |
| --- | --- | --- | --- |
| >gi|307069503|ref|NC_014497.1| Candidatus Zinderia insecticola CARI chromosome, complete genome | 2055 | 13.54% | 85.64 |
| >gi|400403787|ref|NC_018414.1| Candidatus Carsonella ruddii CE isolate Thao2000 chromosome, complete genome | 3397 | 13.98% | 83.75 |
| >gi|400404263|ref|NC_018416.1| Candidatus Carsonella ruddii HC isolate Thao2000 chromosome, complete genome | 3340 | 14.18% | 83.62 |
| >gi|400404025|ref|NC_018415.1| Candidatus Carsonella ruddii CS isolate Thao2000 chromosome, complete genome | 3575 | 14.18% | 82.35 |
| >gi|400404501|ref|NC_018417.1| Candidatus Carsonella ruddii HT isolate Thao2000 chromosome, complete genome | 2925 | 14.55% | 85.30 |
| >gi|400404723|ref|NC_018418.1| Candidatus Carsonella ruddii PC isolate NHV chromosome, complete genome | 2396 | 15.63% | 83.81 |
| >gi|386858811|ref|NC_017811.1| Borrelia crocidurae str. Achema plasmid unnamed, complete sequence | 11 | 16.30% | 90.91 |
| >gi|116334902|ref|NC_008512.1| Candidatus Carsonella ruddii PV, complete genome | 2223 | 16.56% | 83.58 |
| >gi|529067324|ref|NC_021919.1| Candidatus Nasuia deltocephalinicola str. NAS-ALF, complete genome | 1375 | 17.13% | 85.38 |
| >gi|386860091|ref|NC_017815.1| Borrelia crocidurae str. Achema plasmid unnamed, complete sequence | 7 | 17.32% | 85.71 |
| >gi|527324386|ref|NC_021894.1| Candidatus Carsonella ruddii DC, complete genome | 2650 | 17.63% | 85.96 |
| >gi|386858678|ref|NC_017783.1| Borrelia crocidurae str. Achema plasmid unnamed, complete sequence | 9 | 19.08% | 88.89 |
| >gi|386858817|ref|NC_017812.1| Borrelia crocidurae str. Achema plasmid unnamed, complete sequence | 13 | 19.32% | 100.00 |
| >gi|116514950|ref|NC_008513.1| Buchnera aphidicola str. Cc (Cinara cedri), complete genome | 3478 | 20.10% | 87.87 |
| >gi|549734475|ref|NC_022551.1| Blattabacterium sp. (Nauphoeta cinerea) plasmid, complete sequence | 36 | 20.60% | 91.67 |
| >gi|386858667|ref|NC_017782.1| Borrelia crocidurae str. Achema plasmid unnamed, complete sequence | 11 | 20.64% | 90.91 |
| >gi|365823329|ref|NC_000955.2| Borrelia burgdorferi B31 plasmid lp21, complete sequence | 23 | 20.65% | 91.30 |
| >gi|268687599|ref|NC_013516.1| Streptobacillus moniliformis DSM 12112 plasmid pSMON01, complete sequence | 28 | 20.87% | 89.29 |
| >gi|384207492|ref|NC_017242.1| Brachyspira intermedia PWS/A plasmid pInt, complete sequence | 7 | 21.01% | 85.71 |
| >gi|307128504|ref|NC_014499.1| Candidatus Sulcia muelleri CARI chromosome, complete genome | 2140 | 21.13% | 85.61 |
| >gi|194246403|ref|NC_011047.1| Candidatus Phytoplasma mali chromosome, complete genome | 3206 | 21.39% | 88.08 |
| >gi|85057963|ref|NC_007719.1| Aster yellows witches'-broom phytoplasma AYWB plasmid pAYWB-III, complete sequence | 19 | 21.77% | 94.74 |
| >gi|225350699|ref|NC_012226.1| Brachyspira hyodysenteriae WA1 plasmid pBHWA1, complete sequence | 74 | 21.82% | 87.84 |
| >gi|386859015|ref|NC_017822.1| Borrelia crocidurae str. Achema plasmid unnamed, complete sequence | 11 | 21.93% | 72.73 |
| >gi|19225058|ref|NC_003425.1| Wigglesworthia glossinidia endosymbiont of Glossina brevipalpis plasmid pWb1, complete sequence | 35 | 21.97% | 80.00 |
| >gi|161833634|ref|NC_010118.1| Candidatus Sulcia muelleri GWSS, complete genome | 1449 | 22.44% | 87.51 |
| >gi|32490749|ref|NC_004344.2| Wigglesworthia glossinidia endosymbiont of Glossina brevipalpis chromosome, complete genome | 4140 | 22.48% | 88.77 |
| >gi|293977746|ref|NC_014004.1| Candidatus Sulcia muelleri DMIN chromosome, complete genome | 1424 | 22.49% | 87.08 |
| >gi|386859010|ref|NC_017819.1| Borrelia crocidurae str. Achema plasmid unnamed, complete sequence | 9 | 22.57% | 77.78 |
| >gi|256370581|ref|NC_013123.1| Candidatus Sulcia muelleri SMDSEM, complete genome | 2476 | 22.60% | 86.11 |
| >gi|387826532|ref|NC_017413.1| Borrelia burgdorferi JD1 plasmid JD1 lp38, complete sequence | 62 | 22.89% | 90.32 |
| >gi|218875581|ref|NC_011782.1| Borrelia burgdorferi ZS7 plasmid ZS7_lp17, complete sequence | 52 | 22.92% | 92.31 |
| >gi|386859192|ref|NC_017780.1| Borrelia crocidurae str. Achema plasmid unnamed, complete sequence | 15 | 22.99% | 93.33 |
| >gi|525905724|ref|NC_021832.1| Spiroplasma taiwanense CT-1 plasmid, complete sequence | 23 | 23.01% | 86.96 |
| >gi|336233113|ref|NC_015662.1| Buchnera aphidicola (Cinara tujafilina) chromosome, complete genome | 2239 | 23.03% | 90.00 |
| >gi|386858972|ref|NC_017817.1| Borrelia crocidurae str. Achema plasmid unnamed, complete sequence | 28 | 23.06% | 82.14 |
| >gi|365823332|ref|NC_001849.2| Borrelia burgdorferi B31 plasmid lp17, complete sequence | 44 | 23.12% | 90.91 |
| >gi|294679245|ref|NT_167352.1| Borrelia afzelii PKo clone fragment c | 24 | 23.13% | 87.50 |
| >gi|386858845|ref|NC_017801.1| Borrelia crocidurae str. Achema plasmid unnamed, complete sequence | 10 | 23.17% | 90.00 |
| >gi|386858806|ref|NC_017802.1| Borrelia crocidurae str. Achema plasmid unnamed, complete sequence | 5 | 23.22% | 100.00 |
| >gi|224796743|ref|NC_012180.1| Borrelia valaisiana VS116 plasmid VS116_lp17, complete sequence | 57 | 23.23% | 92.98 |
| >gi|387827856|ref|NC_017420.1| Borrelia burgdorferi N40 plasmid N40_lp25, complete sequence | 68 | 23.26% | 85.29 |
| >gi|219405371|ref|NC_011783.1| Borrelia burgdorferi ZS7 plasmid ZS7_lp25, complete sequence | 72 | 23.34% | 84.72 |
| >gi|11496607|ref|NC_001850.1| Borrelia burgdorferi B31 plasmid lp25, complete sequence | 73 | 23.34% | 86.30 |
| >gi|384206319|ref|NC_017234.1| Borrelia afzelii PKo plasmid lp28-4, complete sequence | 63 | 23.36% | 90.48 |
| >gi|224586532|ref|NC_012130.1| Borrelia valaisiana VS116 plasmid VS116_cp9, complete sequence | 48 | 23.43% | 81.25 |
| >gi|384207453|ref|NC_017237.1| Borrelia afzelii PKo plasmid lp38, complete sequence | 99 | 23.54% | 93.94 |
| >gi|117621706|ref|NC_008566.1| Borrelia afzelii PKo plasmid lp34, complete sequence | 97 | 23.55% | 94.85 |
| >gi|386860119|ref|NC_017820.1| Borrelia crocidurae str. Achema plasmid unnamed, complete sequence | 17 | 23.60% | 88.24 |
| >gi|343128166|ref|NC_015922.1| Borrelia bissettii DN127 plasmid lp25, complete sequence | 76 | 23.60% | 85.53 |
| >gi|386858793|ref|NC_017798.1| Borrelia crocidurae str. Achema plasmid unnamed, complete sequence | 8 | 23.62% | 87.50 |
| >gi|392388518|ref|NC_017521.1| Mycoplasma leachii 99/014/6, complete genome | 1784 | 23.67% | 89.29 |
| >gi|11497048|ref|NC_001904.1| Borrelia burgdorferi B31 plasmid cp9, complete sequence | 42 | 23.69% | 80.95 |
| >gi|313664890|ref|NC_014751.1| Mycoplasma leachii PG50 chromosome, complete genome | 1747 | 23.75% | 89.58 |
| >gi|83319253|ref|NC_007633.1| Mycoplasma capricolum subsp. capricolum ATCC 27343 chromosome, complete genome | 1806 | 23.77% | 89.20 |
| >gi|11497445|ref|NC_000957.1| Borrelia burgdorferi B31 plasmid lp5, complete sequence | 11 | 23.81% | 90.91 |
| >gi|331703020|ref|NC_015431.1| Mycoplasma mycoides subsp. capri LC str. 95010, complete genome | 1832 | 23.82% | 90.56 |
| >gi|374290092|ref|NC_016621.1| Blattabacterium sp. (Cryptocercus punctulatus) str. Cpu chromosome, complete genome | 4051 | 23.85% | 85.53 |
| >gi|525938956|ref|NC_021846.1| Spiroplasma taiwanense CT-1, complete genome | 2899 | 23.87% | 88.86 |
| >gi|387826721|ref|NC_017406.1| Borrelia burgdorferi JD1 plasmid JD1 lp28-5, complete sequence | 44 | 23.88% | 88.64 |
| >gi|386858754|ref|NC_017788.1| Borrelia crocidurae str. Achema plasmid unnamed, complete sequence | 4 | 23.89% | 75.00 |
| >gi|386859044|ref|NC_017778.1| Borrelia crocidurae str. Achema plasmid unnamed, complete sequence | 184 | 23.92% | 92.93 |
| >gi|527317804|ref|NC_021886.1| Syncytium symbiont of Diaphorina citri plasmid, complete sequence | 42 | 23.93% | 88.10 |
| >gi|387826736|ref|NC_017410.1| Borrelia burgdorferi JD1 plasmid JD1 lp17, complete sequence | 56 | 23.94% | 89.29 |
| >gi|85057958|ref|NC_007718.1| Aster yellows witches'-broom phytoplasma AYWB plasmid pAYWB-II, complete sequence | 4 | 23.95% | 75.00 |
| >gi|479183780|ref|NC_021025.1| Mycoplasma mycoides subsp. mycoides SC str. Gladysdale MU clone SC5, complete genome | 1808 | 23.95% | 90.82 |
| >gi|127763381|ref|NC_005364.2| Mycoplasma mycoides subsp. mycoides SC str. PG1 chromosome, complete genome | 1836 | 23.97% | 91.01 |
| >gi|529057754|ref|NC_021916.1| Candidatus Sulcia muelleri str. Sulcia-ALF, complete genome | 926 | 24.00% | 89.31 |
| >gi|384206380|ref|NC_017232.1| Borrelia afzelii PKo plasmid lp17, complete sequence | 60 | 24.01% | 93.33 |
| >gi|343128141|ref|NC_015916.1| Borrelia bissettii DN127 plasmid lp28-3, complete sequence | 84 | 24.02% | 90.48 |
| >gi|557606926|ref|NC_022778.1| Clostridium tetani 12124569, plasmid p12124569, complete sequence | 63 | 24.04% | 93.65 |
| >gi|229599876|ref|NC_012657.1| Clostridium botulinum Ba4 str. 657 plasmid pCLJ2, complete sequence | 11 | 24.07% | 81.82 |
| >gi|384228035|ref|NC_017259.1| Buchnera aphidicola str. Ua (Uroleucon ambrosiae) chromosome, complete genome | 2075 | 24.14% | 90.70 |
| >gi|386858858|ref|NC_017775.1| Borrelia crocidurae str. Achema plasmid unnamed, complete sequence | 18 | 24.17% | 88.89 |
| >gi|384206338|ref|NC_017239.1| Borrelia afzelii PKo plasmid lp28-2, complete sequence | 70 | 24.24% | 85.71 |
| >gi|527317437|ref|NC_021885.1| Syncytium symbiont of Diaphorina citri, complete genome | 1702 | 24.24% | 89.01 |
| >gi|387826896|ref|NC_017399.1| Borrelia burgdorferi N40 plasmid N40_cp9, complete sequence | 47 | 24.27% | 87.23 |
| >gi|387827839|ref|NC_017417.1| Borrelia burgdorferi N40 plasmid N40_lp17, complete sequence | 53 | 24.28% | 88.68 |
| >gi|85057971|ref|NC_007720.1| Aster yellows witches'-broom phytoplasma AYWB plasmid pAYWB-IV, complete sequence | 6 | 24.35% | 83.33 |
| >gi|384206297|ref|NC_017233.1| Borrelia afzelii PKo plasmid lp28-3, complete sequence | 59 | 24.36% | 89.83 |
| >gi|117621646|ref|NC_008565.1| Borrelia afzelii PKo plasmid lp60-2, complete sequence | 138 | 24.38% | 89.13 |
| >gi|386858648|ref|NC_017821.1| Borrelia crocidurae str. Achema plasmid unnamed, complete sequence | 5 | 24.38% | 80.00 |
| >gi|386858865|ref|NC_017787.1| Borrelia crocidurae str. Achema plasmid unnamed, complete sequence | 45 | 24.40% | 82.22 |
| >gi|218906779|ref|NC_011785.1| Borrelia burgdorferi ZS7 plasmid ZS7_lp28-4, complete sequence | 62 | 24.40% | 91.94 |
| >gi|343127105|ref|NC_015911.1| Borrelia bissettii DN127 plasmid cp9, complete sequence | 43 | 24.41% | 93.02 |
| >gi|224984009|ref|NC_012166.1| Borrelia valaisiana VS116 plasmid VS116_lp25, complete sequence | 105 | 24.44% | 85.71 |
| >gi|224985707|ref|NC_012204.1| Borrelia valaisiana VS116 plasmid VS116_lp36, complete sequence | 65 | 24.45% | 89.23 |
| >gi|291280551|ref|NC_013940.1| Deferribacter desulfuricans SSM1 megaplasmid pDF308, complete sequence | 426 | 24.46% | 91.31 |
| >gi|11496735|ref|NC_001854.1| Borrelia burgdorferi B31 plasmid lp28-4, complete sequence | 51 | 24.53% | 96.08 |
| >gi|28373131|ref|NC_004565.1| Clostridium tetani E88 plasmid pE88, complete sequence | 71 | 24.53% | 97.18 |
| >gi|148557803|ref|NC_009506.1| Fusobacterium nucleatum subsp. polymorphum ATCC 10953 plasmid pFN3, complete sequence | 24 | 24.53% | 87.50 |
| >gi|343127118|ref|NC_015915.1| Borrelia bissettii DN127 plasmid lp17, complete sequence | 70 | 24.58% | 92.86 |
| >gi|387828029|ref|NC_017416.1| Borrelia burgdorferi N40 plasmid N40_lp28-4, complete sequence | 58 | 24.62% | 91.38 |
| >gi|117621743|ref|NC_008567.1| Borrelia afzelii PKo plasmid lp32, complete sequence | 68 | 24.67% | 91.18 |
| >gi|387826817|ref|NC_017407.1| Borrelia burgdorferi JD1 plasmid JD1 lp28-4, complete sequence | 57 | 24.67% | 91.23 |
| >gi|472328233|ref|NC_020831.1| Candidatus Portiera aleyrodidarum TV, complete genome | 514 | 24.69% | 91.63 |
| >gi|386858961|ref|NC_017813.1| Borrelia crocidurae str. Achema plasmid unnamed, complete sequence | 5 | 24.70% | 80.00 |
| >gi|343127152|ref|NC_015917.1| Borrelia bissettii DN127 plasmid lp28-4, complete sequence | 48 | 24.77% | 87.50 |
| >gi|384207328|ref|NC_017240.1| Borrelia afzelii PKo plasmid lp32-10, complete sequence | 59 | 24.78% | 89.83 |
| >gi|386859201|ref|NC_017797.1| Borrelia crocidurae str. Achema plasmid unnamed, complete sequence | 34 | 24.92% | 88.24 |
| >gi|47458835|ref|NC_006908.1| Mycoplasma mobile 163K, complete genome | 2205 | 24.95% | 89.66 |
| >gi|187935795|ref|NC_010680.1| Clostridium botulinum B str. Eklund 17B plasmid pCLL, complete sequence | 46 | 24.95% | 89.13 |
| >gi|507382352|ref|NC_021277.1| Fusobacterium sp. 4_8 plasmid, complete sequence | 27 | 24.98% | 96.30 |
| >gi|387826795|ref|NC_017405.1| Borrelia burgdorferi JD1 plasmid JD1 lp28-3, complete sequence | 81 | 24.98% | 90.12 |
| >gi|383843668|ref|NC_017177.1| Clostridium difficile BI1, complete genome | 496 | 25.00% | 89.92 |
| >gi|386858640|ref|NC_017789.1| Borrelia crocidurae str. Achema plasmid unnamed, complete sequence | 1 | 25.02% | 100.00 |
| >gi|11496697|ref|NC_001853.1| Borrelia burgdorferi B31 plasmid lp28-3, complete sequence | 83 | 25.04% | 90.36 |
| >gi|224984639|ref|NC_012185.1| Borrelia valaisiana VS116 plasmid VS116_lp28-3, complete sequence | 221 | 25.06% | 88.24 |
| >gi|218875456|ref|NC_011781.1| Borrelia burgdorferi ZS7 plasmid ZS7_lp28-3, complete sequence | 86 | 25.08% | 89.53 |
| >gi|117621799|ref|NC_008568.1| Borrelia afzelii PKo plasmid lp28, complete sequence | 63 | 25.10% | 95.24 |
| >gi|294679220|ref|NT_167351.1| Borrelia afzelii PKo clone fragment b | 34 | 25.12% | 82.35 |
| >gi|379009272|ref|NC_016893.1| Wigglesworthia glossinidia endosymbiont of Glossina morsitans morsitans (Yale colony) chromosome, complete genome | 2914 | 25.22% | 90.91 |
| >gi|203283903|ref|NC_011224.1| Borrelia duttonii Ly plasmid pl11, complete sequence | 5 | 25.29% | 80.00 |
| >gi|21672294|ref|NC_004061.1| Buchnera aphidicola str. Sg (Schizaphis graminum) chromosome, complete genome | 3143 | 25.33% | 88.20 |
| >gi|387828011|ref|NC_017415.1| Borrelia burgdorferi N40 plasmid N40_lp28-5, complete sequence | 62 | 25.34% | 90.32 |
| >gi|27904513|ref|NC_004545.1| Buchnera aphidicola str. Bp (Baizongia pistaciae) chromosome, complete genome | 1324 | 25.34% | 93.13 |
| >gi|169834533|ref|NC_010379.1| Clostridium botulinum B1 str. Okra plasmid pCLD, complete sequence | 142 | 25.36% | 93.66 |
| >gi|387826517|ref|NC_017409.1| Borrelia burgdorferi JD1 plasmid JD1 lp25, complete sequence | 64 | 25.37% | 87.50 |
| >gi|28191365|ref|NC_004555.1| Buchnera aphidicola str. Bp (Baizongia pistaciae) plasmid pBBp1, complete sequence | 6 | 25.39% | 100.00 |
| >gi|525908085|ref|NC_021833.1| Spiroplasma diminutum CUAS-1, complete genome | 1473 | 25.46% | 89.14 |
| >gi|15081479|ref|NC_003042.1| Clostridium perfringens str. 13 plasmid pCP13, complete sequence | 95 | 25.50% | 87.37 |
| >gi|170761847|ref|NC_010503.1| Ureaplasma parvum serovar 3 str. ATCC 27815 chromosome, complete genome | 1291 | 25.50% | 91.63 |
| >gi|13357558|ref|NC_002162.1| Ureaplasma parvum serovar 3 str. ATCC 700970 chromosome, complete genome | 1286 | 25.50% | 91.68 |
| >gi|224586487|ref|NC_012131.1| Borrelia valaisiana VS116 plasmid VS116_cp32-2-7, complete sequence | 63 | 25.51% | 84.13 |
| >gi|387826840|ref|NC_017411.1| Borrelia burgdorferi JD1 plasmid JD1 lp36, complete sequence | 49 | 25.59% | 89.80 |
| >gi|51038597|ref|NC_006128.1| Borrelia garinii PBi plasmid cp26, complete sequence | 66 | 25.60% | 89.39 |
| >gi|229587271|ref|NC_012654.1| Clostridium botulinum Ba4 str. 657 plasmid pCLJ, complete sequence | 287 | 25.60% | 90.24 |
| >gi|85057952|ref|NC_007717.1| Aster yellows witches'-broom phytoplasma AYWB plasmid pAYWB-I, complete sequence | 7 | 25.60% | 85.71 |
| >gi|384228027|ref|NC_017261.1| Buchnera aphidicola str. Ua (Uroleucon ambrosiae) plasmid pLeu, complete sequence | 30 | 25.63% | 86.67 |
| >gi|169834729|ref|NC_010418.1| Clostridium botulinum A3 str. Loch Maree plasmid pCLK, complete sequence | 268 | 25.64% | 88.81 |
| >gi|384227456|ref|NC_017256.1| Buchnera aphidicola str. Ak (Acyrthosiphon kondoi) chromosome, complete genome | 2497 | 25.66% | 89.55 |
| >gi|387906790|ref|NC_017924.1| Blattabacterium sp. (Blaberus giganteus) chromosome, complete genome | 3333 | 25.67% | 87.22 |
| >gi|224586459|ref|NC_012129.1| Borrelia valaisiana VS116 plasmid VS116_cp26, complete sequence | 67 | 25.68% | 91.04 |
| >gi|433624204|ref|NC_019949.1| Mycoplasma cynos C142 complete genome | 1949 | 25.70% | 86.51 |
| >gi|26553452|ref|NC_004432.1| Mycoplasma penetrans HF-2, complete genome | 2409 | 25.72% | 88.67 |
| >gi|203288364|ref|NC_011252.1| Borrelia recurrentis A1 plasmid pl23, complete sequence | 45 | 25.74% | 88.89 |
| >gi|384227452|ref|NC_017258.1| Buchnera aphidicola str. Ak (Acyrthosiphon kondoi) plasmid pTrp, complete sequence | 21 | 25.76% | 85.71 |
| >gi|209553988|ref|NC_011374.1| Ureaplasma urealyticum serovar 10 str. ATCC 33699 chromosome, complete genome | 1170 | 25.77% | 91.54 |
| >gi|110804009|ref|NC_008264.1| Clostridium perfringens SM101 plasmid pSM101B, complete sequence | 42 | 25.82% | 83.33 |
| >gi|384206192|ref|NC_017229.1| Borrelia afzelii PKo plasmid cp26, complete sequence | 90 | 25.88% | 88.89 |
| >gi|385858114|ref|NC_017519.1| Mycoplasma hyorhinis MCLD chromosome, complete genome | 2355 | 25.88% | 87.81 |
| >gi|304372805|ref|NC_014448.1| Mycoplasma hyorhinis HUB-1 chromosome, complete genome | 2419 | 25.88% | 88.14 |
| >gi|121582657|ref|NC_008770.1| Campylobacter jejuni subsp. jejuni 81-176 plasmid pVir, complete sequence | 169 | 25.89% | 80.47 |
| >gi|111074118|ref|NC_008274.1| Borrelia afzelii PKo plasmid cp27, complete sequence | 90 | 25.89% | 88.89 |
| >gi|423262555|ref|NC_019552.1| Mycoplasma hyorhinis SK76 chromosome, complete genome | 2379 | 25.89% | 87.94 |
| >gi|384448934|ref|NC_017284.1| Campylobacter jejuni subsp. jejuni IA3902 plasmid pVir, complete sequence | 175 | 25.91% | 79.43 |
| >gi|378835506|ref|NC_016829.1| Mycoplasma hyorhinis GDL-1 chromosome, complete genome | 2378 | 25.91% | 88.06 |
| >gi|558671575|ref|NC_022807.1| Mycoplasma hyorhinis DBS 1050, complete genome | 2369 | 25.91% | 87.93 |
| >gi|337738947|ref|NC_015688.1| Clostridium acetobutylicum DSM 1731 plasmid pSMBb, complete sequence | 7 | 25.92% | 100.00 |
| >gi|386854243|ref|NC_017725.1| Borrelia garinii BgVir plasmid cp26, complete sequence | 75 | 25.93% | 88.00 |
| >gi|387827769|ref|NC_017421.1| Borrelia burgdorferi N40 plasmid N40_lp38, complete sequence | 76 | 26.00% | 86.84 |
| >gi|224796528|ref|NC_012169.1| Borrelia valaisiana VS116 plasmid VS116_lp28-8, complete sequence | 49 | 26.04% | 89.80 |
| >gi|11496831|ref|NC_001856.1| Borrelia burgdorferi B31 plasmid lp38, complete sequence | 78 | 26.07% | 87.18 |
| >gi|549683697|ref|NC_022550.1| Blattabacterium sp. (Nauphoeta cinerea), complete genome | 3162 | 26.08% | 87.07 |
| >gi|384463962|ref|NC_017298.1| Clostridium botulinum F str. 230613 plasmid pCBF, complete sequence | 17 | 26.09% | 94.12 |
| >gi|153941546|ref|NC_009700.1| Clostridium botulinum F str. Langeland plasmid pCLI, complete sequence | 17 | 26.09% | 94.12 |
| >gi|203288272|ref|NC_011246.1| Borrelia recurrentis A1 plasmid pl124, complete sequence | 128 | 26.10% | 89.84 |
| >gi|407453019|ref|NC_018618.1| Candidatus Portiera aleyrodidarum BT-QVLC chromosome, complete genome | 440 | 26.12% | 92.05 |
| >gi|331270761|ref|NC_015426.1| Clostridium botulinum BKT015925 plasmid p2BKT015925, complete sequence | 78 | 26.14% | 97.44 |
| >gi|407681354|ref|NC_018676.1| Candidatus Portiera aleyrodidarum BT-QVLC chromosome, complete genome | 433 | 26.16% | 92.38 |
| >gi|402575002|ref|NC_018507.1| Candidatus Portiera aleyrodidarum BT-B chromosome, complete genome | 441 | 26.17% | 92.29 |
| >gi|387825554|ref|NC_017395.1| Borrelia burgdorferi JD1 plasmid JD1 cp26, complete sequence | 68 | 26.18% | 91.18 |
| >gi|407681635|ref|NC_018677.1| Candidatus Portiera aleyrodidarum BT-B-HRs chromosome, complete genome | 432 | 26.21% | 92.59 |
| >gi|331268174|ref|NC_015419.1| Clostridium botulinum BKT015925 plasmid p5BKT015925, complete sequence | 8 | 26.25% | 87.50 |
| >gi|219681389|ref|NC_011833.1| Buchnera aphidicola str. 5A (Acyrthosiphon pisum) chromosome, complete genome | 2448 | 26.29% | 88.97 |
| >gi|384226395|ref|NC_017253.1| Buchnera aphidicola str. JF99 (Acyrthosiphon pisum) chromosome, complete genome | 2461 | 26.29% | 88.99 |
| >gi|219681945|ref|NC_011834.1| Buchnera aphidicola str. Tuc7 (Acyrthosiphon pisum) chromosome, complete genome | 2462 | 26.29% | 88.91 |
| >gi|384226874|ref|NC_017255.1| Buchnera aphidicola str. LL01 (Acyrthosiphon pisum) chromosome, complete genome | 2450 | 26.30% | 89.06 |
| >gi|384225821|ref|NC_017252.1| Buchnera aphidicola str. TLW03 (Acyrthosiphon pisum) chromosome, complete genome | 2451 | 26.30% | 89.07 |
| >gi|11497007|ref|NC_001903.1| Borrelia burgdorferi B31 plasmid cp26, complete sequence | 65 | 26.30% | 92.31 |
| >gi|15616630|ref|NC_002528.1| Buchnera aphidicola str. APS (Acyrthosiphon pisum) chromosome, complete genome | 2470 | 26.31% | 88.91 |
| >gi|269122800|ref|NC_013515.1| Streptobacillus moniliformis DSM 12112 chromosome, complete genome | 1974 | 26.31% | 91.08 |
| >gi|387827984|ref|NC_017401.1| Borrelia burgdorferi N40 plasmid N40_cp26, complete sequence | 73 | 26.32% | 90.41 |
| >gi|384226396|ref|NC_017254.1| Buchnera aphidicola str. JF98 (Acyrthosiphon pisum) chromosome, complete genome | 2439 | 26.32% | 89.18 |
| >gi|218203978|ref|NC_011724.1| Borrelia burgdorferi ZS7 plasmid ZS7_cp26, complete sequence | 66 | 26.33% | 90.91 |
| >gi|203288797|ref|NC_011257.1| Borrelia duttonii Ly plasmid pl23, complete sequence | 40 | 26.35% | 87.50 |
| >gi|343126945|ref|NC_015907.1| Borrelia bissettii DN127 plasmid cp26, complete sequence | 90 | 26.39% | 90.00 |
| >gi|386858849|ref|NC_017774.1| Borrelia crocidurae str. Achema plasmid unnamed, complete sequence | 4 | 26.39% | 100.00 |
| >gi|471331658|ref|NC_020510.1| Blattabacterium sp. (Panesthia angustipennis spadica) str. BPAA DNA, complete genome | 3259 | 26.41% | 87.45 |
| >gi|331270865|ref|NC_015417.1| Clostridium botulinum BKT015925 plasmid p1BKT015925, complete sequence | 128 | 26.45% | 96.09 |
| >gi|51038624|ref|NC_006129.1| Borrelia garinii PBi plasmid lp54, complete sequence | 130 | 26.47% | 91.54 |
| >gi|373231418|ref|NC_016748.1| Marinitoga piezophila KA3 plasmid pMARPI01, complete sequence | 20 | 26.52% | 90.00 |
| >gi|384228016|ref|NC_017257.1| Buchnera aphidicola str. Ak (Acyrthosiphon kondoi) plasmid pLeu, complete sequence | 27 | 26.55% | 92.59 |
| >gi|384170815|ref|NC_017192.1| Arcobacter sp. L, complete genome | 5106 | 26.56% | 90.76 |
| >gi|203288506|ref|NC_011247.1| Borrelia duttonii Ly plasmid pl165, complete sequence | 184 | 26.57% | 92.93 |
| >gi|15828471|ref|NC_002771.1| Mycoplasma pulmonis UAB CTIP, complete genome | 3182 | 26.64% | 86.46 |
| >gi|110803998|ref|NC_008263.1| Clostridium perfringens SM101 plasmid pSM101A, complete sequence | 29 | 26.68% | 75.86 |
| >gi|10957103|ref|NC_002253.1| Buchnera aphidicola str. APS (Acyrthosiphon pisum) plasmid pLeu, complete sequence | 32 | 26.71% | 96.88 |
| >gi|386853317|ref|NC_017804.1| Borrelia garinii BgVir plasmid lp54, complete sequence | 136 | 26.75% | 91.91 |
| >gi|331271088|ref|NC_015418.1| Clostridium botulinum BKT015925 plasmid p3BKT015925, complete sequence | 67 | 26.80% | 100.00 |
| >gi|148381586|ref|NC_009496.1| Clostridium botulinum A str. ATCC 3502 plasmid pBOT3502, complete sequence | 16 | 26.80% | 93.75 |
| >gi|479133239|ref|NC_021002.1| Mycoplasma fermentans PG18 DNA, nearly complete genome | 1819 | 26.82% | 88.73 |
| >gi|336120990|ref|NC_015632.1| Methanothermococcus okinawensis IH1 plasmid pMETOK01, complete sequence | 14 | 26.84% | 92.86 |
| >gi|386858623|ref|NC_017799.1| Borrelia crocidurae str. Achema plasmid unnamed, complete sequence | 4 | 26.84% | 100.00 |
| >gi|319776738|ref|NC_014921.1| Mycoplasma fermentans M64 chromosome, complete genome | 2022 | 26.86% | 88.38 |
| >gi|222778487|ref|NC_012040.1| Campylobacter lari RM2100 megaplasmid pCL2100, complete sequence | 108 | 26.86% | 91.67 |
| >gi|386858760|ref|NC_017796.1| Borrelia crocidurae str. Achema plasmid unnamed, complete sequence | 21 | 26.87% | 95.24 |
| >gi|85057280|ref|NC_007716.1| Aster yellows witches'-broom phytoplasma AYWB, complete genome | 2136 | 26.89% | 87.97 |
| >gi|218868668|ref|NC_011778.1| Borrelia burgdorferi ZS7 plasmid ZS7_lp36, complete sequence | 76 | 26.89% | 89.47 |
| >gi|117621570|ref|NC_008564.1| Borrelia afzelii PKo plasmid lp60, complete sequence | 131 | 26.90% | 91.60 |
| >gi|11496779|ref|NC_001855.1| Borrelia burgdorferi B31 plasmid lp36, complete sequence | 77 | 26.93% | 88.31 |
| >gi|344204770|ref|NC_015946.1| Mycoplasma putrefaciens KS1 chromosome, complete genome | 939 | 26.94% | 91.05 |
| >gi|308189587|ref|NC_014552.1| Mycoplasma fermentans JER chromosome, complete genome | 1826 | 26.95% | 88.77 |
| >gi|294155300|ref|NC_014014.1| Mycoplasma crocodyli MP145 chromosome, complete genome | 1410 | 26.95% | 88.72 |
| >gi|552658063|ref|NC_022583.1| Mesoplasma florum W37, complete genome | 1097 | 26.95% | 90.70 |
| >gi|482887500|ref|NC_021083.1| Mycoplasma putrefaciens Mput9231, complete genome | 963 | 26.96% | 91.17 |
| >gi|550986377|ref|NC_022575.1| Mycoplasma parvum str. Indiana, complete genome | 1805 | 26.98% | 86.20 |
| >gi|387827965|ref|NC_017400.1| Borrelia burgdorferi N40 plasmid N40_cp32-4, complete sequence | 30 | 27.02% | 86.67 |
| >gi|294679205|ref|NT_167350.1| Borrelia afzelii PKo clone fragment a | 29 | 27.02% | 96.55 |
| >gi|50364815|ref|NC_006055.1| Mesoplasma florum L1 chromosome, complete genome | 1074 | 27.02% | 90.69 |
| >gi|384207382|ref|NC_017241.1| Borrelia afzelii PKo plasmid lp54, complete sequence | 125 | 27.02% | 92.00 |
| >gi|157736271|ref|NC_009850.1| Arcobacter butzleri RM4018 chromosome, complete genome | 4013 | 27.05% | 89.98 |
| >gi|526459392|ref|NC_021878.1| Arcobacter butzleri 7h1h, complete genome | 3887 | 27.06% | 90.17 |
| >gi|225618950|ref|NC_012225.1| Brachyspira hyodysenteriae WA1 chromosome, complete genome | 3356 | 27.06% | 90.20 |
| >gi|384154712|ref|NC_017187.1| Arcobacter butzleri ED-1, complete genome | 3826 | 27.07% | 89.99 |
| >gi|386858702|ref|NC_017785.1| Borrelia crocidurae str. Achema plasmid unnamed, complete sequence | 1 | 27.09% | 100.00 |
| >gi|507380240|ref|NC_021281.1| Fusobacterium sp. 4_8, complete genome | 3969 | 27.10% | 89.04 |
| >gi|269114774|ref|NC_013511.1| Mycoplasma hominis ATCC 23114 chromosome, complete genome | 1195 | 27.12% | 88.54 |
| >gi|534508017|ref|NC_022196.1| Fusobacterium nucleatum subsp. vincentii 3_1_36A2, complete genome | 3910 | 27.12% | 88.90 |
| >gi|262340793|ref|NC_013454.1| Blattabacterium sp. (Blattella germanica) str. Bge, complete genome | 3106 | 27.14% | 87.12 |
| >gi|19703352|ref|NC_003454.1| Fusobacterium nucleatum subsp. nucleatum ATCC 25586 chromosome, complete genome | 3685 | 27.15% | 89.04 |
| >gi|387827903|ref|NC_017422.1| Borrelia burgdorferi N40 plasmid N40_cp32-7, complete sequence | 38 | 27.16% | 76.32 |
| >gi|384863363|ref|NC_017344.1| Staphylococcus aureus subsp. aureus ECT-R 2 plasmid pLUH02, complete sequence | 32 | 27.17% | 84.38 |
| >gi|501675884|ref|NC_021236.1| Strawberry lethal yellows phytoplasma (CPA) str. NZSb11, complete genome | 3736 | 27.19% | 86.67 |
| >gi|384207496|ref|NC_017243.1| Brachyspira intermedia PWS/A chromosome, complete genome | 3628 | 27.22% | 90.24 |
| >gi|479330719|ref|NC_021060.1| Staphylococcus aureus M1 plasmid pSK67-M1, complete sequence | 32 | 27.22% | 84.38 |
| >gi|134046898|ref|NC_009136.1| Methanococcus maripaludis C5 plasmid pMMC501, complete sequence | 10 | 27.24% | 90.00 |
| >gi|203288400|ref|NC_011255.1| Borrelia recurrentis A1 plasmid pl35, complete sequence | 29 | 27.27% | 93.10 |
| >gi|219882535|ref|NC_011878.1| Buchnera aphidicola BCc plasmid pLeu-BCc, complete sequence | 26 | 27.29% | 80.77 |
| >gi|11497372|ref|NC_000956.1| Borrelia burgdorferi B31 plasmid lp56, complete sequence | 83 | 27.29% | 85.54 |
| >gi|188587536|ref|NC_010723.1| Clostridium botulinum E3 str. Alaska E43 chromosome, complete genome | 2981 | 27.36% | 91.41 |
| >gi|563706614|ref|NC_023003.1| delta proteobacterium BABL1 complete genome | 1280 | 27.38% | 91.02 |
| >gi|33519483|ref|NC_005061.1| Candidatus Blochmannia floridanus chromosome, complete genome | 776 | 27.38% | 93.17 |
| >gi|197294169|ref|NC_010544.1| Candidatus Phytoplasma australiense, complete genome | 3441 | 27.42% | 85.67 |
| >gi|386858706|ref|NC_017786.1| Borrelia crocidurae str. Achema plasmid unnamed, complete sequence | 40 | 27.42% | 97.50 |
| >gi|224984381|ref|NC_012177.1| Borrelia valaisiana VS116 plasmid VS116_lp54, complete sequence | 115 | 27.44% | 90.43 |
| >gi|434380778|ref|NC_018604.1| Brachyspira pilosicoli WesB complete genome | 3217 | 27.45% | 89.74 |
| >gi|343126844|ref|NC_015906.1| Borrelia bissettii DN127 plasmid cp32-quad, complete sequence | 145 | 27.47% | 85.52 |
| >gi|386858652|ref|NC_017779.1| Borrelia crocidurae str. Achema plasmid unnamed, complete sequence | 3 | 27.48% | 66.67 |
| >gi|57238731|ref|NC_005295.2| Ehrlichia ruminantium str. Welgevonden chromosome, complete genome | 878 | 27.48% | 94.65 |
| >gi|58578664|ref|NC_006832.1| Ehrlichia ruminantium str. Welgevonden, complete genome | 877 | 27.48% | 94.64 |
| >gi|357420708|ref|NC_016146.1| Blattabacterium sp. (Mastotermes darwiniensis) str. MADAR chromosome, complete genome | 2390 | 27.48% | 86.61 |
| >gi|365823447|ref|NC_014909.2| Candidatus Blochmannia vafer str. BVAF chromosome, complete genome | 949 | 27.51% | 93.47 |
| >gi|187932320|ref|NC_010674.1| Clostridium botulinum B str. Eklund 17B chromosome, complete genome | 3139 | 27.51% | 91.84 |
| >gi|58616727|ref|NC_006831.1| Ehrlichia ruminantium str. Gardel, complete genome | 894 | 27.51% | 94.63 |
| >gi|203287471|ref|NC_011244.1| Borrelia recurrentis A1, complete genome | 1301 | 27.52% | 91.01 |
| >gi|343127183|ref|NC_015918.1| Borrelia bissettii DN127 plasmid lp28-7, complete sequence | 51 | 27.53% | 90.20 |
| >gi|384228024|ref|NC_017260.1| Buchnera aphidicola str. Ua (Uroleucon ambrosiae) plasmid pTrp, complete sequence | 4 | 27.54% | 100.00 |
| >gi|386859226|ref|NC_017808.1| Borrelia crocidurae str. Achema chromosome, complete genome | 1297 | 27.55% | 91.13 |
| >gi|203283926|ref|NC_011229.1| Borrelia duttonii Ly, complete genome | 1284 | 27.59% | 90.73 |
| >gi|554085530|ref|NC_022588.1| strain 284/09 Stolbur phytoplasma draft | 1617 | 27.60% | 88.37 |
| >gi|84488831|ref|NC_007681.1| Methanosphaera stadtmanae DSM 3091 chromosome, complete genome | 2082 | 27.63% | 89.82 |
| >gi|386858626|ref|NC_017781.1| Borrelia crocidurae str. Achema plasmid unnamed, complete sequence | 3 | 27.74% | 100.00 |
| >gi|296125058|ref|NC_014150.1| Brachyspira murdochii DSM 12563 chromosome, complete genome | 3887 | 27.75% | 90.76 |
| >gi|255961248|ref|NC_005303.2| Onion yellows phytoplasma OY-M, complete genome | 2077 | 27.76% | 89.65 |
| >gi|404474675|ref|NC_018607.1| Brachyspira pilosicoli B2904 chromosome, complete genome | 3052 | 27.79% | 89.78 |
| >gi|203288867|ref|NC_011262.1| Borrelia duttonii Ly plasmid pl31, complete sequence | 28 | 27.82% | 92.86 |
| >gi|300869639|ref|NC_014330.1| Brachyspira pilosicoli 95/1000 chromosome, complete genome | 2798 | 27.90% | 89.71 |
| >gi|110666922|ref|NC_008226.1| Peptoclostridium difficile 630 plasmid pCD630, complete sequence | 6 | 27.90% | 100.00 |
| >gi|449338574|ref|NC_020264.1| Staphylococcus warneri SG1 plasmid clone pvSw2 genomic sequence | 9 | 27.90% | 100.00 |
| >gi|431806727|ref|NC_019908.1| Brachyspira pilosicoli P43/6/78 chromosome, complete genome | 2844 | 27.92% | 89.38 |
| >gi|218202726|ref|NC_011720.1| Borrelia burgdorferi ZS7 plasmid ZS7_cp32-3+10, complete sequence | 113 | 27.97% | 83.19 |
| >gi|347541852|ref|NC_016012.1| Candidatus Arthromitus sp. SFB-rat-Yit, complete genome | 1173 | 27.98% | 92.75 |
| >gi|32470555|ref|NC_005005.1| Staphylococcus epidermidis ATCC 12228 plasmid pSE-12228-04, complete sequence | 11 | 27.99% | 81.82 |
| >gi|383843667|ref|NC_017176.1| Clostridium difficile BI1 plasmid pCDBI1, complete sequence | 30 | 28.03% | 90.00 |
| >gi|387827926|ref|NC_017423.1| Borrelia burgdorferi N40 plasmid N40_cp32-12, complete sequence | 59 | 28.07% | 86.44 |
| >gi|203283912|ref|NC_011226.1| Borrelia duttonii Ly plasmid pl15, complete sequence | 11 | 28.09% | 90.91 |
| >gi|331270707|ref|NC_015427.1| Clostridium botulinum BKT015925 plasmid p4BKT015925, complete sequence | 25 | 28.10% | 88.00 |
| >gi|384455125|ref|NC_017294.1| Candidatus Arthromitus sp. SFB-mouse-Yit, complete genome | 1073 | 28.11% | 91.99 |
| >gi|110804020|ref|NC_008265.1| Clostridium phage phiSM101, complete genome | 39 | 28.13% | 94.87 |
| >gi|386644995|ref|NC_017776.1| Borrelia crocidurae str. Achema plasmid unnamed, complete sequence | 2 | 28.13% | 100.00 |
| >gi|256055273|ref|NC_013130.1| Borrelia burgdorferi N40 plasmid N40_lp54, complete sequence | 101 | 28.14% | 87.13 |
| >gi|365823346|ref|NC_001857.2| Borrelia burgdorferi B31 plasmid lp54, complete sequence | 100 | 28.14% | 89.00 |
| >gi|219499124|ref|NC_011784.1| Borrelia burgdorferi ZS7 plasmid ZS7_lp54, complete sequence | 98 | 28.15% | 87.76 |
| >gi|222142561|ref|NC_011995.1| Macrococcus caseolyticus JCSC5402 plasmid pMCCL1, complete sequence | 34 | 28.18% | 82.35 |
| >gi|153934468|ref|NC_009698.1| Clostridium botulinum A str. Hall chromosome, complete genome | 3289 | 28.18% | 92.09 |
| >gi|444335299|ref|NC_020195.1| Blattabacterium sp. (Blatta orientalis) str. Tarazona, complete genome | 2747 | 28.19% | 86.20 |
| >gi|268793183|ref|NC_013518.1| Sebaldella termitidis ATCC 33386 plasmid pSTERM01, complete sequence | 90 | 28.19% | 88.89 |
| >gi|10954488|ref|NC_001732.1| Methanocaldococcus jannaschii DSM 2661 plasmid large ECE, complete sequence | 40 | 28.19% | 92.50 |
| >gi|256041951|ref|NC_013129.1| Borrelia burgdorferi JD1 plasmid JD1_lp54, complete sequence | 104 | 28.20% | 88.46 |
| >gi|384868560|ref|NC_017345.1| Staphylococcus aureus subsp. aureus TCH60 plasmid unnamed, complete sequence | 28 | 28.20% | 82.14 |
| >gi|153930785|ref|NC_009697.1| Clostridium botulinum A str. ATCC 19397 chromosome, complete genome | 3364 | 28.21% | 92.03 |
| >gi|387816237|ref|NC_017299.1| Clostridium botulinum H04402 065, complete genome | 3394 | 28.21% | 92.58 |
| >gi|295656029|ref|NC_013418.2| Blattabacterium sp. (Periplaneta americana) str. BPLAN, complete genome | 2838 | 28.21% | 85.52 |
| >gi|226947222|ref|NC_012563.1| Clostridium botulinum A2 str. Kyoto chromosome, complete genome | 3577 | 28.21% | 92.12 |
| >gi|237793320|ref|NC_012658.1| Clostridium botulinum Ba4 str. 657 chromosome, complete genome | 3476 | 28.22% | 91.37 |
| >gi|148378011|ref|NC_009495.1| Clostridium botulinum A str. ATCC 3502 chromosome, complete genome | 3395 | 28.24% | 91.90 |
| >gi|110801439|ref|NC_008262.1| Clostridium perfringens SM101 chromosome, complete genome | 3114 | 28.25% | 90.85 |
| >gi|342731686|ref|NC_015913.1| Candidatus Arthromitus sp. SFB-mouse-Japan, complete genome | 1084 | 28.26% | 91.97 |
| >gi|384460459|ref|NC_017297.1| Clostridium botulinum F str. 230613 chromosome, complete genome | 3428 | 28.30% | 91.66 |
| >gi|563693166|ref|NC_022998.1| Spiroplasma apis B31, complete genome | 1585 | 28.30% | 90.03 |
| >gi|51598263|ref|NC_006156.1| Borrelia garinii PBi chromosome linear, complete sequence | 1831 | 28.30% | 89.40 |
| >gi|384206503|ref|NC_017238.1| Borrelia afzelii PKo chromosome, complete genome | 1854 | 28.31% | 90.18 |
| >gi|153937894|ref|NC_009699.1| Clostridium botulinum F str. Langeland chromosome, complete genome | 3425 | 28.31% | 91.74 |
| >gi|170758191|ref|NC_010520.1| Clostridium botulinum A3 str. Loch Maree chromosome, complete genome | 3366 | 28.31% | 91.98 |
| >gi|111114823|ref|NC_008277.1| Borrelia afzelii PKo, complete genome | 1864 | 28.31% | 90.13 |
| >gi|409187975|ref|NC_018887.1| Borrelia afzelii HLJ01 chromosome, complete genome | 1877 | 28.32% | 90.36 |
| >gi|170754211|ref|NC_010516.1| Clostridium botulinum B1 str. Okra chromosome, complete genome | 3398 | 28.34% | 91.88 |
| >gi|343127253|ref|NC_015919.1| Borrelia bissettii DN127 plasmid lp54, complete sequence | 121 | 28.34% | 85.12 |
| >gi|384551479|ref|NC_017339.1| Staphylococcus aureus subsp. aureus JKD6159 plasmid pSaa6159, complete sequence | 31 | 28.36% | 87.10 |
| >gi|296271545|ref|NC_014166.1| Arcobacter nitrofigilis DSM 7299 chromosome, complete genome | 4149 | 28.36% | 89.71 |
| >gi|49398098|ref|NC_005951.1| Staphylococcus aureus subsp. aureus MSSA476 plasmid pSAS, complete sequence | 32 | 28.37% | 84.38 |
| >gi|386858843|ref|NC_017800.1| Borrelia crocidurae str. Achema plasmid unnamed, complete sequence | 1 | 28.38% | 100.00 |
| >gi|110798562|ref|NC_008261.1| Clostridium perfringens ATCC 13124 chromosome, complete genome | 3531 | 28.38% | 90.54 |
| >gi|426202379|ref|NT_187145.1| Borrelia hermsii DAH | 3 | 28.38% | 66.67 |
| >gi|408670633|ref|NC_018747.1| Borrelia garinii NMJW1 chromosome, complete genome | 1813 | 28.39% | 89.19 |
| >gi|167626220|ref|NC_010331.1| Francisella philomiragia subsp. philomiragia ATCC 25017 plasmid pFPHI01, complete sequence | 1 | 28.43% | 100.00 |
| >gi|386853410|ref|NC_017717.1| Borrelia garinii BgVir chromosome linear, complete sequence | 1797 | 28.44% | 89.48 |
| >gi|261599111|ref|NC_013419.1| Blattabacterium sp. (Periplaneta americana) str. BPLAN plasmid pBPLAN, complete sequence | 18 | 28.45% | 83.33 |
| >gi|385334155|ref|NC_017509.1| Mycoplasma hyopneumoniae 168 chromosome, complete genome | 3282 | 28.46% | 83.82 |
| >gi|507382422|ref|NC_021283.1| Mycoplasma hyopneumoniae 168-L, complete genome | 3278 | 28.46% | 83.77 |
| >gi|384206262|ref|NC_017231.1| Borrelia afzelii PKo plasmid cp32-7, complete sequence | 53 | 28.47% | 88.68 |
| >gi|295698239|ref|NC_014109.1| Candidatus Riesia pediculicola USDA chromosome, complete genome | 2112 | 28.48% | 86.93 |
| >gi|72080342|ref|NC_007332.1| Mycoplasma hyopneumoniae 7448 chromosome, complete genome | 3278 | 28.49% | 83.47 |
| >gi|331268188|ref|NC_015425.1| Clostridium botulinum BKT015925 chromosome, complete genome | 2232 | 28.49% | 91.22 |
| >gi|240047135|ref|NC_012806.1| Mycoplasma conjunctivae HRC/581 chromosome, complete genome | 1865 | 28.49% | 87.83 |
| >gi|71894025|ref|NC_007294.1| Mycoplasma synoviae 53, complete genome | 1697 | 28.50% | 85.56 |
| >gi|224586575|ref|NC_012133.1| Borrelia valaisiana VS116 plasmid VS116_cp32-10, complete sequence | 62 | 28.50% | 83.87 |
| >gi|525903163|ref|NC_021831.1| Mycoplasma hyopneumoniae 7422, complete genome | 3268 | 28.51% | 83.23 |
| >gi|71893359|ref|NC_007295.1| Mycoplasma hyopneumoniae J chromosome, complete genome | 3230 | 28.52% | 83.59 |
| >gi|379793806|ref|NC_016942.1| Staphylococcus aureus subsp. aureus MSHR1132 plasmid pST75, complete sequence | 23 | 28.52% | 82.61 |
| >gi|203288911|ref|NC_011265.1| Borrelia duttonii Ly plasmid pl27, complete sequence | 30 | 28.53% | 86.67 |
| >gi|383843664|ref|NC_017173.1| Clostridium difficile CF5, complete genome | 3275 | 28.54% | 91.66 |
| >gi|218249165|ref|NC_011728.1| Borrelia burgdorferi ZS7, complete genome | 1751 | 28.54% | 89.95 |
| >gi|386860095|ref|NC_017818.1| Borrelia crocidurae str. Achema plasmid unnamed, complete sequence | 27 | 28.54% | 88.89 |
| >gi|387825662|ref|NC_017403.1| Borrelia burgdorferi JD1 chromosome, complete genome | 1818 | 28.55% | 89.88 |
| >gi|51247073|ref|NC_006140.1| Desulfotalea psychrophila LSv54 plasmid small, complete sequence | 46 | 28.56% | 69.57 |
| >gi|54019969|ref|NC_006360.1| Mycoplasma hyopneumoniae 232 chromosome, complete genome | 3208 | 28.56% | 83.45 |
| >gi|260681769|ref|NC_013315.1| Clostridium difficile CD196 chromosome, complete genome | 3203 | 28.56% | 91.85 |
| >gi|18308982|ref|NC_003366.1| Clostridium perfringens str. 13 chromosome, complete genome | 3128 | 28.57% | 91.08 |
| >gi|224586416|ref|NC_012128.1| Borrelia valaisiana VS116 plasmid VS116_cp32-5, complete sequence | 50 | 28.57% | 86.00 |
| >gi|15594346|ref|NC_001318.1| Borrelia burgdorferi B31 chromosome, complete genome | 1754 | 28.59% | 89.91 |
| >gi|297618528|ref|NC_014222.1| Methanococcus voltae A3 chromosome, complete genome | 1221 | 28.59% | 89.76 |
| >gi|530320924|ref|NC_022048.1| Borrelia burgdorferi CA382, complete genome | 1761 | 28.60% | 89.84 |
| >gi|383843669|ref|NC_017179.1| Clostridium difficile BI1, complete genome | 3199 | 28.61% | 91.84 |
| >gi|292659168|ref|NC_013974.1| Clostridium difficile BI9 chromosome | 3319 | 28.62% | 91.65 |
| >gi|406705597|ref|NC_018643.1| Alpha proteobacterium HIMB5 chromosome, complete genome | 2400 | 28.63% | 89.08 |
| >gi|256821123|ref|NC_013164.1| Anaerococcus prevotii DSM 20548 plasmid pAPRE01, complete sequence | 88 | 28.63% | 86.36 |
| >gi|387826928|ref|NC_017418.1| Borrelia burgdorferi N40 chromosome, complete genome | 1736 | 28.64% | 90.03 |
| >gi|469816294|ref|NC_020535.1| Staphylococcus aureus subsp. aureus ST228 plasmid pI5S5 complete sequence, isolate 16125 | 47 | 28.66% | 78.72 |
| >gi|550916528|ref|NC_022571.1| Clostridium saccharobutylicum DSM 13864, complete genome | 3480 | 28.66% | 91.98 |
| >gi|514064933|ref|NC_021552.1| Staphylococcus aureus CA-347 plasmid, complete sequence | 28 | 28.67% | 85.71 |
| >gi|87159847|ref|NC_007792.1| Staphylococcus aureus subsp. aureus USA300_FPR3757 plasmid pUSA03, complete sequence | 47 | 28.67% | 78.72 |
| >gi|383843666|ref|NC_017178.1| Clostridium difficile complete genome, strain 2007855 | 3236 | 28.67% | 91.87 |
| >gi|383843665|ref|NC_017174.1| Clostridium difficile M120, complete genome | 3288 | 28.67% | 91.97 |
| >gi|315123578|ref|NC_014801.1| Campylobacter jejuni subsp. jejuni ICDCCJ07001 plasmid pTet, complete sequence | 83 | 28.69% | 89.16 |
| >gi|16119200|ref|NC_003140.1| Staphylococcus aureus subsp. aureus N315 plasmid pN315, complete sequence | 29 | 28.70% | 86.21 |
| >gi|343127324|ref|NC_015921.1| Borrelia bissettii DN127 chromosome, complete genome | 1841 | 28.70% | 90.17 |
| >gi|530575369|ref|NC_022079.1| Borrelia miyamotoi LB-2001, complete genome | 1076 | 28.72% | 92.29 |
| >gi|87159837|ref|NC_007790.1| Staphylococcus aureus subsp. aureus USA300_FPR3757 plasmid pUSA01, complete sequence | 11 | 28.74% | 81.82 |
| >gi|225631039|ref|NC_012417.1| Staphylococcus aureus subsp. aureus USA300_TCH1516 plasmid pUSA01-HOU, complete sequence | 11 | 28.74% | 81.82 |
| >gi|28209834|ref|NC_004557.1| Clostridium tetani E88 chromosome, complete genome | 2199 | 28.75% | 90.45 |
| >gi|507379056|ref|NC_021280.1| Spiroplasma chrysopicola DF-1, complete genome | 1472 | 28.80% | 91.17 |
| >gi|407472016|ref|NC_018657.1| Clostridium acidurici 9a plasmid pCuri3, complete sequence | 2 | 28.80% | 50.00 |
| >gi|260685375|ref|NC_013316.1| Clostridium difficile R20291 chromosome, complete genome | 3235 | 28.81% | 91.87 |
| >gi|557604010|ref|NC_022777.1| Clostridium tetani 12124569 main chromosome, complete genome | 2269 | 28.81% | 90.57 |
| >gi|343126973|ref|NC_015908.1| Borrelia bissettii DN127 plasmid cp32-3, complete sequence | 44 | 28.83% | 84.09 |
| >gi|384206409|ref|NC_017224.1| Borrelia afzelii PKo plasmid cp32-11, complete sequence | 55 | 28.85% | 85.45 |
| >gi|11497103|ref|NC_000949.1| Borrelia burgdorferi B31 plasmid cp32-3, complete sequence | 55 | 28.85% | 83.64 |
| >gi|118442852|ref|NC_008593.1| Clostridium novyi NT chromosome, complete genome | 2070 | 28.86% | 90.43 |
| >gi|203288469|ref|NC_011245.1| Borrelia duttonii Ly plasmid pl28, complete sequence | 37 | 28.87% | 83.78 |
| >gi|479133135|ref|NC_017175.1| Clostridium difficile M68, complete genome | 3306 | 28.88% | 91.53 |
| >gi|449338638|ref|NC_020268.1| Staphylococcus warneri SG1 plasmid clone pvSw6 genomic sequence | 6 | 28.88% | 100.00 |
| >gi|10954532|ref|NC_001733.1| Methanocaldococcus jannaschii DSM 2661 plasmid small ECE, complete sequence | 10 | 28.89% | 90.00 |
| >gi|400406007|ref|NC_018420.1| Secondary endosymbiont of Heteropsylla cubana chromosome, complete genome | 2827 | 28.90% | 92.22 |
| >gi|386859037|ref|NC_017777.1| Borrelia crocidurae str. Achema plasmid unnamed, complete sequence | 5 | 28.92% | 100.00 |
| >gi|383751989|ref|NC_017066.1| Rickettsia typhi str. TH1527 chromosome, complete genome | 895 | 28.92% | 92.63 |
| >gi|51473215|ref|NC_006142.1| Rickettsia typhi str. Wilmington, complete genome | 892 | 28.92% | 92.71 |
| >gi|383842824|ref|NC_017062.1| Rickettsia typhi str. B9991CWPP chromosome, complete genome | 895 | 28.92% | 92.63 |
| >gi|203288832|ref|NC_011261.1| Borrelia duttonii Ly plasmid pl26, complete sequence | 32 | 28.92% | 81.25 |
| >gi|387826639|ref|NC_017427.1| Borrelia burgdorferi JD1 plasmid JD1 cp32-3, complete sequence | 48 | 28.92% | 77.08 |
| >gi|14141823|ref|NC_002774.1| Staphylococcus aureus subsp. aureus Mu50 plasmid VRSAp, complete sequence | 22 | 28.93% | 77.27 |
| >gi|498827228|ref|NC_021183.1| Clostridium pasteurianum BC1 plasmid pCLOPA01, complete sequence | 46 | 28.94% | 91.30 |
| >gi|203288819|ref|NC_011259.1| Borrelia duttonii Ly plasmid pl23b, complete sequence | 41 | 28.95% | 92.68 |
| >gi|73666633|ref|NC_007354.1| Ehrlichia canis str. Jake chromosome, complete genome | 731 | 28.96% | 95.21 |
| >gi|262260515|ref|NC_013453.1| Staphylococcus aureus subsp. aureus ED98 plasmid pAVX, complete sequence | 21 | 28.96% | 95.24 |
| >gi|203288436|ref|NC_011260.1| Borrelia recurrentis A1 plasmid pl53, complete sequence | 71 | 28.97% | 87.32 |
| >gi|218442394|ref|NC_011736.1| Borrelia burgdorferi ZS7 plasmid ZS7_cp32-4, complete sequence | 39 | 28.98% | 82.05 |
| >gi|651865246|ref|NC_022538.1| complete chromosome Acholeplasma palmae | 1496 | 28.98% | 90.04 |
| >gi|383486602|ref|NC_017048.1| Rickettsia prowazekii str. GvV257 chromosome, complete genome | 891 | 28.99% | 92.03 |
| >gi|384442304|ref|NC_017282.1| Campylobacter jejuni subsp. jejuni S3 plasmid pTet, complete sequence | 88 | 28.99% | 89.77 |
| >gi|383500100|ref|NC_017057.1| Rickettsia prowazekii str. RpGvF24 chromosome, complete genome | 889 | 28.99% | 92.01 |
| >gi|387827867|ref|NC_017402.1| Borrelia burgdorferi N40 plasmid N40_cp32-9, complete sequence | 44 | 28.99% | 81.82 |
| >gi|387144417|ref|NC_017332.1| Staphylococcus aureus subsp. aureus TW20 plasmid pTW20_2, complete sequence | 8 | 28.99% | 87.50 |
| >gi|554645846|ref|NC_022605.1| Staphylococcus aureus subsp. aureus Z172 plasmid pZ172_2, complete sequence | 8 | 28.99% | 87.50 |
| >gi|478692434|ref|NC_020992.1| Rickettsia prowazekii str. NMRC Madrid E, complete genome | 902 | 29.00% | 92.46 |
| >gi|383488278|ref|NC_017050.1| Rickettsia prowazekii str. Katsinyian chromosome, complete genome | 890 | 29.00% | 92.02 |
| >gi|386081834|ref|NC_017560.1| Rickettsia prowazekii Rp22 chromosome, complete genome | 892 | 29.00% | 92.04 |
| >gi|383499256|ref|NC_017056.1| Rickettsia prowazekii str. BuV67-CWPP chromosome, complete genome | 891 | 29.00% | 92.03 |
| >gi|15603881|ref|NC_000963.1| Rickettsia prowazekii str. Madrid E chromosome, complete genome | 890 | 29.00% | 92.02 |
| >gi|383489123|ref|NC_017051.1| Rickettsia prowazekii str. Dachau chromosome, complete genome | 890 | 29.00% | 92.02 |
| >gi|387826553|ref|NC_017425.1| Borrelia burgdorferi JD1 plasmid JD1 cp32-6, complete sequence | 37 | 29.00% | 81.08 |
| >gi|433653606|ref|NC_019956.1| Thermoanaerobacterium thermosaccharolyticum M0795 plasmid pTHETHE01, complete sequence | 128 | 29.00% | 89.06 |
| >gi|387603996|ref|NC_017336.1| Staphylococcus aureus subsp. aureus ST398 plasmid pS0385-3, complete sequence | 2 | 29.01% | 100.00 |
| >gi|478693373|ref|NC_020993.1| Rickettsia prowazekii str. Breinl, complete genome | 895 | 29.01% | 92.07 |
| >gi|383487432|ref|NC_017049.1| Rickettsia prowazekii str. Chernikova chromosome, complete genome | 889 | 29.01% | 92.01 |
| >gi|390935776|ref|NC_017998.1| Thermoanaerobacterium saccharolyticum JW/SL-YS485 plasmid pMU3262, complete sequence | 128 | 29.01% | 89.06 |
| >gi|387825444|ref|NC_017393.1| Borrelia burgdorferi JD1 plasmid JD1 cp32-11, complete sequence | 49 | 29.01% | 77.55 |
| >gi|343127060|ref|NC_015910.1| Borrelia bissettii DN127 plasmid cp32-7, complete sequence | 33 | 29.03% | 90.91 |
| >gi|442556480|ref|NC_020128.1| Lawsonia intracellularis N343 plasmid 1, complete sequence | 43 | 29.04% | 95.35 |
| >gi|509154128|ref|NC_021355.1| Methanobrevibacter sp. AbM4, complete genome | 2487 | 29.04% | 90.91 |
| >gi|94972343|ref|NC_008012.1| Lawsonia intracellularis PHE/MN1-00 plasmid 1, complete sequence | 43 | 29.05% | 95.35 |
| >gi|385782932|ref|NC_017350.1| Staphylococcus aureus subsp. aureus 11819-97 plasmid p11819-97, complete sequence | 6 | 29.06% | 100.00 |
| >gi|126697566|ref|NC_009089.1| Peptoclostridium difficile 630, complete genome | 3358 | 29.06% | 91.60 |
| >gi|11497236|ref|NC_000952.1| Borrelia burgdorferi B31 plasmid cp32-7, complete sequence | 37 | 29.07% | 75.68 |
| >gi|121999251|ref|NC_008790.1| Campylobacter jejuni subsp. jejuni 81-176 plasmid pTet, complete sequence | 74 | 29.09% | 90.54 |
| >gi|387826596|ref|NC_017426.1| Borrelia burgdorferi JD1 plasmid JD1 cp32-8, complete sequence | 44 | 29.09% | 75.00 |
| >gi|387781658|ref|NC_017348.1| Staphylococcus aureus subsp. aureus LGA251 plasmid pLGA251, complete sequence | 5 | 29.10% | 80.00 |
| >gi|399140225|ref|NC_018265.1| Melissococcus plutonius DAT561 plasmid 1, complete sequence | 231 | 29.10% | 95.67 |
| >gi|119952806|ref|NC_008710.1| Borrelia turicatae 91E135 chromosome, complete genome | 1039 | 29.12% | 91.43 |
| >gi|386858947|ref|NC_017809.1| Borrelia crocidurae str. Achema plasmid unnamed, complete sequence | 1 | 29.13% | 100.00 |
| >gi|11497281|ref|NC_000953.1| Borrelia burgdorferi B31 plasmid cp32-8, complete sequence | 53 | 29.13% | 81.13 |
| >gi|218442268|ref|NC_011731.1| Borrelia burgdorferi ZS7 plasmid ZS7_cp32-1, complete sequence | 36 | 29.14% | 86.11 |
| >gi|387826859|ref|NC_017398.1| Borrelia burgdorferi N40 plasmid N40_cp32-5, complete sequence | 52 | 29.15% | 84.62 |
| >gi|374339022|ref|NC_016751.1| Marinitoga piezophila KA3 chromosome, complete genome | 3022 | 29.16% | 89.48 |
| >gi|384206020|ref|NC_017225.1| Borrelia afzelii PKo plasmid cp32-12, complete sequence | 41 | 29.16% | 85.37 |
| >gi|387825484|ref|NC_017394.1| Borrelia burgdorferi JD1 plasmid JD1 cp32-1+5, complete sequence | 91 | 29.16% | 86.81 |
| >gi|384206106|ref|NC_017227.1| Borrelia afzelii PKo plasmid cp32-5, complete sequence | 52 | 29.18% | 88.46 |
| >gi|384206150|ref|NC_017228.1| Borrelia afzelii PKo plasmid cp32-9, complete sequence | 58 | 29.18% | 84.48 |
| >gi|330370665|ref|NC_015407.1| Mycoplasma mycoides subsp. capri LC str. 95010 plasmid pMmc-95010, complete sequence | 4 | 29.18% | 50.00 |
| >gi|384206220|ref|NC_017230.1| Borrelia afzelii PKo plasmid cp32-1, complete sequence | 44 | 29.19% | 86.36 |
| >gi|332687277|ref|NC_015517.1| Melissococcus plutonius ATCC 35311 plasmid pMP1, complete sequence | 200 | 29.19% | 95.50 |
| >gi|507383177|ref|NC_021284.1| Spiroplasma syrphidicola EA-1, complete genome | 1416 | 29.20% | 90.68 |
| >gi|343126758|ref|NC_015904.1| Borrelia bissettii DN127 plasmid cp32-4, complete sequence | 36 | 29.21% | 88.89 |
| >gi|111074074|ref|NC_008273.1| Borrelia afzelii PKo plasmid cp30, complete sequence | 63 | 29.22% | 84.13 |
| >gi|442556508|ref|NC_020129.1| Lawsonia intracellularis N343 plasmid 2, complete sequence | 54 | 29.22% | 88.89 |
| >gi|387825622|ref|NC_017397.1| Borrelia burgdorferi JD1 plasmid JD1 cp32-9, complete sequence | 38 | 29.22% | 84.21 |
| >gi|374313777|ref|NC_016632.1| Serratia symbiotica str. 'Cinara cedri' chromosome, complete genome | 1310 | 29.23% | 94.58 |
| >gi|94972373|ref|NC_008013.1| Lawsonia intracellularis PHE/MN1-00 plasmid 2, complete sequence | 54 | 29.23% | 88.89 |
| >gi|387827798|ref|NC_017424.1| Borrelia burgdorferi N40 plasmid N40_cp32-10, complete sequence | 41 | 29.24% | 80.49 |
| >gi|32470532|ref|NC_005004.1| Staphylococcus epidermidis ATCC 12228 plasmid pSE-12228-05, complete sequence | 13 | 29.27% | 100.00 |
| >gi|343126801|ref|NC_015905.1| Borrelia bissettii DN127 plasmid cp32-6, complete sequence | 33 | 29.27% | 87.88 |
| >gi|387825581|ref|NC_017396.1| Borrelia burgdorferi JD1 plasmid JD1 cp32-12, complete sequence | 38 | 29.28% | 78.95 |
| >gi|339320528|ref|NC_015725.1| Mycoplasma bovis Hubei-1 chromosome, complete genome | 989 | 29.29% | 90.50 |
| >gi|11497149|ref|NC_000950.1| Borrelia burgdorferi B31 plasmid cp32-4, complete sequence | 46 | 29.29% | 78.26 |
| >gi|336121001|ref|NC_015636.1| Methanothermococcus okinawensis IH1 chromosome, complete genome | 1019 | 29.30% | 89.70 |
| >gi|392429594|ref|NC_018077.1| Mycoplasma bovis HB0801 chromosome, complete genome | 1040 | 29.31% | 90.96 |
| >gi|384206063|ref|NC_017226.1| Borrelia afzelii PKo plasmid cp32-3, complete sequence | 66 | 29.31% | 86.36 |
| >gi|313678134|ref|NC_014760.1| Mycoplasma bovis PG45 chromosome, complete genome | 1068 | 29.31% | 90.64 |
| >gi|11497325|ref|NC_000954.1| Borrelia burgdorferi B31 plasmid cp32-9, complete sequence | 36 | 29.32% | 86.11 |
| >gi|387826681|ref|NC_017428.1| Borrelia burgdorferi JD1 plasmid JD1 cp32-10, complete sequence | 57 | 29.33% | 78.95 |
| >gi|218203897|ref|NC_011722.1| Borrelia burgdorferi ZS7 plasmid ZS7_cp32-9, complete sequence | 35 | 29.33% | 85.71 |
| >gi|218442354|ref|NC_011735.1| Borrelia burgdorferi ZS7 plasmid ZS7_cp32-12, complete sequence | 43 | 29.33% | 79.07 |
| >gi|472455798|ref|NC_020538.1| Staphylococcus aureus subsp. aureus ST228 plasmid pI7S6 complete sequence, isolate 18412 | 32 | 29.33% | 90.63 |
| >gi|469816323|ref|NC_020539.1| Staphylococcus aureus subsp. aureus ST228 plasmid pI8T7 complete sequence, isolate 18583 | 32 | 29.34% | 90.63 |
| >gi|11497193|ref|NC_000951.1| Borrelia burgdorferi B31 plasmid cp32-6, complete sequence | 45 | 29.34% | 82.22 |
| >gi|11497060|ref|NC_000948.1| Borrelia burgdorferi B31 plasmid cp32-1, complete sequence | 51 | 29.36% | 78.43 |
| >gi|451822614|ref|NC_020292.1| Clostridium saccharoperbutylacetonicum N1-4(HMT) plasmid Csp_135p, complete sequence | 78 | 29.37% | 92.31 |
| >gi|268796558|ref|NC_013519.1| Sebaldella termitidis ATCC 33386 plasmid pSTERM02, complete sequence | 22 | 29.38% | 86.36 |
| >gi|203288416|ref|NC_011258.1| Borrelia recurrentis A1 plasmid pl37, complete sequence | 38 | 29.40% | 81.58 |
| >gi|521147365|ref|NC_021657.1| Staphylococcus aureus Bmb9393 plasmid pBmb9393, complete genome | 5 | 29.40% | 100.00 |
| >gi|203288701|ref|NC_011251.1| Borrelia duttonii Ly plasmid pl41, complete sequence | 40 | 29.40% | 92.50 |
| >gi|556561977|ref|NC_022656.1| Campylobacter coli 15-537360 plasmid pCC42yr, complete sequence | 51 | 29.41% | 88.24 |
| >gi|543950741|ref|NC_022354.1| Campylobacter jejuni subsp. jejuni 00-2544 plasmid, complete sequence | 95 | 29.41% | 88.42 |
| >gi|440509586|ref|NC_020075.1| Candidatus Blochmannia chromaiodes str. 640 chromosome, complete genome | 1031 | 29.45% | 92.73 |
| >gi|203288661|ref|NC_011249.1| Borrelia duttonii Ly plasmid pl36, complete sequence | 51 | 29.48% | 88.24 |
| >gi|442313459|ref|NC_020124.1| Bacillus thuringiensis YBT-1518 plasmid pBMB0228, complete sequence | 38 | 29.52% | 84.21 |
| >gi|537462571|ref|NC_022227.1| Staphylococcus aureus subsp. aureus CN1 plasmid, complete sequence | 8 | 29.53% | 87.50 |
| >gi|343127016|ref|NC_015909.1| Borrelia bissettii DN127 plasmid cp32-5, complete sequence | 46 | 29.54% | 80.43 |
| >gi|203288382|ref|NC_011253.1| Borrelia recurrentis A1 plasmid pl33, complete sequence | 20 | 29.54% | 90.00 |
| >gi|451816898|ref|NC_020291.1| Clostridium saccharoperbutylacetonicum N1-4(HMT), complete genome | 4324 | 29.54% | 92.44 |
| >gi|343126711|ref|NC_015903.1| Borrelia bissettii DN127 plasmid cp32-11, complete sequence | 32 | 29.54% | 93.75 |
| >gi|71891793|ref|NC_007292.1| Candidatus Blochmannia pennsylvanicus str. BPEN chromosome, complete genome | 988 | 29.56% | 93.02 |
| >gi|291319937|ref|NC_013948.1| Mycoplasma agalactiae chromosome, complete genome | 1062 | 29.62% | 88.23 |
| >gi|386858925|ref|NC_017794.1| Borrelia crocidurae str. Achema plasmid unnamed, complete sequence | 15 | 29.63% | 93.33 |
| >gi|150375692|ref|NC_009619.1| Staphylococcus aureus subsp. aureus JH1 plasmid pSJH101, complete sequence | 18 | 29.64% | 77.78 |
| >gi|148244139|ref|NC_009477.1| Staphylococcus aureus subsp. aureus JH9 plasmid pSJH901, complete sequence | 18 | 29.64% | 77.78 |
| >gi|384206482|ref|NC_017236.1| Borrelia afzelii PKo plasmid lp28-8, complete sequence | 48 | 29.64% | 91.67 |
| >gi|257124814|ref|NC_013192.1| Leptotrichia buccalis C-1013-b chromosome, complete genome | 4034 | 29.65% | 87.98 |
| >gi|554651112|ref|NC_022608.1| Carnobacterium sp. WN1359 plasmid pWNCR9, complete sequence | 16 | 29.65% | 81.25 |
| >gi|568132683|ref|NC_023063.1| Ehrlichia muris AS145, complete genome | 648 | 29.66% | 93.98 |
| >gi|71082709|ref|NC_007205.1| Candidatus Pelagibacter ubique HTCC1062 chromosome, complete genome | 1909 | 29.68% | 89.10 |
| >gi|222823046|ref|NC_012039.1| Campylobacter lari RM2100, complete genome | 2772 | 29.70% | 88.67 |
| >gi|148377268|ref|NC_009497.1| Mycoplasma agalactiae PG2 chromosome, complete genome | 977 | 29.71% | 88.43 |
| >gi|169823514|ref|NC_010371.1| Finegoldia magna ATCC 29328 plasmid pFMC, complete sequence | 164 | 29.73% | 87.20 |
| >gi|449092676|ref|NC_020242.1| Bacillus thuringiensis serovar kurstaki str. HD73 plasmid pHT8_2, complete sequence | 11 | 29.73% | 90.91 |
| >gi|409187966|ref|NC_018881.1| Bacillus thuringiensis Bt407 plasmid BTB_8p, complete sequence | 11 | 29.75% | 90.91 |
| >gi|336478154|ref|NC_015679.1| Blattabacterium sp. (Blattella germanica) str. Bge plasmid pBge, complete sequence | 17 | 29.82% | 88.24 |
| >gi|187917883|ref|NC_010673.1| Borrelia hermsii DAH chromosome, complete genome | 947 | 29.83% | 91.02 |
| >gi|150014892|ref|NC_009617.1| Clostridium beijerinckii NCIMB 8052 chromosome, complete genome | 3660 | 29.86% | 92.68 |
| >gi|68535043|ref|NC_007169.1| Staphylococcus haemolyticus JCSC1435 plasmid pSHaeA, complete sequence | 3 | 29.87% | 100.00 |
| >gi|203288682|ref|NC_011250.1| Borrelia duttonii Ly plasmid pl40, complete sequence | 51 | 29.89% | 88.24 |
| >gi|386858829|ref|NC_017814.1| Borrelia crocidurae str. Achema plasmid unnamed, complete sequence | 6 | 29.90% | 83.33 |
| >gi|407472532|ref|NC_018664.1| Clostridium acidurici 9a chromosome, complete genome | 1935 | 29.93% | 91.21 |
| >gi|87159843|ref|NC_007791.1| Staphylococcus aureus subsp. aureus USA300_FPR3757 plasmid pUSA02, complete sequence | 0 | 30.01% | 0.00 |
| >gi|554074371|ref|NC_022587.1| Lactococcus lactis subsp. lactis KLDS 4.0325 strain Lactococcus lactis subsp. lactis strain plasmid 1, complete sequence | 4 | 30.02% | 100.00 |
| >gi|218847927|ref|NC_011775.1| Bacillus cereus G9842 plasmid pG9842_209, complete sequence | 141 | 30.03% | 93.62 |
| >gi|429216255|ref|NC_019791.1| Caldisphaera lagunensis DSM 15908 chromosome, complete genome | 1165 | 30.03% | 91.24 |
| >gi|150400439|ref|NC_009635.1| Methanococcus aeolicus Nankai-3 chromosome, complete genome | 947 | 30.04% | 91.76 |
| >gi|77102894|ref|NC_006629.2| Staphylococcus aureus subsp. aureus COL plasmid pT181, complete sequence | 0 | 30.05% | 0.00 |
| >gi|32470588|ref|NC_005008.1| Staphylococcus epidermidis ATCC 12228 plasmid pSE-12228-01, complete sequence | 0 | 30.05% | 0.00 |
| >gi|262225767|ref|NC_013452.1| Staphylococcus aureus subsp. aureus ED98 plasmid pT181, complete sequence | 0 | 30.07% | 0.00 |
| >gi|402574993|ref|NC_018490.1| Bacillus thuringiensis HD-771 plasmid p08, complete sequence | 14 | 30.07% | 92.86 |
| >gi|414075296|ref|NC_019434.1| Lactococcus lactis subsp. cremoris UC509.9 plasmid pCIS2, complete sequence | 13 | 30.07% | 84.62 |
| >gi|261403873|ref|NC_013409.1| Methanocaldococcus vulcanius M7 plasmid pMETVU02, complete sequence | 0 | 30.08% | 0.00 |
| >gi|88657561|ref|NC_007799.1| Ehrlichia chaffeensis str. Arkansas, complete genome | 660 | 30.10% | 93.48 |
| >gi|451822721|ref|NC_020294.1| Candidatus Kinetoplastibacterium desouzaii TCC079E, complete genome | 856 | 30.17% | 92.99 |
| >gi|442743028|ref|NC_020135.1| Candidatus Uzinura diaspidicola str. ASNER, complete genome | 452 | 30.20% | 90.49 |
| >gi|203288888|ref|NC_011264.1| Borrelia duttonii Ly plasmid pl32, complete sequence | 16 | 30.21% | 81.25 |
| >gi|203288751|ref|NC_011256.1| Borrelia duttonii Ly plasmid pl70, complete sequence | 61 | 30.23% | 85.25 |
| >gi|203288730|ref|NC_011254.1| Borrelia duttonii Ly plasmid pl42, complete sequence | 29 | 30.25% | 79.31 |
| >gi|68535050|ref|NC_007171.1| Staphylococcus haemolyticus JCSC1435 plasmid pSHaeC, complete sequence | 8 | 30.26% | 75.00 |
| >gi|152973842|ref|NC_009673.1| Bacillus cereus subsp. cytotoxis NVH 391-98 plasmid pBC9801, complete sequence | 15 | 30.26% | 86.67 |
| >gi|385798917|ref|NC_017455.1| Halanaerobium praevalens DSM 2228 chromosome, complete genome | 2692 | 30.29% | 89.64 |
| >gi|57236892|ref|NC_003912.7| Campylobacter jejuni RM1221, complete genome | 3372 | 30.31% | 88.20 |
| >gi|543948972|ref|NC_022353.1| Campylobacter jejuni subsp. jejuni 00-2544 genome | 3052 | 30.37% | 88.79 |
| >gi|268318538|ref|NC_013505.1| Lactobacillus johnsonii FI9785 plasmid p9785L, complete sequence | 19 | 30.38% | 89.47 |
| >gi|543947230|ref|NC_022352.1| Campylobacter jejuni subsp. jejuni 00-2426 genome | 2981 | 30.40% | 88.80 |
| >gi|543945414|ref|NC_022351.1| Campylobacter jejuni subsp. jejuni 00-2538 genome | 3056 | 30.43% | 88.81 |
| >gi|116326540|ref|NC_008504.1| Lactococcus lactis subsp. cremoris SK11 plasmid 2, complete sequence | 10 | 30.44% | 60.00 |
| >gi|374290638|ref|NC_016598.1| Blattabacterium sp. (Cryptocercus punctulatus) str. Cpu plasmid pCpu, complete sequence | 13 | 30.45% | 76.92 |
| >gi|426202340|ref|NT_187140.1| Borrelia hermsii DAH clone fragment 1047L | 11 | 30.45% | 100.00 |
| >gi|549691986|ref|NC_022529.1| Campylobacter jejuni 4031 genome sequence | 3114 | 30.47% | 89.15 |
| >gi|384442354|ref|NC_017281.1| Campylobacter jejuni subsp. jejuni S3 chromosome, complete genome | 3141 | 30.49% | 88.19 |
| >gi|525911748|ref|NC_021834.1| Campylobacter jejuni 32488, complete sequence | 3209 | 30.49% | 88.78 |
| >gi|544061647|ref|NC_022362.1| Campylobacter jejuni subsp. jejuni 00-2425, complete genome | 3160 | 30.51% | 89.02 |
| >gi|479205603|ref|NC_021041.1| Megamonas hypermegale ART12/1 draft genome | 1629 | 30.51% | 90.85 |
| >gi|189182907|ref|NC_010793.1| Orientia tsutsugamushi str. Ikeda, complete genome | 1016 | 30.51% | 92.22 |
| >gi|161510924|ref|NC_010063.1| Staphylococcus aureus subsp. aureus USA300_TCH1516 plasmid pUSA300HOUMR, complete sequence | 14 | 30.52% | 92.86 |
| >gi|148283997|ref|NC_009488.1| Orientia tsutsugamushi str. Boryong, complete genome | 1161 | 30.53% | 91.99 |
| >gi|410687359|ref|NC_018709.2| Campylobacter jejuni subsp. jejuni PT14 chromosome, complete genome | 3009 | 30.54% | 89.10 |
| >gi|157414322|ref|NC_009839.1| Campylobacter jejuni subsp. jejuni 81116, complete genome | 3048 | 30.54% | 88.62 |
| >gi|15791399|ref|NC_002163.1| Campylobacter jejuni subsp. jejuni NCTC 11168 = ATCC 700819 chromosome, complete genome | 3037 | 30.55% | 88.90 |
| >gi|403054816|ref|NC_018521.1| Campylobacter jejuni subsp. jejuni NCTC 11168-BN148, complete genome | 3037 | 30.55% | 88.90 |
| >gi|384447320|ref|NC_017279.1| Campylobacter jejuni subsp. jejuni IA3902 chromosome, complete genome | 3012 | 30.57% | 89.11 |
| >gi|549730541|ref|NC_022547.1| Halyomorpha halys symbiont DNA, complete genome | 1437 | 30.57% | 93.46 |
| >gi|153950938|ref|NC_009707.1| Campylobacter jejuni subsp. doylei 269.97 chromosome, complete genome | 3549 | 30.57% | 88.79 |
| >gi|488768999|ref|NC_021182.1| Clostridium pasteurianum BC1, complete genome | 3255 | 30.58% | 92.63 |
| >gi|337287573|ref|NC_015682.1| Thermodesulfobacterium geofontis OPF15 chromosome, complete genome | 4002 | 30.59% | 85.96 |
| >gi|472320519|ref|NC_020830.1| Polaribacter sp. MED152, complete genome | 2807 | 30.59% | 91.09 |
| >gi|315123616|ref|NC_014802.1| Campylobacter jejuni subsp. jejuni ICDCCJ07001 chromosome, complete genome | 3059 | 30.60% | 89.02 |
| >gi|384440681|ref|NC_017280.1| Campylobacter jejuni subsp. jejuni M1 chromosome, complete genome | 2981 | 30.60% | 88.66 |
| >gi|10957099|ref|NC_002252.1| Buchnera aphidicola str. APS (Acyrthosiphon pisum) plasmid pTrp, complete sequence | 24 | 30.61% | 91.67 |
| >gi|121612099|ref|NC_008787.1| Campylobacter jejuni subsp. jejuni 81-176 chromosome, complete genome | 2990 | 30.62% | 88.80 |
| >gi|444335882|ref|NC_020196.1| Blattabacterium sp. (Blatta orientalis) str. Tarazona plasmid, complete sequence | 11 | 30.63% | 90.91 |
| >gi|384863394|ref|NC_017346.1| Staphylococcus aureus subsp. aureus ECT-R 2 plasmid pLUH01, complete sequence | 1 | 30.66% | 100.00 |
| >gi|392395704|ref|NC_018018.1| Flexibacter litoralis DSM 6794 chromosome, complete genome | 8270 | 30.67% | 89.95 |
| >gi|449338590|ref|NC_020266.1| Staphylococcus warneri SG1 plasmid clone pvSw4 genomic sequence | 17 | 30.70% | 100.00 |
| >gi|193082772|ref|NC_011025.1| Mycoplasma arthritidis 158L3-1 chromosome, complete genome | 951 | 30.71% | 90.43 |
| >gi|402558206|ref|NC_018499.1| Bacillus cereus FRI-35 plasmid p03, complete sequence | 32 | 30.72% | 87.50 |
| >gi|387603991|ref|NC_017334.1| Staphylococcus aureus subsp. aureus ST398 plasmid pS0385-1, complete sequence | 0 | 30.73% | 0.00 |
| >gi|73663756|ref|NC_007351.1| Staphylococcus saprophyticus subsp. saprophyticus ATCC 15305 plasmid pSSP1, complete sequence | 15 | 30.75% | 93.33 |
| >gi|449092563|ref|NC_020239.1| Bacillus thuringiensis serovar kurstaki str. HD73 plasmid pHT77, complete sequence | 54 | 30.78% | 92.59 |
| >gi|409187974|ref|NC_018886.1| Bacillus thuringiensis Bt407 plasmid BTB_9p, complete sequence | 17 | 30.79% | 94.12 |
| >gi|384183624|ref|NC_017207.1| Bacillus thuringiensis serovar chinensis CT-43 plasmid pCT8513, complete sequence | 17 | 30.79% | 94.12 |
| >gi|449092659|ref|NC_020241.1| Bacillus thuringiensis serovar kurstaki str. HD73 plasmid pHT8_1, complete sequence | 17 | 30.79% | 94.12 |
| >gi|123965234|ref|NC_008817.1| Prochlorococcus marinus str. MIT 9515, complete genome | 2258 | 30.79% | 90.92 |
| >gi|33860560|ref|NC_005072.1| Prochlorococcus marinus subsp. pastoris str. CCMP1986 chromosome, complete genome | 2132 | 30.80% | 90.62 |
| >gi|217076133|ref|NC_011653.1| Thermosipho africanus TCF52B chromosome, complete genome | 2547 | 30.81% | 87.63 |
| >gi|222142576|ref|NC_011996.1| Macrococcus caseolyticus JCSC5402 plasmid pMCCL2, complete sequence | 71 | 30.82% | 84.51 |
| >gi|336171143|ref|NC_015638.1| Lacinutrix sp. 5H-3-7-4 chromosome, complete genome | 2860 | 30.82% | 89.06 |
| >gi|537462731|ref|NC_022228.1| Staphylococcus aureus subsp. aureus CN1 plasmid, complete sequence | 6 | 30.83% | 66.67 |
| >gi|529230077|ref|NC_021989.1| Enterococcus faecium Aus0085 plasmid p4, complete sequence | 21 | 30.87% | 85.71 |
| >gi|387907363|ref|NC_017925.1| Blattabacterium sp. (Blaberus giganteus) plasmid pBGIBA, complete sequence | 9 | 30.88% | 88.89 |
| >gi|384206451|ref|NC_017235.1| Borrelia afzelii PKo plasmid lp28-7, complete sequence | 47 | 30.89% | 91.49 |
| >gi|67078320|ref|NC_007104.1| Bacillus cereus E33L plasmid pE33L5, complete sequence | 9 | 30.89% | 88.89 |
| >gi|384456541|ref|NC_017296.1| Clostridium acetobutylicum EA 2018 EA2018plasmid, complete sequence | 141 | 30.91% | 91.49 |
| >gi|15004705|ref|NC_001988.2| Clostridium acetobutylicum ATCC 824 plasmid pSOL1, complete sequence | 141 | 30.91% | 91.49 |
| >gi|337735032|ref|NC_015686.1| Clostridium acetobutylicum DSM 1731 plasmid pSMBa, complete sequence | 141 | 30.91% | 91.49 |
| >gi|203288465|ref|NC_011263.1| Borrelia recurrentis A1 plasmid pl6, complete sequence | 20 | 30.92% | 85.00 |
| >gi|15893298|ref|NC_003030.1| Clostridium acetobutylicum ATCC 824 chromosome, complete genome | 2533 | 30.93% | 90.68 |
| >gi|384456718|ref|NC_017295.1| Clostridium acetobutylicum EA 2018 chromosome, complete genome | 2528 | 30.93% | 90.70 |
| >gi|337735209|ref|NC_015687.1| Clostridium acetobutylicum DSM 1731 chromosome, complete genome | 2534 | 30.93% | 90.69 |
| >gi|451811147|ref|NC_020283.1| Candidatus Kinetoplastibacterium crithidii TCC036E, complete genome | 906 | 30.96% | 93.27 |
| >gi|222143522|ref|NC_012003.1| Macrococcus caseolyticus JCSC5402 plasmid pMCCL8, complete sequence | 6 | 30.96% | 100.00 |
| >gi|429462518|ref|NC_019815.1| Candidatus Kinetoplastibacterium crithidii (ex Angomonas deanei ATCC 30255), complete genome | 903 | 30.96% | 93.02 |
| >gi|67078390|ref|NC_007107.1| Bacillus cereus E33L plasmid pE33L9, complete sequence | 13 | 31.01% | 92.31 |
| >gi|379022404|ref|NC_016929.1| Rickettsia canadensis str. CA410 chromosome, complete genome | 798 | 31.01% | 91.98 |
| >gi|386858840|ref|NC_017795.1| Borrelia crocidurae str. Achema plasmid unnamed, complete sequence | 4 | 31.02% | 100.00 |
| >gi|148642060|ref|NC_009515.1| Methanobrevibacter smithii ATCC 35061 chromosome, complete genome | 1425 | 31.03% | 90.18 |
| >gi|452202695|ref|NC_020393.1| Bacillus thuringiensis serovar thuringiensis str. IS5056 plasmid pIS56-107, complete sequence | 65 | 31.05% | 93.85 |
| >gi|313652020|ref|NC_014749.1| Calditerrivibrio nitroreducens DSM 19672 plasmid pCALNI01, complete sequence | 71 | 31.05% | 84.51 |
| >gi|157803189|ref|NC_009879.1| Rickettsia canadensis str. McKiel, complete genome | 795 | 31.05% | 91.95 |
| >gi|433653593|ref|NC_019961.1| Prevotella dentalis DSM 3688 plasmid pPREDE01, complete sequence | 3 | 31.06% | 66.67 |
| >gi|563700047|ref|NC_023002.1| Candidate division SR1 bacterium RAAC1_SR1_1, complete genome | 1580 | 31.06% | 88.80 |
| >gi|217316512|ref|NC_011654.1| Bacillus cereus AH187 plasmid pAH187_12, complete sequence | 6 | 31.06% | 100.00 |
| >gi|68535047|ref|NC_007170.1| Staphylococcus haemolyticus JCSC1435 plasmid pSHaeB, complete sequence | 6 | 31.07% | 66.67 |
| >gi|325972867|ref|NC_015155.1| Mycoplasma suis str. Illinois chromosome, complete genome | 1836 | 31.08% | 83.01 |
| >gi|325989358|ref|NC_015153.1| Mycoplasma suis KI3806, complete genome | 1774 | 31.08% | 83.37 |
| >gi|376269859|ref|NC_016780.1| Bacillus cereus F837/76 plasmid pF837_10, complete sequence | 11 | 31.08% | 81.82 |
| >gi|554377794|ref|NC_022592.1| Clostridium autoethanogenum DSM 10061, complete genome | 2957 | 31.09% | 92.05 |
| >gi|300853232|ref|NC_014328.1| Clostridium ljungdahlii DSM 13528 chromosome, complete genome | 3087 | 31.11% | 92.03 |
| >gi|557822170|ref|NC_022793.1| Candidatus Liberibacter americanus str. Sao Paulo, complete genome | 1073 | 31.11% | 92.26 |
| >gi|284928601|ref|NC_013771.1| Cyanobacterium UCYN-A, complete genome | 1319 | 31.12% | 91.51 |
| >gi|291278433|ref|NC_013939.1| Deferribacter desulfuricans SSM1, complete genome | 2652 | 31.12% | 90.84 |
| >gi|543939817|ref|NC_022348.1| Campylobacter coli CVM N29710 plasmid pN29710-2, complete sequence | 9 | 31.13% | 77.78 |
| >gi|157412338|ref|NC_009840.1| Prochlorococcus marinus str. MIT 9215 chromosome, complete genome | 2390 | 31.15% | 90.71 |
| >gi|302872922|ref|NC_014393.1| Clostridium cellulovorans 743B chromosome, complete genome | 3159 | 31.21% | 90.82 |
| >gi|78778385|ref|NC_007577.1| Prochlorococcus marinus str. MIT 9312, complete genome | 2299 | 31.21% | 91.13 |
| >gi|451936134|ref|NC_020299.1| Candidatus Kinetoplastibacterium oncopeltii TCC290E, complete genome | 655 | 31.23% | 91.15 |
| >gi|123967536|ref|NC_008816.1| Prochlorococcus marinus str. AS9601, complete genome | 2216 | 31.32% | 91.16 |
| >gi|150398760|ref|NC_009634.1| Methanococcus vannielii SB chromosome, complete genome | 1985 | 31.33% | 89.27 |
| >gi|126695337|ref|NC_009091.1| Prochlorococcus marinus str. MIT 9301, complete genome | 2185 | 31.34% | 91.03 |
| >gi|73663802|ref|NC_007352.1| Staphylococcus saprophyticus subsp. saprophyticus ATCC 15305 plasmid pSSP2, complete sequence | 9 | 31.34% | 100.00 |
| >gi|449338615|ref|NC_020267.1| Staphylococcus warneri SG1 plasmid clone pvSw5 genomic sequence | 7 | 31.36% | 100.00 |
| >gi|117621834|ref|NC_008569.1| Borrelia afzelii PKo plasmid lp25, complete sequence | 44 | 31.37% | 90.91 |
| >gi|383449352|ref|NC_017025.1| Flavobacterium indicum GPTSA100-9, complete genome | 3085 | 31.38% | 90.08 |
| >gi|385325853|ref|NC_017503.1| Mycoplasma gallisepticum str. F chromosome, complete genome | 771 | 31.40% | 89.62 |
| >gi|150019913|ref|NC_009616.1| Thermosipho melanesiensis BI429 chromosome, complete genome | 2474 | 31.40% | 88.00 |
| >gi|557624504|ref|NC_022783.1| Bacillus toyonensis BCT-7112 plasmid pBCT8, complete sequence | 9 | 31.40% | 77.78 |
| >gi|15668172|ref|NC_000909.1| Methanocaldococcus jannaschii DSM 2661 chromosome, complete genome | 1040 | 31.43% | 89.23 |
| >gi|332685534|ref|NC_015516.1| Melissococcus plutonius ATCC 35311 chromosome, complete genome | 2032 | 31.43% | 89.37 |
| >gi|479169109|ref|NC_021019.1| Eubacterium cylindroides T2-87 draft genome | 741 | 31.43% | 89.88 |
| >gi|543939971|ref|NC_022347.1| Campylobacter coli CVM N29710, complete genome | 2672 | 31.44% | 88.06 |
| >gi|381356294|ref|NC_017055.1| Rickettsia rhipicephali str. 3-7-female6-CWPP plasmid pMCC_1, complete sequence | 6 | 31.45% | 100.00 |
| >gi|556577981|ref|NC_022660.1| Campylobacter coli 15-537360, complete genome | 2664 | 31.45% | 88.59 |
| >gi|406025741|ref|NC_018606.1| Cardinium endosymbiont cEper1 of Encarsia pergandiella plasmid pCher, complete sequence | 66 | 31.45% | 81.82 |
| >gi|379726618|ref|NC_016938.1| Melissococcus plutonius DAT561 chromosome 1, complete genome | 2001 | 31.45% | 89.21 |
| >gi|386262266|ref|NC_016510.2| Flavobacterium columnare ATCC 49512 chromosome, complete genome | 4247 | 31.46% | 89.88 |
| >gi|565626784|ref|NC_023030.1| Mycoplasma gallisepticum S6, complete genome | 808 | 31.47% | 88.49 |
| >gi|384183635|ref|NC_017209.1| Bacillus thuringiensis serovar chinensis CT-43 plasmid pCT14, complete sequence | 15 | 31.47% | 86.67 |
| >gi|385325086|ref|NC_017502.1| Mycoplasma gallisepticum str. R(high) chromosome, complete genome | 854 | 31.47% | 87.59 |
| >gi|294660180|ref|NC_004829.2| Mycoplasma gallisepticum str. R(low) chromosome, complete genome | 854 | 31.47% | 87.59 |
| >gi|449338585|ref|NC_020265.1| Staphylococcus warneri SG1 plasmid clone pvSw3 genomic sequence | 0 | 31.47% | 0.00 |
| >gi|261402131|ref|NC_013407.1| Methanocaldococcus vulcanius M7 chromosome, complete genome | 1033 | 31.49% | 86.45 |
| >gi|32470520|ref|NC_005003.1| Staphylococcus epidermidis ATCC 12228 plasmid pSE-12228-06, complete sequence | 12 | 31.50% | 83.33 |
| >gi|409187969|ref|NC_018878.1| Bacillus thuringiensis Bt407 plasmid BTB_502p, complete sequence | 428 | 31.52% | 89.95 |
| >gi|343127220|ref|NC_015920.1| Borrelia bissettii DN127 plasmid lp56, complete sequence | 44 | 31.52% | 84.09 |
| >gi|328958803|ref|NC_015390.1| Carnobacterium sp. 17-4 plasmid pCAR50, complete sequence | 48 | 31.53% | 87.50 |
| >gi|218875340|ref|NC_011780.1| Borrelia burgdorferi ZS7 plasmid ZS7_lp28-1, complete sequence | 38 | 31.55% | 89.47 |
| >gi|386829695|ref|NC_017762.1| Helicobacter cinaedi PAGU611 plasmid pHci1, complete sequence | 15 | 31.55% | 93.33 |
| >gi|387826906|ref|NC_017414.1| Borrelia burgdorferi N40 plasmid N40_lp36, complete sequence | 60 | 31.55% | 86.67 |
| >gi|38349555|ref|NC_005213.1| Nanoarchaeum equitans Kin4-M chromosome, complete genome | 272 | 31.56% | 90.07 |
| >gi|401768909|ref|NC_018410.1| Mycoplasma gallisepticum WI01_2001.043-13-2P chromosome, complete genome | 723 | 31.56% | 89.49 |
| >gi|203288641|ref|NC_011248.1| Borrelia duttonii Ly plasmid pl35, complete sequence | 26 | 31.57% | 88.46 |
| >gi|452202023|ref|NC_020391.1| Bacillus thuringiensis serovar thuringiensis str. IS5056 plasmid pIS56-11, complete sequence | 15 | 31.58% | 86.67 |
| >gi|543950817|ref|NC_022355.1| Campylobacter coli CVM N29710 plasmid pN29710-1, complete sequence | 95 | 31.58% | 87.37 |
| >gi|401765853|ref|NC_018406.1| Mycoplasma gallisepticum VA94_7994-1-7P chromosome, complete genome | 735 | 31.58% | 89.66 |
| >gi|401771165|ref|NC_018413.1| Mycoplasma gallisepticum NC08_2008.031-4-3P chromosome, complete genome | 700 | 31.59% | 90.00 |
| >gi|401766621|ref|NC_018407.1| Mycoplasma gallisepticum NC95_13295-2-2P chromosome, complete genome | 727 | 31.59% | 89.55 |
| >gi|148244169|ref|NC_009465.1| Candidatus Vesicomyosocius okutanii HA, complete genome | 582 | 31.59% | 93.81 |
| >gi|401768148|ref|NC_018409.1| Mycoplasma gallisepticum NY01_2001.047-5-1P chromosome, complete genome | 742 | 31.60% | 89.22 |
| >gi|222143325|ref|NC_012000.1| Macrococcus caseolyticus JCSC5402 plasmid pMCCL5, complete sequence | 2 | 31.61% | 100.00 |
| >gi|157149578|ref|NC_009795.1| Campylobacter concisus 13826 plasmid pCCON31, complete sequence | 33 | 31.61% | 90.91 |
| >gi|218868768|ref|NC_011779.1| Borrelia burgdorferi ZS7 plasmid ZS7_lp28-2, complete sequence | 58 | 31.62% | 86.21 |
| >gi|387826748|ref|NC_017412.1| Borrelia burgdorferi JD1 plasmid JD1 lp28-7, complete sequence | 49 | 31.62% | 83.67 |
| >gi|401769656|ref|NC_018411.1| Mycoplasma gallisepticum NC06_2006.080-5-2P chromosome, complete genome | 716 | 31.62% | 89.53 |
| >gi|409187968|ref|NC_018884.1| Bacillus thuringiensis Bt407 plasmid BTB_5p, complete sequence | 4 | 31.62% | 75.00 |
| >gi|157826385|ref|NC_009883.1| Rickettsia bellii OSU 85-389 chromosome, complete genome | 963 | 31.63% | 92.42 |
| >gi|401770401|ref|NC_018412.1| Mycoplasma gallisepticum CA06_2006.052-5-2P chromosome, complete genome | 755 | 31.63% | 88.61 |
| >gi|384551502|ref|NC_017335.1| Staphylococcus aureus subsp. aureus ST398 plasmid pS0385-2, complete sequence | 3 | 31.64% | 66.67 |
| >gi|312136230|ref|NC_014658.1| Methanothermus fervidus DSM 2088 chromosome, complete genome | 963 | 31.64% | 86.19 |
| >gi|436736068|ref|NC_020052.1| Stanieria cyanosphaera PCC 7437 plasmid pSTA7437.04, complete sequence | 13 | 31.64% | 84.62 |
| >gi|11496664|ref|NC_001852.1| Borrelia burgdorferi B31 plasmid lp28-2, complete sequence | 57 | 31.64% | 85.96 |
| >gi|91204815|ref|NC_007940.1| Rickettsia bellii RML369-C chromosome, complete genome | 947 | 31.65% | 91.97 |
| >gi|401767376|ref|NC_018408.1| Mycoplasma gallisepticum NC96_1596-4-2P chromosome, complete genome | 761 | 31.65% | 88.44 |
| >gi|289193301|ref|NC_013888.1| Methanocaldococcus sp. FS406-22 plasmid pFS01, complete sequence | 15 | 31.65% | 93.33 |
| >gi|330812975|ref|NC_015380.1| Candidatus Pelagibacter sp. IMCC9063 chromosome, complete genome | 2327 | 31.67% | 88.27 |
| >gi|402551299|ref|NC_018496.1| Mycoplasma genitalium M6282 chromosome, complete genome | 705 | 31.67% | 91.21 |
| >gi|402550799|ref|NC_018495.1| Mycoplasma genitalium M2321 chromosome, complete genome | 704 | 31.67% | 91.34 |
| >gi|402552294|ref|NC_018498.1| Mycoplasma genitalium M2288 chromosome, complete genome | 726 | 31.67% | 91.05 |
| >gi|402551784|ref|NC_018497.1| Mycoplasma genitalium M6320 chromosome, complete genome | 704 | 31.68% | 91.05 |
| >gi|108885074|ref|NC_000908.2| Mycoplasma genitalium G37 chromosome, complete genome | 704 | 31.69% | 91.19 |
| >gi|568131692|ref|NC_023062.1| Mycoplasma ovis str. Michigan, complete genome | 967 | 31.69% | 88.42 |
| >gi|157954006|ref|NC_009897.1| Rickettsia massiliae MTU5 plasmid pRMA, complete sequence | 6 | 31.71% | 100.00 |
| >gi|154147866|ref|NC_009714.1| Campylobacter hominis ATCC BAA-381, complete genome | 2956 | 31.73% | 89.24 |
| >gi|387827738|ref|NC_017419.1| Borrelia burgdorferi N40 plasmid N40_lp28-2, complete sequence | 58 | 31.74% | 86.21 |
| >gi|408671635|ref|NC_018745.1| Emticicia oligotrophica DSM 17448 plasmid pEMTOL05, complete sequence | 34 | 31.75% | 94.12 |
| >gi|533108975|ref|NC_022132.1| Campylobacter coli 76339, complete genome | 2448 | 31.76% | 87.87 |
| >gi|409187967|ref|NC_018883.1| Bacillus thuringiensis Bt407 plasmid BTB_6p, complete sequence | 8 | 31.76% | 87.50 |
| >gi|452202016|ref|NC_020390.1| Bacillus thuringiensis serovar thuringiensis str. IS5056 plasmid pIS56-6, complete sequence | 8 | 31.76% | 87.50 |
| >gi|384183658|ref|NC_017210.1| Bacillus thuringiensis serovar chinensis CT-43 plasmid pCT6880, complete sequence | 8 | 31.76% | 87.50 |
| >gi|32470581|ref|NC_005007.1| Staphylococcus epidermidis ATCC 12228 plasmid pSE-12228-02, complete sequence | 5 | 31.78% | 60.00 |
| >gi|387908781|ref|NC_017919.1| Helicobacter pylori XZ274 plasmid pXZ274, complete sequence | 23 | 31.78% | 82.61 |
| >gi|449092687|ref|NC_020250.1| Bacillus thuringiensis serovar kurstaki str. HD73 plasmid pHT11, complete sequence | 15 | 31.81% | 86.67 |
| >gi|452202187|ref|NC_020382.1| Bacillus thuringiensis serovar thuringiensis str. IS5056 plasmid pIS56-68, complete sequence | 54 | 31.81% | 92.59 |
| >gi|327399879|ref|NC_015319.1| Lactobacillus amylovorus GRL 1112 plasmid1, complete sequence | 26 | 31.83% | 96.15 |
| >gi|445060929|ref|NC_020165.1| Staphylococcus warneri SG1 plasmid pSZ4, complete sequence | 6 | 31.87% | 83.33 |
| >gi|307591243|ref|NC_014535.1| Cyanothece sp. PCC 7822 plasmid Cy782206, complete sequence | 8 | 31.87% | 75.00 |
| >gi|357420702|ref|NC_016150.1| Blattabacterium sp. (Mastotermes darwiniensis) str. MADAR plasmid pMADAR_001, complete sequence | 8 | 31.90% | 75.00 |
| >gi|67078381|ref|NC_007106.1| Bacillus cereus E33L plasmid pE33L8, complete sequence | 9 | 31.90% | 100.00 |
| >gi|57854744|ref|NC_006663.1| Staphylococcus epidermidis RP62A plasmid pSERP, complete sequence | 13 | 31.93% | 92.31 |
| >gi|162446888|ref|NC_010163.1| Acholeplasma laidlawii PG-8A chromosome, complete genome | 584 | 31.93% | 90.24 |
| >gi|67078326|ref|NC_007105.1| Bacillus cereus E33L plasmid pE33L54, complete sequence | 43 | 31.95% | 93.02 |
| >gi|414073319|ref|NC_019438.1| Lactococcus lactis subsp. cremoris UC509.9 plasmid pCIS1, complete sequence | 2 | 31.97% | 100.00 |
| >gi|219853343|ref|NC_011837.1| Clostridium kluyveri NBRC 12016, complete genome | 2601 | 31.99% | 91.77 |
| >gi|385839642|ref|NC_017499.1| Lactobacillus salivarius CECT 5713 plasmid pHN3, complete sequence | 142 | 32.00% | 87.32 |
| >gi|402558329|ref|NC_018488.1| Bacillus thuringiensis HD-771 plasmid p04, complete sequence | 59 | 32.01% | 93.22 |
| >gi|153952670|ref|NC_009706.1| Clostridium kluyveri DSM 555 chromosome, complete genome | 2615 | 32.02% | 91.78 |
| >gi|188995955|ref|NC_010730.1| Sulfurihydrogenibium sp. YO3AOP1 chromosome, complete genome | 1934 | 32.03% | 87.64 |
| >gi|386858645|ref|NC_017816.1| Borrelia crocidurae str. Achema plasmid unnamed, complete sequence | 5 | 32.03% | 100.00 |
| >gi|289191496|ref|NC_013887.1| Methanocaldococcus sp. FS406-22 chromosome, complete genome | 1048 | 32.04% | 87.88 |
| >gi|384189584|ref|NC_017205.1| Bacillus thuringiensis serovar chinensis CT-43 plasmid pCT72, complete sequence | 57 | 32.04% | 91.23 |
| >gi|402496444|ref|NC_018267.1| Wolbachia endosymbiont of Onchocerca ochengi, complete genome | 606 | 32.07% | 90.76 |
| >gi|385831891|ref|NC_017483.1| Lactococcus lactis subsp. lactis CV56 plasmid pCV56A, complete sequence | 49 | 32.08% | 83.67 |
| >gi|384183674|ref|NC_017202.1| Bacillus thuringiensis serovar chinensis CT-43 plasmid pCT127, complete sequence | 76 | 32.09% | 93.42 |
| >gi|90962708|ref|NC_007930.1| Lactobacillus salivarius UCC118 plasmid pMP118, complete sequence | 140 | 32.09% | 87.14 |
| >gi|27466918|ref|NC_004461.1| Staphylococcus epidermidis ATCC 12228 chromosome, complete genome | 1088 | 32.10% | 92.92 |
| >gi|433653600|ref|NC_019969.1| Prevotella dentalis DSM 3688 plasmid pPREDE02, complete sequence | 6 | 32.10% | 66.67 |
| >gi|325284916|ref|NC_015167.1| Cellulophaga lytica DSM 7489 chromosome, complete genome | 3243 | 32.11% | 90.84 |
| >gi|407708754|ref|NC_018684.1| Bacillus thuringiensis MC28 plasmid pMC8, complete sequence | 3 | 32.11% | 100.00 |
| >gi|386858688|ref|NC_017784.1| Borrelia crocidurae str. Achema plasmid unnamed, complete sequence | 12 | 32.12% | 75.00 |
| >gi|472455754|ref|NC_020530.1| Staphylococcus aureus subsp. aureus ST228 plasmid pI1T1 complete sequence, isolate 10388 | 32 | 32.14% | 90.63 |
| >gi|472455776|ref|NC_020531.1| Staphylococcus aureus subsp. aureus ST228 plasmid pI2T2 complete sequence, isolate 10497 | 32 | 32.14% | 90.63 |
| >gi|378750097|ref|NC_016939.1| Rickettsia massiliae str. AZT80 plasmid pRMB, complete sequence | 6 | 32.14% | 100.00 |
| >gi|469816271|ref|NC_020534.1| Staphylococcus aureus subsp. aureus ST228 plasmid pI4T8 complete sequence, isolate 16035 | 32 | 32.14% | 90.63 |
| >gi|57865352|ref|NC_002976.3| Staphylococcus epidermidis RP62A, complete genome | 1124 | 32.15% | 92.17 |
| >gi|89255449|ref|NC_007880.1| Francisella tularensis subsp. holarctica LVS chromosome, complete genome | 1017 | 32.15% | 93.02 |
| >gi|422937995|ref|NC_019551.1| Francisella tularensis subsp. holarctica FSC200 chromosome, complete genome | 1011 | 32.15% | 93.08 |
| >gi|115313981|ref|NC_008369.1| Francisella tularensis subsp. holarctica OSU18 chromosome, complete genome | 1009 | 32.16% | 92.86 |
| >gi|156501369|ref|NC_009749.1| Francisella tularensis subsp. holarctica FTNF002-00 chromosome, complete genome | 1007 | 32.16% | 92.85 |
| >gi|225871489|ref|NC_012473.1| Bacillus cereus 03BB102 plasmid p03BB102_179, complete sequence | 137 | 32.17% | 89.78 |
| >gi|423049750|ref|NC_019537.1| Francisella tularensis subsp. holarctica F92 chromosome, complete genome | 1004 | 32.17% | 92.83 |
| >gi|381214373|ref|NC_017029.1| Candidatus Rickettsia amblyommii str. GAT-30V plasmid pMCE_2, complete sequence | 9 | 32.19% | 88.89 |
| >gi|409187972|ref|NC_018882.1| Bacillus thuringiensis Bt407 plasmid BTB_7p, complete sequence | 18 | 32.21% | 77.78 |
| >gi|449086547|ref|NC_020243.1| Bacillus thuringiensis serovar kurstaki str. HD73 plasmid pHT7, complete sequence | 18 | 32.21% | 77.78 |
| >gi|256809973|ref|NC_013156.1| Methanocaldococcus fervens AG86 chromosome, complete genome | 983 | 32.21% | 86.88 |
| >gi|387885754|ref|NC_017909.1| Francisella noatunensis subsp. orientalis str. Toba 04 chromosome, complete genome | 1106 | 32.22% | 93.49 |
| >gi|384044079|ref|NC_017140.1| Bacillus megaterium WSH-002 plasmid WSH-002_p2, complete sequence | 14 | 32.22% | 92.86 |
| >gi|60650141|ref|NC_006873.1| Bacteroides fragilis NCTC 9343 plasmid pBF9343, complete sequence | 47 | 32.24% | 82.98 |
| >gi|385831960|ref|NC_017485.1| Lactococcus lactis subsp. lactis CV56 plasmid pCV56D, complete sequence | 9 | 32.24% | 77.78 |
| >gi|187930913|ref|NC_010677.1| Francisella tularensis subsp. mediasiatica FSC147 chromosome, complete genome | 1056 | 32.25% | 92.99 |
| >gi|383500935|ref|NC_017058.1| Rickettsia australis str. Cutlack chromosome, complete genome | 847 | 32.25% | 92.56 |
| >gi|426202350|ref|NT_187141.1| Borrelia hermsii DAH clone fragment 1047R | 12 | 32.26% | 83.33 |
| >gi|379725073|ref|NC_016937.1| Francisella tularensis subsp. tularensis TI0902 chromosome, complete genome | 1055 | 32.26% | 92.99 |
| >gi|255961454|ref|NC_006570.2| Francisella tularensis subsp. tularensis SCHU S4 chromosome, complete genome | 1053 | 32.26% | 92.97 |
| >gi|110669657|ref|NC_008245.1| Francisella tularensis subsp. tularensis FSC198 chromosome, complete genome | 1055 | 32.26% | 92.99 |
| >gi|385793751|ref|NC_017453.1| Francisella tularensis subsp. tularensis NE061598 chromosome, complete genome | 1053 | 32.26% | 92.97 |
| >gi|406707029|ref|NC_018644.1| Alpha proteobacterium HIMB59 chromosome, complete genome | 1839 | 32.26% | 89.40 |
| >gi|134301169|ref|NC_009257.1| Francisella tularensis subsp. tularensis WY96-3418 chromosome, complete genome | 1057 | 32.27% | 93.00 |
| >gi|409187970|ref|NC_018879.1| Bacillus thuringiensis Bt407 plasmid BTB_78p, complete sequence | 59 | 32.29% | 93.22 |
| >gi|472455813|ref|NC_020567.1| Staphylococcus aureus subsp. aureus ST228 plasmid pI6T6 complete sequence, isolate 18341 | 32 | 32.29% | 90.63 |
| >gi|434408556|ref|NC_019766.1| Stanieria cyanosphaera PCC 7437 plasmid pSTA7437.05, complete sequence | 3 | 32.29% | 66.67 |
| >gi|565641727|ref|NC_023029.1| Francisella noatunensis subsp. orientalis LADL--07-285A, complete genome | 1130 | 32.30% | 93.45 |
| >gi|379716390|ref|NC_016933.1| Francisella tularensis TIGB03 chromosome, complete genome | 1085 | 32.30% | 93.09 |
| >gi|333909894|ref|NC_015562.1| Methanotorris igneus Kol 5 chromosome, complete genome | 1137 | 32.30% | 86.46 |
| >gi|554639511|ref|NC_022602.1| Carnobacterium sp. WN1359 plasmid pWNCR47, complete sequence | 52 | 32.31% | 90.38 |
| >gi|469823627|ref|NC_020565.1| Staphylococcus aureus subsp. aureus ST228 plasmid pI3T3 complete sequence, isolate 15532 | 32 | 32.31% | 90.63 |
| >gi|169823697|ref|NC_010376.1| Finegoldia magna ATCC 29328 chromosome, complete genome | 1303 | 32.31% | 89.03 |
| >gi|341583222|ref|NC_015866.1| Rickettsia heilongjiangensis 054 chromosome, complete genome | 815 | 32.32% | 92.64 |
| >gi|387823583|ref|NC_017449.1| Francisella cf. novicida 3523 chromosome, complete genome | 1187 | 32.32% | 92.33 |
| >gi|157825125|ref|NC_009881.1| Rickettsia akari str. Hartford chromosome, complete genome | 755 | 32.33% | 92.19 |
| >gi|350273053|ref|NC_016050.1| Rickettsia japonica YH, complete genome | 810 | 32.35% | 93.09 |
| >gi|451811876|ref|NC_020284.1| Candidatus Kinetoplastibacterium galatii TCC219, complete genome | 573 | 32.36% | 90.58 |
| >gi|325300726|ref|NC_015165.1| Bacteroides salanitronis DSM 18170 plasmid pBACSA01, complete sequence | 38 | 32.37% | 86.84 |
| >gi|281427968|ref|NC_013657.1| Lactococcus lactis subsp. lactis KF147 plasmid pKF147A, complete sequence | 38 | 32.38% | 81.58 |
| >gi|379794527|ref|NC_016941.1| Staphylococcus aureus subsp. aureus MSHR1132, complete genome | 1061 | 32.39% | 90.57 |
| >gi|229586230|ref|NC_012633.1| Rickettsia africae ESF-5 chromosome, complete genome | 791 | 32.40% | 92.41 |
| >gi|414073260|ref|NC_019431.1| Lactococcus lactis subsp. cremoris UC509.9 plasmid pCIS7, complete sequence | 54 | 32.40% | 94.44 |
| >gi|383480870|ref|NC_017042.1| Rickettsia rhipicephali str. 3-7-female6-CWPP chromosome, complete genome | 858 | 32.41% | 92.42 |
| >gi|189485779|ref|NS_000194.1| Uncultured Termite group 1 bacterium phylotype Rs-D17 plasmid pTGRD3 DNA, complete sequence | 4 | 32.41% | 75.00 |
| >gi|455743340|ref|NC_020422.1| Uncultured Termite group 1 bacterium phylotype Rs-D17 plasmid pTGRD3 DNA, complete sequence | 4 | 32.41% | 75.00 |
| >gi|384183665|ref|NC_017211.1| Bacillus thuringiensis serovar chinensis CT-43 plasmid pCT8252, complete sequence | 14 | 32.42% | 85.71 |
| >gi|452202001|ref|NC_020377.1| Bacillus thuringiensis serovar thuringiensis str. IS5056 plasmid pIS56-8, complete sequence | 15 | 32.42% | 86.67 |
| >gi|479328021|ref|NC_021059.1| Staphylococcus aureus M1 complete genome | 1010 | 32.42% | 90.40 |
| >gi|383311892|ref|NC_017028.1| Candidatus Rickettsia amblyommii str. GAT-30V chromosome, complete genome | 881 | 32.43% | 91.94 |
| >gi|383483341|ref|NC_017044.1| Rickettsia parkeri str. Portsmouth chromosome, complete genome | 826 | 32.43% | 92.13 |
| >gi|15891923|ref|NC_003103.1| Rickettsia conorii str. Malish 7, complete genome | 797 | 32.44% | 91.97 |
| >gi|310780578|ref|NC_014634.1| Ilyobacter polytropus DSM 2926 plasmid pILYOP02, complete sequence | 131 | 32.44% | 87.02 |
| >gi|378722019|ref|NC_016909.1| Rickettsia rickettsii str. Arizona chromosome, complete genome | 777 | 32.44% | 92.41 |
| >gi|387826486|ref|NC_017408.1| Borrelia burgdorferi JD1 plasmid JD1 lp28-6, complete sequence | 45 | 32.44% | 93.33 |
| >gi|319717301|ref|NC_010263.2| Rickettsia rickettsii str. Iowa chromosome, complete genome | 781 | 32.45% | 92.32 |
| >gi|67458392|ref|NC_007109.1| Rickettsia felis URRWXCal2 chromosome, complete genome | 947 | 32.45% | 91.76 |
| >gi|379015834|ref|NC_016913.1| Rickettsia rickettsii str. Brazil chromosome, complete genome | 764 | 32.45% | 92.02 |
| >gi|378723377|ref|NC_016911.1| Rickettsia rickettsii str. Hauke chromosome, complete genome | 780 | 32.45% | 92.31 |
| >gi|379017167|ref|NC_016914.1| Rickettsia rickettsii str. Hino chromosome, complete genome | 781 | 32.45% | 92.32 |
| >gi|435852812|ref|NC_019978.1| Halobacteroides halobius DSM 5150, complete genome | 1001 | 32.46% | 91.01 |
| >gi|378720668|ref|NC_016908.1| Rickettsia rickettsii str. Colombia chromosome, complete genome | 775 | 32.46% | 92.52 |
| >gi|157827862|ref|NC_009882.1| Rickettsia rickettsii str. 'Sheila Smith' chromosome, complete genome | 773 | 32.47% | 92.24 |
| >gi|379711742|ref|NC_016930.1| Rickettsia philipii str. 364D chromosome, complete genome | 781 | 32.47% | 92.70 |
| >gi|379018503|ref|NC_016915.1| Rickettsia rickettsii str. Hlp#2 chromosome, complete genome | 785 | 32.47% | 92.23 |
| >gi|118496615|ref|NC_008601.1| Francisella novicida U112 chromosome, complete genome | 1082 | 32.48% | 93.07 |
| >gi|407702534|ref|NC_018688.1| Bacillus thuringiensis MC28 plasmid pMC319, complete sequence | 187 | 32.48% | 90.91 |
| >gi|385829559|ref|NC_017484.1| Lactococcus lactis subsp. lactis CV56 plasmid pCV56C, complete sequence | 32 | 32.49% | 81.25 |
| >gi|374318767|ref|NC_016639.1| Rickettsia slovaca 13-B chromosome, complete genome | 823 | 32.50% | 92.10 |
| >gi|383750641|ref|NC_017065.1| Rickettsia slovaca str. D-CWPP chromosome, complete genome | 823 | 32.50% | 92.10 |
| >gi|402552801|ref|NC_018493.1| Bacillus cereus FRI-35 plasmid p02, complete sequence | 62 | 32.52% | 88.71 |
| >gi|229599713|ref|NC_012656.1| Bacillus anthracis str. A0248 plasmid pXO1, complete sequence | 120 | 32.53% | 88.33 |
| >gi|21392688|ref|NC_003980.1| Bacillus anthracis str. A2012 plasmid pXO1, complete sequence | 120 | 32.53% | 88.33 |
| >gi|47566322|ref|NC_007322.2| Bacillus anthracis str. 'Ames Ancestor' plasmid pXO1, complete sequence | 120 | 32.53% | 88.33 |
| >gi|386733559|ref|NC_017726.1| Bacillus anthracis str. H9401 plasmid BAP1, complete sequence | 120 | 32.53% | 88.33 |
| >gi|227811427|ref|NC_012579.1| Bacillus anthracis str. CDC 684 plasmid pX01, complete sequence | 119 | 32.53% | 88.24 |
| >gi|511542232|ref|NC_009613.3| Flavobacterium psychrophilum JIP02/86 complete genome | 3471 | 32.53% | 88.65 |
| >gi|385791932|ref|NC_017450.1| Francisella cf. novicida Fx1 chromosome, complete genome | 1106 | 32.54% | 92.50 |
| >gi|301068040|ref|NC_014331.1| Bacillus cereus biovar anthracis str. CI plasmid pCI-XO1, complete sequence | 116 | 32.54% | 88.79 |
| >gi|157964072|ref|NC_009900.1| Rickettsia massiliae MTU5 chromosome, complete genome | 888 | 32.54% | 93.13 |
| >gi|437999533|ref|NC_019814.1| Candidatus Kinetoplastibacterium blastocrithidii (ex Strigomonas culicis) chromosome, complete genome | 490 | 32.55% | 91.22 |
| >gi|451812604|ref|NC_020285.1| Candidatus Kinetoplastibacterium blastocrithidii TCC012E, complete genome | 489 | 32.55% | 91.21 |
| >gi|238650213|ref|NC_012730.1| Rickettsia peacockii str. Rustic, complete genome | 770 | 32.56% | 91.95 |
| >gi|407702824|ref|NC_018689.1| Bacillus thuringiensis MC28 plasmid pMC429, complete sequence | 297 | 32.57% | 89.23 |
| >gi|383482123|ref|NC_017043.1| Rickettsia montanensis str. OSU 85-930 chromosome, complete genome | 838 | 32.57% | 92.12 |
| >gi|167626225|ref|NC_010336.1| Francisella philomiragia subsp. philomiragia ATCC 25017 chromosome, complete genome | 1102 | 32.57% | 93.28 |
| >gi|452202391|ref|NC_020385.1| Bacillus thuringiensis serovar thuringiensis str. IS5056 plasmid pIS56-328, complete sequence | 230 | 32.59% | 90.87 |
| >gi|469820173|ref|NC_020525.1| Acinetobacter baumannii D1279779 plasmid pD1279779, complete sequence | 13 | 32.59% | 92.31 |
| >gi|379713087|ref|NC_016931.1| Rickettsia massiliae str. AZT80 chromosome, complete genome | 794 | 32.61% | 92.95 |
| >gi|56899872|ref|NC_006578.1| Bacillus thuringiensis serovar konkukian str. 97-27 plasmid pBT9727, complete sequence | 57 | 32.64% | 91.23 |
| >gi|288559258|ref|NC_013790.1| Methanobrevibacter ruminantium M1 chromosome, complete genome | 3518 | 32.64% | 87.98 |
| >gi|384519756|ref|NC_017732.1| Enterococcus phage EF62phi, complete genome | 16 | 32.66% | 100.00 |
| >gi|212550357|ref|NC_011565.1| Candidatus Azobacteroides pseudotrichonymphae genomovar. CFP2 chromosome, complete genome | 1429 | 32.66% | 91.04 |
| >gi|387826782|ref|NC_017404.1| Borrelia burgdorferi JD1 plasmid JD1 lp28-1, complete sequence | 36 | 32.67% | 91.67 |
| >gi|556589855|ref|NC_022737.1| Staphylococcus pasteuri SP1, complete genome | 855 | 32.71% | 91.58 |
| >gi|222142646|ref|NC_011997.1| Macrococcus caseolyticus JCSC5402 plasmid pMCCL3, complete sequence | 3 | 32.71% | 66.67 |
| >gi|426202358|ref|NT_187142.1| Borrelia hermsii DAH clone fragment 1050 | 15 | 32.71% | 86.67 |
| >gi|452196180|ref|NC_020394.1| Bacillus thuringiensis serovar thuringiensis str. IS5056 plasmid pIS56-233, complete sequence | 125 | 32.71% | 93.60 |
| >gi|386747020|ref|NC_017736.1| Helicobacter cetorum MIT 99-5656 plasmid pHCD, complete sequence | 20 | 32.72% | 100.00 |
| >gi|445058371|ref|NC_020164.1| Staphylococcus warneri SG1, complete genome | 746 | 32.73% | 91.29 |
| >gi|365823337|ref|NC_001851.2| Borrelia burgdorferi B31 plasmid lp28-1, complete sequence | 45 | 32.75% | 91.11 |
| >gi|87159884|ref|NC_007793.1| Staphylococcus aureus subsp. aureus USA300_FPR3757 chromosome, complete genome | 1045 | 32.75% | 91.10 |
| >gi|225847840|ref|NC_012438.1| Sulfurihydrogenibium azorense Az-Fu1 chromosome, complete genome | 1691 | 32.75% | 87.88 |
| >gi|161508266|ref|NC_010079.1| Staphylococcus aureus subsp. aureus USA300_TCH1516 chromosome, complete genome | 1036 | 32.76% | 91.02 |
| >gi|426202317|ref|NT_187136.1| Borrelia hermsii DAH clone fragment 1025rc | 7 | 32.77% | 71.43 |
| >gi|82749777|ref|NC_007622.1| Staphylococcus aureus RF122, complete genome | 1005 | 32.78% | 89.55 |
| >gi|387141638|ref|NC_017331.1| Staphylococcus aureus subsp. aureus TW20, complete genome | 1082 | 32.78% | 90.57 |
| >gi|537441500|ref|NC_022222.1| Staphylococcus aureus subsp. aureus 6850, complete genome | 992 | 32.78% | 90.22 |
| >gi|256811520|ref|NC_013157.1| Methanocaldococcus fervens AG86 plasmid pMEFER01, complete sequence | 25 | 32.79% | 80.00 |
| >gi|24473558|ref|NC_003106.2| Sulfolobus tokodaii str. 7 chromosome, complete genome | 1487 | 32.79% | 89.58 |
| >gi|70725001|ref|NC_007168.1| Staphylococcus haemolyticus JCSC1435 chromosome, complete genome | 960 | 32.79% | 92.92 |
| >gi|537459744|ref|NC_022226.1| Staphylococcus aureus subsp. aureus CN1, complete genome | 988 | 32.80% | 90.49 |
| >gi|406598909|ref|NC_018637.1| Chlamydia psittaci NJ1 plasmid pcpNJ1, complete sequence | 11 | 32.80% | 81.82 |
| >gi|384868588|ref|NC_017347.1| Staphylococcus aureus subsp. aureus T0131 chromosome, complete genome | 1034 | 32.80% | 90.33 |
| >gi|384548923|ref|NC_017338.1| Staphylococcus aureus subsp. aureus JKD6159 chromosome, complete genome | 1002 | 32.81% | 90.22 |
| >gi|49482253|ref|NC_002952.2| Staphylococcus aureus subsp. aureus MRSA252 chromosome, complete genome | 1085 | 32.81% | 89.40 |
| >gi|532358222|ref|NC_022113.1| Staphylococcus aureus subsp. aureus 55/2053, complete genome | 982 | 32.81% | 90.43 |
| >gi|379013365|ref|NC_016912.1| Staphylococcus aureus subsp. aureus VC40 chromosome, complete genome | 946 | 32.81% | 90.80 |
| >gi|406598874|ref|NC_018633.1| Chlamydia psittaci 84/55 plasmid pcp8455, complete sequence | 15 | 32.82% | 80.00 |
| >gi|57650036|ref|NC_002951.2| Staphylococcus aureus subsp. aureus COL chromosome, complete genome | 1004 | 32.82% | 90.74 |
| >gi|21281729|ref|NC_003923.1| Staphylococcus aureus subsp. aureus MW2, complete genome | 1033 | 32.83% | 90.22 |
| >gi|386829725|ref|NC_017763.1| Staphylococcus aureus subsp. aureus HO 5096 0412, complete genome | 1041 | 32.83% | 90.39 |
| >gi|554642795|ref|NC_022604.1| Staphylococcus aureus subsp. aureus Z172, complete genome | 1052 | 32.84% | 90.49 |
| >gi|269201690|ref|NC_013450.1| Staphylococcus aureus subsp. aureus ED98, complete genome | 998 | 32.84% | 90.28 |
| >gi|29165615|ref|NC_002745.2| Staphylococcus aureus subsp. aureus N315 chromosome, complete genome | 1002 | 32.84% | 89.72 |
| >gi|407702137|ref|NC_018686.1| Bacillus thuringiensis MC28 plasmid pMC183, complete sequence | 111 | 32.85% | 88.29 |
| >gi|470194245|ref|NC_020533.1| Staphylococcus aureus subsp. aureus ST228 complete genome, isolate 16035 | 962 | 32.85% | 89.81 |
| >gi|470192280|ref|NC_020532.1| Staphylococcus aureus subsp. aureus ST228 complete genome, isolate 15532 | 967 | 32.85% | 89.76 |
| >gi|49484912|ref|NC_002953.3| Staphylococcus aureus subsp. aureus MSSA476 chromosome, complete genome | 1025 | 32.85% | 90.24 |
| >gi|470221770|ref|NC_020566.1| Staphylococcus aureus subsp. aureus ST228 complete genome, isolate 16125 | 968 | 32.85% | 89.77 |
| >gi|470190315|ref|NC_020529.1| Staphylococcus aureus subsp. aureus ST228 complete genome, isolate 10388 | 966 | 32.85% | 89.75 |
| >gi|470217946|ref|NC_020564.1| Staphylococcus aureus subsp. aureus ST228 complete genome, isolate 10497 | 966 | 32.85% | 89.75 |
| >gi|470196211|ref|NC_020536.1| Staphylococcus aureus subsp. aureus ST228 complete genome, isolate 18341 | 966 | 32.85% | 89.75 |
| >gi|470223734|ref|NC_020568.1| Staphylococcus aureus subsp. aureus ST228 complete genome, isolate 18583 | 966 | 32.85% | 89.75 |
| >gi|470198176|ref|NC_020537.1| Staphylococcus aureus subsp. aureus ST228 complete genome, isolate 18412 | 967 | 32.85% | 89.76 |
| >gi|545633528|ref|NC_022442.1| Staphylococcus aureus subsp. aureus SA957, complete genome | 1023 | 32.85% | 90.03 |
| >gi|379019812|ref|NC_016928.1| Staphylococcus aureus subsp. aureus M013 chromosome, complete genome | 1015 | 32.85% | 90.15 |
| >gi|384863396|ref|NC_017343.1| Staphylococcus aureus subsp. aureus ECT-R 2, complete genome | 963 | 32.86% | 90.24 |
| >gi|347534971|ref|NC_016001.1| Flavobacterium branchiophilum FL-15, complete genome | 5517 | 32.86% | 85.41 |
| >gi|545636471|ref|NC_022443.1| Staphylococcus aureus subsp. aureus SA40, complete genome | 984 | 32.86% | 89.94 |
| >gi|410858954|ref|NC_019392.1| Chlamydia psittaci 01DC12 plasmid p01DC12, complete sequence | 14 | 32.86% | 78.57 |
| >gi|337754017|ref|NC_015696.1| Francisella sp. TX077308 chromosome, complete genome | 1145 | 32.87% | 93.54 |
| >gi|88193823|ref|NC_007795.1| Staphylococcus aureus subsp. aureus NCTC 8325 chromosome, complete genome | 976 | 32.87% | 90.47 |
| >gi|384865886|ref|NC_017342.1| Staphylococcus aureus subsp. aureus TCH60 chromosome, complete genome | 1003 | 32.87% | 90.53 |
| >gi|385780298|ref|NC_017351.1| Staphylococcus aureus subsp. aureus 11819-97 chromosome, complete genome | 1014 | 32.88% | 90.34 |
| >gi|156978331|ref|NC_009782.1| Staphylococcus aureus subsp. aureus Mu3, complete genome | 1011 | 32.88% | 90.01 |
| >gi|57634611|ref|NC_002758.2| Staphylococcus aureus subsp. aureus Mu50 chromosome, complete genome | 1008 | 32.88% | 89.98 |
| >gi|387144420|ref|NC_017352.1| Staphylococcus aureus subsp. aureus TW20 plasmid pTW20_1, complete sequence | 28 | 32.88% | 85.71 |
| >gi|406598883|ref|NC_018634.1| Chlamydia psittaci CP3 plasmid pcpCP3, complete sequence | 14 | 32.88% | 78.57 |
| >gi|384888493|ref|NC_017383.1| Helicobacter pylori v225d plasmid pHPv225d, complete sequence | 9 | 32.88% | 100.00 |
| >gi|384860682|ref|NC_017341.1| Staphylococcus aureus subsp. aureus str. JKD6008 chromosome, complete genome | 1042 | 32.89% | 90.12 |
| >gi|406598892|ref|NC_018635.1| Chlamydia psittaci M56 plasmid pcpM56, complete sequence | 13 | 32.89% | 76.92 |
| >gi|384451098|ref|NC_017288.1| Chlamydophila psittaci 6BC plasmid pCps6BC, complete sequence | 15 | 32.89% | 80.00 |
| >gi|332286950|ref|NC_015217.1| Chlamydophila psittaci 6BC plasmid p6BC, complete sequence | 14 | 32.89% | 78.57 |
| >gi|409191175|ref|NC_014797.1| Chlamydophila psittaci RD1 plasmid pRD1, complete sequence | 14 | 32.89% | 78.57 |
| >gi|151220212|ref|NC_009641.1| Staphylococcus aureus subsp. aureus str. Newman chromosome, complete genome | 1007 | 32.89% | 90.67 |
| >gi|387149188|ref|NC_017340.1| Staphylococcus aureus 04-02981 chromosome, complete genome | 996 | 32.90% | 89.96 |
| >gi|308183744|ref|NC_014556.1| Helicobacter pylori PeCan4 plasmid pHPPC4, complete sequence | 7 | 32.90% | 85.71 |
| >gi|385839818|ref|NC_017481.1| Lactobacillus salivarius CECT 5713 chromosome, complete genome | 1003 | 32.90% | 91.13 |
| >gi|218847808|ref|NC_011774.1| Bacillus cereus G9842 plasmid pG9842_140, complete sequence | 71 | 32.90% | 85.92 |
| >gi|442556535|ref|NC_020130.1| Lawsonia intracellularis N343 plasmid 3, complete sequence | 254 | 32.91% | 89.76 |
| >gi|94972398|ref|NC_008014.1| Lawsonia intracellularis PHE/MN1-00 plasmid 3, complete sequence | 254 | 32.91% | 89.76 |
| >gi|406598918|ref|NC_018638.1| Chlamydia psittaci VS225 plasmid pcpVS225, complete sequence | 15 | 32.91% | 80.00 |
| >gi|521210823|ref|NC_021670.1| Staphylococcus aureus Bmb9393, complete genome | 1043 | 32.92% | 90.22 |
| >gi|384546269|ref|NC_017337.1| Staphylococcus aureus subsp. aureus ED133 chromosome, complete genome | 964 | 32.92% | 89.94 |
| >gi|387601291|ref|NC_017333.1| Staphylococcus aureus subsp. aureus ST398, complete genome | 1056 | 32.92% | 90.15 |
| >gi|406598927|ref|NC_018639.1| Chlamydia psittaci WC plasmid pcpWC, complete sequence | 15 | 32.93% | 80.00 |
| >gi|557624419|ref|NC_022782.1| Bacillus toyonensis BCT-7112 plasmid pBCT77, complete sequence | 86 | 32.93% | 86.05 |
| >gi|406598901|ref|NC_018636.1| Chlamydia psittaci MN plasmid pcpMN, complete sequence | 14 | 32.93% | 78.57 |
| >gi|386727822|ref|NC_017673.1| Staphylococcus aureus subsp. aureus 71193 chromosome, complete genome | 988 | 32.94% | 90.28 |
| >gi|404477334|ref|NC_018608.1| Staphylococcus aureus 08BA02176 chromosome, complete genome | 1011 | 32.94% | 90.21 |
| >gi|406598936|ref|NC_018640.1| Chlamydia psittaci WS/RT/E30 plasmid pcpWSRTE30, complete sequence | 14 | 32.94% | 78.57 |
| >gi|340623184|ref|NC_015847.1| Methanococcus maripaludis X1 chromosome, complete genome | 1892 | 32.94% | 86.10 |
| >gi|90960990|ref|NC_007929.1| Lactobacillus salivarius UCC118 chromosome, complete genome | 984 | 32.94% | 91.06 |
| >gi|148266447|ref|NC_009487.1| Staphylococcus aureus subsp. aureus JH9 chromosome, complete genome | 1016 | 32.95% | 89.96 |
| >gi|150392480|ref|NC_009632.1| Staphylococcus aureus subsp. aureus JH1 chromosome, complete genome | 1014 | 32.95% | 89.94 |
| >gi|514064966|ref|NC_021554.1| Staphylococcus aureus CA-347, complete genome | 999 | 32.95% | 90.49 |
| >gi|387779217|ref|NC_017349.1| Staphylococcus aureus subsp. aureus LGA251, complete genome | 935 | 32.96% | 90.70 |
| >gi|402558636|ref|NC_018486.1| Bacillus thuringiensis HD-771 plasmid p01, complete sequence | 106 | 32.97% | 90.57 |
| >gi|294505704|ref|NC_014031.1| Bacillus megaterium QM B1551 plasmid pBM600, complete sequence | 70 | 32.97% | 87.14 |
| >gi|554652311|ref|NC_022610.1| Staphylococcus aureus subsp. aureus Z172 plasmid pZ172_1, complete sequence | 31 | 32.97% | 90.32 |
| >gi|425790159|ref|NC_019561.1| Helicobacter pylori Aklavik117 plasmid p1HPAKL117, complete sequence | 14 | 33.00% | 85.71 |
| >gi|134045046|ref|NC_009135.1| Methanococcus maripaludis C5 chromosome, complete genome | 1871 | 33.00% | 87.17 |
| >gi|170016273|ref|NC_010467.1| Leuconostoc citreum KM20 plasmid pLCK3, complete sequence | 10 | 33.00% | 90.00 |
| >gi|384183816|ref|NC_017203.1| Bacillus thuringiensis serovar chinensis CT-43 plasmid pCT281, complete sequence | 150 | 33.01% | 92.00 |
| >gi|452195884|ref|NC_020384.1| Bacillus thuringiensis serovar thuringiensis str. IS5056 plasmid pIS56-285, complete sequence | 150 | 33.02% | 92.00 |
| >gi|384450086|ref|NC_017286.1| Chlamydophila pneumoniae LPCoLN plasmid unnamed, complete sequence | 11 | 33.03% | 100.00 |
| >gi|452202110|ref|NC_020378.1| Bacillus thuringiensis serovar thuringiensis str. IS5056 plasmid pIS56-9, complete sequence | 8 | 33.04% | 87.50 |
| >gi|229599623|ref|NC_012655.1| Bacillus anthracis str. A0248 plasmid pXO2, complete sequence | 59 | 33.04% | 88.14 |
| >gi|50163691|ref|NC_007323.3| Bacillus anthracis str. 'Ames Ancestor' plasmid pXO2, complete sequence | 59 | 33.04% | 88.14 |
| >gi|21392893|ref|NC_003981.1| Bacillus anthracis str. A2012 plasmid pXO2, complete sequence | 59 | 33.04% | 88.14 |
| >gi|434379567|ref|NC_018510.1| Bacillus thuringiensis HD-789 plasmid p03, complete sequence | 143 | 33.05% | 95.10 |
| >gi|227811309|ref|NC_012577.1| Bacillus anthracis str. CDC 684 plasmid pX02, complete sequence | 59 | 33.05% | 88.14 |
| >gi|386733762|ref|NC_017727.1| Bacillus anthracis str. H9401 plasmid BAP2, complete sequence | 59 | 33.05% | 88.14 |
| >gi|222142648|ref|NC_011998.1| Macrococcus caseolyticus JCSC5402 plasmid pMCCL4, complete sequence | 0 | 33.07% | 0.00 |
| >gi|384189578|ref|NC_017212.1| Bacillus thuringiensis serovar chinensis CT-43 plasmid pCT9547, complete sequence | 7 | 33.09% | 85.71 |
| >gi|301067930|ref|NC_014332.1| Bacillus cereus biovar anthracis str. CI plasmid pCI-XO2, complete sequence | 58 | 33.09% | 87.93 |
| >gi|45357563|ref|NC_005791.1| Methanococcus maripaludis S2 chromosome, complete genome | 1775 | 33.10% | 86.20 |
| >gi|169825414|ref|NC_010381.1| Lysinibacillus sphaericus C3-41 plasmid pBsph, complete sequence | 93 | 33.10% | 89.25 |
| >gi|384183356|ref|NC_017201.1| Bacillus thuringiensis serovar finitimus YBT-020 plasmid pBMB26, complete sequence | 111 | 33.11% | 85.59 |
| >gi|67077889|ref|NC_007103.1| Bacillus cereus E33L plasmid pE33L466, complete sequence | 302 | 33.12% | 91.06 |
| >gi|557235156|ref|NC_022759.1| Campylobacter fetus subsp. testudinum 03-427 chromosome, complete genome | 1328 | 33.12% | 90.66 |
| >gi|384189671|ref|NC_017206.1| Bacillus thuringiensis serovar chinensis CT-43 plasmid pCT83, complete sequence | 70 | 33.15% | 90.00 |
| >gi|312142408|ref|NC_014654.1| Halanaerobium hydrogeniformans chromosome, complete genome | 2345 | 33.16% | 89.98 |
| >gi|428771774|ref|NC_019777.1| Cyanobacterium aponinum PCC 10605 plasmid pCYAN10605.01, complete sequence | 32 | 33.17% | 84.38 |
| >gi|402558404|ref|NC_018501.1| Bacillus thuringiensis HD-771 plasmid p02, complete sequence | 109 | 33.17% | 88.99 |
| >gi|67459862|ref|NC_007111.1| Rickettsia felis URRWXCal2 plasmid pRFdelta, complete sequence | 15 | 33.19% | 93.33 |
| >gi|452202278|ref|NC_020383.1| Bacillus thuringiensis serovar thuringiensis str. IS5056 plasmid pIS56-85, complete sequence | 70 | 33.21% | 90.00 |
| >gi|384044091|ref|NC_017141.1| Bacillus megaterium WSH-002 plasmid WSH-002_p3, complete sequence | 1 | 33.21% | 100.00 |
| >gi|94676460|ref|NC_007984.1| Baumannia cicadellinicola str. Hc (Homalodisca coagulata), complete genome | 360 | 33.23% | 94.44 |
| >gi|73661309|ref|NC_007350.1| Staphylococcus saprophyticus subsp. saprophyticus ATCC 15305, complete genome | 967 | 33.24% | 92.14 |
| >gi|426202324|ref|NT_187137.1| Borrelia hermsii DAH clone fragment 1034rc | 5 | 33.26% | 100.00 |
| >gi|296506382|ref|NC_014172.1| Bacillus thuringiensis BMB171 plasmid pBMB171, complete sequence | 185 | 33.27% | 93.51 |
| >gi|340620745|ref|NC_015845.1| Enterococcus hirae ATCC 9790 plasmid pTG9790, complete sequence | 23 | 33.27% | 95.65 |
| >gi|150401930|ref|NC_009637.1| Methanococcus maripaludis C7 chromosome, complete genome | 1897 | 33.28% | 86.35 |
| >gi|94986445|ref|NC_008011.1| Lawsonia intracellularis PHE/MN1-00 chromosome, complete genome | 1181 | 33.28% | 90.77 |
| >gi|442555261|ref|NC_020127.1| Lawsonia intracellularis N343, complete genome | 1180 | 33.29% | 90.76 |
| >gi|261876182|ref|NC_013438.1| Aggregatibacter actinomycetemcomitans plasmid S25, complete sequence | 22 | 33.29% | 81.82 |
| >gi|310657316|ref|NC_014614.1| [Clostridium] sticklandii, complete genome | 1684 | 33.30% | 91.15 |
| >gi|118474057|ref|NC_008599.1| Campylobacter fetus subsp. fetus 82-40 chromosome, complete genome | 1263 | 33.31% | 90.58 |
| >gi|29377876|ref|NC_004670.1| Enterococcus faecalis V583 plasmid pTEF3, complete sequence | 8 | 33.32% | 75.00 |
| >gi|452202118|ref|NC_020380.1| Bacillus thuringiensis serovar thuringiensis str. IS5056 plasmid pIS56-16, complete sequence | 17 | 33.34% | 94.12 |
| >gi|385239413|ref|NC_017166.1| Acinetobacter baumannii TCDC-AB0715 plasmid p2ABTCDC0715, complete sequence | 59 | 33.34% | 88.14 |
| >gi|163943459|ref|NC_010181.1| Bacillus weihenstephanensis KBAB4 plasmid pBWB402, complete sequence | 64 | 33.35% | 89.06 |
| >gi|434379898|ref|NC_018516.1| Bacillus thuringiensis HD-789 plasmid p01, complete sequence | 107 | 33.35% | 85.98 |
| >gi|513842865|ref|NC_021515.1| Lactobacillus plantarum 16 plasmid Lp16A, complete sequence | 8 | 33.37% | 87.50 |
| >gi|148245107|ref|NC_009466.1| Clostridium kluyveri DSM 555 plasmid pCKL555A, complete sequence | 33 | 33.37% | 84.85 |
| >gi|219684028|ref|NC_011836.1| Clostridium kluyveri NBRC 12016 plasmid pCKL1, complete sequence | 31 | 33.37% | 83.87 |
| >gi|383327284|ref|NC_017021.1| Candidatus Rickettsia amblyommii str. GAT-30V plasmid pMCE_3, complete sequence | 6 | 33.40% | 100.00 |
| >gi|184160017|ref|NC_010606.1| Acinetobacter baumannii ACICU plasmid pACICU2, complete sequence | 53 | 33.40% | 84.91 |
| >gi|407702348|ref|NC_018687.1| Bacillus thuringiensis MC28 plasmid pMC189, complete sequence | 114 | 33.41% | 85.96 |
| >gi|402557992|ref|NC_018492.1| Bacillus cereus FRI-35 plasmid p01, complete sequence | 99 | 33.41% | 91.92 |
| >gi|159904396|ref|NC_009975.1| Methanococcus maripaludis C6 chromosome, complete genome | 1852 | 33.42% | 86.56 |
| >gi|44004339|ref|NC_005707.1| Bacillus cereus ATCC 10987 plasmid pBc10987, complete sequence | 83 | 33.44% | 91.57 |
| >gi|269118642|ref|NC_013517.1| Sebaldella termitidis ATCC 33386 chromosome, complete genome | 5172 | 33.45% | 88.52 |
| >gi|157149612|ref|NC_009796.1| Campylobacter concisus 13826 plasmid pCCON16, complete sequence | 16 | 33.46% | 81.25 |
| >gi|228478451|ref|NC_012634.1| Rickettsia africae ESF-5 plasmid pRAF, complete sequence | 2 | 33.46% | 100.00 |
| >gi|384133685|ref|NC_017163.1| Acinetobacter baumannii 1656-2 plasmid ABKp1, complete sequence | 52 | 33.47% | 86.54 |
| >gi|409183164|ref|NC_018751.1| Flavobacterium branchiophilum FL-15 plasmid pFB1, complete sequence | 12 | 33.48% | 75.00 |
| >gi|294508916|ref|NC_014023.1| Bacillus megaterium QM B1551 plasmid pBM700, complete sequence | 131 | 33.49% | 88.55 |
| >gi|221642090|ref|NC_011973.1| Bacillus cereus Q1 plasmid pBc239, complete sequence | 141 | 33.49% | 92.91 |
| >gi|402558241|ref|NC_018503.1| Bacillus thuringiensis HD-771 plasmid p07, complete sequence | 9 | 33.50% | 77.78 |
| >gi|384133676|ref|NC_017164.1| Acinetobacter baumannii 1656-2 plasmid ABKp2, complete sequence | 6 | 33.50% | 100.00 |
| >gi|224372070|ref|NC_012115.1| Nautilia profundicola AmH chromosome, complete genome | 2328 | 33.51% | 87.33 |
| >gi|397698492|ref|NC_018222.1| Enterococcus faecalis D32 plasmid EFD32pA, complete sequence | 7 | 33.51% | 100.00 |
| >gi|384893581|ref|NC_017356.1| Helicobacter pylori Sat464 plasmid pHPSAT464, complete sequence | 25 | 33.52% | 68.00 |
| >gi|52421214|ref|NC_006297.1| Bacteroides fragilis YCH46 plasmid pBFY46, complete sequence | 26 | 33.54% | 92.31 |
| >gi|298501435|ref|NC_014250.1| 'Nostoc azollae' 0708 plasmid pAzo02, complete sequence | 5 | 33.54% | 80.00 |
| >gi|116326645|ref|NC_008507.1| Lactococcus lactis subsp. cremoris SK11 plasmid 5, complete sequence | 18 | 33.55% | 77.78 |
| >gi|67459793|ref|NC_007110.1| Rickettsia felis URRWXCal2 plasmid pRF, complete sequence | 19 | 33.58% | 89.47 |
| >gi|407463877|ref|NC_018656.1| Candidatus Nitrosopumilus sp. AR2 chromosome, complete genome | 971 | 33.59% | 90.63 |
| >gi|73663826|ref|NC_007349.1| Methanosarcina barkeri str. fusaro plasmid 1, complete sequence | 17 | 33.59% | 94.12 |
| >gi|449338649|ref|NC_020269.1| Staphylococcus warneri SG1 plasmid clone pvSw7 genomic sequence | 3 | 33.61% | 100.00 |
| >gi|218847796|ref|NC_011771.1| Bacillus cereus AH820 plasmid pAH820_10, complete sequence | 12 | 33.61% | 83.33 |
| >gi|296108688|ref|NC_014122.1| Methanocaldococcus infernus ME chromosome, complete genome | 795 | 33.62% | 86.16 |
| >gi|298676127|ref|NC_014254.1| Methanohalobium evestigatum Z-7303 plasmid pMETEV01, complete sequence | 90 | 33.64% | 93.33 |
| >gi|218848185|ref|NC_011777.1| Bacillus cereus AH820 plasmid pAH820_272, complete sequence | 126 | 33.64% | 91.27 |
| >gi|163943169|ref|NC_010180.1| Bacillus weihenstephanensis KBAB4 plasmid pBWB401, complete sequence | 236 | 33.67% | 91.53 |
| >gi|558684373|ref|NC_022875.1| Bacillus thuringiensis YBT-1518 plasmid pBMB0230, complete sequence | 39 | 33.67% | 89.74 |
| >gi|384896665|ref|NC_017363.1| Helicobacter pylori Lithuania75 plasmid unnamed, complete sequence | 22 | 33.68% | 72.73 |
| >gi|29839220|ref|NC_004720.1| Chlamydophila caviae GPIC plasmid pCpGP1, complete sequence | 8 | 33.69% | 100.00 |
| >gi|383192418|ref|NC_017092.1| Rahnella aquatilis CIP 78.65 = ATCC 33071 plasmid pRahaq203, complete sequence | 5 | 33.71% | 80.00 |
| >gi|462045533|ref|NC_020274.1| Staphylococcus warneri SG1 plasmid clone pvSw1 genomic sequence | 5 | 33.73% | 100.00 |
| >gi|383479184|ref|NC_017041.1| Rickettsia australis str. Cutlack plasmid pMC5_1, complete sequence | 10 | 33.73% | 80.00 |
| >gi|206900094|ref|NC_011297.1| Dictyoglomus thermophilum H-6-12, complete genome | 1960 | 33.74% | 86.89 |
| >gi|385221474|ref|NC_017373.1| Helicobacter pylori SouthAfrica7 plasmid unnamed, complete sequence | 37 | 33.75% | 75.68 |
| >gi|558687842|ref|NC_022880.1| Enterococcus mundtii QU 25 plasmid pQY039 DNA, complete genome | 39 | 33.75% | 82.05 |
| >gi|449086377|ref|NC_020240.1| Bacillus thuringiensis serovar kurstaki str. HD73 plasmid pAW63, complete sequence | 46 | 33.76% | 89.13 |
| >gi|319951593|ref|NC_014934.1| Cellulophaga algicola DSM 14237 chromosome, complete genome | 3331 | 33.77% | 90.03 |
| >gi|426202328|ref|NT_187138.1| Borrelia hermsii DAH clone fragment 1029 | 10 | 33.78% | 90.00 |
| >gi|425791554|ref|NC_019564.1| Helicobacter pylori Aklavik86 plasmid p1HPAKL86, complete sequence | 10 | 33.79% | 80.00 |
| >gi|385782956|ref|NC_017353.1| Staphylococcus lugdunensis N920143, complete genome | 851 | 33.82% | 92.01 |
| >gi|558684593|ref|NC_022877.1| Bacillus thuringiensis YBT-1518 plasmid pBMB0232, complete sequence | 143 | 33.82% | 92.31 |
| >gi|385829555|ref|NC_017488.1| Lactococcus lactis subsp. lactis CV56 plasmid pCV56E, complete sequence | 8 | 33.82% | 75.00 |
| >gi|523525145|ref|NC_021727.1| Acinetobacter baumannii BJAB07104 plasmid p1BJAB07104, complete sequence | 26 | 33.82% | 92.31 |
| >gi|523529013|ref|NC_021731.1| Acinetobacter baumannii BJAB0868 plasmid p2BJAB0868, complete sequence | 26 | 33.83% | 92.31 |
| >gi|332294861|ref|NC_015499.1| Thermodesulfobium narugense DSM 14796 chromosome, complete genome | 2115 | 33.87% | 89.22 |
| >gi|289549371|ref|NC_013893.1| Staphylococcus lugdunensis HKU09-01 chromosome, complete genome | 876 | 33.87% | 92.12 |
| >gi|384177763|ref|NC_017199.1| Bacillus thuringiensis serovar finitimus YBT-020 plasmid pBMB28, complete sequence | 96 | 33.88% | 92.71 |
| >gi|29377895|ref|NC_004671.1| Enterococcus faecalis V583 plasmid pTEF2, complete sequence | 62 | 33.90% | 85.48 |
| >gi|426202372|ref|NT_187143.1| Borrelia hermsii DAH clone fragment 1051L | 10 | 33.90% | 80.00 |
| >gi|298206591|ref|NC_014230.1| Croceibacter atlanticus HTCC2559 chromosome, complete genome | 1225 | 33.90% | 91.59 |
| >gi|433653743|ref|NC_019970.1| Thermoanaerobacterium thermosaccharolyticum M0795, complete genome | 1573 | 33.90% | 89.64 |
| >gi|558684436|ref|NC_022876.1| Bacillus thuringiensis YBT-1518 plasmid pBMB0231, complete sequence | 108 | 33.90% | 87.04 |
| >gi|294509091|ref|NC_014025.1| Bacillus megaterium QM B1551 plasmid pBM500, complete sequence | 51 | 33.91% | 94.12 |
| >gi|89898813|ref|NC_007900.1| Chlamydophila felis Fe/C-56 plasmid pCfe1, complete sequence | 7 | 33.91% | 85.71 |
| >gi|397677603|ref|NC_018149.1| Mycoplasma wenyonii str. Massachusetts chromosome, complete genome | 668 | 33.92% | 84.43 |
| >gi|385808586|ref|NC_017464.1| Ignavibacterium album JCM 16511 chromosome, complete genome | 2872 | 33.93% | 90.53 |
| >gi|217966449|ref|NC_011661.1| Dictyoglomus turgidum DSM 6724 chromosome, complete genome | 1913 | 33.96% | 86.88 |
| >gi|414073192|ref|NC_019430.1| Lactococcus lactis subsp. cremoris UC509.9 plasmid pCIS8, complete sequence | 76 | 33.97% | 85.53 |
| >gi|383327248|ref|NC_017020.1| Candidatus Rickettsia amblyommii str. GAT-30V plasmid pMCE_1, complete sequence | 7 | 33.97% | 85.71 |
| >gi|336053279|ref|NC_015598.1| Lactobacillus kefiranofaciens ZW3 plasmid pWW1, complete sequence | 106 | 33.98% | 88.68 |
| >gi|408671611|ref|NC_018744.1| Emticicia oligotrophica DSM 17448 plasmid pEMTOL04, complete sequence | 28 | 34.01% | 89.29 |
| >gi|482888170|ref|NC_021084.1| Wolbachia endosymbiont of Drosophila simulans wNo, complete genome | 632 | 34.01% | 87.66 |
| >gi|407702035|ref|NC_018685.1| Bacillus thuringiensis MC28 plasmid pMC95, complete sequence | 61 | 34.02% | 91.80 |
| >gi|118602060|ref|NC_008610.1| Candidatus Ruthia magnifica str. Cm (Calyptogena magnifica), complete genome | 638 | 34.03% | 92.48 |
| >gi|387125794|ref|NC_017848.1| Acinetobacter baumannii MDR-TJ plasmid pABTJ1, complete sequence | 28 | 34.05% | 92.86 |
| >gi|414073302|ref|NC_019432.1| Lactococcus lactis subsp. cremoris UC509.9 plasmid pCIS5, complete sequence | 18 | 34.06% | 77.78 |
| >gi|408671447|ref|NC_018742.1| Emticicia oligotrophica DSM 17448 plasmid pEMTOL01, complete sequence | 137 | 34.09% | 91.97 |
| >gi|304315537|ref|NC_014410.1| Thermoanaerobacterium thermosaccharolyticum DSM 571 chromosome, complete genome | 1526 | 34.11% | 89.58 |
| >gi|146297766|ref|NC_009441.1| Flavobacterium johnsoniae UW101 chromosome, complete genome | 5640 | 34.11% | 89.10 |
| >gi|160901491|ref|NC_010003.1| Petrotoga mobilis SJ95 chromosome, complete genome | 2162 | 34.12% | 87.00 |
| >gi|206889172|ref|NC_011296.1| Thermodesulfovibrio yellowstonii DSM 11347 chromosome, complete genome | 2155 | 34.13% | 88.82 |
| >gi|385218260|ref|NC_017369.1| Helicobacter pylori F30 plasmid pHPF30, complete sequence | 13 | 34.13% | 84.62 |
| >gi|375287575|ref|NC_016773.1| Bacillus cereus NC7401 plasmid pNC2, complete sequence | 10 | 34.14% | 80.00 |
| >gi|113473942|ref|NC_008312.1| Trichodesmium erythraeum IMS101 chromosome, complete genome | 4450 | 34.14% | 91.19 |
| >gi|289577265|ref|NC_013921.1| Thermoanaerobacter italicus Ab9 chromosome, complete genome | 2061 | 34.15% | 89.81 |
| >gi|426400511|ref|NC_019566.1| Candidatus Endolissoclinum patella L2 chromosome, complete genome | 1437 | 34.15% | 95.13 |
| >gi|332795693|ref|NC_015518.1| Acidianus hospitalis W1 chromosome, complete genome | 977 | 34.15% | 86.90 |
| >gi|154147860|ref|NC_009713.1| Campylobacter hominis ATCC BAA-381 plasmid pCH4, complete sequence | 7 | 34.15% | 71.43 |
| >gi|217956691|ref|NC_011655.1| Bacillus cereus AH187 plasmid pAH187_270, complete sequence | 109 | 34.16% | 90.83 |
| >gi|376258298|ref|NC_016792.1| Bacillus cereus NC7401 plasmid pNCcld, complete sequence | 109 | 34.16% | 90.83 |
| >gi|188584627|ref|NC_010715.1| Natranaerobius thermophilus JW/NM-WN-LF plasmid pNTHE01, complete sequence | 10 | 34.16% | 90.00 |
| >gi|161527512|ref|NC_010085.1| Nitrosopumilus maritimus SCM1 chromosome, complete genome | 1080 | 34.17% | 89.63 |
| >gi|58584261|ref|NC_006833.1| Wolbachia endosymbiont strain TRS of Brugia malayi, complete genome | 530 | 34.18% | 87.92 |
| >gi|455743321|ref|NC_020420.1| Uncultured Termite group 1 bacterium phylotype Rs-D17 plasmid pTGRD1 DNA, complete sequence | 5 | 34.18% | 80.00 |
| >gi|189485765|ref|NS_000192.1| Uncultured Termite group 1 bacterium phylotype Rs-D17 plasmid pTGRD1 DNA, complete sequence | 5 | 34.18% | 80.00 |
| >gi|407461552|ref|NC_018655.1| Candidatus Nitrosopumilus koreensis AR1 chromosome, complete genome | 1040 | 34.18% | 90.38 |
| >gi|190570478|ref|NC_010981.1| Wolbachia endosymbiont of Culex quinquefasciatus Pel chromosome, complete genome | 658 | 34.19% | 87.99 |
| >gi|408671659|ref|NC_018749.1| Emticicia oligotrophica DSM 17448 plasmid pEMTOL02, complete sequence | 87 | 34.21% | 94.25 |
| >gi|385818454|ref|NC_017471.1| Lactobacillus amylovorus GRL1118 plasmid1, complete sequence | 2 | 34.22% | 100.00 |
| >gi|295987464|ref|NC_014131.1| Leuconostoc kimchii IMSNU 11154 plasmid LkipL4701, complete sequence | 12 | 34.25% | 100.00 |
| >gi|310779695|ref|NC_014633.1| Ilyobacter polytropus DSM 2926 plasmid pILYOP01, complete sequence | 1164 | 34.25% | 87.03 |
| >gi|402558249|ref|NC_018487.1| Bacillus thuringiensis HD-771 plasmid p03, complete sequence | 33 | 34.32% | 90.91 |
| >gi|297543498|ref|NC_014209.1| Thermoanaerobacter mathranii subsp. mathranii str. A3 chromosome, complete genome | 1853 | 34.32% | 89.96 |
| >gi|326789139|ref|NC_015275.1| Clostridium lentocellum DSM 5427 chromosome, complete genome | 2149 | 34.32% | 89.39 |
| >gi|54307232|ref|NC_006376.1| Lactobacillus plantarum WCFS1 plasmid pWCFS102, complete sequence | 4 | 34.33% | 100.00 |
| >gi|345016447|ref|NC_015958.1| Thermoanaerobacter wiegelii Rt8.B1 chromosome, complete genome | 2231 | 34.34% | 89.15 |
| >gi|523529002|ref|NC_021730.1| Acinetobacter baumannii BJAB0868 plasmid p1BJAB0868, complete sequence | 5 | 34.34% | 80.00 |
| >gi|225685337|ref|NC_012439.1| Persephonella marina EX-H1 plasmid unnamed, complete sequence | 36 | 34.35% | 88.89 |
| >gi|426202335|ref|NT_187139.1| Borrelia hermsii DAH clone fragment 1046T | 6 | 34.37% | 83.33 |
| >gi|116326529|ref|NC_008503.1| Lactococcus lactis subsp. cremoris SK11 plasmid 1, complete sequence | 28 | 34.37% | 75.00 |
| >gi|385239402|ref|NC_017165.1| Acinetobacter baumannii TCDC-AB0715 plasmid p1ABTCDC0715, complete sequence | 5 | 34.37% | 80.00 |
| >gi|213155358|ref|NC_011585.1| Acinetobacter baumannii AB0057 plasmid pAB0057, complete sequence | 5 | 34.38% | 80.00 |
| >gi|389870171|ref|NC_017962.1| Enterococcus faecium DO plasmid 2, complete sequence | 69 | 34.38% | 85.51 |
| >gi|169786864|ref|NC_010398.1| Acinetobacter baumannii SDF plasmid p3ABSDF, complete sequence | 20 | 34.40% | 95.00 |
| >gi|169302980|ref|NC_010402.1| Acinetobacter baumannii AYE plasmid p2ABAYE, complete sequence | 6 | 34.40% | 83.33 |
| >gi|472279773|ref|NC_020828.1| Lactobacillus brevis KB290 plasmid pKB290-8 DNA, complete genome | 5 | 34.41% | 80.00 |
| >gi|29377803|ref|NC_004669.1| Enterococcus faecalis V583 plasmid pTEF1, complete sequence | 57 | 34.41% | 82.46 |
| >gi|78776201|ref|NC_007575.1| Sulfurimonas denitrificans DSM 1251 chromosome, complete genome | 1609 | 34.46% | 89.31 |
| >gi|560151351|ref|NC_022909.1| Lactobacillus johnsonii N6.2, complete genome | 1119 | 34.48% | 89.99 |
| >gi|268318562|ref|NC_013504.1| Lactobacillus johnsonii FI9785 chromosome, complete genome | 1056 | 34.49% | 89.39 |
| >gi|386748711|ref|NC_017738.1| Helicobacter cetorum MIT 00-7128 plasmid pHCW, complete sequence | 16 | 34.50% | 93.75 |
| >gi|513844557|ref|NC_021520.1| Lactobacillus plantarum 16 plasmid Lp16L, complete sequence | 3 | 34.50% | 66.67 |
| >gi|294672655|ref|NC_010009.2| Bacillus megaterium QM B1551 plasmid pBM200, complete sequence | 12 | 34.50% | 83.33 |
| >gi|320114857|ref|NC_014964.1| Thermoanaerobacter brockii subsp. finnii Ako-1 chromosome, complete genome | 1889 | 34.50% | 89.57 |
| >gi|167036431|ref|NC_010321.1| Thermoanaerobacter pseudethanolicus ATCC 33223 chromosome, complete genome | 1901 | 34.51% | 89.48 |
| >gi|169302972|ref|NC_010401.1| Acinetobacter baumannii AYE plasmid p1ABAYE, complete sequence | 5 | 34.51% | 80.00 |
| >gi|167038675|ref|NC_010320.1| Thermoanaerobacter sp. X514 chromosome, complete genome | 1981 | 34.52% | 89.25 |
| >gi|307723218|ref|NC_014538.1| Thermoanaerobacter sp. X513 chromosome, complete genome | 1994 | 34.52% | 89.12 |
| >gi|408489461|ref|NC_018721.1| Psychroflexus torquis ATCC 700755 chromosome, complete genome | 2637 | 34.53% | 90.71 |
| >gi|508605261|ref|NC_019425.2| Carnobacterium maltaromaticum LMA28 complete genome | 2525 | 34.53% | 87.92 |
| >gi|310777805|ref|NC_014632.1| Ilyobacter polytropus DSM 2926 chromosome, complete genome | 2306 | 34.53% | 87.51 |
| >gi|386748726|ref|NC_017737.1| Helicobacter cetorum MIT 00-7128 chromosome, complete genome | 2192 | 34.53% | 88.87 |
| >gi|385831931|ref|NC_017487.1| Lactococcus lactis subsp. lactis CV56 plasmid pCV56B, complete sequence | 37 | 34.54% | 75.68 |
| >gi|169302963|ref|NC_010395.1| Acinetobacter baumannii SDF plasmid p1ABSDF, complete sequence | 3 | 34.54% | 100.00 |
| >gi|109948253|ref|NC_008230.1| Helicobacter acinonychis str. Sheeba plasmid pHac1, complete sequence | 2 | 34.55% | 100.00 |
| >gi|529230034|ref|NC_021988.1| Enterococcus faecium Aus0085 plasmid p3, complete sequence | 27 | 34.60% | 81.48 |
| >gi|554650830|ref|NC_022607.1| Carnobacterium sp. WN1359 plasmid pWNCR15, complete sequence | 19 | 34.61% | 89.47 |
| >gi|42518084|ref|NC_005362.1| Lactobacillus johnsonii NCC 533, complete genome | 1201 | 34.61% | 90.42 |
| >gi|554639959|ref|NC_022603.1| Carnobacterium sp. WN1359 plasmid pWNCR64, complete sequence | 35 | 34.62% | 88.57 |
| >gi|224475494|ref|NC_012121.1| Staphylococcus carnosus subsp. carnosus TM300 chromosome, complete genome | 985 | 34.63% | 88.93 |
| >gi|449086465|ref|NC_020249.1| Bacillus thuringiensis serovar kurstaki str. HD73 plasmid pHT73, complete sequence | 38 | 34.66% | 86.84 |
| >gi|374858101|ref|NC_016793.1| Bacillus cereus NC7401 plasmid pNC3, complete sequence | 10 | 34.66% | 70.00 |
| >gi|238651159|ref|NC_012732.1| Rickettsia peacockii str. Rustic plasmid pRPR, complete sequence | 12 | 34.66% | 91.67 |
| >gi|452202047|ref|NC_020392.1| Bacillus thuringiensis serovar thuringiensis str. IS5056 plasmid pIS56-63, complete sequence | 36 | 34.68% | 88.89 |
| >gi|384519696|ref|NC_017315.1| Enterococcus faecalis 62 plasmid EF62pC, complete sequence | 32 | 34.70% | 90.63 |
| >gi|347533753|ref|NC_015978.1| Lactobacillus sanfranciscensis TMW 1.1304 chromosome, complete genome | 618 | 34.70% | 87.86 |
| >gi|529087732|ref|NC_021181.2| Lactobacillus acidophilus La-14, complete genome | 1166 | 34.71% | 88.51 |
| >gi|159162017|ref|NC_006814.3| Lactobacillus acidophilus NCFM chromosome, complete genome | 1169 | 34.71% | 88.45 |
| >gi|386858954|ref|NC_017810.1| Borrelia crocidurae str. Achema plasmid unnamed, complete sequence | 1 | 34.72% | 100.00 |
| >gi|46445634|ref|NC_005861.1| Candidatus Protochlamydia amoebophila UWE25 chromosome, complete genome | 2588 | 34.72% | 90.22 |
| >gi|407708681|ref|NC_018694.1| Bacillus thuringiensis MC28 plasmid pMC54, complete sequence | 25 | 34.73% | 96.00 |
| >gi|471263591|ref|NC_020814.1| Hydrogenobaculum sp. SN, complete genome | 1544 | 34.75% | 88.28 |
| >gi|452943219|ref|NC_020411.1| Hydrogenobaculum sp. HO, complete genome | 1544 | 34.75% | 88.28 |
| >gi|385824947|ref|NC_017477.1| Lactobacillus johnsonii DPC 6026 chromosome, complete genome | 1066 | 34.78% | 89.77 |
| >gi|197336471|ref|NC_011185.1| Vibrio fischeri MJ11 plasmid pMJ100, complete sequence | 140 | 34.80% | 90.71 |
| >gi|426202377|ref|NT_187144.1| Borrelia hermsii DAH clone fragment 983 | 2 | 34.82% | 100.00 |
| >gi|409187973|ref|NC_018885.1| Bacillus thuringiensis Bt407 plasmid BTB_2p, complete sequence | 0 | 34.82% | 0.00 |
| >gi|116326609|ref|NC_008506.1| Lactococcus lactis subsp. cremoris SK11 plasmid 4, complete sequence | 30 | 34.84% | 83.33 |
| >gi|294672670|ref|NC_010008.2| Bacillus megaterium QM B1551 plasmid pBM100, complete sequence | 9 | 34.84% | 88.89 |
| >gi|379703889|ref|NC_016827.1| Leuconostoc mesenteroides subsp. mesenteroides J18 plasmid pKLE01, complete sequence | 20 | 34.85% | 90.00 |
| >gi|195952380|ref|NC_011126.1| Hydrogenobaculum sp. Y04AAS1 chromosome, complete genome | 1508 | 34.85% | 88.53 |
| >gi|381280264|ref|NC_017017.1| Pediococcus claussenii ATCC BAA-344 plasmid pPECL-6, complete sequence | 10 | 34.85% | 100.00 |
| >gi|452202137|ref|NC_020381.1| Bacillus thuringiensis serovar thuringiensis str. IS5056 plasmid pIS56-39, complete sequence | 17 | 34.85% | 100.00 |
| >gi|385836852|ref|NC_017496.1| Lactococcus lactis subsp. cremoris A76 plasmid pQA554, complete sequence | 38 | 34.86% | 86.84 |
| >gi|529229871|ref|NC_021987.1| Enterococcus faecium Aus0085 plasmid p1, complete sequence | 88 | 34.87% | 92.05 |
| >gi|208435470|ref|NC_011334.1| Helicobacter pylori G27 plasmid pHPG27, complete sequence | 10 | 34.87% | 90.00 |
| >gi|169786833|ref|NC_010396.1| Acinetobacter baumannii SDF plasmid p2ABSDF, complete sequence | 29 | 34.89% | 89.66 |
| >gi|217322982|ref|NC_011657.1| Bacillus cereus AH187 plasmid pAH187_3, complete sequence | 8 | 34.91% | 87.50 |
| >gi|402558238|ref|NC_018494.1| Bacillus cereus FRI-35 plasmid p04, complete sequence | 7 | 34.91% | 85.71 |
| >gi|374717315|ref|NC_016774.1| Bacillus cereus NC7401 plasmid pNC4, complete sequence | 8 | 34.91% | 87.50 |
| >gi|281490498|ref|NC_013656.1| Lactococcus lactis subsp. lactis KF147 chromosome, complete genome | 2007 | 34.91% | 89.59 |
| >gi|530341448|ref|NC_022051.1| Listeria monocytogenes strain J1926 plasmid, complete sequence | 38 | 34.92% | 97.37 |
| >gi|530314038|ref|NC_022046.1| Listeria monocytogenes strain J1776 plasmid, complete sequence | 38 | 34.92% | 97.37 |
| >gi|530314110|ref|NC_022047.1| Listeria monocytogenes strain J1817 plasmid, complete sequence | 38 | 34.92% | 97.37 |
| >gi|407719167|ref|NC_018699.1| Leuconostoc carnosum JB16 plasmid pKLC4, complete sequence | 10 | 34.93% | 90.00 |
| >gi|218848179|ref|NC_011776.1| Bacillus cereus AH820 plasmid pAH820_3, complete sequence | 8 | 34.94% | 87.50 |
| >gi|325294206|ref|NC_015185.1| Desulfurobacterium thermolithotrophum DSM 11699 chromosome, complete genome | 2116 | 34.95% | 84.17 |
| >gi|428768415|ref|NC_019776.1| Cyanobacterium aponinum PCC 10605 chromosome, complete genome | 2767 | 34.96% | 84.06 |
| >gi|379704341|ref|NC_016837.1| Streptococcus infantarius subsp. infantarius CJ18 plasmid pSICJ18-1, complete sequence | 11 | 34.96% | 100.00 |
| >gi|384183548|ref|NC_017204.1| Bacillus thuringiensis serovar chinensis CT-43 plasmid pCT51, complete sequence | 19 | 34.97% | 94.74 |
| >gi|52421262|ref|NC_006298.1| Haemophilus somnus 129PT plasmid pHS129, complete sequence | 4 | 34.97% | 75.00 |
| >gi|333895862|ref|NC_015555.1| Thermoanaerobacterium xylanolyticum LX-11 chromosome, complete genome | 1373 | 34.98% | 90.09 |
| >gi|124024712|ref|NC_008819.1| Prochlorococcus marinus str. NATL1A, complete genome | 1259 | 34.98% | 91.02 |
| >gi|45655585|ref|NC_005824.1| Leptospira interrogans serovar Copenhageni str. Fiocruz L1-130 chromosome II, complete sequence | 393 | 34.98% | 91.09 |
| >gi|154248705|ref|NC_009718.1| Fervidobacterium nodosum Rt17-B1 chromosome, complete genome | 1128 | 34.99% | 87.77 |
| >gi|384519623|ref|NC_017313.1| Enterococcus faecalis 62 plasmid EF62pB, complete sequence | 54 | 35.00% | 79.63 |
| >gi|238618479|ref|NC_012726.1| Sulfolobus islandicus M.16.4 chromosome, complete genome | 850 | 35.00% | 89.06 |
| >gi|383484660|ref|NC_017045.1| Riemerella anatipestifer ATCC 11845 = DSM 15868 chromosome, complete genome | 1669 | 35.01% | 90.65 |
| >gi|386320386|ref|NC_017569.1| Riemerella anatipestifer RA-GD chromosome, complete genome | 1696 | 35.01% | 90.80 |
| >gi|229583573|ref|NC_012632.1| Sulfolobus islandicus M.16.27 chromosome, complete genome | 876 | 35.01% | 89.27 |
| >gi|313205511|ref|NC_014738.1| Riemerella anatipestifer ATCC 11845 = DSM 15868 chromosome, complete genome | 1666 | 35.01% | 90.70 |
| >gi|294827553|ref|NC_004342.2| Leptospira interrogans serovar Lai str. 56601 chromosome I, complete sequence | 5864 | 35.02% | 89.44 |
| >gi|558687884|ref|NC_022882.1| Bacillus thuringiensis YBT-1518 plasmid pBMB0233, complete sequence | 162 | 35.02% | 91.36 |
| >gi|212550259|ref|NC_011561.1| Candidatus Azobacteroides pseudotrichonymphae genomovar. CFP2 plasmid pCFPG2, complete sequence | 19 | 35.02% | 78.95 |
| >gi|386072488|ref|NC_017551.1| Leptospira interrogans serovar Lai str. IPAV chromosome chromosome 1, complete sequence | 5867 | 35.02% | 89.47 |
| >gi|532438800|ref|NC_022126.1| Staphylococcus aureus subsp. aureus 55/2053 plasmid, complete sequence | 13 | 35.02% | 92.31 |
| >gi|163659859|ref|NC_010160.1| Bartonella tribocorum CIP 105476 plasmid pBT01, complete sequence | 28 | 35.03% | 85.71 |
| >gi|442313696|ref|NC_020125.1| Riemerella anatipestifer RA-CH-2, complete genome | 1702 | 35.04% | 90.54 |
| >gi|373248555|ref|NC_016750.1| Streptococcus macedonicus ACA-DC 198 plasmid pSMA198, complete sequence | 9 | 35.04% | 77.78 |
| >gi|189501470|ref|NC_010830.1| Candidatus Amoebophilus asiaticus 5a2 chromosome, complete genome | 727 | 35.05% | 91.33 |
| >gi|45655914|ref|NC_005823.1| Leptospira interrogans serovar Copenhageni str. Fiocruz L1-130 chromosome I, complete sequence | 5759 | 35.05% | 89.46 |
| >gi|210135770|ref|NC_011499.1| Helicobacter pylori P12 plasmid HPP12, complete sequence | 7 | 35.05% | 100.00 |
| >gi|325955697|ref|NC_015213.1| Lactobacillus acidophilus 30SC plasmid pRKC30SC1, complete sequence | 10 | 35.07% | 80.00 |
| >gi|407450786|ref|NC_018609.1| Riemerella anatipestifer RA-CH-1 chromosome, complete genome | 1889 | 35.07% | 90.05 |
| >gi|116326684|ref|NC_008501.1| Streptococcus thermophilus LMD-9 plasmid 2, complete sequence | 1 | 35.08% | 100.00 |
| >gi|221316922|ref|NC_011971.1| Bacillus cereus Q1 plasmid pBc53, complete sequence | 14 | 35.08% | 92.86 |
| >gi|482891467|ref|NC_021089.1| Wolbachia endosymbiont of Drosophila simulans wHa, complete genome | 577 | 35.09% | 88.91 |
| >gi|312621127|ref|NC_014720.1| Caldicellulosiruptor kronotskyensis 2002 chromosome, complete genome | 2240 | 35.10% | 87.59 |
| >gi|227826411|ref|NC_012588.1| Sulfolobus islandicus M.14.25 chromosome, complete genome | 827 | 35.10% | 89.60 |
| >gi|459284225|ref|NC_020450.1| Lactococcus lactis subsp. lactis IO-1 DNA, complete genome | 1897 | 35.10% | 88.61 |
| >gi|227829020|ref|NC_012589.1| Sulfolobus islandicus L.S.2.15 chromosome, complete genome | 910 | 35.11% | 90.00 |
| >gi|162958048|ref|NC_007335.2| Prochlorococcus marinus str. NATL2A chromosome, complete genome | 1169 | 35.12% | 91.70 |
| >gi|385836926|ref|NC_017493.1| Lactococcus lactis subsp. cremoris A76 plasmid pQA549, complete sequence | 34 | 35.14% | 82.35 |
| >gi|479324431|ref|NC_021058.1| Sulfolobus islandicus LAL14/1, complete genome | 798 | 35.14% | 90.23 |
| >gi|294653513|ref|NC_004343.2| Leptospira interrogans serovar Lai str. 56601 chromosome II, complete sequence | 395 | 35.14% | 90.63 |
| >gi|386076037|ref|NC_017552.1| Leptospira interrogans serovar Lai str. IPAV chromosome chromosome 2, complete sequence | 395 | 35.14% | 90.63 |
| >gi|385772020|ref|NC_017275.1| Sulfolobus islandicus HVE10/4 chromosome, complete genome | 866 | 35.15% | 89.61 |
| >gi|379703843|ref|NC_016820.1| Leuconostoc mesenteroides subsp. mesenteroides J18 plasmid pKLE02, complete sequence | 22 | 35.15% | 81.82 |
| >gi|379987604|ref|NC_016828.1| Leuconostoc mesenteroides subsp. mesenteroides J18 plasmid pKLE04, complete sequence | 4 | 35.16% | 100.00 |
| >gi|222143330|ref|NC_012001.1| Macrococcus caseolyticus JCSC5402 plasmid pMCCL6, complete sequence | 1 | 35.16% | 100.00 |
| >gi|225629872|ref|NC_012416.1| Wolbachia sp. wRi, complete genome | 584 | 35.16% | 87.16 |
| >gi|402558824|ref|NC_018500.1| Bacillus thuringiensis HD-771 chromosome, complete genome | 3221 | 35.16% | 90.00 |
| >gi|222528057|ref|NC_012034.1| Caldicellulosiruptor bescii DSM 6725 chromosome, complete genome | 2315 | 35.17% | 89.16 |
| >gi|126640109|ref|NC_009084.1| Acinetobacter baumannii ATCC 17978 plasmid pAB2, complete sequence | 11 | 35.18% | 81.82 |
| >gi|529235635|ref|NC_021995.1| Enterococcus faecium Aus0085 plasmid p2, complete sequence | 57 | 35.19% | 85.96 |
| >gi|455743294|ref|NC_020419.1| Uncultured Termite group 1 bacterium phylotype Rs-D17 DNA, complete genome | 1007 | 35.21% | 88.48 |
| >gi|189485003|ref|NS_000191.1| Uncultured Termite group 1 bacterium phylotype Rs-D17, complete genome | 1007 | 35.21% | 88.48 |
| >gi|397698507|ref|NC_018223.1| Enterococcus faecalis D32 plasmid EFD32pB, complete sequence | 63 | 35.21% | 84.13 |
| >gi|402558809|ref|NC_018489.1| Bacillus thuringiensis HD-771 plasmid p06, complete sequence | 8 | 35.23% | 100.00 |
| >gi|42519920|ref|NC_002978.6| Wolbachia endosymbiont of Drosophila melanogaster, complete genome | 546 | 35.23% | 88.64 |
| >gi|307719921|ref|NC_014506.1| Sulfurimonas autotrophica DSM 16294 chromosome, complete genome | 1545 | 35.24% | 89.51 |
| >gi|385829589|ref|NC_017486.1| Lactococcus lactis subsp. lactis CV56 chromosome, complete genome | 1812 | 35.24% | 89.40 |
| >gi|315121750|ref|NC_014774.1| Candidatus Liberibacter solanacearum CLso-ZC1 chromosome, complete genome | 1282 | 35.24% | 88.22 |
| >gi|302870731|ref|NC_014392.1| Caldicellulosiruptor obsidiansis OB47 chromosome, complete genome | 2043 | 35.24% | 87.91 |
| >gi|292493920|ref|NC_013962.1| Candidatus Riesia pediculicola USDA plasmid pPAN, complete sequence | 47 | 35.25% | 72.34 |
| >gi|328956382|ref|NC_015391.1| Carnobacterium sp. 17-4 chromosome, complete genome | 1599 | 35.25% | 89.31 |
| >gi|284996407|ref|NC_013769.1| Sulfolobus islandicus L.D.8.5 chromosome, complete genome | 874 | 35.25% | 90.39 |
| >gi|146295085|ref|NC_009437.1| Caldicellulosiruptor saccharolyticus DSM 8903 chromosome, complete genome | 2211 | 35.25% | 88.78 |
| >gi|455743332|ref|NC_020421.1| Uncultured Termite group 1 bacterium phylotype Rs-D17 plasmid pTGRD2 DNA, complete sequence | 4 | 35.26% | 100.00 |
| >gi|189485775|ref|NS_000193.1| Uncultured Termite group 1 bacterium phylotype Rs-D17 plasmid pTGRD2 DNA, complete sequence | 4 | 35.26% | 100.00 |
| >gi|116628683|ref|NC_008530.1| Lactobacillus gasseri ATCC 33323 chromosome, complete genome | 989 | 35.26% | 89.48 |
| >gi|434379791|ref|NC_018511.1| Bacillus thuringiensis HD-789 plasmid p05, complete sequence | 11 | 35.26% | 72.73 |
| >gi|554648462|ref|NC_022606.1| Carnobacterium sp. WN1359, complete genome | 1537 | 35.26% | 89.20 |
| >gi|434373506|ref|NC_018508.1| Bacillus thuringiensis HD-789, complete genome | 2983 | 35.26% | 90.21 |
| >gi|218895141|ref|NC_011772.1| Bacillus cereus G9842 chromosome, complete genome | 2973 | 35.26% | 89.91 |
| >gi|558688223|ref|NC_022884.1| Enterococcus mundtii QU 25 plasmid pQY024 DNA, complete genome | 20 | 35.27% | 85.00 |
| >gi|449086670|ref|NC_020238.1| Bacillus thuringiensis serovar kurstaki str. HD73, complete genome | 3072 | 35.28% | 90.53 |
| >gi|30018278|ref|NC_004722.1| Bacillus cereus ATCC 14579 chromosome, complete genome | 3007 | 35.28% | 89.72 |
| >gi|390933132|ref|NC_017992.1| Thermoanaerobacterium saccharolyticum JW/SL-YS485 chromosome, complete genome | 1486 | 35.29% | 90.58 |
| >gi|296500838|ref|NC_014171.1| Bacillus thuringiensis BMB171 chromosome, complete genome | 3007 | 35.29% | 90.02 |
| >gi|407719082|ref|NC_018675.1| Leuconostoc carnosum JB16 plasmid pKLC3, complete sequence | 18 | 35.29% | 100.00 |
| >gi|229580722|ref|NC_012623.1| Sulfolobus islandicus Y.N.15.51 chromosome, complete genome | 886 | 35.29% | 90.97 |
| >gi|218230750|ref|NC_011725.1| Bacillus cereus B4264 chromosome, complete genome | 2978 | 35.30% | 89.76 |
| >gi|385774741|ref|NC_017276.1| Sulfolobus islandicus REY15A chromosome, complete genome | 799 | 35.31% | 89.74 |
| >gi|443241923|ref|NC_020156.1| Nonlabens dokdonensis DSW-6, complete genome | 2118 | 35.32% | 89.57 |
| >gi|15671982|ref|NC_002662.1| Lactococcus lactis subsp. lactis Il1403 chromosome, complete genome | 1736 | 35.33% | 89.23 |
| >gi|374317610|ref|NC_016638.1| Mycoplasma haemocanis str. Illinois chromosome, complete genome | 628 | 35.33% | 88.38 |
| >gi|512697424|ref|NC_021507.1| Streptococcus agalactiae ILRI112 complete genome | 1123 | 35.34% | 88.60 |
| >gi|160878162|ref|NC_010001.1| Clostridium phytofermentans ISDg chromosome, complete genome | 2067 | 35.35% | 89.79 |
| >gi|52140164|ref|NC_006274.1| Bacillus cereus E33L chromosome, complete genome | 2922 | 35.35% | 90.11 |
| >gi|431805346|ref|NC_019907.1| Liberibacter crescens BT-1 chromosome, complete genome | 1498 | 35.36% | 89.79 |
| >gi|554463091|ref|NC_022593.1| Lactococcus lactis subsp. lactis KLDS 4.0325, complete genome | 1927 | 35.36% | 89.52 |
| >gi|386733873|ref|NC_017729.1| Bacillus anthracis str. H9401 chromosome, complete genome | 2844 | 35.37% | 89.73 |
| >gi|163937941|ref|NC_010183.1| Bacillus weihenstephanensis KBAB4 plasmid pBWB404, complete sequence | 20 | 35.37% | 90.00 |
| >gi|376264031|ref|NC_016779.1| Bacillus cereus F837/76 chromosome, complete genome | 2947 | 35.37% | 88.80 |
| >gi|383787661|ref|NC_017096.1| Caldisericum exile AZM16c01, complete genome | 1212 | 35.37% | 85.97 |
| >gi|384184088|ref|NC_017208.1| Bacillus thuringiensis serovar chinensis CT-43 chromosome, complete genome | 2940 | 35.38% | 90.24 |
| >gi|30260195|ref|NC_003997.3| Bacillus anthracis str. Ames chromosome, complete genome | 2839 | 35.38% | 89.68 |
| >gi|229599883|ref|NC_012659.1| Bacillus anthracis str. A0248, complete genome | 2839 | 35.38% | 89.68 |
| >gi|50196905|ref|NC_007530.2| Bacillus anthracis str. 'Ames Ancestor' chromosome, complete genome | 2839 | 35.38% | 89.68 |
| >gi|49183039|ref|NC_005945.1| Bacillus anthracis str. Sterne chromosome, complete genome | 2839 | 35.38% | 89.68 |
| >gi|301051741|ref|NC_014335.1| Bacillus cereus biovar anthracis str. CI chromosome, complete genome | 2818 | 35.38% | 89.82 |
| >gi|227812678|ref|NC_012581.1| Bacillus anthracis str. CDC 684 chromosome, complete genome | 2841 | 35.38% | 89.58 |
| >gi|452196381|ref|NC_020376.1| Bacillus thuringiensis serovar thuringiensis str. IS5056, complete genome | 2947 | 35.38% | 90.19 |
| >gi|229577818|ref|NC_012622.1| Sulfolobus islandicus Y.G.57.14 chromosome, complete genome | 853 | 35.39% | 90.39 |
| >gi|383315282|ref|NC_017032.1| Enterococcus faecium Aus0004 plasmid AUS0004_p1, complete sequence | 52 | 35.39% | 82.69 |
| >gi|479179495|ref|NC_021023.1| Enterococcus sp. 7L76 draft genome | 2001 | 35.39% | 88.46 |
| >gi|116326493|ref|NC_008496.1| Leuconostoc mesenteroides subsp. mesenteroides ATCC 8293 plasmid pLEUM1, complete sequence | 18 | 35.39% | 88.89 |
| >gi|218901206|ref|NC_011773.1| Bacillus cereus AH820 chromosome, complete genome | 2871 | 35.40% | 89.62 |
| >gi|49476684|ref|NC_005957.1| Bacillus thuringiensis serovar konkukian str. 97-27 chromosome, complete genome | 2889 | 35.41% | 89.96 |
| >gi|409187965|ref|NC_018877.1| Bacillus thuringiensis Bt407 chromosome, complete genome | 2937 | 35.41% | 90.19 |
| >gi|116326547|ref|NC_008505.1| Lactococcus lactis subsp. cremoris SK11 plasmid 3, complete sequence | 68 | 35.41% | 88.24 |
| >gi|407703236|ref|NC_018693.1| Bacillus thuringiensis MC28 chromosome, complete genome | 2931 | 35.41% | 89.59 |
| >gi|435849688|ref|NC_019972.1| Methanomethylovorans hollandica DSM 15978 plasmid pMETHO01, complete sequence | 133 | 35.42% | 92.48 |
| >gi|558678294|ref|NC_022873.1| Bacillus thuringiensis YBT-1518, complete genome | 3321 | 35.42% | 91.30 |
| >gi|118475778|ref|NC_008600.1| Bacillus thuringiensis str. Al Hakam chromosome, complete genome | 2937 | 35.43% | 88.90 |
| >gi|512543719|ref|NC_021486.1| Streptococcus agalactiae ILRI005 complete genome | 1148 | 35.43% | 88.41 |
| >gi|312134082|ref|NC_014657.1| Caldicellulosiruptor owensensis OL chromosome, complete genome | 1868 | 35.44% | 88.22 |
| >gi|225862057|ref|NC_012472.1| Bacillus cereus 03BB102, complete genome | 2929 | 35.44% | 89.62 |
| >gi|374307319|ref|NC_016630.1| Filifactor alocis ATCC 35896 chromosome, complete genome | 1652 | 35.45% | 89.29 |
| >gi|288557107|ref|NC_013793.1| Bacillus pseudofirmus OF4 plasmid pBpOF4-02, complete sequence | 62 | 35.45% | 90.32 |
| >gi|217322921|ref|NC_011656.1| Bacillus cereus AH187 plasmid pAH187_45, complete sequence | 40 | 35.46% | 87.50 |
| >gi|494702401|ref|NC_021195.1| Streptococcus agalactiae 2-22, complete genome | 945 | 35.47% | 89.10 |
| >gi|385818461|ref|NC_017472.1| Lactobacillus amylovorus GRL1118 plasmid2, complete sequence | 42 | 35.47% | 92.86 |
| >gi|409188585|ref|NC_018888.1| Listeria monocytogenes serotype 7 str. SLCC2482 plasmid pLM7UG1, complete sequence | 31 | 35.48% | 96.77 |
| >gi|409188586|ref|NC_018889.1| Listeria monocytogenes SLCC2372 plasmid pLM1-2cUG1, complete sequence | 31 | 35.48% | 96.77 |
| >gi|339633914|ref|NC_015759.1| Weissella koreensis KACC 15510 chromosome, complete genome | 544 | 35.48% | 92.46 |
| >gi|407719128|ref|NC_018698.1| Leuconostoc carnosum JB16 plasmid pKLC2, complete sequence | 14 | 35.50% | 78.57 |
| >gi|294672678|ref|NC_010010.2| Bacillus megaterium QM B1551 plasmid pBM300, complete sequence | 10 | 35.50% | 90.00 |
| >gi|296100303|ref|NC_014134.1| Leuconostoc kimchii IMSNU 11154 plasmid LkipL4726, complete sequence | 14 | 35.51% | 78.57 |
| >gi|313674129|ref|NC_014759.1| Marivirga tractuosa DSM 4126 chromosome, complete genome | 2243 | 35.51% | 87.47 |
| >gi|18450286|ref|NC_003383.1| Listeria innocua Clip11262 plasmid pLI100, complete sequence | 49 | 35.52% | 87.76 |
| >gi|479134201|ref|NC_021007.1| Candidatus Mycoplasma haemominutum 'Birmingham 1' noncontiguous finished genome | 363 | 35.52% | 88.43 |
| >gi|384177910|ref|NC_017200.1| Bacillus thuringiensis serovar finitimus YBT-020 chromosome, complete genome | 2974 | 35.54% | 89.68 |
| >gi|512538239|ref|NC_021485.1| Streptococcus agalactiae 09mas018883 complete genome | 1122 | 35.55% | 88.86 |
| >gi|333904249|ref|NC_015558.1| Streptococcus parauberis KCTC 11537 chromosome, complete genome | 1122 | 35.55% | 90.82 |
| >gi|296100146|ref|NC_014132.1| Leuconostoc kimchii IMSNU 11154 plasmid LkipL4704, complete sequence | 8 | 35.55% | 100.00 |
| >gi|427740454|ref|NC_019686.1| Rivularia sp. PCC 7116 plasmid pRIV7116.02, complete sequence | 1 | 35.55% | 100.00 |
| >gi|410593712|ref|NC_019048.1| Streptococcus agalactiae SA20-06 chromosome, complete genome | 924 | 35.56% | 88.64 |
| >gi|386747036|ref|NC_017735.1| Helicobacter cetorum MIT 99-5656 chromosome, complete genome | 1773 | 35.56% | 87.54 |
| >gi|163938013|ref|NC_010184.1| Bacillus weihenstephanensis KBAB4 chromosome, complete genome | 2728 | 35.56% | 89.74 |
| >gi|222093774|ref|NC_011969.1| Bacillus cereus Q1 chromosome, complete genome | 2866 | 35.56% | 89.39 |
| >gi|262225764|ref|NC_013451.1| Staphylococcus aureus subsp. aureus ED98 plasmid pAVY, complete sequence | 1 | 35.58% | 100.00 |
| >gi|42779081|ref|NC_003909.8| Bacillus cereus ATCC 10987, complete genome | 2780 | 35.58% | 89.71 |
| >gi|32470572|ref|NC_005006.1| Staphylococcus epidermidis ATCC 12228 plasmid pSE-12228-03, complete sequence | 6 | 35.58% | 100.00 |
| >gi|217957581|ref|NC_011658.1| Bacillus cereus AH187 chromosome, complete genome | 2846 | 35.59% | 89.39 |
| >gi|375282101|ref|NC_016771.1| Bacillus cereus NC7401, complete genome | 2839 | 35.59% | 89.36 |
| >gi|402552844|ref|NC_018491.1| Bacillus cereus FRI-35 chromosome, complete genome | 2769 | 35.60% | 89.35 |
| >gi|557619566|ref|NC_022781.1| Bacillus toyonensis BCT-7112, complete genome | 2634 | 35.60% | 89.45 |
| >gi|215538592|ref|NC_011316.1| Aliivibrio salmonicida LFI1238 plasmid pVSAL43, complete sequence | 3 | 35.61% | 100.00 |
| >gi|76786714|ref|NC_007432.1| Streptococcus agalactiae A909 chromosome, complete genome | 1127 | 35.62% | 87.67 |
| >gi|25010075|ref|NC_004368.1| Streptococcus agalactiae NEM316, complete genome | 1212 | 35.63% | 88.94 |
| >gi|212550288|ref|NC_011562.1| Candidatus Azobacteroides pseudotrichonymphae genomovar. CFP2 plasmid pCFPG3, complete sequence | 12 | 35.64% | 83.33 |
| >gi|22536185|ref|NC_004116.1| Streptococcus agalactiae 2603V/R chromosome, complete genome | 1137 | 35.65% | 88.30 |
| >gi|406708523|ref|NC_018646.1| Streptococcus agalactiae GD201008-001 chromosome, complete genome | 1080 | 35.65% | 88.24 |
| >gi|409187971|ref|NC_018880.1| Bacillus thuringiensis Bt407 plasmid BTB_15p, complete sequence | 11 | 35.65% | 90.91 |
| >gi|452202826|ref|NC_020379.1| Bacillus thuringiensis serovar thuringiensis str. IS5056 plasmid pIS56-15, complete sequence | 9 | 35.65% | 100.00 |
| >gi|298501383|ref|NC_014249.1| 'Nostoc azollae' 0708 plasmid pAzo01, complete sequence | 36 | 35.68% | 97.22 |
| >gi|23097455|ref|NC_004193.1| Oceanobacillus iheyensis HTE831 chromosome, complete genome | 1345 | 35.68% | 88.92 |
| >gi|305662445|ref|NC_014471.1| Ignisphaera aggregans DSM 17230 chromosome, complete genome | 318 | 35.69% | 89.94 |
| >gi|472279762|ref|NC_020827.1| Lactobacillus brevis KB290 plasmid pKB290-6 DNA, complete genome | 5 | 35.69% | 80.00 |
| >gi|408671769|ref|NC_018748.1| Emticicia oligotrophica DSM 17448 chromosome, complete genome | 3205 | 35.70% | 91.11 |
| >gi|188587525|ref|NC_010724.1| Natranaerobius thermophilus JW/NM-WN-LF plasmid pNTHE02, complete sequence | 3 | 35.70% | 100.00 |
| >gi|10957566|ref|NC_002182.1| Chlamydia muridarum Nigg plasmid pMoPn, complete sequence | 3 | 35.72% | 100.00 |
| >gi|374294493|ref|NC_016627.1| Clostridium clariflavum DSM 19732 chromosome, complete genome | 2603 | 35.72% | 89.59 |
| >gi|408355363|ref|NC_018704.1| Amphibacillus xylanus NBRC 15112, complete genome | 880 | 35.72% | 87.95 |
| >gi|126464913|ref|NC_009033.1| Staphylothermus marinus F1 chromosome, complete genome | 440 | 35.73% | 91.14 |
| >gi|319898193|ref|NC_014932.1| Bartonella clarridgeiae 73, complete genome | 1264 | 35.73% | 89.87 |
| >gi|333986242|ref|NC_015574.1| Methanobacterium sp. SWAN-1 chromosome, complete genome | 1636 | 35.73% | 87.47 |
| >gi|389853198|ref|NC_017949.1| Lactococcus lactis subsp. cremoris NZ9000 chromosome, complete genome | 1832 | 35.74% | 89.74 |
| >gi|544393402|ref|NC_022369.1| Lactococcus lactis subsp. cremoris KW2, complete genome | 1892 | 35.74% | 89.11 |
| >gi|125622882|ref|NC_009004.1| Lactococcus lactis subsp. cremoris MG1363 chromosome, complete genome | 1830 | 35.75% | 89.73 |
| >gi|313671969|ref|NC_014758.1| Calditerrivibrio nitroreducens DSM 19672 chromosome, complete genome | 1658 | 35.75% | 89.57 |
| >gi|472279709|ref|NC_020824.1| Lactobacillus brevis KB290 plasmid pKB290-7 DNA, complete genome | 0 | 35.77% | 0.00 |
| >gi|651866761|ref|NC_022549.1| complete chromosome Acholeplasma brassicae | 733 | 35.77% | 89.22 |
| >gi|385229299|ref|NC_017377.1| Helicobacter pylori Puno120 plasmid pHPPN120, complete sequence | 9 | 35.77% | 100.00 |
| >gi|312281360|ref|NC_014719.1| Caldicellulosiruptor kristjanssonii I77R1B plasmid pCALKR01, complete sequence | 12 | 35.79% | 83.33 |
| >gi|15896971|ref|NC_002754.1| Sulfolobus solfataricus P2 chromosome, complete genome | 1014 | 35.79% | 86.88 |
| >gi|332290580|ref|NC_015461.1| Gallibacterium anatis UMN179 plasmid pUMN179, complete sequence | 11 | 35.80% | 81.82 |
| >gi|226526990|ref|NC_012552.1| Lactobacillus johnsonii FI9785 plasmid p9785S, complete sequence | 4 | 35.81% | 75.00 |
| >gi|384432549|ref|NC_017274.1| Sulfolobus solfataricus 98/2 chromosome, complete genome | 912 | 35.83% | 86.84 |
| >gi|325957759|ref|NC_015216.1| Methanobacterium sp. AL-21 chromosome, complete genome | 1261 | 35.83% | 90.01 |
| >gi|558688140|ref|NC_022883.1| Enterococcus mundtii QU 25 plasmid pQY082 DNA, complete genome | 63 | 35.83% | 79.37 |
| >gi|414073314|ref|NC_019433.1| Lactococcus lactis subsp. cremoris UC509.9 plasmid pCIS3, complete sequence | 10 | 35.85% | 80.00 |
| >gi|116510843|ref|NC_008527.1| Lactococcus lactis subsp. cremoris SK11, complete genome | 1804 | 35.86% | 89.80 |
| >gi|298717635|ref|NC_014257.1| Helicobacter pylori B8 plasmid HPB8p, complete sequence | 2 | 35.88% | 100.00 |
| >gi|152973854|ref|NC_009674.1| Bacillus cytotoxicus NVH 391-98 chromosome, complete genome | 2317 | 35.88% | 88.22 |
| >gi|414073323|ref|NC_019435.1| Lactococcus lactis subsp. cremoris UC509.9, complete genome | 1662 | 35.88% | 90.31 |
| >gi|385836969|ref|NC_017492.1| Lactococcus lactis subsp. cremoris A76 chromosome, complete genome | 1813 | 35.88% | 89.91 |
| >gi|325953647|ref|NC_015144.1| Weeksella virosa DSM 16922 chromosome, complete genome | 1612 | 35.92% | 89.21 |
| >gi|383750634|ref|NC_017064.1| Helicobacter pylori ELS37 plasmid pHPELS37, complete sequence | 6 | 35.92% | 66.67 |
| >gi|513844788|ref|NC_021525.1| Lactobacillus plantarum 16 plasmid Lp16B, complete sequence | 2 | 35.93% | 100.00 |
| >gi|32265499|ref|NC_004917.1| Helicobacter hepaticus ATCC 51449 chromosome, complete genome | 962 | 35.93% | 91.79 |
| >gi|434396591|ref|NC_019748.1| Stanieria cyanosphaera PCC 7437, complete genome | 2381 | 35.95% | 92.06 |
| >gi|48477072|ref|NC_005877.1| Picrophilus torridus DSM 9790 chromosome, complete genome | 699 | 35.97% | 90.41 |
| >gi|389869887|ref|NC_017963.1| Enterococcus faecium DO plasmid 3, complete sequence | 155 | 35.97% | 86.45 |
| >gi|212550319|ref|NC_011564.1| Candidatus Azobacteroides pseudotrichonymphae genomovar. CFP2 plasmid pCFPG1, complete sequence | 26 | 35.97% | 80.77 |
| >gi|393202538|ref|NC_018069.1| Solibacillus silvestris StLB046 plasmid pSSIL1, complete sequence | 15 | 35.98% | 86.67 |
| >gi|447914114|ref|NC_020208.1| Enterococcus faecium NRRL B-2354 plasmid pNB2354_1, complete sequence | 125 | 35.98% | 88.00 |
| >gi|501148317|ref|NC_021228.1| Lactobacillus plantarum subsp. plantarum P-8 plasmid LBPp6, complete sequence | 1 | 35.98% | 100.00 |
| >gi|288561476|ref|NC_013792.1| Bacillus pseudofirmus OF4 plasmid pBpOF4-01, complete sequence | 143 | 35.98% | 85.31 |
| >gi|434379816|ref|NC_018512.1| Bacillus thuringiensis HD-789 plasmid p06, complete sequence | 5 | 35.99% | 80.00 |
| >gi|384044106|ref|NC_017139.1| Bacillus megaterium WSH-002 plasmid WSH-002_p1, complete sequence | 51 | 36.00% | 90.20 |
| >gi|402558574|ref|NC_018502.1| Bacillus thuringiensis HD-771 plasmid p05, complete sequence | 21 | 36.02% | 80.95 |
| >gi|336055419|ref|NC_015603.1| Lactobacillus kefiranofaciens ZW3 plasmid pWW2, complete sequence | 35 | 36.02% | 85.71 |
| >gi|525901154|ref|NC_021828.1| Listeria monocytogenes strain R2-502 plasmid, complete sequence | 36 | 36.03% | 94.44 |
| >gi|307069350|ref|NC_014495.1| Listeria monocytogenes SLCC2755 plasmid pLM1-2bUG1, complete sequence | 33 | 36.04% | 96.97 |
| >gi|352516173|ref|NC_016052.1| Tetragenococcus halophilus NBRC 12172, complete genome | 2030 | 36.04% | 88.47 |
| >gi|257065520|ref|NC_013171.1| Anaerococcus prevotii DSM 20548 chromosome, complete genome | 1005 | 36.07% | 87.16 |
| >gi|312792283|ref|NC_014721.1| Caldicellulosiruptor kristjanssonii I77R1B chromosome, complete genome | 2017 | 36.10% | 88.60 |
| >gi|228288719|ref|NC_012624.1| Sulfolobus islandicus Y.N.15.51 plasmid pYN01, complete sequence | 19 | 36.11% | 78.95 |
| >gi|340620779|ref|NC_015846.1| Capnocytophaga canimorsus Cc5 chromosome, complete genome | 2717 | 36.11% | 88.59 |
| >gi|312126262|ref|NC_014652.1| Caldicellulosiruptor hydrothermalis 108 chromosome, complete genome | 1959 | 36.11% | 88.21 |
| >gi|347533684|ref|NC_015980.1| Lactobacillus sanfranciscensis TMW 1.1304 plasmid pLS2, complete sequence | 19 | 36.12% | 84.21 |
| >gi|344995333|ref|NC_015949.1| Caldicellulosiruptor lactoaceticus 6A chromosome, complete genome | 2039 | 36.13% | 88.33 |
| >gi|284807103|ref|NC_013770.1| Sulfolobus islandicus L.D.8.5 plasmid pLD8501, complete sequence | 14 | 36.14% | 85.71 |
| >gi|118445179|ref|NC_008598.1| Bacillus thuringiensis str. Al Hakam plasmid pALH1, complete sequence | 27 | 36.15% | 85.19 |
| >gi|126640097|ref|NC_009083.1| Acinetobacter baumannii ATCC 17978 plasmid pAB1, complete sequence | 11 | 36.16% | 100.00 |
| >gi|529235714|ref|NC_021996.1| Enterococcus faecium Aus0085 plasmid p5, complete sequence | 7 | 36.17% | 85.71 |
| >gi|412336930|ref|NC_019100.1| Bacteriovorax marinus SJ plasmid pBMS1, complete sequence | 3 | 36.19% | 100.00 |
| >gi|376270191|ref|NC_016794.1| Bacillus cereus F837/76 plasmid pF837_55, complete sequence | 27 | 36.19% | 85.19 |
| >gi|390952789|ref|NC_018013.1| Aequorivita sublithincola DSM 14238 chromosome, complete genome | 2525 | 36.19% | 89.47 |
| >gi|478454922|ref|NC_020956.1| Chlamydia trachomatis L2b/UCH-2 plasmid pL2bUCH2, complete sequence | 6 | 36.20% | 100.00 |
| >gi|172055989|ref|NC_010555.1| Proteus mirabilis plasmid pHI4320, complete sequence | 46 | 36.21% | 95.65 |
| >gi|478452215|ref|NC_020951.1| Chlamydia trachomatis L1/115 plasmid pL1115, complete sequence | 6 | 36.21% | 100.00 |
| >gi|295131884|ref|NC_014041.1| Zunongwangia profunda SM-A87 chromosome, complete genome | 3233 | 36.22% | 89.51 |
| >gi|228298555|ref|NC_012626.1| Chlamydia trachomatis plasmid pSW5, complete sequence | 6 | 36.22% | 100.00 |
| >gi|478462155|ref|NC_020952.1| Chlamydia trachomatis L1/224 plasmid pL1224, complete sequence | 6 | 36.23% | 100.00 |
| >gi|478469360|ref|NC_020983.1| Chlamydia trachomatis L2b/795 plasmid pL2b795, complete sequence | 6 | 36.23% | 100.00 |
| >gi|478470260|ref|NC_020984.1| Chlamydia trachomatis L2b/820007 plasmid pL2b820007 complete sequence | 6 | 36.23% | 100.00 |
| >gi|478467551|ref|NC_020980.1| Chlamydia trachomatis L2b/Ams1 plasmid pAms1, complete sequence | 6 | 36.23% | 100.00 |
| >gi|478450416|ref|NC_020948.1| Chlamydia trachomatis L2b/Ams2 plasmid pAms2, complete sequence | 6 | 36.23% | 100.00 |
| >gi|478464852|ref|NC_020949.1| Chlamydia trachomatis L2b/Ams3 plasmid pAms3, complete sequence | 6 | 36.23% | 100.00 |
| >gi|478468451|ref|NC_020981.1| Chlamydia trachomatis L2b/Ams4 plasmid pAms4, complete sequence | 6 | 36.23% | 100.00 |
| >gi|478451316|ref|NC_020950.1| Chlamydia trachomatis L2b/Ams5 plasmid pAms5, complete sequence | 6 | 36.23% | 100.00 |
| >gi|478463952|ref|NC_020955.1| Chlamydia trachomatis L2b/CV204 plasmid pL2bCV204, complete sequence | 6 | 36.23% | 100.00 |
| >gi|478453120|ref|NC_020953.1| Chlamydia trachomatis L2b/Canada1 plasmid pL2bCan1, complete sequence | 6 | 36.23% | 100.00 |
| >gi|478453129|ref|NC_020954.1| Chlamydia trachomatis L2b/Canada2 plasmid pL2bCan2, complete sequence | 6 | 36.23% | 100.00 |
| >gi|385270623|ref|NC_017438.1| Chlamydia trachomatis A2497 plasmid0001, complete sequence | 5 | 36.23% | 100.00 |
| >gi|478455832|ref|NC_020960.1| Chlamydia trachomatis E/SotonE8 plasmid pSotonE8, complete sequence | 6 | 36.23% | 100.00 |
| >gi|482546019|ref|NC_021049.1| Chlamydia trachomatis L2/434/Bu(f) plasmid pL2/434/Bu(f), complete sequence | 5 | 36.23% | 100.00 |
| >gi|482545077|ref|NC_021051.1| Chlamydia trachomatis L2/434/Bu(i) plasmid pL2/434/Bu(i), complete sequence | 5 | 36.23% | 100.00 |
| >gi|558687663|ref|NC_022879.1| Enterococcus mundtii QU 25 plasmid pQY182 DNA, complete genome | 138 | 36.24% | 88.41 |
| >gi|478472051|ref|NC_020985.1| Chlamydia trachomatis L2b/LST plasmid pL2bLST, complete sequence | 6 | 36.24% | 100.00 |
| >gi|478462146|ref|NC_020947.1| Chlamydia trachomatis E/Bour plasmid pBour, complete sequence | 6 | 36.24% | 100.00 |
| >gi|228404350|ref|NC_012631.1| Chlamydia trachomatis plasmid pSW3, complete sequence | 6 | 36.24% | 100.00 |
| >gi|469820164|ref|NC_020551.1| Chlamydia trachomatis IU824 plasmid pIU824, complete sequence | 6 | 36.24% | 100.00 |
| >gi|469816136|ref|NC_020513.1| Chlamydia trachomatis IU888 plasmid pIU888, complete sequence | 6 | 36.24% | 100.00 |
| >gi|478472955|ref|NC_020986.1| Chlamydia trachomatis D/SotonD1 plasmid pSotonD1, complete sequence | 6 | 36.25% | 100.00 |
| >gi|228292900|ref|NC_012625.1| Chlamydia trachomatis plasmid pSW4, complete sequence | 6 | 36.25% | 100.00 |
| >gi|478474760|ref|NC_020988.1| Chlamydia trachomatis F/SotonF3 plasmid pSotonF3, complete sequence | 6 | 36.25% | 100.00 |
| >gi|568110865|ref|NC_023057.1| Chlamydia trachomatis C/TW-3 plasmid unnamed, complete sequence | 8 | 36.25% | 100.00 |
| >gi|478468460|ref|NC_020982.1| Chlamydia trachomatis L1/1322/p2 plasmid pL11322, complete sequence | 6 | 36.25% | 100.00 |
| >gi|410686362|ref|NC_019272.1| Chlamydia trachomatis A/363 plasmid pA363, complete sequence | 6 | 36.26% | 100.00 |
| >gi|478449516|ref|NC_020946.1| Chlamydia trachomatis A/5291 plasmid pA5291, complete sequence | 6 | 36.26% | 100.00 |
| >gi|478465752|ref|NC_020979.1| Chlamydia trachomatis A/7249 plasmid pA7249, complete sequence | 6 | 36.26% | 100.00 |
| >gi|478473858|ref|NC_020987.1| Chlamydia trachomatis E/SotonE4 plasmid pSotonE4, complete sequence | 6 | 36.26% | 100.00 |
| >gi|158319059|ref|NC_009922.1| Alkaliphilus oremlandii OhILAs chromosome, complete genome | 1493 | 36.26% | 89.15 |
| >gi|76789623|ref|NC_007430.1| Chlamydia trachomatis A/HAR-13 plasmid pCTA, complete sequence | 5 | 36.27% | 100.00 |
| >gi|479158859|ref|NC_021016.1| Butyrate-producing bacterium SSC/2, complete genome | 1361 | 36.28% | 85.01 |
| >gi|568162877|ref|NC_023069.1| Sulfolobus acidocaldarius SUSAZ, complete genome | 731 | 36.30% | 86.59 |
| >gi|478457632|ref|NC_020961.1| Chlamydia trachomatis G/SotonG1 plasmid pSotonG1, complete sequence | 6 | 36.30% | 100.00 |
| >gi|327399851|ref|NC_015322.1| Lactobacillus amylovorus GRL 1112 plasmid2, complete sequence | 23 | 36.30% | 82.61 |
| >gi|478463055|ref|NC_020957.1| Chlamydia trachomatis L3/404/LN plasmid pL3404, complete sequence | 6 | 36.32% | 100.00 |
| >gi|478455823|ref|NC_020958.1| Chlamydia trachomatis D/SotonD5 plasmid pSotonD5, complete sequence | 6 | 36.32% | 100.00 |
| >gi|478460345|ref|NC_020962.1| Chlamydia trachomatis Ia/SotonIa1 plasmid pSotonIa1, complete sequence | 7 | 36.32% | 100.00 |
| >gi|478475664|ref|NC_020989.1| Chlamydia trachomatis Ia/SotonIa3 plasmid pSotonIa3, complete sequence | 7 | 36.32% | 100.00 |
| >gi|222143513|ref|NC_012002.1| Macrococcus caseolyticus JCSC5402 plasmid pMCCL7, complete sequence | 1 | 36.32% | 100.00 |
| >gi|188584642|ref|NC_010718.1| Natranaerobius thermophilus JW/NM-WN-LF chromosome, complete genome | 1194 | 36.32% | 91.62 |
| >gi|385244078|ref|NC_017435.1| Chlamydia trachomatis D-EC plasmid pCTDEC1, complete sequence | 5 | 36.33% | 100.00 |
| >gi|385244958|ref|NC_017433.1| Chlamydia trachomatis D-LC plasmid pCTDLC1, complete sequence | 5 | 36.33% | 100.00 |
| >gi|478459444|ref|NC_020959.1| Chlamydia trachomatis D/SotonD6 plasmid pSotonD6, complete sequence | 6 | 36.33% | 100.00 |
| >gi|478457641|ref|NC_020963.1| Chlamydia trachomatis K/SotonK1 plasmid pSotonK1, complete sequence | 6 | 36.33% | 100.00 |
| >gi|116871422|ref|NC_008555.1| Listeria welshimeri serovar 6b str. SLCC5334 chromosome, complete genome | 1615 | 36.35% | 89.66 |
| >gi|509152352|ref|NC_021354.1| Erysipelothrix rhusiopathiae SY1027, complete genome | 533 | 36.36% | 92.31 |
| >gi|260738835|ref|NC_013358.1| Zymomonas mobilis subsp. mobilis NCIB 11163 plasmid pZA1003, complete sequence | 8 | 36.37% | 87.50 |
| >gi|240851420|ref|NC_012847.1| Bartonella grahamii as4aup plasmid pBGR3, complete sequence | 18 | 36.38% | 88.89 |
| >gi|425790176|ref|NC_019562.1| Helicobacter pylori Aklavik117 plasmid p2HPAKL117, complete sequence | 6 | 36.39% | 100.00 |
| >gi|108564598|ref|NC_008087.1| Helicobacter pylori HPAG1 plasmid pHPAG1, complete sequence | 4 | 36.39% | 75.00 |
| >gi|469823624|ref|NC_020556.1| Helicobacter pylori OK310 plasmid pHPOK310 DNA, complete genome | 10 | 36.41% | 70.00 |
| >gi|427740400|ref|NC_019679.1| Rivularia sp. PCC 7116 plasmid pRIV7116.01, complete sequence | 18 | 36.43% | 100.00 |
| >gi|253750612|ref|NC_012923.1| Streptococcus suis BM407 plasmid pBM407, complete sequence | 19 | 36.43% | 73.68 |
| >gi|10957041|ref|NC_001880.1| Aquifex aeolicus VF5 plasmid ece1, complete sequence | 41 | 36.44% | 75.61 |
| >gi|33239452|ref|NC_005042.1| Prochlorococcus marinus subsp. marinus str. CCMP1375 complete genome | 898 | 36.44% | 88.75 |
| >gi|518651360|ref|NC_021592.1| Ferroplasma acidarmanus fer1, complete genome | 622 | 36.47% | 92.77 |
| >gi|346722692|ref|NC_012985.3| Candidatus Liberibacter asiaticus str. psy62 chromosome, complete genome | 882 | 36.47% | 88.89 |
| >gi|294663009|ref|NC_004604.2| Bacillus megaterium QM B1551 plasmid pBM400, complete sequence | 27 | 36.47% | 88.89 |
| >gi|512656346|ref|NC_021503.1| Lactobacillus reuteri I5007 plasmid pLRI01, complete sequence | 16 | 36.47% | 87.50 |
| >gi|327402011|ref|NC_015321.1| Fluviicola taffensis DSM 16823 chromosome, complete genome | 2093 | 36.49% | 89.20 |
| >gi|386118364|ref|NC_017670.1| Halobacillus halophilus DSM 2266 plasmid PL3, complete sequence | 7 | 36.50% | 71.43 |
| >gi|386745517|ref|NC_017734.1| Helicobacter pylori HUP-B14 plasmid pHPB14, complete sequence | 10 | 36.50% | 70.00 |
| >gi|389870257|ref|NC_017961.1| Enterococcus faecium DO plasmid 1, complete sequence | 23 | 36.51% | 69.57 |
| >gi|521182576|ref|NC_021662.1| Psychrobacter sp. G plasmid PsyG_4, complete sequence | 4 | 36.52% | 75.00 |
| >gi|317126741|ref|NC_014829.1| Bacillus cellulosilyticus DSM 2522 chromosome, complete genome | 2242 | 36.52% | 88.40 |
| >gi|375287510|ref|NC_016772.1| Bacillus cereus NC7401 plasmid pNC1, complete sequence | 12 | 36.54% | 100.00 |
| >gi|558684320|ref|NC_022874.1| Bacillus thuringiensis YBT-1518 plasmid pBMB0229, complete sequence | 31 | 36.54% | 90.32 |
| >gi|339319317|ref|NC_015722.1| Candidatus Midichloria mitochondrii IricVA chromosome, complete genome | 620 | 36.55% | 90.65 |
| >gi|336065242|ref|NC_015601.1| Erysipelothrix rhusiopathiae str. Fujisawa chromosome, complete genome | 523 | 36.56% | 91.78 |
| >gi|470204499|ref|NC_020549.1| Candidatus Liberibacter asiaticus str. gxpsy, complete genome | 892 | 36.57% | 88.90 |
| >gi|325102757|ref|NC_015177.1| Pedobacter saltans DSM 12145 chromosome, complete genome | 2529 | 36.58% | 88.97 |
| >gi|434379279|ref|NC_018509.1| Bacillus thuringiensis HD-789 plasmid p02, complete sequence | 82 | 36.59% | 86.59 |
| >gi|284803266|ref|NC_013767.1| Listeria monocytogenes 08-5578 plasmid pLM5578, complete sequence | 35 | 36.59% | 97.14 |
| >gi|120434372|ref|NC_008571.1| Gramella forsetii KT0803 chromosome, complete genome | 2145 | 36.61% | 89.79 |
| >gi|406024901|ref|NC_018605.1| Cardinium endosymbiont cEper1 of Encarsia pergandiella, complete genome | 586 | 36.62% | 92.32 |
| >gi|408671740|ref|NC_018743.1| Emticicia oligotrophica DSM 17448 plasmid pEMTOL03, complete sequence | 36 | 36.63% | 88.89 |
| >gi|298673978|ref|NC_014253.1| Methanohalobium evestigatum Z-7303 chromosome, complete genome | 834 | 36.63% | 89.33 |
| >gi|302390797|ref|NC_014378.1| Acetohalobium arabaticum DSM 5501 chromosome, complete genome | 688 | 36.63% | 91.28 |
| >gi|222152201|ref|NC_012004.1| Streptococcus uberis 0140J chromosome, complete genome | 1142 | 36.63% | 87.30 |
| >gi|325955704|ref|NC_015218.1| Lactobacillus acidophilus 30SC plasmid pRKC30SC2, complete sequence | 11 | 36.65% | 81.82 |
| >gi|300172302|ref|NC_014319.1| Leuconostoc gasicomitatum LMG 18811, complete genome | 622 | 36.66% | 91.80 |
| >gi|449066047|ref|NC_020246.1| Sulfolobus acidocaldarius N8, complete genome | 696 | 36.67% | 88.94 |
| >gi|406598989|ref|NC_018631.1| Leuconostoc gelidum JB7 chromosome, complete genome | 643 | 36.68% | 92.22 |
| >gi|339635250|ref|NC_015756.1| Weissella koreensis KACC 15510 plasmid WKp2903, complete sequence | 8 | 36.70% | 75.00 |
| >gi|449068323|ref|NC_020247.1| Sulfolobus acidocaldarius Ron12/I, complete genome | 710 | 36.71% | 88.87 |
| >gi|70605853|ref|NC_007181.1| Sulfolobus acidocaldarius DSM 639 chromosome, complete genome | 709 | 36.71% | 88.86 |
| >gi|425791567|ref|NC_019565.1| Helicobacter pylori Aklavik86 plasmid p2HPAKL86, complete sequence | 1 | 36.72% | 100.00 |
| >gi|315630409|ref|NC_014826.1| Ruminococcus albus 7 plasmid pRUMAL03, complete sequence | 9 | 36.73% | 77.78 |
| >gi|508126650|ref|NC_021314.1| Streptococcus iniae SF1, complete genome | 1226 | 36.74% | 86.70 |
| >gi|386262355|ref|NC_017370.1| Helicobacter pylori F32 plasmid pHPF32, complete sequence | 5 | 36.75% | 100.00 |
| >gi|374286830|ref|NC_016620.1| Bacteriovorax marinus SJ, complete genome | 2225 | 36.75% | 85.89 |
| >gi|397912605|ref|NC_018264.1| Thermoanaerobacterium phage THSA-485A, complete genome | 5 | 36.77% | 100.00 |
| >gi|238921767|ref|NC_012780.1| Eubacterium eligens ATCC 27750 plasmid unnamed, complete sequence | 230 | 36.78% | 87.83 |
| >gi|385812838|ref|NC_017467.1| Lactobacillus helveticus H10 chromosome, complete genome | 1159 | 36.79% | 89.21 |
| >gi|385814792|ref|NC_017468.1| Lactobacillus helveticus H10 plasmid pH10, complete sequence | 21 | 36.79% | 100.00 |
| >gi|403514032|ref|NC_018528.1| Lactobacillus helveticus R0052 chromosome, complete genome | 1082 | 36.80% | 89.56 |
| >gi|172034820|ref|NC_010539.1| Cyanothece sp. ATCC 51142 plasmid A, complete sequence | 11 | 36.80% | 90.91 |
| >gi|383315276|ref|NC_017024.1| Enterococcus faecium Aus0004 plasmid AUS0004_p3, complete sequence | 6 | 36.81% | 83.33 |
| >gi|384519687|ref|NC_017314.1| Enterococcus faecalis 62 plasmid EF62pA, complete sequence | 5 | 36.81% | 80.00 |
| >gi|390941666|ref|NC_018010.1| Belliella baltica DSM 15883 chromosome, complete genome | 2653 | 36.81% | 88.20 |
| >gi|377808793|ref|NC_016605.1| Pediococcus claussenii ATCC BAA-344 chromosome, complete genome | 666 | 36.82% | 91.44 |
| >gi|150387853|ref|NC_009633.1| Alkaliphilus metalliredigens QYMF chromosome, complete genome | 1847 | 36.82% | 88.79 |
| >gi|347750429|ref|NC_004350.2| Streptococcus mutans UA159 chromosome, complete genome | 1249 | 36.83% | 88.95 |
| >gi|397648808|ref|NC_018089.1| Streptococcus mutans GS-5 chromosome, complete genome | 1273 | 36.84% | 88.61 |
| >gi|297526028|ref|NC_014205.1| Staphylothermus hellenicus DSM 12710 chromosome, complete genome | 406 | 36.84% | 93.35 |
| >gi|290579526|ref|NC_013928.1| Streptococcus mutans NN2025, complete genome | 1227 | 36.85% | 89.24 |
| >gi|472279727|ref|NC_020826.1| Lactobacillus brevis KB290 plasmid pKB290-3 DNA, complete genome | 13 | 36.86% | 100.00 |
| >gi|338204606|ref|NC_015701.1| Lactobacillus reuteri SD2112 plasmid pLR584, complete sequence | 5 | 36.86% | 100.00 |
| >gi|222150250|ref|NC_011999.1| Macrococcus caseolyticus JCSC5402, complete genome | 596 | 36.88% | 87.25 |
| >gi|295691862|ref|NC_014106.1| Lactobacillus crispatus ST1, complete genome | 1010 | 36.88% | 89.80 |
| >gi|530891924|ref|NC_022097.1| Treponema pedis str. T A4, complete genome | 3777 | 36.90% | 88.19 |
| >gi|170016294|ref|NC_010469.1| Leuconostoc citreum KM20 plasmid pLCK4, complete sequence | 8 | 36.91% | 75.00 |
| >gi|525706521|ref|NC_021744.1| Lactobacillus helveticus CNRZ32, complete genome | 1080 | 36.93% | 89.54 |
| >gi|392987295|ref|NC_018081.1| Enterococcus hirae ATCC 9790 chromosome, complete genome | 1935 | 36.94% | 87.03 |
| >gi|512593096|ref|NC_021495.1| Lactobacillus reuteri I5007 plasmid pLRI03, complete sequence | 3 | 36.94% | 100.00 |
| >gi|172034905|ref|NC_010543.1| Cyanothece sp. ATCC 51142 plasmid D, complete sequence | 16 | 36.97% | 68.75 |
| >gi|305664376|ref|NC_014472.1| Maribacter sp. HTCC2170 chromosome, complete genome | 1726 | 36.98% | 90.61 |
| >gi|554639096|ref|NC_022601.1| Carnobacterium sp. WN1359 plasmid pWNCR12, complete sequence | 10 | 36.99% | 60.00 |
| >gi|116326679|ref|NC_008500.1| Streptococcus thermophilus LMD-9 plasmid 1, complete sequence | 1 | 37.00% | 100.00 |
| >gi|172087787|ref|NC_006841.2| Vibrio fischeri ES114 chromosome II, complete sequence | 394 | 37.02% | 91.88 |
| >gi|530313872|ref|NC_022045.1| Listeria monocytogenes strain N1-011A plasmid, complete sequence | 78 | 37.05% | 92.31 |
| >gi|172059053|ref|NC_010549.1| Exiguobacterium sibiricum 255-15 plasmid pEXIG01, complete sequence | 7 | 37.05% | 85.71 |
| >gi|387785181|ref|NC_017768.1| Streptococcus mutans LJ23, complete genome | 1203 | 37.05% | 88.61 |
| >gi|161506634|ref|NC_010080.1| Lactobacillus helveticus DPC 4571, complete genome | 1050 | 37.08% | 89.62 |
| >gi|296005657|ref|NC_014135.1| Leuconostoc kimchii IMSNU 11154 plasmid LkipL48, complete sequence | 1 | 37.08% | 100.00 |
| >gi|218442611|ref|NC_011738.1| Cyanothece sp. PCC 7424 plasmid pP742401, complete sequence | 214 | 37.09% | 87.85 |
| >gi|289163353|ref|NC_013861.1| Legionella longbeachae NSW150 chromosome, complete genome | 1694 | 37.11% | 92.03 |
| >gi|440685390|ref|NC_019775.1| Anabaena cylindrica PCC 7122 plasmid pANACY.06, complete sequence | 7 | 37.12% | 100.00 |
| >gi|414075268|ref|NC_019436.1| Lactococcus lactis subsp. cremoris UC509.9 plasmid pCIS6, complete sequence | 30 | 37.12% | 86.67 |
| >gi|347547510|ref|NC_016011.1| Listeria ivanovii subsp. ivanovii PAM 55, complete genome | 1423 | 37.13% | 89.60 |
| >gi|568306560|ref|NC_023147.1| Paenibacillus larvae subsp. larvae DSM 25430 plasmid pPLA2_10, complete sequence | 4 | 37.14% | 100.00 |
| >gi|225849564|ref|NC_012440.1| Persephonella marina EX-H1 chromosome, complete genome | 1094 | 37.16% | 86.84 |
| >gi|239828745|ref|NC_012795.1| Desulfovibrio magneticus RS-1 plasmid pDMC2, complete sequence | 4 | 37.17% | 100.00 |
| >gi|113460149|ref|NC_008309.1| Haemophilus somnus 129PT chromosome, complete genome | 968 | 37.20% | 90.50 |
| >gi|392389424|ref|NC_018016.1| Ornithobacterium rhinotracheale DSM 15997 chromosome, complete genome | 2557 | 37.22% | 83.73 |
| >gi|414078960|ref|NC_019428.1| Anabaena sp. 90 plasmid pANA01, complete sequence | 27 | 37.23% | 77.78 |
| >gi|407717513|ref|NC_018673.1| Leuconostoc carnosum JB16 chromosome, complete genome | 415 | 37.24% | 94.22 |
| >gi|197336667|ref|NC_011186.1| Vibrio fischeri MJ11 chromosome II, complete sequence | 390 | 37.25% | 92.05 |
| >gi|414079049|ref|NC_019429.1| Anabaena sp. 90 plasmid pANA02, complete sequence | 17 | 37.25% | 82.35 |
| >gi|376259380|ref|NC_016791.1| Clostridium sp. BNL1100 chromosome, complete genome | 1824 | 37.26% | 90.35 |
| >gi|325926838|ref|NC_015219.1| Streptococcus gallolyticus subsp. gallolyticus ATCC BAA-2069 plasmid pSGG1, complete sequence | 12 | 37.26% | 83.33 |
| >gi|209967944|ref|NC_011314.1| Aliivibrio salmonicida LFI1238 plasmid pVSAL320, complete sequence | 6 | 37.28% | 100.00 |
| >gi|332290650|ref|NC_015496.1| Krokinobacter sp. 4H-3-7-5 chromosome, complete genome | 1333 | 37.29% | 90.25 |
| >gi|169825618|ref|NC_010382.1| Lysinibacillus sphaericus C3-41 chromosome, complete genome | 1797 | 37.29% | 91.10 |
| >gi|557607004|ref|NC_022780.1| Pediococcus pentosaceus SL4, complete genome | 656 | 37.30% | 91.16 |
| >gi|387789281|ref|NC_017770.1| Solitalea canadensis DSM 3403 chromosome, complete genome | 2114 | 37.31% | 88.84 |
| >gi|532354121|ref|NC_022111.1| Prevotella sp. oral taxon 299 str. F0039 plasmid, complete sequence | 954 | 37.32% | 88.78 |
| >gi|397661013|ref|NC_018108.1| Taylorella equigenitalis ATCC 35865 chromosome, complete genome | 615 | 37.34% | 92.36 |
| >gi|116491818|ref|NC_008525.1| Pediococcus pentosaceus ATCC 25745, complete genome | 702 | 37.36% | 90.88 |
| >gi|218442435|ref|NC_011737.1| Cyanothece sp. PCC 7424 plasmid pP742402, complete sequence | 96 | 37.37% | 92.71 |
| >gi|170717206|ref|NC_010519.1| Haemophilus somnus 2336 chromosome, complete genome | 1068 | 37.38% | 90.45 |
| >gi|336063372|ref|NC_015600.1| Streptococcus pasteurianus ATCC 43144, complete genome | 1171 | 37.38% | 87.53 |
| >gi|385227768|ref|NC_017380.1| Helicobacter pylori SNT49 plasmid pHPSNT, complete sequence | 6 | 37.38% | 83.33 |
| >gi|289433373|ref|NC_013891.1| Listeria seeligeri serovar 1/2b str. SLCC3954 chromosome, complete genome | 1382 | 37.38% | 89.22 |
| >gi|170016308|ref|NC_010470.1| Leuconostoc citreum KM20 plasmid pLCK1, complete sequence | 9 | 37.39% | 88.89 |
| >gi|385836839|ref|NC_017495.1| Lactococcus lactis subsp. cremoris A76 plasmid pQA518, complete sequence | 20 | 37.40% | 70.00 |
| >gi|220927459|ref|NC_011898.1| Clostridium cellulolyticum H10 chromosome, complete genome | 1583 | 37.40% | 91.41 |
| >gi|414079804|ref|NC_019440.1| Anabaena sp. 90 plasmid pANA03, complete sequence | 8 | 37.42% | 87.50 |
| >gi|319778189|ref|NC_014914.1| Taylorella equigenitalis MCE9 chromosome, complete genome | 600 | 37.42% | 92.33 |
| >gi|385219871|ref|NC_017364.1| Helicobacter pylori Gambia94/24 plasmid unnamed, complete sequence | 1 | 37.43% | 100.00 |
| >gi|54295843|ref|NC_006365.1| Legionella pneumophila str. Paris plasmid pLPP, complete sequence | 53 | 37.44% | 88.68 |
| >gi|16799079|ref|NC_003212.1| Listeria innocua Clip11262, complete genome | 1474 | 37.44% | 90.71 |
| >gi|189913115|ref|NC_010846.1| Leptospira biflexa serovar Patoc strain 'Patoc 1 (Ames)' plasmid p74, complete sequence | 44 | 37.47% | 88.64 |
| >gi|479196022|ref|NC_021036.1| Taylorella equigenitalis 14/56 draft genome | 558 | 37.47% | 92.83 |
| >gi|189913450|ref|NC_010844.1| Leptospira biflexa serovar Patoc strain 'Patoc 1 (Paris)' plasmid p74, complete sequence | 44 | 37.47% | 88.64 |
| >gi|385805051|ref|NC_017461.1| Fervidicoccus fontis Kam940 chromosome, complete genome | 1196 | 37.47% | 85.62 |
| >gi|327398173|ref|NC_015318.1| Hippea maritima DSM 10411 chromosome, complete genome | 806 | 37.47% | 89.95 |
| >gi|384516725|ref|NC_017312.1| Enterococcus faecalis 62 chromosome, complete genome | 1968 | 37.48% | 88.41 |
| >gi|409249552|ref|NT_187090.1| Salmonella enterica subsp. enterica serovar Weltevreden str. 2007-60-3289-1 | 0 | 37.48% | 0.00 |
| >gi|169786889|ref|NC_010404.1| Acinetobacter baumannii AYE plasmid p3ABAYE, complete sequence | 46 | 37.48% | 93.48 |
| >gi|397698583|ref|NC_018221.1| Enterococcus faecalis D32 chromosome, complete genome | 2095 | 37.49% | 88.26 |
| >gi|386336689|ref|NC_017576.1| Streptococcus gallolyticus subsp. gallolyticus ATCC 43143, complete genome | 1274 | 37.52% | 88.38 |
| >gi|29374661|ref|NC_004668.1| Enterococcus faecalis V583 chromosome, complete genome | 2096 | 37.53% | 89.27 |
| >gi|555077094|ref|NC_019954.2| Tepidanaerobacter acetatoxydans Re1 complete genome | 1257 | 37.53% | 91.09 |
| >gi|319891290|ref|NC_014925.1| Staphylococcus pseudintermedius HKU10-03 chromosome, complete genome | 556 | 37.53% | 92.63 |
| >gi|332798023|ref|NC_015519.1| Tepidanaerobacter sp. Re1 chromosome, complete genome | 1254 | 37.53% | 91.15 |
| >gi|427733619|ref|NC_019678.1| Rivularia sp. PCC 7116 chromosome, complete genome | 3452 | 37.54% | 88.82 |
| >gi|379704392|ref|NC_016826.1| Streptococcus infantarius subsp. infantarius CJ18 chromosome, complete genome | 1112 | 37.56% | 88.04 |
| >gi|538379295|ref|NC_022246.1| Streptococcus intermedius B196, complete genome | 1117 | 37.56% | 88.09 |
| >gi|20806542|ref|NC_003869.1| Thermoanaerobacter tengcongensis MB4 chromosome, complete genome | 2082 | 37.57% | 87.66 |
| >gi|381184504|ref|NC_016635.1| Pediococcus claussenii ATCC BAA-344 plasmid pPECL-1, complete sequence | 1 | 37.58% | 100.00 |
| >gi|169302992|ref|NC_010403.1| Acinetobacter baumannii AYE plasmid p4ABAYE, complete sequence | 0 | 37.60% | 0.00 |
| >gi|310830362|ref|NC_014628.1| Paenibacillus polymyxa SC2 plasmid pSC2, complete sequence | 226 | 37.61% | 91.15 |
| >gi|297660606|ref|NC_014226.1| Waddlia chondrophila WSU 86-1044 plasmid pWc, complete sequence | 14 | 37.61% | 71.43 |
| >gi|347533700|ref|NC_015979.1| Lactobacillus sanfranciscensis TMW 1.1304 plasmid pLS1, complete sequence | 16 | 37.62% | 93.75 |
| >gi|374337044|ref|NC_016749.1| Streptococcus macedonicus ACA-DC 198, complete genome | 1089 | 37.63% | 88.61 |
| >gi|330370666|ref|NC_015421.1| Lactobacillus buchneri NRRL B-30929 plasmid pLBUC03, complete sequence | 2 | 37.63% | 100.00 |
| >gi|384410883|ref|NC_017182.1| Zymomonas mobilis subsp. mobilis ATCC 10988 plasmid pZMOB05, complete sequence | 6 | 37.63% | 83.33 |
| >gi|288904223|ref|NC_013798.1| Streptococcus gallolyticus UCN34 chromosome, complete genome | 1236 | 37.64% | 87.46 |
| >gi|386318029|ref|NC_017568.1| Staphylococcus pseudintermedius ED99 chromosome, complete genome | 528 | 37.65% | 93.18 |
| >gi|325977200|ref|NC_015215.1| Streptococcus gallolyticus subsp. gallolyticus ATCC BAA-2069, complete genome | 1254 | 37.65% | 87.40 |
| >gi|538349851|ref|NC_022237.1| Streptococcus intermedius C270, complete genome | 1049 | 37.65% | 88.47 |
| >gi|527329219|ref|NC_021900.1| Streptococcus lutetiensis 033, complete genome | 1061 | 37.68% | 87.28 |
| >gi|336053510|ref|NC_015602.1| Lactobacillus kefiranofaciens ZW3 chromosome, complete genome | 977 | 37.70% | 87.82 |
| >gi|116617174|ref|NC_008531.1| Leuconostoc mesenteroides subsp. mesenteroides ATCC 8293 chromosome, complete genome | 596 | 37.71% | 94.13 |
| >gi|392427891|ref|NC_018073.1| Streptococcus intermedius JTH08, complete genome | 1026 | 37.71% | 89.08 |
| >gi|238915976|ref|NC_012778.1| Eubacterium eligens ATCC 27750 chromosome, complete genome | 721 | 37.71% | 88.49 |
| >gi|428765681|ref|NC_019770.1| Enterococcus faecalis str. Symbioflor 1, complete genome | 1860 | 37.72% | 88.39 |
| >gi|406025807|ref|NC_018611.1| Lactobacillus buchneri CD034 plasmid pCD034-3, complete sequence | 36 | 37.75% | 91.67 |
| >gi|384511964|ref|NC_017316.1| Enterococcus faecalis OG1RF chromosome, complete genome | 1847 | 37.75% | 88.63 |
| >gi|434401063|ref|NC_019749.1| Stanieria cyanosphaera PCC 7437 plasmid pSTA7437.02, complete sequence | 52 | 37.77% | 100.00 |
| >gi|381335653|ref|NC_016805.1| Leuconostoc mesenteroides subsp. mesenteroides J18 chromosome, complete genome | 567 | 37.77% | 93.30 |
| >gi|385836922|ref|NC_017497.1| Lactococcus lactis subsp. cremoris A76 plasmid pQA504, complete sequence | 5 | 37.83% | 100.00 |
| >gi|184159988|ref|NC_010605.1| Acinetobacter baumannii ACICU plasmid pACICU1, complete sequence | 14 | 37.84% | 85.71 |
| >gi|386045634|ref|NC_017545.1| Listeria monocytogenes J0161 chromosome, complete genome | 1457 | 37.86% | 89.84 |
| >gi|459466043|ref|NC_020449.1| Candidatus Cloacamonas acidaminovorans str. Evry provisional genome sequence from WWE1 candidate division | 924 | 37.87% | 88.31 |
| >gi|218960350|ref|NS_000195.1| Candidatus Cloacamonas acidaminovorans | 924 | 37.87% | 88.31 |
| >gi|42516522|ref|NC_002967.9| Treponema denticola ATCC 35405 chromosome, complete genome | 3181 | 37.87% | 86.36 |
| >gi|172034917|ref|NC_010546.1| Cyanothece sp. ATCC 51142 chromosome circular, complete sequence | 1899 | 37.88% | 88.10 |
| >gi|220930850|ref|NC_011899.1| Halothermothrix orenii H 168 chromosome, complete genome | 1324 | 37.88% | 88.22 |
| >gi|116490126|ref|NC_008528.1| Oenococcus oeni PSU-1, complete genome | 1332 | 37.89% | 88.74 |
| >gi|479188040|ref|NC_021031.1| Butyrivibrio fibrisolvens 16/4 draft genome | 1064 | 37.89% | 89.85 |
| >gi|339490182|ref|NC_015734.1| Leuconostoc sp. C2 chromosome, complete genome | 470 | 37.90% | 92.98 |
| >gi|513846967|ref|NC_021528.1| Lactobacillus plantarum 16 plasmid Lp16I, complete sequence | 9 | 37.91% | 100.00 |
| >gi|300885230|ref|NC_014333.1| Bacillus cereus biovar anthracis str. CI plasmid pBAslCI14, complete sequence | 3 | 37.91% | 100.00 |
| >gi|525924641|ref|NC_021838.1| Listeria monocytogenes strain R2-502, complete genome | 1435 | 37.93% | 90.38 |
| >gi|405754079|ref|NC_018586.1| Listeria monocytogenes SLCC2540, complete genome | 1426 | 37.93% | 90.46 |
| >gi|525732797|ref|NC_021830.1| Listeria monocytogenes J1-220, complete genome | 1415 | 37.94% | 90.39 |
| >gi|532435496|ref|NC_022124.1| Prevotella sp. oral taxon 299 str. F0039, complete genome | 371 | 37.95% | 89.22 |
| >gi|404282430|ref|NC_018588.1| Listeria monocytogenes SLCC2372, complete genome | 1405 | 37.95% | 90.04 |
| >gi|405756987|ref|NC_018589.1| Listeria monocytogenes SLCC2479, complete genome | 1403 | 37.95% | 90.02 |
| >gi|488570484|ref|NC_021171.1| Bacillus sp. 1NLA3E, complete genome | 2560 | 37.95% | 89.49 |
| >gi|662858600|ref|NC_013766.2| Listeria monocytogenes 08-5578, complete genome | 1436 | 37.96% | 89.90 |
| >gi|377810572|ref|NC_016636.1| Pediococcus claussenii ATCC BAA-344 plasmid pPECL-3, complete sequence | 3 | 37.96% | 100.00 |
| >gi|386048930|ref|NC_017546.1| Listeria monocytogenes FSL R2-561 chromosome, complete genome | 1407 | 37.96% | 90.26 |
| >gi|284993440|ref|NC_013768.1| Listeria monocytogenes 08-5923, complete genome | 1421 | 37.96% | 89.94 |
| >gi|170016236|ref|NC_010466.1| Leuconostoc citreum KM20 plasmid pLCK2, complete sequence | 16 | 37.97% | 75.00 |
| >gi|525865813|ref|NC_021823.1| Listeria monocytogenes strain C1-387, complete genome | 1422 | 37.97% | 90.15 |
| >gi|525916939|ref|NC_021837.1| Listeria monocytogenes strain J2-031, complete genome | 1443 | 37.97% | 89.60 |
| >gi|525721265|ref|NC_021829.1| Listeria monocytogenes J1816, complete genome | 1410 | 37.97% | 90.57 |
| >gi|525877702|ref|NC_021825.1| Listeria monocytogenes strain J2-1091, complete genome | 1412 | 37.98% | 90.72 |
| >gi|16802048|ref|NC_003210.1| Listeria monocytogenes EGD-e chromosome, complete genome | 1379 | 37.98% | 89.99 |
| >gi|525869762|ref|NC_021824.1| Listeria monocytogenes strain J2-064, complete genome | 1384 | 37.99% | 90.32 |
| >gi|525934212|ref|NC_021840.1| Listeria monocytogenes strain J1926, complete genome | 1406 | 37.99% | 90.68 |
| >gi|525929673|ref|NC_021839.1| Listeria monocytogenes strain J1776, complete genome | 1395 | 37.99% | 90.54 |
| >gi|405751204|ref|NC_018585.1| Listeria monocytogenes SLCC2378, complete genome | 1390 | 37.99% | 90.65 |
| >gi|525896797|ref|NC_021827.1| Listeria monocytogenes strain J1817, complete genome | 1416 | 37.99% | 90.47 |
| >gi|405748335|ref|NC_018584.1| Listeria monocytogenes ATCC 19117, complete genome | 1388 | 37.99% | 90.42 |
| >gi|296110131|ref|NC_014136.1| Leuconostoc kimchii IMSNU 11154 chromosome, complete genome | 501 | 38.00% | 92.02 |
| >gi|550896179|ref|NC_022568.1| Listeria monocytogenes EGD, complete genome | 1388 | 38.00% | 89.70 |
| >gi|71277742|ref|NC_003910.7| Colwellia psychrerythraea 34H chromosome, complete genome | 1243 | 38.01% | 92.20 |
| >gi|159902540|ref|NC_009976.1| Prochlorococcus marinus str. MIT 9211, complete genome | 746 | 38.01% | 89.14 |
| >gi|148826757|ref|NC_009567.1| Haemophilus influenzae PittGG chromosome, complete genome | 921 | 38.01% | 89.69 |
| >gi|404409239|ref|NC_018592.1| Listeria monocytogenes SLCC5850, complete genome | 1387 | 38.01% | 89.69 |
| >gi|404285367|ref|NC_018591.1| Listeria monocytogenes serotype 7 str. SLCC2482, complete genome | 1379 | 38.01% | 90.79 |
| >gi|525886094|ref|NC_021826.1| Listeria monocytogenes strain N1-011A, complete genome | 1430 | 38.01% | 90.42 |
| >gi|386265029|ref|NC_017452.1| Haemophilus influenzae R2846 chromosome, complete genome | 905 | 38.02% | 89.50 |
| >gi|56973315|ref|NC_004721.2| Bacillus cereus ATCC 14579 plasmid pBClin15, complete sequence | 2 | 38.03% | 100.00 |
| >gi|407719062|ref|NC_018674.1| Leuconostoc carnosum JB16 plasmid pKLC1, complete sequence | 5 | 38.03% | 100.00 |
| >gi|447911521|ref|NC_020207.1| Enterococcus faecium NRRL B-2354, complete genome | 1815 | 38.03% | 85.45 |
| >gi|386042347|ref|NC_017544.1| Listeria monocytogenes 10403S chromosome, complete genome | 1375 | 38.03% | 89.82 |
| >gi|424712850|ref|NC_019556.1| Listeria monocytogenes serotype 4b str. LL195, complete genome | 1367 | 38.04% | 90.64 |
| >gi|148825133|ref|NC_009566.1| Haemophilus influenzae PittEE chromosome, complete genome | 912 | 38.04% | 89.47 |
| >gi|404412106|ref|NC_018593.1| Listeria monocytogenes SLCC7179, complete genome | 1373 | 38.04% | 89.95 |
| >gi|85700163|ref|NC_002973.6| Listeria monocytogenes serotype 4b str. F2365 chromosome, complete genome | 1369 | 38.04% | 90.65 |
| >gi|386730761|ref|NC_017728.1| Listeria monocytogenes 07PF0776 chromosome, complete genome | 1378 | 38.04% | 90.35 |
| >gi|386052273|ref|NC_017547.1| Listeria monocytogenes Finland 1998 chromosome, complete genome | 1376 | 38.05% | 90.04 |
| >gi|543951066|ref|NC_022356.1| Haemophilus influenzae KR494, complete genome | 880 | 38.05% | 90.80 |
| >gi|240849682|ref|NC_012846.1| Bartonella grahamii as4aup, complete genome | 1703 | 38.06% | 90.14 |
| >gi|226222639|ref|NC_012488.1| Listeria monocytogenes Clip81459, complete genome | 1379 | 38.06% | 90.57 |
| >gi|406702775|ref|NC_018642.1| Listeria monocytogenes L312, complete genome | 1379 | 38.06% | 90.57 |
| >gi|443329695|ref|NC_020157.1| Anabaena cylindrica PCC 7122 plasmid pANACY.02, complete sequence | 51 | 38.08% | 88.24 |
| >gi|386263233|ref|NC_017451.1| Haemophilus influenzae R2866, complete genome | 941 | 38.08% | 88.52 |
| >gi|404279552|ref|NC_018587.1| Listeria monocytogenes SLCC2755, complete genome | 1376 | 38.08% | 90.77 |
| >gi|330818917|ref|NC_015420.1| Lactobacillus buchneri NRRL B-30929 plasmid pLBUC01, complete sequence | 24 | 38.09% | 83.33 |
| >gi|317133719|ref|NC_014824.1| Ruminococcus albus 7 plasmid pRUMAL01, complete sequence | 224 | 38.09% | 88.39 |
| >gi|397668686|ref|NC_018141.1| Legionella pneumophila subsp. pneumophila str. Lorraine plasmid pLELO, complete sequence | 41 | 38.09% | 95.12 |
| >gi|172034885|ref|NC_010542.1| Cyanothece sp. ATCC 51142 plasmid C, complete sequence | 12 | 38.09% | 75.00 |
| >gi|209809832|ref|NC_011315.1| Aliivibrio salmonicida LFI1238 plasmid pVSAL54, complete sequence | 2 | 38.10% | 100.00 |
| >gi|302668499|ref|NC_014390.1| Butyrivibrio proteoclasticus B316 plasmid pCY186, complete sequence | 85 | 38.10% | 89.41 |
| >gi|470208773|ref|NC_020558.1| Listeria monocytogenes N53-1 complete genome | 1316 | 38.10% | 89.67 |
| >gi|414075311|ref|NC_019427.1| Anabaena sp. 90 chromosome chANA01, complete sequence | 1633 | 38.10% | 81.81 |
| >gi|470205641|ref|NC_020557.1| Listeria monocytogenes La111 complete genome | 1314 | 38.11% | 89.57 |
| >gi|295702242|ref|NC_014103.1| Bacillus megaterium DSM 319 chromosome, complete genome | 3018 | 38.13% | 88.10 |
| >gi|538353400|ref|NC_022238.1| Streptococcus constellatus subsp. pharyngis C1050, complete genome | 986 | 38.13% | 87.42 |
| >gi|378696079|ref|NC_016809.1| Haemophilus influenzae 10810, complete genome | 936 | 38.14% | 90.17 |
| >gi|343082721|ref|NC_015914.1| Cyclobacterium marinum DSM 745 chromosome, complete genome | 3155 | 38.15% | 89.41 |
| >gi|385816611|ref|NC_017470.1| Lactobacillus amylovorus GRL1118 chromosome, complete genome | 879 | 38.15% | 89.76 |
| >gi|325955721|ref|NC_015214.1| Lactobacillus acidophilus 30SC chromosome, complete genome | 895 | 38.15% | 88.04 |
| >gi|16271976|ref|NC_000907.1| Haemophilus influenzae Rd KW20 chromosome, complete genome | 842 | 38.15% | 90.02 |
| >gi|389867183|ref|NC_017960.1| Enterococcus faecium DO chromosome, complete genome | 1803 | 38.15% | 86.02 |
| >gi|162960935|ref|NC_007146.2| Haemophilus influenzae 86-028NP chromosome, complete genome | 901 | 38.16% | 90.46 |
| >gi|218442325|ref|NC_011733.1| Cyanothece sp. PCC 7424 plasmid pP742405, complete sequence | 7 | 38.17% | 85.71 |
| >gi|378775961|ref|NC_016811.1| Legionella pneumophila subsp. pneumophila ATCC 43290 chromosome, complete genome | 1268 | 38.17% | 90.77 |
| >gi|313669532|ref|NC_014756.1| Sulfuricurvum kujiense DSM 16994 plasmid pSULKU03, complete sequence | 18 | 38.18% | 94.44 |
| >gi|109946640|ref|NC_008229.1| Helicobacter acinonychis str. Sheeba chromosome, complete genome | 1506 | 38.18% | 89.04 |
| >gi|538346599|ref|NC_022236.1| Streptococcus constellatus subsp. pharyngis C232, complete genome | 957 | 38.18% | 87.36 |
| >gi|538374242|ref|NC_022245.1| Streptococcus constellatus subsp. pharyngis C818, complete genome | 957 | 38.18% | 87.36 |
| >gi|308051505|ref|NC_014544.1| Legionella longbeachae NSW150 plasmid pLLO, complete sequence | 35 | 38.19% | 94.29 |
| >gi|386006713|ref|NC_017529.1| Listeria monocytogenes L99, complete genome | 1416 | 38.19% | 89.48 |
| >gi|386025288|ref|NC_017537.1| Listeria monocytogenes M7 chromosome, complete genome | 1414 | 38.19% | 89.53 |
| >gi|217963303|ref|NC_011660.1| Listeria monocytogenes HCC23 chromosome, complete genome | 1424 | 38.19% | 89.47 |
| >gi|315037230|ref|NC_014724.1| Lactobacillus amylovorus GRL 1112 chromosome, complete genome | 950 | 38.20% | 88.11 |
| >gi|436843263|ref|NC_020056.1| Anabaena cylindrica PCC 7122 plasmid pANACY.05, complete sequence | 8 | 38.20% | 100.00 |
| >gi|414079097|ref|NC_019439.1| Anabaena sp. 90 chromosome chANA02, complete sequence | 361 | 38.21% | 80.89 |
| >gi|33151282|ref|NC_002940.2| Haemophilus ducreyi 35000HP chromosome, complete genome | 550 | 38.22% | 89.82 |
| >gi|319774951|ref|NC_014922.1| Haemophilus influenzae F3047 chromosome, complete genome | 929 | 38.22% | 89.88 |
| >gi|319896422|ref|NC_014920.1| Haemophilus influenzae F3031 chromosome, complete genome | 934 | 38.22% | 89.29 |
| >gi|49474831|ref|NC_005956.1| Bartonella henselae str. Houston-1 chromosome, complete genome | 1484 | 38.23% | 87.94 |
| >gi|509149353|ref|NC_021350.1| Legionella pneumophila subsp. pneumophila str. Thunder Bay, complete genome | 1292 | 38.23% | 90.48 |
| >gi|538357775|ref|NC_022239.1| Streptococcus anginosus C238, complete genome | 1197 | 38.23% | 87.05 |
| >gi|209808847|ref|NC_011313.1| Aliivibrio salmonicida LFI1238 chromosome 2, complete sequence | 259 | 38.24% | 91.89 |
| >gi|121601635|ref|NC_008783.1| Bartonella bacilliformis KC583, complete genome | 1040 | 38.24% | 87.40 |
| >gi|529232713|ref|NC_021994.1| Enterococcus faecium Aus0085, complete genome | 1999 | 38.24% | 86.14 |
| >gi|384044176|ref|NC_017138.1| Bacillus megaterium WSH-002 chromosome, complete genome | 2921 | 38.24% | 87.85 |
| >gi|294496875|ref|NC_014019.1| Bacillus megaterium QM B1551 chromosome, complete genome | 2919 | 38.26% | 87.74 |
| >gi|404406483|ref|NC_018590.1| Listeria monocytogenes SLCC2376, complete genome | 1331 | 38.26% | 90.61 |
| >gi|93004786|ref|NC_007968.1| Psychrobacter cryohalolentis K5 plasmid 1, complete sequence | 9 | 38.26% | 88.89 |
| >gi|52840256|ref|NC_002942.5| Legionella pneumophila subsp. pneumophila str. Philadelphia 1 chromosome, complete genome | 1259 | 38.27% | 91.02 |
| >gi|338731758|ref|NC_015710.1| Simkania negevensis Z plasmid pSn, complete sequence | 72 | 38.28% | 86.11 |
| >gi|381335622|ref|NC_016821.1| Leuconostoc mesenteroides subsp. mesenteroides J18 plasmid pKLE03, complete sequence | 3 | 38.28% | 100.00 |
| >gi|148641524|ref|NC_009516.1| Psychrobacter sp. PRwf-1 plasmid pRWF101, complete sequence | 3 | 38.28% | 66.67 |
| >gi|256823891|ref|NC_013168.1| Cyanothece sp. PCC 8802 plasmid pP880204, complete sequence | 6 | 38.29% | 83.33 |
| >gi|336322299|ref|NC_015672.1| Flexistipes sinusarabici DSM 4947 chromosome, complete genome | 1651 | 38.29% | 88.25 |
| >gi|342164902|ref|NC_015876.1| Streptococcus pseudopneumoniae IS7493 plasmid pDRPIS7493, complete sequence | 4 | 38.31% | 50.00 |
| >gi|348589352|ref|NC_016043.1| Taylorella asinigenitalis MCE3 chromosome, complete genome | 558 | 38.31% | 90.86 |
| >gi|94993396|ref|NC_008024.1| Streptococcus pyogenes MGAS10750 chromosome, complete genome | 936 | 38.32% | 87.39 |
| >gi|331746790|ref|NC_015471.1| Thermococcus barophilus MP plasmid pTBMP1, complete sequence | 22 | 38.32% | 90.91 |
| >gi|470180668|ref|NC_020521.1| Legionella pneumophila subsp. pneumophila LPE509 chromosome, complete genome | 1270 | 38.33% | 91.42 |
| >gi|471314537|ref|NC_020555.1| Helicobacter cinaedi ATCC BAA-847 DNA, complete genome | 1027 | 38.34% | 89.29 |
| >gi|347825839|ref|NC_016035.1| Lactobacillus buchneri CD034 plasmid pCD034-1, complete sequence | 1 | 38.35% | 100.00 |
| >gi|397665626|ref|NC_018140.1| Legionella pneumophila subsp. pneumophila, complete genome | 1239 | 38.35% | 91.53 |
| >gi|312193364|ref|NC_014629.1| Aggregatibacter actinomycetemcomitans D11S-1 plasmid S57, complete sequence | 23 | 38.35% | 100.00 |
| >gi|71902667|ref|NC_007296.1| Streptococcus pyogenes MGAS6180 chromosome, complete genome | 930 | 38.35% | 87.31 |
| >gi|383327320|ref|NC_017022.1| Enterococcus faecium Aus0004 chromosome, complete genome | 1937 | 38.36% | 86.11 |
| >gi|397662556|ref|NC_018139.1| Legionella pneumophila subsp. pneumophila, complete genome | 1207 | 38.36% | 91.80 |
| >gi|440685202|ref|NC_019773.1| Anabaena cylindrica PCC 7122 plasmid pANACY.03, complete sequence | 42 | 38.37% | 92.86 |
| >gi|54295983|ref|NC_006368.1| Legionella pneumophila str. Paris, complete genome | 1271 | 38.37% | 91.82 |
| >gi|296105497|ref|NC_014125.1| Legionella pneumophila 2300/99 Alcoy chromosome, complete genome | 1284 | 38.38% | 91.98 |
| >gi|386037981|ref|NC_017543.1| Paenibacillus polymyxa M1 plasmid pPPM1a, complete sequence | 118 | 38.38% | 92.37 |
| >gi|54292907|ref|NC_006366.1| Legionella pneumophila str. Lens plasmid pLPL, complete sequence | 28 | 38.39% | 89.29 |
| >gi|479191087|ref|NC_021033.1| Taylorella asinigenitalis 14/45 draft genome | 495 | 38.40% | 90.30 |
| >gi|54292964|ref|NC_006369.1| Legionella pneumophila str. Lens, complete genome | 1267 | 38.41% | 91.24 |
| >gi|59714356|ref|NC_006842.1| Vibrio fischeri ES114 plasmid pES100, complete sequence | 16 | 38.42% | 100.00 |
| >gi|414075302|ref|NC_019437.1| Lactococcus lactis subsp. cremoris UC509.9 plasmid pCIS4, complete sequence | 0 | 38.42% | 0.00 |
| >gi|94989509|ref|NC_008022.1| Streptococcus pyogenes MGAS10270 chromosome, complete genome | 916 | 38.43% | 87.66 |
| >gi|307591951|ref|NC_014533.1| Cyanothece sp. PCC 7822 plasmid Cy782201, complete sequence | 492 | 38.44% | 89.84 |
| >gi|298489614|ref|NC_014248.1| 'Nostoc azollae' 0708 chromosome, complete genome | 1457 | 38.45% | 87.92 |
| >gi|428303567|ref|NC_019728.1| Calothrix sp. PCC 6303 plasmid pCAL6303.02, complete sequence | 21 | 38.47% | 90.48 |
| >gi|383493132|ref|NC_017053.1| Streptococcus pyogenes MGAS1882 chromosome, complete genome | 879 | 38.48% | 87.71 |
| >gi|295815281|ref|NC_009494.2| Legionella pneumophila str. Corby chromosome, complete genome | 1301 | 38.48% | 91.93 |
| >gi|383479207|ref|NC_017040.1| Streptococcus pyogenes MGAS15252 chromosome, complete genome | 847 | 38.48% | 87.72 |
| >gi|385221504|ref|NC_017361.1| Helicobacter pylori SouthAfrica7 chromosome, complete genome | 1699 | 38.50% | 88.46 |
| >gi|521206439|ref|NC_021669.1| Psychrobacter sp. G plasmid PsyG_3, complete sequence | 1 | 38.50% | 100.00 |
| >gi|75812629|ref|NC_007411.1| Anabaena variabilis ATCC 29413 plasmid B, complete sequence | 16 | 38.50% | 100.00 |
| >gi|523414886|ref|NC_020540.2| Streptococcus pyogenes M1 476 DNA, complete genome | 874 | 38.50% | 87.19 |
| >gi|434408147|ref|NC_019758.1| Cylindrospermum stagnale PCC 7417 plasmid pCYLST.03, complete sequence | 6 | 38.50% | 83.33 |
| >gi|15674250|ref|NC_002737.1| Streptococcus pyogenes SF370 chromosome, complete genome | 893 | 38.51% | 87.01 |
| >gi|116334879|ref|NC_008499.1| Lactobacillus brevis ATCC 367 plasmid 2, complete sequence | 14 | 38.51% | 92.86 |
| >gi|71909814|ref|NC_007297.1| Streptococcus pyogenes MGAS5005 chromosome, complete genome | 881 | 38.53% | 87.06 |
| >gi|409913960|ref|NC_018936.1| Streptococcus pyogenes A20 chromosome, complete genome | 878 | 38.54% | 87.13 |
| >gi|222530712|ref|NC_012036.1| Caldicellulosiruptor bescii DSM 6725 plasmid pATHE01, complete sequence | 9 | 38.54% | 77.78 |
| >gi|94987631|ref|NC_008021.1| Streptococcus pyogenes MGAS9429 chromosome, complete genome | 878 | 38.54% | 87.02 |
| >gi|525808973|ref|NC_021807.1| Streptococcus pyogenes HSC5, complete genome | 854 | 38.54% | 88.52 |
| >gi|19745201|ref|NC_003485.1| Streptococcus pyogenes MGAS8232 chromosome, complete genome | 887 | 38.55% | 88.16 |
| >gi|28894912|ref|NC_004606.1| Streptococcus pyogenes SSI-1 chromosome, complete genome | 916 | 38.55% | 87.55 |
| >gi|532530467|ref|NC_022130.1| Helicobacter pylori SouthAfrica20, complete genome | 1602 | 38.57% | 88.51 |
| >gi|209558587|ref|NC_011375.1| Streptococcus pyogenes NZ131 chromosome, complete genome | 854 | 38.57% | 86.89 |
| >gi|172054853|ref|NC_010547.1| Cyanothece sp. ATCC 51142 chromosome linear, complete sequence | 142 | 38.57% | 85.92 |
| >gi|480527845|ref|NC_017525.1| Legionella pneumophila subsp. pneumophila str. Hextuple_2q chromosome, complete genome | 952 | 38.58% | 90.02 |
| >gi|480527846|ref|NC_017526.1| Legionella pneumophila subsp. pneumophila str. Hextuple_3a chromosome, complete genome | 952 | 38.58% | 90.02 |
| >gi|386361880|ref|NC_017596.1| Streptococcus pyogenes Alab49 chromosome, complete genome | 878 | 38.58% | 87.36 |
| >gi|529235720|ref|NC_022000.1| Proteus mirabilis BB2000, complete genome | 1509 | 38.59% | 91.05 |
| >gi|21909536|ref|NC_004070.1| Streptococcus pyogenes MGAS315 chromosome, complete genome | 904 | 38.59% | 87.50 |
| >gi|393198727|ref|NC_018065.1| Solibacillus silvestris StLB046, complete genome | 1406 | 38.60% | 91.89 |
| >gi|347825835|ref|NC_016034.1| Lactobacillus buchneri CD034 plasmid pCD034-2, complete sequence | 1 | 38.60% | 100.00 |
| >gi|218437013|ref|NC_011729.1| Cyanothece sp. PCC 7424 chromosome, complete genome | 2895 | 38.61% | 85.66 |
| >gi|558684762|ref|NC_022878.1| Enterococcus mundtii QU 25 DNA, complete genome | 1620 | 38.61% | 87.84 |
| >gi|470478036|ref|NC_020802.1| Psychromonas sp. CNPT3, complete genome | 1120 | 38.62% | 90.36 |
| >gi|528816008|ref|NC_021218.2| Helicobacter pylori UM066, complete genome | 1542 | 38.62% | 88.20 |
| >gi|512594165|ref|NC_021496.1| Lactobacillus reuteri I5007 plasmid pLRI02, complete sequence | 5 | 38.62% | 100.00 |
| >gi|139472888|ref|NC_009332.1| Streptococcus pyogenes str. Manfredo chromosome, complete genome | 855 | 38.63% | 88.42 |
| >gi|269797069|ref|NC_013520.1| Veillonella parvula DSM 2008 chromosome, complete genome | 414 | 38.63% | 91.79 |
| >gi|386760820|ref|NC_017761.1| Helicobacter cinaedi PAGU611, complete genome | 941 | 38.63% | 89.16 |
| >gi|116334867|ref|NC_008498.1| Lactobacillus brevis ATCC 367 plasmid 1, complete sequence | 7 | 38.64% | 85.71 |
| >gi|328949383|ref|NC_015386.1| Treponema succinifaciens DSM 2489 plasmid pTRESU01, complete sequence | 103 | 38.66% | 87.38 |
| >gi|428771848|ref|NC_019778.1| Cyanobacterium stanieri PCC 7202 chromosome, complete genome | 1897 | 38.66% | 81.18 |
| >gi|387907368|ref|NC_017926.1| Helicobacter pylori XZ274 chromosome, complete genome | 1530 | 38.67% | 88.43 |
| >gi|50913346|ref|NC_006086.1| Streptococcus pyogenes MGAS10394 chromosome, complete genome | 899 | 38.69% | 86.76 |
| >gi|344915391|ref|NC_014558.2| Lactobacillus plantarum subsp. plantarum ST-III plasmid pST-III, complete sequence | 9 | 38.69% | 100.00 |
| >gi|385839804|ref|NC_017480.1| Lactobacillus salivarius CECT 5713 plasmid pHN2, complete sequence | 6 | 38.69% | 83.33 |
| >gi|528821563|ref|NC_021904.1| Lactobacillus plantarum ZJ316 plasmid pLP-ZJ102, complete sequence | 3 | 38.69% | 100.00 |
| >gi|157362870|ref|NC_009828.1| Thermotoga lettingae TMO chromosome, complete genome | 1165 | 38.70% | 89.70 |
| >gi|385224642|ref|NC_017375.1| Helicobacter pylori 83 chromosome, complete genome | 1505 | 38.72% | 88.37 |
| >gi|557820703|ref|NC_022792.1| Thermotoga elfii NBRC 107921 DNA, complete genome | 1173 | 38.72% | 89.86 |
| >gi|385248496|ref|NC_017367.1| Helicobacter pylori F57, complete genome | 1532 | 38.73% | 88.25 |
| >gi|94991497|ref|NC_008023.1| Streptococcus pyogenes MGAS2096 chromosome, complete genome | 864 | 38.73% | 87.27 |
| >gi|470164400|ref|NC_020508.1| Helicobacter pylori OK113 DNA, complete genome | 1525 | 38.73% | 87.87 |
| >gi|299768250|ref|NC_014259.1| Acinetobacter oleivorans DR1 chromosome, complete genome | 1557 | 38.73% | 90.69 |
| >gi|403529933|ref|NC_018533.1| Bartonella quintana RM-11 chromosome, complete genome | 1085 | 38.76% | 88.94 |
| >gi|385831969|ref|NC_017490.1| Lactococcus garvieae Lg2, complete genome | 1159 | 38.77% | 86.89 |
| >gi|387781698|ref|NC_017382.1| Helicobacter pylori 51 chromosome, complete genome | 1507 | 38.77% | 88.19 |
| >gi|392376213|ref|NC_014796.1| Chlamydophila psittaci RD1, complete genome | 790 | 38.77% | 86.33 |
| >gi|386750444|ref|NC_017739.1| Helicobacter pylori Shi417 chromosome, complete genome | 1532 | 38.77% | 88.51 |
| >gi|386753519|ref|NC_017741.1| Helicobacter pylori Shi112 chromosome, complete genome | 1485 | 38.77% | 88.89 |
| >gi|478429022|ref|NC_020509.1| Helicobacter pylori OK310 DNA, complete genome | 1477 | 38.78% | 87.81 |
| >gi|526230725|ref|NC_021872.1| Lactobacillus reuteri TD1, complete genome | 653 | 38.78% | 87.14 |
| >gi|381335651|ref|NC_016806.1| Leuconostoc mesenteroides subsp. mesenteroides J18 plasmid pKLE05, complete sequence | 0 | 38.78% | 0.00 |
| >gi|407458794|ref|NC_018623.1| Chlamydia psittaci M56 chromosome, complete genome | 830 | 38.79% | 86.87 |
| >gi|298735500|ref|NC_014256.1| Helicobacter pylori B8 chromosome, complete genome | 1617 | 38.79% | 87.82 |
| >gi|440679730|ref|NC_019771.1| Anabaena cylindrica PCC 7122, complete genome | 1991 | 38.80% | 86.24 |
| >gi|49473688|ref|NC_005955.1| Bartonella quintana str. Toulouse, complete genome | 1049 | 38.80% | 89.23 |
| >gi|425788638|ref|NC_019560.1| Helicobacter pylori Aklavik117 chromosome, complete genome | 1520 | 38.80% | 88.88 |
| >gi|210134201|ref|NC_011498.1| Helicobacter pylori P12 chromosome, complete genome | 1656 | 38.81% | 88.83 |
| >gi|385858893|ref|NC_017520.1| Mycoplasma haemofelis Ohio2 chromosome, complete genome | 511 | 38.81% | 84.34 |
| >gi|529858346|ref|NC_021216.2| Helicobacter pylori UM299, complete genome | 1493 | 38.81% | 88.35 |
| >gi|381184514|ref|NC_016606.1| Pediococcus claussenii ATCC BAA-344 plasmid pPECL-2, complete sequence | 0 | 38.82% | 0.00 |
| >gi|526465356|ref|NC_021882.1| Helicobacter pylori UM298, complete genome | 1513 | 38.82% | 88.24 |
| >gi|390939174|ref|NC_018002.1| Sulfurospirillum barnesii SES-3 chromosome, complete genome | 1595 | 38.82% | 87.90 |
| >gi|385229315|ref|NC_017379.1| Helicobacter pylori Puno135 chromosome, complete genome | 1516 | 38.82% | 89.38 |
| >gi|375133618|ref|NC_016603.1| Acinetobacter calcoaceticus PHEA-2 chromosome, complete genome | 1502 | 38.82% | 91.15 |
| >gi|529973389|ref|NC_021215.2| Helicobacter pylori UM032, complete genome | 1484 | 38.82% | 88.27 |
| >gi|347520606|ref|NC_015930.1| Lactococcus garvieae ATCC 49156, complete genome | 1137 | 38.83% | 86.63 |
| >gi|384898367|ref|NC_017365.1| Helicobacter pylori F30, complete genome | 1475 | 38.83% | 89.02 |
| >gi|451941309|ref|NC_020301.1| Bartonella vinsonii subsp. berkhoffii str. Winnie, complete genome | 1255 | 38.83% | 88.53 |
| >gi|428308218|ref|NC_019755.1| Crinalium epipsammum PCC 9333 plasmid pCRI9333.06, complete sequence | 20 | 38.84% | 90.00 |
| >gi|110636427|ref|NC_008255.1| Cytophaga hutchinsonii ATCC 33406 chromosome, complete genome | 1806 | 38.85% | 90.86 |
| >gi|321309518|ref|NC_014970.1| Mycoplasma haemofelis str. Langford 1, complete genome | 504 | 38.85% | 84.92 |
| >gi|218203844|ref|NC_011721.1| Cyanothece sp. PCC 8801 plasmid pP880101, complete sequence | 30 | 38.85% | 96.67 |
| >gi|163867306|ref|NC_010161.1| Bartonella tribocorum CIP 105476 chromosome, complete genome | 1914 | 38.86% | 89.18 |
| >gi|313202490|ref|NC_014734.1| Paludibacter propionicigenes WB4 chromosome, complete genome | 1618 | 38.86% | 89.62 |
| >gi|385215269|ref|NC_017366.1| Helicobacter pylori F32 chromosome, complete genome | 1464 | 38.86% | 88.39 |
| >gi|384892008|ref|NC_017358.1| Helicobacter pylori Cuz20 chromosome, complete genome | 1463 | 38.86% | 89.13 |
| >gi|386751993|ref|NC_017740.1| Helicobacter pylori Shi169 chromosome, complete genome | 1412 | 38.86% | 88.88 |
| >gi|523529121|ref|NC_021733.1| Acinetobacter baumannii BJAB0715, complete genome | 1512 | 38.87% | 90.94 |
| >gi|384895178|ref|NC_017360.1| Helicobacter pylori 35A chromosome, complete genome | 1449 | 38.87% | 88.27 |
| >gi|148543243|ref|NC_009513.1| Lactobacillus reuteri DSM 20016 chromosome, complete genome | 593 | 38.87% | 87.35 |
| >gi|410023236|ref|NC_018937.1| Helicobacter pylori Rif1 chromosome, complete genome | 1603 | 38.87% | 87.27 |
| >gi|410024832|ref|NC_018939.1| Helicobacter pylori 26695 chromosome, complete genome | 1601 | 38.87% | 87.32 |
| >gi|15644634|ref|NC_000915.1| Helicobacter pylori 26695 chromosome, complete genome | 1601 | 38.87% | 87.32 |
| >gi|410081538|ref|NC_018938.1| Helicobacter pylori Rif2 chromosome, complete genome | 1601 | 38.87% | 87.32 |
| >gi|197333880|ref|NC_011184.1| Vibrio fischeri MJ11 chromosome I, complete sequence | 741 | 38.87% | 89.74 |
| >gi|560153071|ref|NC_022911.1| Helicobacter pylori BM012S, complete genome | 1529 | 38.88% | 88.23 |
| >gi|383785713|ref|NC_017095.1| Fervidobacterium pennivorans DSM 9078 chromosome, complete genome | 762 | 38.88% | 85.56 |
| >gi|560107639|ref|NC_022886.1| Helicobacter pylori BM012A, complete genome | 1525 | 38.88% | 88.33 |
| >gi|184152655|ref|NC_010609.1| Lactobacillus reuteri JCM 1112, complete genome | 603 | 38.88% | 87.56 |
| >gi|385216759|ref|NC_017368.1| Helicobacter pylori F16, complete genome | 1440 | 38.88% | 88.89 |
| >gi|383749063|ref|NC_017063.1| Helicobacter pylori ELS37 chromosome, complete genome | 1606 | 38.89% | 87.80 |
| >gi|189909570|ref|NC_010842.1| Leptospira biflexa serovar Patoc strain 'Patoc 1 (Ames)' chromosome I, complete sequence | 1990 | 38.89% | 87.99 |
| >gi|208433976|ref|NC_011333.1| Helicobacter pylori G27 chromosome, complete genome | 1537 | 38.89% | 88.42 |
| >gi|530280362|ref|NC_021217.2| Helicobacter pylori UM037, complete genome | 1548 | 38.89% | 88.70 |
| >gi|183219427|ref|NC_010602.1| Leptospira biflexa serovar Patoc strain 'Patoc 1 (Paris)' chromosome I, complete sequence | 1990 | 38.89% | 87.99 |
| >gi|385219873|ref|NC_017372.1| Helicobacter pylori India7 chromosome, complete genome | 1556 | 38.90% | 87.85 |
| >gi|308183796|ref|NC_014560.1| Helicobacter pylori SJM180 chromosome, complete genome | 1585 | 38.90% | 87.13 |
| >gi|197283915|ref|NC_010554.1| Proteus mirabilis HI4320 chromosome, complete genome | 1643 | 38.90% | 90.51 |
| >gi|209406223|ref|NC_010698.2| Helicobacter pylori Shi470 chromosome, complete genome | 1453 | 38.91% | 89.40 |
| >gi|384896752|ref|NC_017362.1| Helicobacter pylori Lithuania75 chromosome, complete genome | 1557 | 38.92% | 87.93 |
| >gi|386343608|ref|NC_017581.1| Streptococcus thermophilus JIM 8232, complete genome | 791 | 38.92% | 87.74 |
| >gi|529230090|ref|NC_021990.1| Enterococcus faecium Aus0085 plasmid p6, complete sequence | 5 | 38.92% | 60.00 |
| >gi|385227773|ref|NC_017378.1| Helicobacter pylori Puno120 chromosome, complete genome | 1523 | 38.93% | 88.84 |
| >gi|558687879|ref|NC_022881.1| Enterococcus mundtii QU 25 plasmid pQY003 DNA, complete genome | 6 | 38.93% | 66.67 |
| >gi|126640115|ref|NC_009085.1| Acinetobacter baumannii ATCC 17978 chromosome, complete genome | 1512 | 38.94% | 91.14 |
| >gi|384887043|ref|NC_017354.1| Helicobacter pylori 52 chromosome, complete genome | 1447 | 38.94% | 88.87 |
| >gi|308182188|ref|NC_014555.1| Helicobacter pylori PeCan4 chromosome, complete genome | 1551 | 38.94% | 87.88 |
| >gi|302668698|ref|NC_014389.1| Butyrivibrio proteoclasticus B316 plasmid pCY360, complete sequence | 122 | 38.95% | 84.43 |
| >gi|172087630|ref|NC_006840.2| Vibrio fischeri ES114 chromosome I, complete sequence | 780 | 38.95% | 89.74 |
| >gi|523521389|ref|NC_021726.1| Acinetobacter baumannii BJAB07104, complete genome | 1531 | 38.96% | 90.86 |
| >gi|406592921|ref|NC_018626.1| Chlamydia psittaci NJ1 chromosome, complete genome | 808 | 38.96% | 86.01 |
| >gi|512595901|ref|NC_021498.1| Lactobacillus reuteri I5007 plasmid pLRI06, complete sequence | 1 | 38.96% | 100.00 |
| >gi|410857988|ref|NC_019391.1| Chlamydia psittaci 01DC12, complete genome | 809 | 38.96% | 85.78 |
| >gi|268678642|ref|NC_013512.1| Sulfurospirillum deleyianum DSM 6946 chromosome, complete genome | 1386 | 38.97% | 88.96 |
| >gi|384888657|ref|NC_017355.1| Helicobacter pylori v225d chromosome, complete genome | 1413 | 38.97% | 88.54 |
| >gi|538369494|ref|NC_022244.1| Streptococcus anginosus C1051, complete genome | 961 | 38.97% | 87.72 |
| >gi|125972525|ref|NC_009012.1| Clostridium thermocellum ATCC 27405 chromosome, complete genome | 1780 | 38.99% | 89.61 |
| >gi|512590512|ref|NC_021494.1| Lactobacillus reuteri I5007, complete genome | 589 | 38.99% | 86.42 |
| >gi|170016358|ref|NC_010471.1| Leuconostoc citreum KM20, complete genome | 352 | 38.99% | 92.90 |
| >gi|307149708|ref|NC_014502.1| Cyanothece sp. PCC 7822 plasmid Cy782203, complete sequence | 121 | 38.99% | 85.12 |
| >gi|383315346|ref|NC_017023.1| Enterococcus faecium Aus0004 plasmid AUS0004_p2, complete sequence | 7 | 38.99% | 85.71 |
| >gi|469820182|ref|NC_020547.1| Acinetobacter baumannii D1279779, complete genome | 1413 | 39.00% | 90.59 |
| >gi|385226252|ref|NC_017376.1| Helicobacter pylori SNT49 chromosome, complete genome | 1446 | 39.00% | 89.07 |
| >gi|386755084|ref|NC_017742.1| Helicobacter pylori PeCan18 chromosome, complete genome | 1567 | 39.02% | 88.07 |
| >gi|407456172|ref|NC_018621.1| Chlamydia psittaci VS225 chromosome, complete genome | 785 | 39.02% | 86.11 |
| >gi|407930685|ref|NC_018706.1| Acinetobacter baumannii TYTH-1 chromosome, complete genome | 1515 | 39.02% | 90.69 |
| >gi|385235550|ref|NC_017387.1| Acinetobacter baumannii TCDC-AB0715 chromosome, complete genome | 1575 | 39.02% | 90.92 |
| >gi|523525298|ref|NC_021729.1| Acinetobacter baumannii BJAB0868, complete genome | 1489 | 39.02% | 90.73 |
| >gi|338173994|ref|NC_015702.1| Parachlamydia acanthamoebae UV-7 chromosome, complete genome | 2097 | 39.03% | 88.84 |
| >gi|407457548|ref|NC_018622.1| Chlamydia psittaci WS/RT/E30 chromosome, complete genome | 792 | 39.03% | 85.61 |
| >gi|184156320|ref|NC_010611.1| Acinetobacter baumannii ACICU chromosome, complete genome | 1490 | 39.03% | 90.81 |
| >gi|338202359|ref|NC_015697.1| Lactobacillus reuteri SD2112 chromosome, complete genome | 715 | 39.03% | 87.69 |
| >gi|384141246|ref|NC_017171.1| Acinetobacter baumannii MDR-ZJ06 chromosome, complete genome | 1526 | 39.04% | 90.96 |
| >gi|307149679|ref|NC_014503.1| Cyanothece sp. PCC 7822 plasmid Cy782204, complete sequence | 23 | 39.04% | 82.61 |
| >gi|215481761|ref|NC_011595.1| Acinetobacter baumannii AB307-0294, complete genome | 1484 | 39.04% | 91.31 |
| >gi|384453056|ref|NC_017291.1| Chlamydophila psittaci C19/98 chromosome, complete genome | 793 | 39.05% | 86.38 |
| >gi|386085705|ref|NC_017563.1| Streptococcus thermophilus ND03 chromosome, complete genome | 764 | 39.05% | 87.83 |
| >gi|512657636|ref|NC_021504.1| Lactobacillus reuteri I5007 plasmid pLRI04, complete sequence | 20 | 39.05% | 85.00 |
| >gi|384452082|ref|NC_017290.1| Chlamydophila psittaci 08DC60 chromosome, complete genome | 795 | 39.05% | 86.29 |
| >gi|406593966|ref|NC_018627.1| Chlamydia psittaci MN chromosome, complete genome | 803 | 39.06% | 86.30 |
| >gi|384454035|ref|NC_017292.1| Chlamydophila psittaci 02DC15 chromosome, complete genome | 796 | 39.06% | 86.31 |
| >gi|384451106|ref|NC_017289.1| Chlamydophila psittaci 01DC11 chromosome, complete genome | 796 | 39.06% | 86.31 |
| >gi|406591804|ref|NC_018625.1| Chlamydia psittaci CP3 chromosome, complete genome | 808 | 39.06% | 85.89 |
| >gi|386745526|ref|NC_017733.1| Helicobacter pylori HUP-B14 chromosome, complete genome | 1560 | 39.06% | 88.01 |
| >gi|449070641|ref|NC_020248.1| Chlamydophila psittaci Mat116, complete genome | 784 | 39.06% | 86.35 |
| >gi|387908808|ref|NC_017927.1| Streptococcus thermophilus MN-ZLW-002 chromosome, complete genome | 767 | 39.06% | 87.74 |
| >gi|332286959|ref|NC_015470.1| Chlamydophila psittaci 6BC chromosome, complete genome | 793 | 39.06% | 86.25 |
| >gi|407453476|ref|NC_018619.1| Chlamydia psittaci 84/55 chromosome, complete genome | 790 | 39.06% | 86.46 |
| >gi|384450095|ref|NC_017287.1| Chlamydophila psittaci 6BC chromosome, complete genome | 793 | 39.06% | 86.25 |
| >gi|565636615|ref|NC_023028.1| Acinetobacter baumannii ZW85-1, complete genome | 1441 | 39.07% | 90.77 |
| >gi|407454812|ref|NC_018620.1| Chlamydia psittaci GR9 chromosome, complete genome | 795 | 39.08% | 85.66 |
| >gi|407460163|ref|NC_018624.1| Chlamydia psittaci WC chromosome, complete genome | 795 | 39.08% | 86.16 |
| >gi|116626972|ref|NC_008532.1| Streptococcus thermophilus LMD-9, complete genome | 757 | 39.08% | 87.19 |
| >gi|55821993|ref|NC_006449.1| Streptococcus thermophilus CNRZ1066 chromosome, complete genome | 758 | 39.08% | 87.20 |
| >gi|108562424|ref|NC_008086.1| Helicobacter pylori HPAG1 chromosome, complete genome | 1492 | 39.08% | 87.87 |
| >gi|296100281|ref|NC_014133.1| Leuconostoc kimchii IMSNU 11154 plasmid LkipL4719, complete sequence | 9 | 39.09% | 88.89 |
| >gi|55820103|ref|NC_006448.1| Streptococcus thermophilus LMG 18311 chromosome, complete genome | 727 | 39.09% | 87.35 |
| >gi|307591289|ref|NC_014534.1| Cyanothece sp. PCC 7822 plasmid Cy782202, complete sequence | 222 | 39.10% | 89.19 |
| >gi|440685051|ref|NC_019772.1| Anabaena cylindrica PCC 7122 plasmid pANACY.01, complete sequence | 40 | 39.10% | 90.00 |
| >gi|479213596|ref|NC_021044.1| Eubacterium rectale M104/1 draft genome | 1046 | 39.10% | 89.87 |
| >gi|56707107|ref|NC_006529.1| Lactobacillus salivarius UCC118 plasmid pSF118-20, complete sequence | 7 | 39.11% | 85.71 |
| >gi|387122089|ref|NC_017847.1| Acinetobacter baumannii MDR-TJ chromosome, complete genome | 1540 | 39.11% | 91.23 |
| >gi|384893616|ref|NC_017359.1| Helicobacter pylori Sat464 chromosome, complete genome | 1390 | 39.12% | 89.42 |
| >gi|385218266|ref|NC_017371.1| Helicobacter pylori Gambia94/24 chromosome, complete genome | 1581 | 39.13% | 87.48 |
| >gi|385777386|ref|NC_017304.1| Clostridium thermocellum DSM 1313 chromosome, complete genome | 1653 | 39.15% | 89.84 |
| >gi|336475959|ref|NC_015676.1| Methanosalsum zhilinae DSM 4017 chromosome, complete genome | 662 | 39.16% | 91.09 |
| >gi|289595678|ref|NC_013926.1| Aciduliprofundum boonei T469 chromosome, complete genome | 509 | 39.16% | 86.05 |
| >gi|254778738|ref|NC_012973.1| Helicobacter pylori B38 chromosome, complete genome | 1446 | 39.16% | 88.11 |
| >gi|328946930|ref|NC_015385.1| Treponema succinifaciens DSM 2489 chromosome, complete genome | 2528 | 39.16% | 88.61 |
| >gi|384129960|ref|NC_017162.1| Acinetobacter baumannii 1656-2 chromosome, complete genome | 1498 | 39.17% | 90.79 |
| >gi|15611071|ref|NC_000921.1| Helicobacter pylori J99 chromosome, complete genome | 1535 | 39.19% | 87.10 |
| >gi|169632029|ref|NC_010400.1| Acinetobacter baumannii SDF chromosome, complete genome | 1062 | 39.19% | 91.43 |
| >gi|213155370|ref|NC_011586.1| Acinetobacter baumannii AB0057 chromosome, complete genome | 1496 | 39.21% | 90.71 |
| >gi|29839769|ref|NC_003361.3| Chlamydophila caviae GPIC chromosome, complete genome | 814 | 39.22% | 85.38 |
| >gi|209693634|ref|NC_011312.1| Aliivibrio salmonicida LFI1238 chromosome chromosome 1, complete sequence | 728 | 39.23% | 90.52 |
| >gi|338202351|ref|NC_015699.1| Lactobacillus reuteri SD2112 plasmid pLR580, complete sequence | 1 | 39.24% | 100.00 |
| >gi|425790179|ref|NC_019563.1| Helicobacter pylori Aklavik86 chromosome, complete genome | 1311 | 39.25% | 89.02 |
| >gi|256823861|ref|NC_013167.1| Cyanothece sp. PCC 8802 plasmid pP880203, complete sequence | 8 | 39.26% | 100.00 |
| >gi|397691844|ref|NC_018219.1| Candidatus Mycoplasma haemolamae str. Purdue chromosome, complete genome | 377 | 39.27% | 83.02 |
| >gi|189913173|ref|NC_010843.1| Leptospira biflexa serovar Patoc strain 'Patoc 1 (Paris)' chromosome II, complete sequence | 150 | 39.27% | 92.00 |
| >gi|189912848|ref|NC_010845.1| Leptospira biflexa serovar Patoc strain 'Patoc 1 (Ames)' chromosome II, complete sequence | 150 | 39.27% | 92.00 |
| >gi|73667559|ref|NC_007355.1| Methanosarcina barkeri str. Fusaro, complete genome | 2338 | 39.28% | 86.18 |
| >gi|434408264|ref|NC_019765.1| Stanieria cyanosphaera PCC 7437 plasmid pSTA7437.01, complete sequence | 97 | 39.28% | 90.72 |
| >gi|385230889|ref|NC_017381.1| Helicobacter pylori 2018 chromosome, complete genome | 1444 | 39.29% | 88.30 |
| >gi|384890373|ref|NC_017357.1| Helicobacter pylori 908 chromosome, complete genome | 1437 | 39.30% | 88.24 |
| >gi|385223048|ref|NC_017374.1| Helicobacter pylori 2017 chromosome, complete genome | 1444 | 39.30% | 88.23 |
| >gi|29648114|ref|NC_004704.1| Coxiella burnetii RSA 493 plasmid pQpH1, complete sequence | 16 | 39.31% | 87.50 |
| >gi|161789063|ref|NC_010115.1| Coxiella burnetii RSA 331 plasmid QpH1, complete sequence | 15 | 39.33% | 86.67 |
| >gi|379009891|ref|NC_016894.1| Acetobacterium woodii DSM 1030 chromosome, complete genome | 1607 | 39.34% | 89.05 |
| >gi|218442305|ref|NC_011732.1| Cyanothece sp. PCC 7424 plasmid pP742404, complete sequence | 10 | 39.37% | 90.00 |
| >gi|77361923|ref|NC_007482.1| Pseudoalteromonas haloplanktis TAC125 chromosome II, complete sequence | 134 | 39.37% | 94.78 |
| >gi|89897807|ref|NC_007899.1| Chlamydophila felis Fe/C-56, complete genome | 806 | 39.38% | 85.86 |
| >gi|428308168|ref|NC_019754.1| Crinalium epipsammum PCC 9333 plasmid pCRI9333.03, complete sequence | 16 | 39.38% | 93.75 |
| >gi|428303471|ref|NC_019727.1| Calothrix sp. PCC 6303 plasmid pCAL6303.01, complete sequence | 24 | 39.38% | 91.67 |
| >gi|169794206|ref|NC_010410.1| Acinetobacter baumannii AYE chromosome, complete genome | 1492 | 39.38% | 90.95 |
| >gi|385839613|ref|NC_017479.1| Lactobacillus salivarius CECT 5713 plasmid pHN1, complete sequence | 10 | 39.38% | 80.00 |
| >gi|501148328|ref|NC_021233.1| Lactobacillus plantarum subsp. plantarum P-8 plasmid LBPp1, complete sequence | 18 | 39.41% | 88.89 |
| >gi|428223448|ref|NC_019692.1| Synechococcus sp. PCC 7502 plasmid pSYN7502.02, complete sequence | 0 | 39.41% | 0.00 |
| >gi|157163852|ref|NC_009802.1| Campylobacter concisus 13826, complete genome | 1146 | 39.43% | 89.53 |
| >gi|381280317|ref|NC_017018.1| Pediococcus claussenii ATCC BAA-344 plasmid pPECL-7, complete sequence | 6 | 39.45% | 83.33 |
| >gi|182682970|ref|NC_010582.1| Streptococcus pneumoniae CGSP14 chromosome, complete genome | 1022 | 39.46% | 86.30 |
| >gi|256818682|ref|NC_013160.1| Cyanothece sp. PCC 8802 plasmid pP880201, complete sequence | 36 | 39.46% | 75.00 |
| >gi|408400746|ref|NC_018712.1| Streptococcus dysgalactiae subsp. equisimilis RE378, complete genome | 934 | 39.49% | 87.58 |
| >gi|221230948|ref|NC_011900.1| Streptococcus pneumoniae ATCC 700669, complete genome | 1012 | 39.49% | 86.86 |
| >gi|54307228|ref|NC_006375.1| Lactobacillus plantarum WCFS1 plasmid pWCFS101, complete sequence | 1 | 39.49% | 100.00 |
| >gi|386315972|ref|NC_017567.1| Streptococcus dysgalactiae subsp. equisimilis ATCC 12394 chromosome, complete genome | 931 | 39.50% | 87.00 |
| >gi|528838742|ref|NC_021912.1| Lactobacillus plantarum ZJ316 plasmid pLP-ZJ103, complete sequence | 8 | 39.50% | 87.50 |
| >gi|392423388|ref|NC_018066.1| Desulfosporosinus acidiphilus SJ4 plasmid pDESACI.01, complete sequence | 18 | 39.51% | 83.33 |
| >gi|307066636|ref|NC_014494.1| Streptococcus pneumoniae AP200 chromosome, complete genome | 964 | 39.51% | 85.79 |
| >gi|410475456|ref|NC_019042.1| Streptococcus dysgalactiae subsp. equisimilis AC-2713, complete genome | 917 | 39.52% | 88.00 |
| >gi|472279618|ref|NC_020821.1| Lactobacillus brevis KB290 plasmid pKB290-2 DNA, complete genome | 3 | 39.55% | 100.00 |
| >gi|239812973|ref|NC_012790.1| Geobacillus sp. WCH70 plasmid pWCH7002, complete sequence | 22 | 39.55% | 77.27 |
| >gi|239828713|ref|NC_012794.1| Geobacillus sp. WCH70 plasmid pWCH7001, complete sequence | 15 | 39.56% | 86.67 |
| >gi|345428590|ref|NC_015964.1| Haemophilus parainfluenzae T3T1, complete genome | 821 | 39.57% | 90.38 |
| >gi|549700897|ref|NC_022532.1| Streptococcus dysgalactiae subsp. equisimilis 167 DNA, complete genome | 881 | 39.57% | 87.17 |
| >gi|251781468|ref|NC_012891.1| Streptococcus dysgalactiae subsp. equisimilis GGS_124 chromosome 1, complete sequence | 860 | 39.58% | 88.72 |
| >gi|56707135|ref|NC_006530.1| Lactobacillus salivarius UCC118 plasmid pSF118-44, complete sequence | 10 | 39.58% | 80.00 |
| >gi|256818848|ref|NC_013162.1| Capnocytophaga ochracea DSM 7271 chromosome, complete genome | 1631 | 39.59% | 83.81 |
| >gi|479134147|ref|NC_021003.1| Streptococcus pneumoniae SPN032672 draft genome | 969 | 39.59% | 85.96 |
| >gi|479134154|ref|NC_021004.1| Streptococcus pneumoniae SPN033038 draft genome | 970 | 39.59% | 85.98 |
| >gi|307126151|ref|NC_014498.1| Streptococcus pneumoniae 670-6B chromosome, complete genome | 1009 | 39.59% | 86.52 |
| >gi|428308241|ref|NC_019756.1| Crinalium epipsammum PCC 9333 plasmid pCRI9333.08, complete sequence | 1 | 39.59% | 100.00 |
| >gi|472279679|ref|NC_020823.1| Lactobacillus brevis KB290 plasmid pKB290-5 DNA, complete genome | 2 | 39.62% | 100.00 |
| >gi|387758384|ref|NC_017593.1| Streptococcus pneumoniae INV200, complete genome | 932 | 39.62% | 85.73 |
| >gi|169832377|ref|NC_010380.1| Streptococcus pneumoniae Hungary19A-6, complete genome | 997 | 39.63% | 87.16 |
| >gi|194396645|ref|NC_011072.1| Streptococcus pneumoniae G54 chromosome, complete genome | 952 | 39.64% | 86.66 |
| >gi|387625543|ref|NC_017591.1| Streptococcus pneumoniae INV104, complete genome | 971 | 39.64% | 86.10 |
| >gi|145294016|ref|NC_009344.1| Shigella dysenteriae Sd197 plasmid pSD197_spA, complete sequence | 5 | 39.65% | 80.00 |
| >gi|377549525|ref|NC_016834.1| Shigella sonnei 53G plasmid E, complete sequence | 5 | 39.65% | 80.00 |
| >gi|212208405|ref|NC_011526.1| Coxiella burnetii CbuK_Q154 plasmid pQpRS_K_Q154, complete sequence | 17 | 39.66% | 82.35 |
| >gi|479181986|ref|NC_021024.1| Butyrate-producing bacterium SM4/1, complete genome | 519 | 39.66% | 79.38 |
| >gi|472279720|ref|NC_020825.1| Lactobacillus brevis KB290 plasmid pKB290-9 DNA, complete genome | 1 | 39.69% | 100.00 |
| >gi|313652076|ref|NC_014750.1| Marivirga tractuosa DSM 4126 plasmid pFTRAC01, complete sequence | 2 | 39.69% | 100.00 |
| >gi|152989753|ref|NC_009662.1| Nitratiruptor sp. SB155-2, complete genome | 1382 | 39.69% | 87.41 |
| >gi|194172857|ref|NC_003028.3| Streptococcus pneumoniae TIGR4 chromosome, complete genome | 957 | 39.70% | 86.73 |
| >gi|116515308|ref|NC_008533.1| Streptococcus pneumoniae D39 chromosome, complete genome | 909 | 39.71% | 86.69 |
| >gi|209395638|ref|NC_011351.1| Escherichia coli O157:H7 str. EC4115 plasmid pEC4115, complete sequence | 23 | 39.71% | 86.96 |
| >gi|15902044|ref|NC_003098.1| Streptococcus pneumoniae R6 chromosome, complete genome | 905 | 39.72% | 86.74 |
| >gi|225857809|ref|NC_012468.1| Streptococcus pneumoniae 70585, complete genome | 977 | 39.73% | 87.10 |
| >gi|387787130|ref|NC_017769.1| Streptococcus pneumoniae ST556 chromosome, complete genome | 929 | 39.74% | 87.08 |
| >gi|434380362|ref|NC_018517.1| Bacillus thuringiensis HD-789 plasmid p04, complete sequence | 2 | 39.74% | 100.00 |
| >gi|566002026|ref|NC_023044.1| Methanobacterium sp. MB1 complete sequence | 828 | 39.74% | 86.84 |
| >gi|225853611|ref|NC_012466.1| Streptococcus pneumoniae JJA, complete genome | 913 | 39.74% | 85.98 |
| >gi|225855735|ref|NC_012467.1| Streptococcus pneumoniae P1031, complete genome | 938 | 39.75% | 85.93 |
| >gi|138898339|ref|NC_009329.1| Geobacillus thermodenitrificans NG80-2 plasmid pLW1071, complete sequence | 20 | 39.75% | 85.00 |
| >gi|556560056|ref|NC_022655.1| Streptococcus pneumoniae A026 genome | 905 | 39.76% | 86.96 |
| >gi|218244892|ref|NC_011726.1| Cyanothece sp. PCC 8801 chromosome, complete genome | 1268 | 39.76% | 91.01 |
| >gi|225860012|ref|NC_012469.1| Streptococcus pneumoniae Taiwan19F-14 chromosome, complete genome | 914 | 39.77% | 86.98 |
| >gi|513843967|ref|NC_021518.1| Lactobacillus plantarum 16 plasmid Lp16F, complete sequence | 15 | 39.78% | 93.33 |
| >gi|501148388|ref|NC_021234.1| Lactobacillus plantarum subsp. plantarum P-8 plasmid LBPp4, complete sequence | 4 | 39.79% | 100.00 |
| >gi|405759923|ref|NC_018594.1| Streptococcus pneumoniae SPNA45, complete genome | 958 | 39.79% | 86.12 |
| >gi|154688247|ref|NC_009726.1| Coxiella burnetii Dugway 5J108-111 plasmid pQpDG, complete sequence | 26 | 39.79% | 80.77 |
| >gi|342162671|ref|NC_015875.1| Streptococcus pseudopneumoniae IS7493 chromosome, complete genome | 1000 | 39.79% | 86.70 |
| >gi|428296779|ref|NC_019751.1| Calothrix sp. PCC 6303 chromosome, complete genome | 1488 | 39.80% | 90.59 |
| >gi|410475457|ref|NC_018630.1| Streptococcus pneumoniae gamPNI0373 chromosome, complete genome | 895 | 39.81% | 85.59 |
| >gi|387756560|ref|NC_017592.1| Streptococcus pneumoniae OXC141, complete genome | 942 | 39.81% | 86.94 |
| >gi|480535788|ref|NC_021028.1| Streptococcus pneumoniae SPN034183 draft genome | 942 | 39.81% | 86.94 |
| >gi|315122943|ref|NC_014800.1| Pseudoalteromonas sp. SM9913 chromosome II, complete sequence | 142 | 39.82% | 93.66 |
| >gi|428308292|ref|NC_019734.1| Crinalium epipsammum PCC 9333 plasmid pCRI9333.02, complete sequence | 20 | 39.82% | 85.00 |
| >gi|257057919|ref|NC_013161.1| Cyanothece sp. PCC 8802 chromosome, complete genome | 1245 | 39.82% | 90.28 |
| >gi|480533968|ref|NC_021026.1| Streptococcus pneumoniae SPN994038 draft genome | 938 | 39.83% | 86.99 |
| >gi|480530348|ref|NC_021005.1| Streptococcus pneumoniae SPN994039 draft genome | 938 | 39.83% | 86.99 |
| >gi|298501502|ref|NC_014251.1| Streptococcus pneumoniae TCH8431/19A chromosome, complete genome | 914 | 39.84% | 86.76 |
| >gi|284162948|ref|NC_013742.1| Archaeoglobus profundus DSM 5631 plasmid pArcpr01, complete sequence | 4 | 39.84% | 75.00 |
| >gi|480532168|ref|NC_021006.1| Streptococcus pneumoniae SPN034156 draft genome | 926 | 39.85% | 86.72 |
| >gi|513843219|ref|NC_021516.1| Lactobacillus plantarum 16 plasmid Lp16C, complete sequence | 1 | 39.86% | 100.00 |
| >gi|62184647|ref|NC_004552.2| Chlamydophila abortus S26/3, complete genome | 712 | 39.87% | 85.25 |
| >gi|340397867|ref|NC_015760.1| Streptococcus salivarius CCHSS3, complete genome | 870 | 39.87% | 87.36 |
| >gi|332288085|ref|NC_015460.1| Gallibacterium anatis UMN179 chromosome, complete genome | 1209 | 39.88% | 89.16 |
| >gi|13540831|ref|NC_002689.2| Thermoplasma volcanium GSS1 chromosome, complete genome | 369 | 39.92% | 89.43 |
| >gi|387760314|ref|NC_017594.1| Streptococcus salivarius 57.I chromosome, complete genome | 834 | 39.93% | 87.41 |
| >gi|187250423|ref|NC_010644.1| Elusimicrobium minutum Pei191 chromosome, complete genome | 1627 | 39.95% | 88.14 |
| >gi|338202319|ref|NC_015700.1| Lactobacillus reuteri SD2112 plasmid pLR581, complete sequence | 8 | 39.96% | 87.50 |
| >gi|188535911|ref|NC_010696.1| Erwinia tasmaniensis Et1/99 plasmid pET35, complete sequence | 21 | 39.98% | 76.19 |
| >gi|377822306|ref|NC_016807.1| Mycoplasma pneumoniae 309, complete genome | 443 | 39.98% | 84.42 |
| >gi|289166909|ref|NC_013853.1| Streptococcus mitis B6, complete genome | 926 | 39.98% | 85.42 |
| >gi|219870279|ref|NC_011852.1| Haemophilus parasuis SH0165 chromosome, complete genome | 962 | 39.99% | 85.24 |
| >gi|381184517|ref|NC_016607.1| Pediococcus claussenii ATCC BAA-344 plasmid pPECL-4, complete sequence | 8 | 39.99% | 100.00 |
| >gi|385326614|ref|NC_017504.1| Mycoplasma pneumoniae FH chromosome, complete genome | 438 | 40.00% | 84.70 |
| >gi|13507739|ref|NC_000912.1| Mycoplasma pneumoniae M129 chromosome, complete genome | 445 | 40.01% | 84.49 |
| >gi|479052799|ref|NC_020076.1| Mycoplasma pneumoniae M129-B7, complete genome | 447 | 40.01% | 84.56 |
| >gi|302669123|ref|NC_014388.1| Butyrivibrio proteoclasticus B316 chromosome 2, complete genome | 115 | 40.04% | 86.96 |
| >gi|514058762|ref|NC_021521.1| Haemophilus parasuis ZJ0906, complete genome | 966 | 40.06% | 86.44 |
| >gi|387783149|ref|NC_017595.1| Streptococcus salivarius JIM8777, complete genome | 862 | 40.07% | 87.59 |
| >gi|215778350|ref|NC_011311.1| Aliivibrio salmonicida LFI1238 plasmid pVSAL840, complete sequence | 32 | 40.07% | 93.75 |
| >gi|119943794|ref|NC_008709.1| Psychromonas ingrahamii 37 chromosome, complete genome | 1484 | 40.09% | 90.77 |
| >gi|440685330|ref|NC_019774.1| Anabaena cylindrica PCC 7122 plasmid pANACY.04, complete sequence | 23 | 40.09% | 86.96 |
| >gi|479155735|ref|NC_021015.1| Ruminococcus torques L2-14 draft genome | 1301 | 40.14% | 84.09 |
| >gi|209401009|ref|NC_011352.1| Lactobacillus casei str. Zhang plasmid plca36, complete sequence | 5 | 40.15% | 80.00 |
| >gi|428303693|ref|NC_019753.1| Crinalium epipsammum PCC 9333 chromosome, complete genome | 1690 | 40.16% | 82.43 |
| >gi|116329556|ref|NC_008509.1| Leptospira borgpetersenii serovar Hardjo-bovis L550 chromosome chromosome 2, complete sequence | 220 | 40.16% | 86.36 |
| >gi|528821545|ref|NC_021903.1| Lactobacillus plantarum ZJ316 plasmid pLP-ZJ101, complete sequence | 6 | 40.17% | 100.00 |
| >gi|218442342|ref|NC_011734.1| Cyanothece sp. PCC 7424 plasmid pP742406, complete sequence | 10 | 40.20% | 70.00 |
| >gi|242397997|ref|NC_012883.1| Thermococcus sibiricus MM 739, complete genome | 731 | 40.20% | 82.90 |
| >gi|302669374|ref|NC_014387.1| Butyrivibrio proteoclasticus B316 chromosome 1, complete genome | 1262 | 40.21% | 86.85 |
| >gi|383309625|ref|NC_017027.1| Pasteurella multocida subsp. multocida str. HN06 chromosome, complete genome | 857 | 40.22% | 89.26 |
| >gi|307149945|ref|NC_014501.1| Cyanothece sp. PCC 7822 chromosome, complete genome | 2506 | 40.22% | 86.79 |
| >gi|153930562|ref|NC_009704.1| Yersinia pseudotuberculosis IP 31758 plasmid_59kb, complete sequence | 9 | 40.22% | 88.89 |
| >gi|17158637|ref|NC_003240.1| Nostoc sp. PCC 7120 plasmid pCC7120beta, complete sequence | 45 | 40.23% | 91.11 |
| >gi|116329799|ref|NC_008510.1| Leptospira borgpetersenii serovar Hardjo-bovis str. JB197 chromosome 1, complete sequence | 2720 | 40.23% | 85.85 |
| >gi|218203933|ref|NC_011723.1| Cyanothece sp. PCC 8801 plasmid pP880102, complete sequence | 26 | 40.23% | 92.31 |
| >gi|375010684|ref|NC_016599.1| Owenweeksia hongkongensis DSM 17368 chromosome, complete genome | 1188 | 40.23% | 89.48 |
| >gi|77358982|ref|NC_007481.1| Pseudoalteromonas haloplanktis TAC125 chromosome I, complete sequence | 573 | 40.23% | 91.45 |
| >gi|116326852|ref|NC_008508.1| Leptospira borgpetersenii serovar Hardjo-bovis str. L550 chromosome 1, complete sequence | 2789 | 40.23% | 85.80 |
| >gi|407691594|ref|NC_018690.1| Actinobacillus suis H91-0380 chromosome, complete genome | 882 | 40.24% | 86.17 |
| >gi|54307144|ref|NC_006373.1| Bacteroides uniformis mobilizable transposon NBU1, complete sequence | 4 | 40.24% | 100.00 |
| >gi|313669445|ref|NC_014755.1| Sulfuricurvum kujiense DSM 16994 plasmid pSULKU02, complete sequence | 25 | 40.26% | 88.00 |
| >gi|126090338|ref|NC_009038.1| Shewanella baltica OS155 plasmid pSbal04, complete sequence | 5 | 40.26% | 80.00 |
| >gi|316994385|ref|NC_013791.2| Bacillus pseudofirmus OF4 chromosome, complete genome | 1125 | 40.27% | 87.82 |
| >gi|386833612|ref|NC_017764.1| Pasteurella multocida subsp. multocida str. 3480 chromosome, complete genome | 830 | 40.29% | 88.07 |
| >gi|549700063|ref|NC_022531.1| Bacillus amyloliquefaciens subsp. plantarum NAU-B3 plasmid pBamNAU-B3a | 3 | 40.32% | 100.00 |
| >gi|238897251|ref|NC_012751.1| Candidatus Hamiltonella defensa 5AT (Acyrthosiphon pisum) chromosome, complete genome | 1688 | 40.32% | 88.51 |
| >gi|338729812|ref|NC_015707.1| Thermotoga thermarum DSM 5069 chromosome, complete genome | 1011 | 40.32% | 86.15 |
| >gi|29337300|ref|NC_002620.2| Chlamydia muridarum Nigg, complete genome | 934 | 40.34% | 87.47 |
| >gi|324959701|ref|NC_015166.1| Bacteroides salanitronis DSM 18170 plasmid pBACSA03, complete sequence | 1 | 40.34% | 100.00 |
| >gi|470180510|ref|NC_020522.1| Legionella pneumophila subsp. pneumophila LPE509 plasmid unnamed, complete sequence | 16 | 40.34% | 100.00 |
| >gi|428303659|ref|NC_019737.1| Crinalium epipsammum PCC 9333 plasmid pCRI9333.07, complete sequence | 3 | 40.34% | 100.00 |
| >gi|479140210|ref|NC_021010.1| Eubacterium rectale DSM 17629 draft genome | 872 | 40.35% | 88.88 |
| >gi|153930630|ref|NC_009705.1| Yersinia pseudotuberculosis IP 31758 plasmid_153kb, complete sequence | 41 | 40.35% | 92.68 |
| >gi|427705465|ref|NC_019676.1| Nostoc sp. PCC 7107 chromosome, complete genome | 1547 | 40.36% | 89.14 |
| >gi|315125111|ref|NC_014803.1| Pseudoalteromonas sp. SM9913 chromosome I, complete sequence | 639 | 40.38% | 90.77 |
| >gi|15601865|ref|NC_002663.1| Pasteurella multocida subsp. multocida str. Pm70 chromosome, complete genome | 787 | 40.40% | 89.45 |
| >gi|330370681|ref|NC_015429.1| Lactobacillus buchneri NRRL B-30929 plasmid pLBUC02, complete sequence | 8 | 40.41% | 87.50 |
| >gi|312128809|ref|NC_014655.1| Leadbetterella byssophila DSM 17132 chromosome, complete genome | 1555 | 40.41% | 86.69 |
| >gi|336237224|ref|NC_015661.1| Geobacillus thermoglucosidasius C56-YS93 plasmid pGEOTH02, complete sequence | 14 | 40.42% | 71.43 |
| >gi|523532970|ref|NC_021734.1| Acinetobacter baumannii BJAB0715 plasmid pBJAB0715, complete sequence | 23 | 40.43% | 86.96 |
| >gi|116332445|ref|NC_008511.1| Leptospira borgpetersenii serovar Hardjo-bovis JB197 chromosome chromosome 2, complete sequence | 202 | 40.43% | 87.62 |
| >gi|50083297|ref|NC_005966.1| Acinetobacter sp. ADP1 chromosome, complete genome | 965 | 40.43% | 91.09 |
| >gi|148641539|ref|NC_009517.1| Psychrobacter sp. PRwf-1 plasmid pRWF102, complete sequence | 0 | 40.43% | 0.00 |
| >gi|378773696|ref|NC_016808.1| Pasteurella multocida 36950 chromosome, complete genome | 775 | 40.44% | 89.03 |
| >gi|218249153|ref|NC_011727.1| Cyanothece sp. PCC 8801 plasmid pP880103, complete sequence | 2 | 40.44% | 100.00 |
| >gi|451348611|ref|NC_020273.1| Bacillus amyloliquefaciens IT-45 plasmid pBA45-1, whole genome shotgun sequence | 8 | 40.47% | 87.50 |
| >gi|253825511|ref|NC_012961.1| Photorhabdus asymbiotica plasmid pPAU1, complete sequence | 10 | 40.50% | 80.00 |
| >gi|157149651|ref|NC_009785.1| Streptococcus gordonii str. Challis substr. CH1 chromosome, complete genome | 1095 | 40.51% | 87.40 |
| >gi|186686738|ref|NC_010631.1| Nostoc punctiforme PCC 73102 plasmid pNPUN01, complete sequence | 63 | 40.51% | 88.89 |
| >gi|532360755|ref|NC_022114.1| Lactobacillus paracasei subsp. paracasei 8700:2 plasmid 1, complete sequence | 1 | 40.51% | 100.00 |
| >gi|17233017|ref|NC_003276.1| Nostoc sp. PCC 7120 plasmid pCC7120alpha, complete sequence | 77 | 40.51% | 92.21 |
| >gi|75812284|ref|NC_007410.1| Anabaena variabilis ATCC 29413 plasmid A, complete sequence | 65 | 40.53% | 96.92 |
| >gi|387873357|ref|NC_017903.1| Escherichia coli Xuzhou21 plasmid pO157_Sal, complete sequence | 12 | 40.55% | 83.33 |
| >gi|384448988|ref|NC_017285.1| Chlamydophila pneumoniae LPCoLN chromosome, complete genome | 846 | 40.55% | 86.76 |
| >gi|428303616|ref|NC_019735.1| Crinalium epipsammum PCC 9333 plasmid pCRI9333.04, complete sequence | 10 | 40.56% | 100.00 |
| >gi|58021288|ref|NC_002179.2| Chlamydophila pneumoniae AR39, complete genome | 833 | 40.57% | 87.52 |
| >gi|33241335|ref|NC_005043.1| Chlamydophila pneumoniae TW-183, complete genome | 827 | 40.58% | 87.79 |
| >gi|15617929|ref|NC_000922.1| Chlamydophila pneumoniae CWL029 chromosome, complete genome | 837 | 40.58% | 87.81 |
| >gi|15835535|ref|NC_002491.1| Chlamydophila pneumoniae J138 chromosome, complete genome | 832 | 40.58% | 87.86 |
| >gi|377808757|ref|NC_016608.1| Pediococcus claussenii ATCC BAA-344 plasmid pPECL-5, complete sequence | 4 | 40.60% | 100.00 |
| >gi|313669332|ref|NC_014754.1| Sulfuricurvum kujiense DSM 16994 plasmid pSULKU01, complete sequence | 51 | 40.61% | 88.24 |
| >gi|428220140|ref|NC_019702.1| Synechococcus sp. PCC 7502 chromosome, complete genome | 438 | 40.62% | 89.04 |
| >gi|9791176|ref|NC_002180.1| Chlamydia phage phiCPAR39, complete sequence | 0 | 40.62% | 0.00 |
| >gi|186687048|ref|NC_010632.1| Nostoc punctiforme PCC 73102 plasmid pNPUN02, complete sequence | 63 | 40.65% | 95.24 |
| >gi|479162165|ref|NC_021017.1| Bacteroides xylanisolvens XB1A draft genome | 1767 | 40.67% | 87.27 |
| >gi|218442241|ref|NC_011730.1| Cyanothece sp. PCC 7424 plasmid pP742403, complete sequence | 12 | 40.67% | 91.67 |
| >gi|476411542|ref|NC_020514.1| Glaciecola psychrophila 170, complete genome | 942 | 40.69% | 92.14 |
| >gi|197247299|ref|NC_011148.1| Salmonella enterica subsp. enterica serovar Agona str. SL483 plasmid unnamed, complete sequence | 9 | 40.74% | 88.89 |
| >gi|428277405|ref|NC_017194.1| Bacillus subtilis subsp. natto BEST195 plasmid pBEST195S, complete sequence | 10 | 40.75% | 80.00 |
| >gi|238909104|ref|NC_012782.1| Eubacterium eligens ATCC 27750 plasmid unnamed, complete sequence | 19 | 40.75% | 89.47 |
| >gi|91772082|ref|NC_007955.1| Methanococcoides burtonii DSM 6242, complete genome | 684 | 40.76% | 86.55 |
| >gi|18976372|ref|NC_003413.1| Pyrococcus furiosus DSM 3638 chromosome, complete genome | 601 | 40.77% | 82.03 |
| >gi|397650687|ref|NC_018092.1| Pyrococcus furiosus COM1 chromosome, complete genome | 596 | 40.79% | 82.05 |
| >gi|513845190|ref|NC_021526.1| Lactobacillus plantarum 16 plasmid Lp16D, complete sequence | 7 | 40.79% | 100.00 |
| >gi|338209457|ref|NC_015694.1| Runella slithyformis DSM 19594 plasmid pRUNSL03, complete sequence | 32 | 40.80% | 93.75 |
| >gi|54307184|ref|NC_006377.1| Lactobacillus plantarum WCFS1 plasmid pWCFS103, complete sequence | 9 | 40.83% | 100.00 |
| >gi|17227465|ref|NC_003270.1| Nostoc sp. PCC 7120 plasmid pCC7120epsilon, complete sequence | 16 | 40.86% | 87.50 |
| >gi|434389956|ref|NC_019744.1| Cylindrospermum stagnale PCC 7417 plasmid pCYLST.02, complete sequence | 29 | 40.88% | 86.21 |
| >gi|428223389|ref|NC_019691.1| Synechococcus sp. PCC 7502 plasmid pSYN7502.01, complete sequence | 13 | 40.89% | 92.31 |
| >gi|302344773|ref|NC_014370.1| Prevotella melaninogenica ATCC 25845 chromosome I, complete sequence | 723 | 40.92% | 83.82 |
| >gi|186686664|ref|NC_010630.1| Nostoc punctiforme PCC 73102 plasmid pNPUN03, complete sequence | 25 | 40.92% | 88.00 |
| >gi|428308248|ref|NC_019733.1| Crinalium epipsammum PCC 9333 plasmid pCRI9333.01, complete sequence | 17 | 40.94% | 94.12 |
| >gi|338708677|ref|NC_015715.1| Zymomonas mobilis subsp. pomaceae ATCC 29192 plasmid pZYMOP01, complete sequence | 24 | 40.96% | 83.33 |
| >gi|472335239|ref|NC_020834.1| Mannheimia haemolytica USDA-ARS-SAM-185, complete genome | 938 | 40.97% | 88.59 |
| >gi|434389900|ref|NC_019698.1| Chamaesiphon minutus PCC 6605 plasmid pCHA6605.02, complete sequence | 9 | 40.98% | 77.78 |
| >gi|386583127|ref|NC_017620.1| Streptococcus suis D9 chromosome, complete genome | 849 | 40.99% | 87.40 |
| >gi|556587607|ref|NC_022665.1| Streptococcus suis T15, complete genome | 836 | 40.99% | 88.64 |
| >gi|472332507|ref|NC_020833.1| Mannheimia haemolytica USDA-ARS-USMARC-183, complete genome | 982 | 41.01% | 88.19 |
| >gi|397677586|ref|NC_018146.1| Zymomonas mobilis subsp. mobilis ATCC 29191 plasmid pZZ6.01, complete sequence | 35 | 41.02% | 88.57 |
| >gi|17227374|ref|NC_003267.1| Nostoc sp. PCC 7120 plasmid pCC7120gamma, complete sequence | 19 | 41.03% | 100.00 |
| >gi|525701099|ref|NC_021743.1| Mannheimia haemolytica D153, complete genome | 980 | 41.04% | 88.06 |
| >gi|470165991|ref|NC_020515.1| Bibersteinia trehalosi USDA-ARS-USMARC-192, complete genome | 895 | 41.05% | 86.37 |
| >gi|482884694|ref|NC_021082.1| Mannheimia haemolytica M42548, complete genome | 983 | 41.05% | 88.61 |
| >gi|479150083|ref|NC_021013.1| Ruminococcus bromii L2-63 draft genome | 972 | 41.05% | 86.42 |
| >gi|526467231|ref|NC_021883.1| Mannheimia haemolytica USMARC_2286, complete genome | 965 | 41.06% | 88.19 |
| >gi|253754647|ref|NC_012926.1| Streptococcus suis BM407 chromosome, complete genome | 767 | 41.06% | 87.87 |
| >gi|302346166|ref|NC_014371.1| Prevotella melaninogenica ATCC 25845 chromosome II, complete sequence | 625 | 41.07% | 78.40 |
| >gi|434401190|ref|NC_019750.1| Stanieria cyanosphaera PCC 7437 plasmid pSTA7437.03, complete sequence | 26 | 41.07% | 92.31 |
| >gi|525662379|ref|NC_021739.1| Mannheimia haemolytica D174, complete genome | 975 | 41.07% | 88.21 |
| >gi|330443755|ref|NC_015408.1| Chlamydophila pecorum E58 chromosome, complete genome | 805 | 41.08% | 83.98 |
| >gi|479166807|ref|NC_021018.1| Coprococcus sp. ART55/1 draft genome | 480 | 41.08% | 86.04 |
| >gi|88607955|ref|NC_007798.1| Neorickettsia sennetsu str. Miyayama chromosome, complete genome | 296 | 41.08% | 89.86 |
| >gi|326797444|ref|NC_015277.1| Sphingobacterium sp. 21 chromosome, complete genome | 2165 | 41.10% | 87.48 |
| >gi|545632602|ref|NC_022441.1| Chlamydia pecorum P787, complete genome | 813 | 41.10% | 84.01 |
| >gi|545631676|ref|NC_022440.1| Chlamydia pecorum W73, complete genome | 805 | 41.11% | 83.85 |
| >gi|253750923|ref|NC_012924.1| Streptococcus suis SC84, complete genome | 749 | 41.11% | 88.65 |
| >gi|146317663|ref|NC_009442.1| Streptococcus suis 05ZYH33 chromosome, complete genome | 767 | 41.11% | 88.27 |
| >gi|146319850|ref|NC_009443.1| Streptococcus suis 98HAH33, complete genome | 747 | 41.11% | 88.35 |
| >gi|428308343|ref|NC_019736.1| Crinalium epipsammum PCC 9333 plasmid pCRI9333.05, complete sequence | 7 | 41.11% | 85.71 |
| >gi|427726395|ref|NC_019677.1| Nostoc sp. PCC 7524 plasmid pNOS7524.01, complete sequence | 12 | 41.12% | 83.33 |
[truncated: 446,523 more chars]
